# Adipose Tissue Deficiency and Chronic Inflammation in Diabetic Goto-Kakizaki Rats

**DOI:** 10.1371/journal.pone.0017386

**Published:** 2011-02-25

**Authors:** Bai Xue, Siddharth Sukumaran, Jing Nie, William J. Jusko, Debra C. DuBois, Richard R. Almon

**Affiliations:** 1 Department of Biological Sciences, State University of New York at Buffalo, Buffalo, New York, United States of America; 2 Department of Pharmaceutical Sciences, State University of New York at Buffalo, Buffalo, New York, United States of America; 3 New York State Center of Excellence in Bioinformatics and Life Sciences, Buffalo, New York, United States of America; University of Valencia, Spain

## Abstract

Type 2 diabetes (T2DM) is a heterogeneous group of diseases that is progressive and involves multiple tissues. Goto-Kakizaki (GK) rats are a polygenic model with elevated blood glucose, peripheral insulin resistance, a non-obese phenotype, and exhibit many degenerative changes observed in human T2DM. As part of a systems analysis of disease progression in this animal model, this study characterized the contribution of adipose tissue to pathophysiology of the disease. We sacrificed subgroups of GK rats and appropriate controls at 4, 8, 12, 16 and 20 weeks of age and carried out a gene array analysis of white adipose tissue. We expanded our physiological analysis of the animals that accompanied our initial gene array study on the livers from these animals. The expanded analysis included adipose tissue weights, HbA1c, additional hormonal profiles, lipid profiles, differential blood cell counts, and food consumption. HbA1c progressively increased in the GK animals. Altered corticosterone, leptin, and adiponectin profiles were also documented in GK animals. Gene array analysis identified 412 genes that were differentially expressed in adipose tissue of GKs relative to controls. The GK animals exhibited an age-specific failure to accumulate body fat despite their relatively higher calorie consumption which was well supported by the altered expression of genes involved in adipogenesis and lipogenesis in the white adipose tissue of these animals, including *Fasn*, *Acly*, *Kklf9*, and *Stat3*. Systemic inflammation was reflected by chronically elevated white blood cell counts. Furthermore, chronic inflammation in adipose tissue was evident from the differential expression of genes involved in inflammatory responses and activation of natural immunity, including two interferon regulated genes, *Ifit* and *Iipg*, as well as MHC class II genes. This study demonstrates an age specific failure to accumulate adipose tissue in the GK rat and the presence of chronic inflammation in adipose tissue from these animals.

## Introduction

Chronic inflammation is the single most common factor associated with the development of type 2 diabetes (T2DM). In the West, more than eighty percent of patients with T2DM have a body mass index greater than 25 kg/m^2^ and are thus considered overweight to obese (>30 kg/m^2^). Obesity is recognized as a strong risk factor for T2DM, and weight reduction often improves glycemic indices in these patients [1]. As adipose tissue expands, macrophages increasingly infiltrate the tissue [2]. It is this increased macrophage infiltration that in part suggests a link between obesity, inflammation, and the development of T2DM. However, not all type 2 diabetics are obese. In fact, in Asia where obesity is not as prevalent, about sixty percent of type 2 diabetics have body mass indexes less than 25 kg/m^2^ and are thus classified as “lean” diabetics [3]. Although in Western societies obesity is a major cause of chronic inflammation, other chronic inflammatory conditions such as periodontal disease, obstructive pulmonary disease (COPD), arthritis, and myotonic dystrophy are also associated with the development of T2DM [4]–[9]. For example, up to a third of patients with chronic hepatitis C infections develop diabetes [10], [11]. Linking these disparate conditions associated with T2DM is chronic innate immune activation.

The role of adipose tissue in energy metabolism is to store energy substrates as triglycerides during periods of nutrient excess and to release fatty acids during periods of low nutrient availability or high energy demand. Only mature adipocytes participate in this cycle of triglyceride storage and fatty acid release. In the rat, the number of mature adipocytes expands for about the first 12 weeks of life [12]. Following this initial period of mature adipocyte increase, additional expansion in the amount of adipose tissue for the most part involves an increase in the quantity of triglycerides stored in individual mature adipocytes. Studies of humans and animals subjected to metabolic overload during which caloric intake greatly exceeds caloric demand demonstrate that the amount of triglyceride storage in individual mature adipocytes can expand many-fold [13]. Mature adipocytes are also the source of several hormones collectively referred to as adipokines, which play major roles in regulating many important physiological processes including systemic energy metabolism. Only about one third of the cells in adult white adipose tissue are mature adipocytes. The remaining two-thirds of the cells are a combination of small blood vessels, nerve tissue, fibroblasts, preadipocytes, stem cells, and a substantial population of immune cells (primarily macrophages).

In the present study we analyzed the postnatal development of adipose tissue from 4 to 20 weeks after birth in two related strains of rats. The analysis included both relevant physiological variables and a time series analysis of gene expression using Affymetrix 230-2 gene arrays. One strain, the Goto-Kakizaki (GK) rat, was developed as an inbred model for T2DM from an outbred Wistar colony at Kyoto University. GKs were derived by inbreeding for high population glucose values during oral glucose tolerance tests. These rats exhibit a spontaneous form of diabetes, with elevated blood glucose, peripheral insulin resistance, and a non-obese phenotype being consistent features [14], [15]. Their polygenetic mode of inheritance together with their non-obese phenotype make this animal model a useful surrogate for the study of human T2DM without the confounding influence of obesity-related factors. This colony was established at Taconic Farms in 2005. The second strain used as controls was the Wistar-Kyoto (WKY) partially inbred strain of rat that was developed by inbreeding from the same outbred Wistar colony at Kyoto University. Taconic Farms has maintained these as a closed colony since 1974.

T2DM develops over time with the individual often being hyperinsulinemic for an extended period of time prior to becoming hyperglycemic, which occurs as beta cells begin to fail. We used a times series approach to capture the development of diabetes in the GK strain. In a previous report, we described a time series gene array analysis of the livers from these animals along with selected plasma measurements (glucose and insulin) and measurements relevant specifically to liver [16]. Those results demonstrate a disruption in lipid metabolism in the liver. In addition, there were clear indications of chronic natural immune system-mediated inflammation in the liver. In the present report, we describe a similar gene array analysis of white adipose tissue (WAT) from the same animals along with additional plasma measurements relevant to WAT including adipokines and lipid profiles, as well as additional measurements relevant to both diabetes and inflammation. The analysis of these animals shows that the lean phenotype involves a failure in the normal development of WAT with age. In addition, despite their much lower WAT mass, array results indicate the presence of chronic inflammation in adipose tissue in the absence of obesity.

## Methods

### Experimental design

A more extensive description of this experiment can be found in our previously published report describing the array results on the livers from these animals [16]. In brief, this study involved 30 GK spontaneously diabetic male rats and 30 WKY control male rats obtained from Taconic Farms (Germantown, NY). Male animals were chosen for this study to avoid potential additional animal variation that might be imposed by reproductive hormone cycling in females. Animals were housed in individual cages with free access to standard rat chow (Rodent Diet 2016, Harlan Teklad) and water. Food intake and body weights were measured twice weekly on all animals. All animal care, manipulations, and sacrifices were carried out during a discrete phase of the circadian cycle, between 1.5 and 3.5 hours after lights on. Six animals from each strain were killed at five different ages: 4, 8, 12, 16, and 20 weeks. Animals were anesthetized with ketamine (80 mg/kg)/valium (5 mg/kg) i.p. and killed by aortic exsanguinations using EDTA (4 mM final concentration) as anticoagulant in order to obtain plasma samples. Discrete abdominal fat pads (perirenal/retroperitoneal fat) as well as epididymal fat pads were harvested, weighed, rapidly frozen in liquid nitrogen, and stored at −80°C. Our research protocol adhered to the ‘Principles of Laboratory Animal Care’ (NIH publication 85-23, revised in 1985) and was approved by the University at Buffalo Institutional Animal Care and Use Committee (IACUC Approval Number BIO19116N).

### Blood Measurements

HbA1c (glycosylated hemoglobin) was measured using A1cNOW InView HbA1c test meters (Metrika, Sunnyvale, CA) from whole blood at sacrifice. Total leukocyte analysis was carried out on an automated hemocytometer (CELLDYN 1700, Abbott Laboratories, Abbott Park, IL). The system was calibrated with commercial quality controls (Abbott Laboratories) at three cell concentration levels (high, normal, and low).

### Plasma measurements

Corticosterone was measured in plasma samples using a commercial RIA (Corticosterone RIA Kit (rat and mouse), MP Biomedicals, Solon, OH), leptin by ELISA (Rat Leptin TiterZyme EIA, Assay Designs, Ann Arbor, MI), and adiponectin by ELISA (Rat Adiponectin EIA, ALPCO Diagnostics, Salem, NH). Plasma free fatty acids (FFA) were measured using Roche Half-micro Test (Roche Applied Sciences, Indianapolis, IN) modified to a microtitre plate format. A standard curve consisting of 7 concentrations of FFA ranging from 0.05–1 uM was constructed from a commercial standard solution (WAKO NEFA, WAKO Chemicals, Richmond, VA). Total cholesterol, HDL-cholesterol, LDL-cholesterol, and triglycerides were measured by colorimetric assays using reagents commercially available (WAKO Chemicals, Richmond VA) with assays adapted to a microtiter plate format [17].

### Assay design and statistical analysis

For all plasma assays, standard curve samples were run in duplicate and experimental samples in triplicate. Two experimental samples were selected as “QC standards” for all plasma assays to exclude possible experimental variations between different runs. Inter-assay variations of QCs were less than 10% for all assays. For statistical comparisons of all data, two-way ANOVAs were carried out using SigmaStat 3.5 software (Systat Software, Point Richmond, CA) with Tukey post-hoc tests on rank-transformed data.

### Microarrays

Due to the high cost, 5 of 6 samples from each experimental group were analyzed by microarrays. Abdominal fat samples from each animal were ground to a fine powder in a mortar cooled by liquid nitrogen and tissue was added to QIAzol Lysis Reagent (QIAGEN Sciences, Germantown, MD) in a ratio of 1∶10. Total RNAs were extracted according to manufacturer's directions and further purified using RNeasy mini columns (RNeasy Mini Kit, QIAGEN Sciences). Final RNA preparations were resuspended in RNase-free water and stored at −80°C. RNAs were quantified spectrophotometrically, and purity and integrity assessed by agarose gel electrophoresis. All samples exhibited 260/280 absorbance ratios of approximately 2.0, and all showed intact ribosomal 28S and 18S RNA bands in an approximate ratio of 2∶1 as visualized by ethidium bromide staining. Isolated RNA from each sample was used to prepare target according to manufacturer's protocols. The biotinylated cRNAs were hybridized to 50 individual Affymetrix GeneChips Rat Genome 230-2 (Affymetrix, Inc., Santa Clara, CA). The 230-2 chips contain more than 31,000 different probe sets. The high reproducibility of in situ synthesis of oligonucleotide chips allows accurate comparison of signals generated by samples hybridized to separate arrays.

### Data mining

Affymetrix Microarray Suite 5.0 (Affymetrix) was used for initial data acquisition and analysis. The signal intensities were normalized for each chip using a distribution of all genes around the 50th percentile. The dataset was then loaded into a data mining program, GeneSpring 7.3.1 (Silicon Genetics, Redwood City, CA), for further analysis. The generated dataset was submitted to the National Center for Biotechnology Information (NCBI) Gene Expression Omnibus (GEO; http://www.ncbi.nlm.nih.gov/projects/geo/) database (GSE 13271).

**Figure 1:**
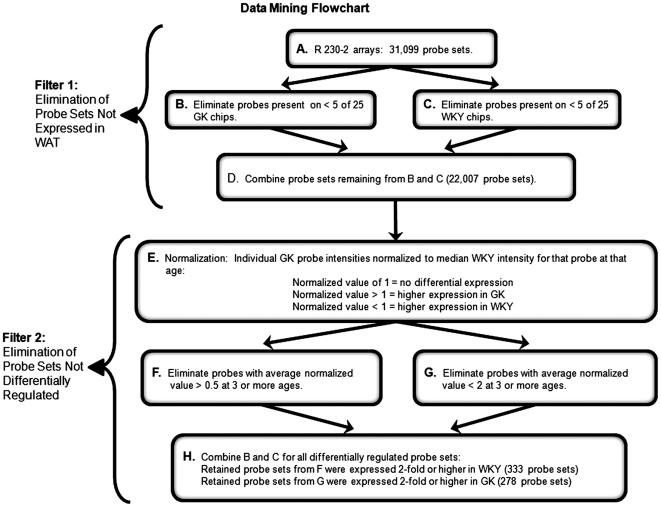
Data mining flowchart. Filters are presented for elimination of non-differentially regulated genes. Step A begins with all probe sets (31,099) normalized to the 50^th^ percentile of signals on that chip. Steps B and C employed a function of Affymetrix Microarray Suite 5.0 which scores signal intensities as present (P), absent (A), or marginal (M). Probe sets not Present on at least 5 of 25 GK chips were eliminated in B, and those not Present on at least 5 of 25 WKY chips were eliminated in C. Step D (probe sets not eliminated in B and C) represent probe sets (22,007) expressed in adipose tissue from either strain. Probe sets remaining in Step D are normalized in Step E by dividing the value of individual GK probe sets by the median value of that probe set from WKY chips. Normalized probe sets from Step E are filtered for differential expression (minimum 2-fold difference) in Steps F and G. Step H represents total probe sets (611) differentially expressed in at least 3 ages and used for further analysis.

In order to objectively identify probe sets of interest, the entire dataset was filtered with criteria similar to the ones applied to previous gene array datasets [18], [19] and identical to the approach used with the liver array results [16]. This approach does not select for probe sets but rather eliminates those probe sets that do not meet certain criteria, leaving the remainder for further consideration. In this case, probe sets that were not expressed in WAT were first eliminated, leaving a remainder of 22,007. These remaining probe sets were then filtered to eliminate those that did not exhibit at least 2-fold differences in expression in at least three ages when comparing GK and WKY. This second filtering step kept a total 611 probe sets for further investigation, 278 higher in GK rats and 333 higher in WKY rats. Filtering steps are presented in Figure 1 and in our previous publication on the livers from these animals [16].

## Results

### Differential growth in GK and WKY animals

**Figure 2:**
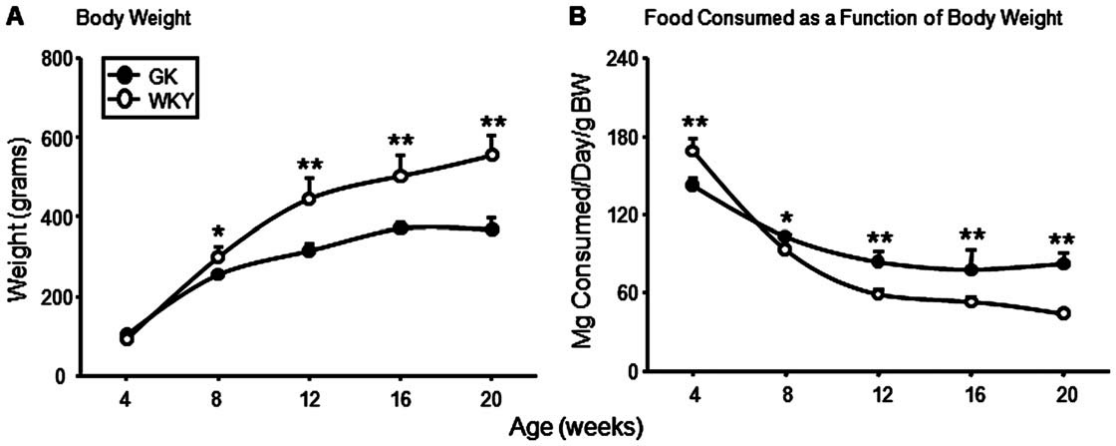
Body weights and food consumption. A) Body weight (grams) at sacrifice B) Daily food consumption (mg) adjusted for body weight (grams). Data represent means and error bars 1 SD of the mean. Closed circles = GK; open circles = WKY). * = P<0.05; ** = P<0.001.

**Figure 3:**
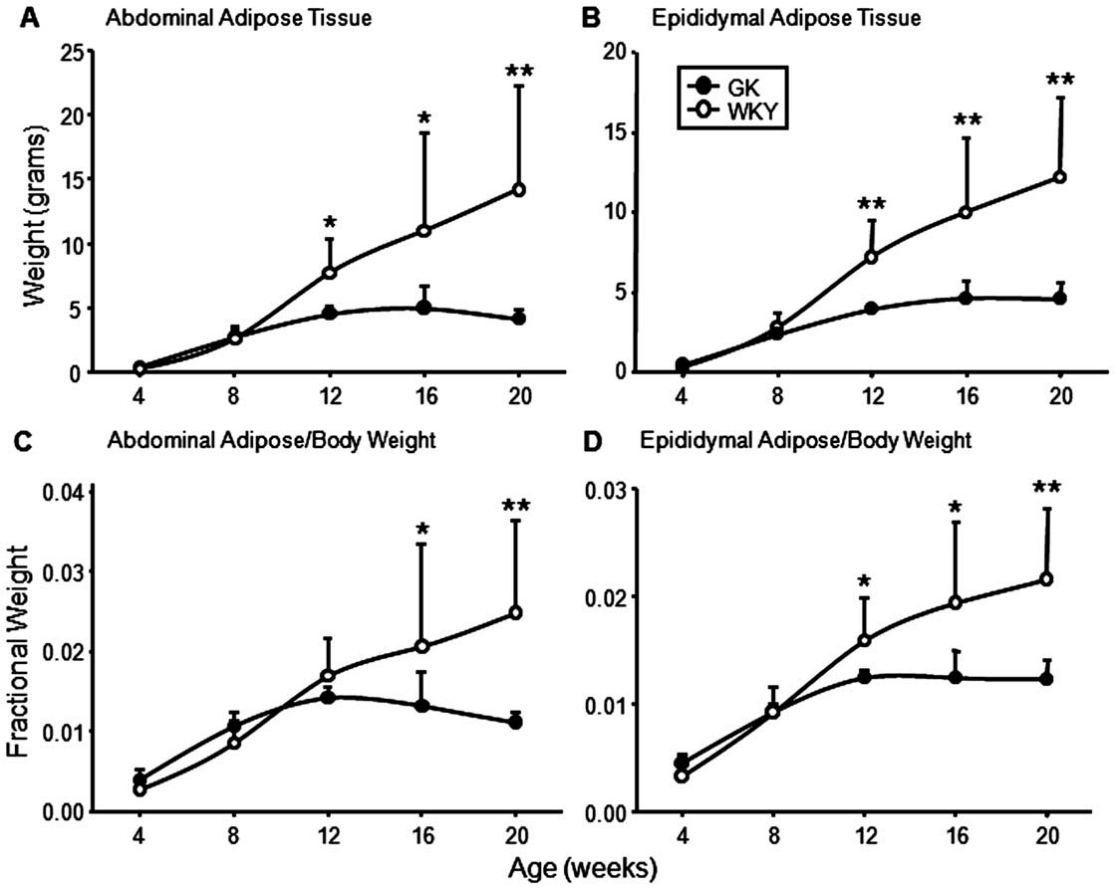
Adipose tissue weights. Weights (grams) of abdominal (A) and epididymal (B) adipose tissue at sacrifice, and abdominal (C) and epididymal (D) adipose weight adjusted for body weight as a function of age in GK and WKY rats. Data represent means and error bars 1 SD of the mean. Closed circles = GK; open circles = WKY). * = P<0.05; ** = P<0.001.

Body weights at sacrifice and food consumption throughout the study are presented in Figure 2. Although there was no difference in body weights between the two groups of animals at 4 weeks of age, WKY were significantly heavier than GK animals by 8 weeks and this difference increased through the experimental period. Until about 14 weeks of age, total grams of food consumed per day were similar among both groups and then slightly decreased in WKY. However, when food consumption was adjusted for body weight (Figure 2), GK animals actually consumed significantly more food than WKYs from 8 weeks on. Figure 3A shows the weight of abdominal fat in both groups of animals. The weight of the fat pads in WKYs increased linearly with age, while the abdominal fat pads in the GK population stopped increasing in weight between 8 and 12 weeks with a slight decline suggested after 16 weeks. The epididymal fat pad showed a similar pattern (Figure 3B). The same data expressed as a percentage of body weight show that as the animals get older adipose tissue is a greater percentage of total body weight in control WKY but not in GK animals (Figures 3C and D). This is in distinct contrast to the results obtained with the livers from these animals. Although the livers from the WKY were somewhat larger, GKs livers were a significantly larger percent of body weight from 8 weeks until the end of the experiment.

### Glucose homeostasis

**Figure 4:**
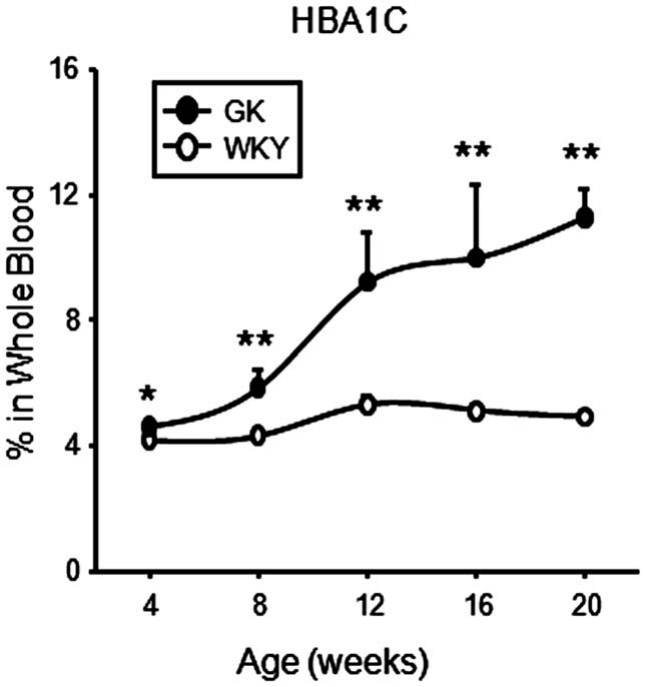
HbA1c in whole blood. Percentages of glycosylated hemoglobin (HbA1c) as a function of age in blood of GK and WKY rats. Data represent means and error bars 1 SD of the mean. Closed circles = GK; open circles = WKY). * = P<0.05; ** = P<0.001.

Plasma glucose measurements in these animals have been previously published [16], but are available here in Figure S1. As early as 4 weeks of age, plasma glucose was significantly higher in GK animals and continued to increase until it reached a plateau at 12 weeks between 500 and 600 mg/dl. To augment those previously reported measurements, we additionally measured glycosylated hemoglobin. Consistent with the hyperglycemic profile measured at the time of sacrifice in the GK group was a continuous increase in HbA1c throughout the experimental period, from a low baseline reading of 4% or less (LLD = 4%) to over 11% at 20 weeks (Figure 4).

### Hormones

**Figure 5:**
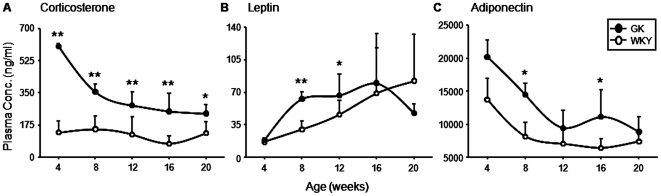
Hormone profiles. Plasma concentrations of A) corticosterone, B) leptin and C) adiponectin as a function of age in GK and WKY animals. Data represent means and error bars 1 SD of the mean. Closed circles = GK; open circles = WKY). * = P<0.05; ** = P<0.001.

Plasma concentrations of four hormones relevant to glucose homeostasis (insulin, corticosterone, leptin, and adiponectin) were measured in both populations of animals. As described previously [16] plasma insulin concentrations were similar in both populations of animals at 4 weeks of age (Figure S1). In WKYs, insulin increased modestly between 4 and 8 weeks of age and remained at a relatively constant level throughout the rest of the experimental period. In contrast, insulin increased dramatically (about 6-fold) in the GK population between 4 and 8 weeks, remained at this elevated level through 12 weeks, then began to decline such that by the end of the experimental period plasma insulin was marginally higher in WKY than in GK. Figure 5 presents plasma corticosterone, leptin, and adiponectin concentrations in these animals. Corticosterone, the natural glucocorticoid in the rat, is released from the adrenal gland in a distinct circadian pattern [20]. These animals were sacrificed close to the nadir of the corticosterone rhythm. The plasma corticosterone concentration in the WKY population was rather constant, with a mean value of 122.4±29.8 ng/ml, with no significant differences across age. In contrast, the corticosterone concentration in GK animals started at about 600 ng/ml at 4 weeks and declined asymptotically, yet was still significantly higher than the WKY animals even at 20 weeks. This result indicates that either the GK population has an unusually high plasma corticosterone concentration or that there is a disturbance in the endogenous circadian rhythm of these animals. Leptin is an adipokine produced by adipose tissue and is also released in a circadian fashion with peak plasma concentration during the late dark/active period and the nadir during the late light/inactive period [21]. Therefore, our time of sacrifice should yield intermediate leptin concentrations. In normal animals, leptin concentrations increase with increasing fat mass [22]–[24]. The plasma leptin concentration in our WKY population continually increased throughout the experiment and essentially mirrored the increase in adipose tissue in these animals. Given the lack of development of adipose tissue in the GK population, plasma leptin concentrations in GK animals were therefore unusually high. For example at 8 weeks both the WKY and GK populations had the same amount of adipose tissue yet the plasma leptin concentration was twice as high in the GK animals. Similarly, at 12 weeks the WKY population had about twice as much adipose tissue than the GK but plasma leptin concentration was still significantly higher in the GK animals. Only at 20 weeks, when the GK population appeared to be losing adipose tissue did the plasma leptin concentration begin to decline. Adiponectin is another adipokine, but in contrast to leptin, plasma concentrations have been reported to be inversely related to the amount of body fat [25]. Plasma concentrations of this hormone were higher in GK animals at all ages, and declined with age in both groups.

### Lipid profiles

As with many hormones, lipids exhibit circadian rhythms and sacrifice time is relevant [21]. The concentration of triglycerides in plasma reaches its peak early in the light/inactive period and its nadir early in the dark/active period. In contrast, plasma LDL cholesterol has exactly the opposite circadian rhythmicity as that of triglycerides with concentrations reaching peak in the early dark/active period and the nadir early in the light/inactive period. In the case of plasma free fatty acid, the concentration is higher during the light/inactive period with peak occurring at the mid-light period and the nadir in the mid-dark/active period. HDL cholesterol in the plasma has no apparent circadian rhythmicity. Lipid profiles as a function of age in both populations are summarized in Table 1. The only significant differences observed were higher triglycerides at 16 and 20 weeks in the GK population.

**Table 1:** Plasma Lipids.

Triglycerides				
	WKY		GK	
Age Weeks	Mean	SD	Mean	SD
4	63.9	25.1	80.6	3.6
8	92.1	42.9	92.9	23.6
12	134.9	32	162.9	55.5
16	138.1	12.1	*175.5	38.6
20	117.3	51.7	*190.7	36.3

Free Fatty Acids				
	WKY		GK	
Age Weeks	Mean	SD	Mean	SD
4	0.06	0.01	0.1	0.067
8	0.06	0.02	0.07	0.03
12	0.07	0.02	0.072	0.01
16	0.07	0.03	0.07	0.03
20	0.1	0.03	0.1	0.02

Total Cholesterol				
	WKY		GK	
Age Weeks	Mean	SD	Mean	SD
4	130.1	17.05	120.77	1.8
8	124.04	19.29	132.6	23.7
12	120	11.69	119.42	27.52
16	124.95	20.87	106.95	1.77
20	120.69	19.98	110.05	6.51

HDL Cholesterol				
	WKY		GK	
Age Weeks	Mean	SD	Mean	SD
4	29.77	10.87	38.73	1.2
8	38.27	15.85	51.16	6.42
12	39.69	6.74	37.95	17.11
16	37.24	12.99	42.44	3.8
20	39.89	8.19	46	4.08

LDL Cholesterol				
	WKY		GK	
Age Weeks	Mean	SD	Mean	SD
4	55.27	16.7	53.91	7.3
8	36.04	7.12	51.31	15.84
12	58.27	13.4	43.52	8.83
16	57.81	12.42	44.31	8.3
20	49.65	9.52	40.86	8.6

### Blood cell profiles

**Figure 6:**
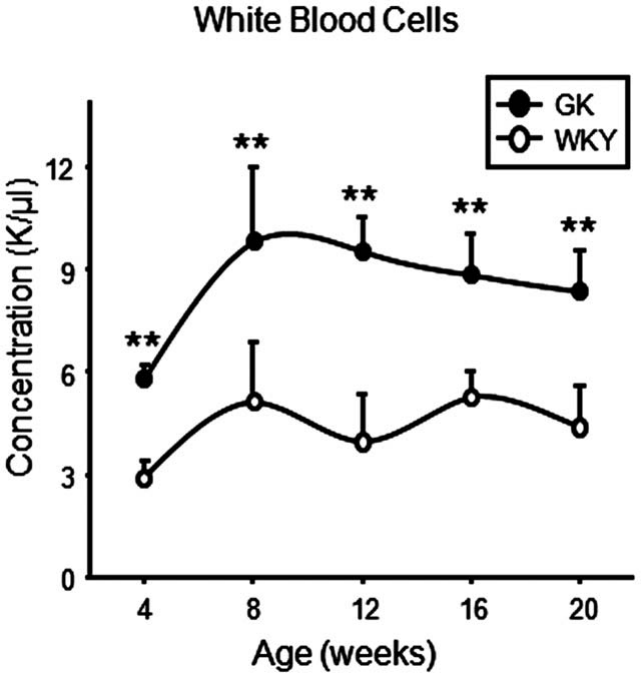
Leukocyte counts. White blood cell counts as a function of age in GK and WKY animals. Data represent means and error bars 1 SD of the mean. Closed circles = GK; open circles = WKY). ** = P<0.001.

Although there were no differences in the number of erythrocytes or platelets, total white cell counts were very significantly elevated in GK animals throughout the entire 20 week experimental period (Figure 6).

### Gene array analysis

The filtering process yielded 611 probe sets exhibiting differential expression in adipose tissue from GK and WKY animal at 3 or more ages. When the same filtering process was applied to the liver gene array data set, 395 probe sets were mined as differentially expressed in the 2 strains [16]. There were 143 probe sets that were differentially regulated in both adipose tissue and liver. Table S1 provides the raw data from individual chips for each of the 611 probe sets in adipose tissue. Raw data for the 395 probe sets identified in liver was provided in our previous report. Probes sets which show differential regulation in both tissues are highlighted in Table S2.

Affymetrix provides an accession number for the sequence from which each probe set was built. We submitted the accession number of each of the 611 selected probe sets to the NCBI Basic Local Alignment Search Tool (BLAST) to identify, as closely as possible, its gene. Of the 611 probe sets, there were 167 probe sets that could not be identified by BLAST. In addition, in 32 instances there were multiple probe sets for the same gene. This left 412 identifiable individual genes that exhibited differential expression in adipose tissue of GK and WKY animals. These genes were then submitted to NCBI “across database search” primarily to identify all aliases and alternate symbols. The preferred symbol was submitted to NCBI AceView as well as extensive PubMed Boolian logic searches to ascertain the function of the gene in adipose tissue. Based on these searches we separated the genes into groups based on their function. These groups were as follows: Transcription/Translation, Signaling, Protein Processing, Energy Metabolism, Immune/Inflammatory, Small Molecule Metabolism, Cell Cycle Control, and Transport. An additional 67 genes which did not readily fit into these categories were grouped as Miscellaneous, and the 167 probe sets not identifiable by BLAST were listed as ESTs. Clearly such functional categorizations are not perfect because overlaps in categories exist. For example, fatty acid binding protein 1 (*Fabp1*) which is involved in fatty acid transport was placed in Energy Metabolism rather than Transport, and cytokines were categorized in the Immune/Inflammatory group rather than in the Signaling group. Table S2 provides a list of all differentially regulated genes organized by these functional categories, and includes their probe set ID, accession number, gene name, symbol, and which strain was higher at how many ages. The functional analysis indicated that the genes that are differentially expressed in the GK and WKY strains are heavily weighted towards four functions. These functional groups are Immune/Inflammatory (103 probe sets), Signal Transduction (64 probe sets), Transcription/Translation (68 probe sets), and Energy Metabolism (44 probe sets). Although normalization of the data by strain provided a convenient way of identifying probe sets that are expressed differently, it does not provide a direct view of actual differences in expression. To accomplish this we reconstructed the data set for the selected probe sets in a form that would allow a direct comparison of the actual expression intensities at each time point for both strains.

#### Immune/Inflammatory processes

**Figure 7:**
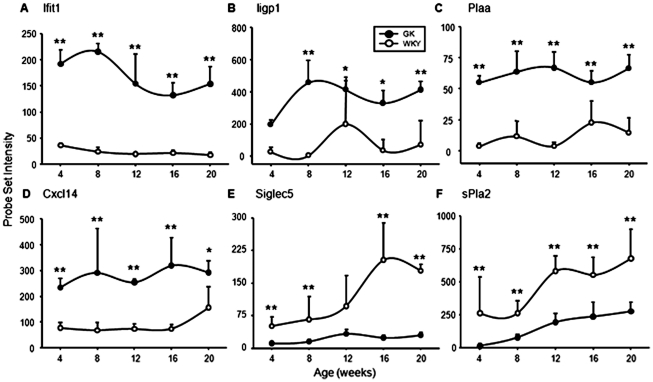
Examples of genes related to immune function. Expression of probe sets related to immune function/inflammation in adipose tissue as a function of age in GK and WKY rats. A Interferon-induced protein with tetra-tricopepetide repeats (Ifit 1: 1369836_at); B Interferon-inducible GTPase (Iigp 1: 1377950_at); C Phospholipade A2 activating protein (Plaa: 1396714_at); D Chemokine C-X-C motif ligand 14 (Cxcl14: 1388485_at); E Sialic acid binding Ig-like lectin 5 (Siglec5: 1385465_at); F Secretory phospholipase A group IIA (sPla2: 168128_at). The Y-axis represents raw, non-normalized probe set intensities, and the x-axis animal age. Data represent means and error bars 1 SD of the mean. Closed circles = GK; open circles = WKY). * = P<0.05; ** = P<0.001.

This functional category of differentially regulated genes (103 probe sets) demonstrates significant differences between the GK and WKY populations with respect to the number of immune cells and inflammation in adipose tissue. Of the 103 probe sets, 73 were higher in WKY as compared to GK animals, while 30 were higher in GK animals. Selected examples of genes in this category are presented in Figure 7. Figures 7A and B show the expression patterns of interferon-induced protein with tetratricopeptide repeats 1 (*Ifit1*) and interferon-inducible GTPase (*Iigp1*). Both of these interferon-induced genes are much higher in the adipose tissue of diabetic GK animals than in control WKY rats at all ages. The expression of both of these genes was also much higher at all ages in the livers of these animals. The high expression of these interferon-induced genes suggests that the previously reported chronic natural immune activation in the liver of GK population extends as well to adipose tissue. In addition to suggesting a heightened inflammatory state in these animals, excessive circulating concentrations of interferon are also associated with insulin resistance [26], [27]. Similarly, phospholipase A2, activating protein (*Plaa*) expression is higher at all ages in the adipose tissue of the GK population (Figure 7C). Pro-inflammatory eicosanoids are released from membrane phospholipids by the action of phospholipase A2 (PLA2), which is activated by PLAA [28]. The expression of *Plaa* was higher at all ages in the livers of these animals as well. A fourth example of a gene suggesting chronic inflammation in the GK rats is chemokine C-X-C motif ligand 14 (*Cxcl14*). In the presence of prostaglandins, CXCL14 can both attract and activate a variety of immune cells. It was previously demonstrated that disruption of *Cxcl14* greatly reduced obesity-induced insulin resistance in mice, and that restoring the gene restored the insulin resistance [29]. Figure 7D shows that *Cxcl14* is chronically higher in GK when compared with WKY. Like the previous three examples, *Cxcl14* is also elevated in the livers of these animals. In contrast to the expression of genes suggesting inflammation, aspects of the data suggest that there are actually more immune cells in the larger adipose tissue mass of the WKY population. For example, the expression of sialic acid binding Ig-like lectin 5 (*Siglec5*) which is primarily expressed by neutrophils [30] is higher as early as 4 weeks in the WKY population and increases with age, while the expression in adipose tissue of the GK diabetic animals remains rather low throughout the 20 week experimental time period (Figure 7E). In addition, the expression of secretory phospholipase A2, group IIA (*sPla2*) which in adipose tissue is likely associated with macrophages [31], increases with age in both groups but is significantly higher at all ages in WKY (Figure 7F). Similarly the expression of other genes such as Tnf receptor-associated factor 6 (*Traf6*), caspase recruitment domain family, member 9 (*Card9)* and integrin alpha M (*Itgam*), a macrophage marker, are also more highly expressed in the adipose tissue of WKY relative to GK (Table S2). The sum of these data suggest that while markers of immune cells in general are higher in the WKY population, which is consistent with their greater amount of adipose tissue, markers suggesting inflammation are higher in the GK population.

**Figure 8:**
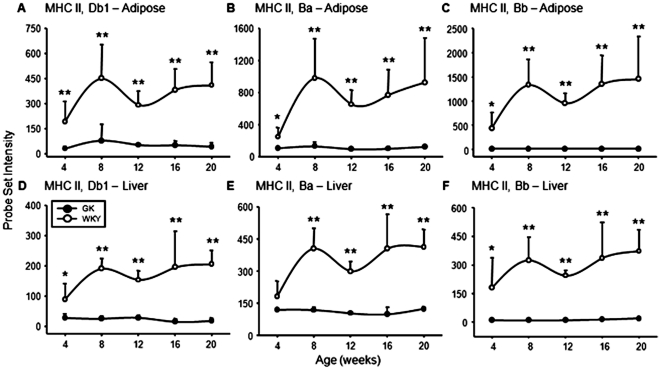
MHC class II gene expression in adipose tissue and liver. Expression of MHC Class II probe sets in adipose tissue and liver from GK and WKY rats as a function of age. MHC II, Db1 (1370382_at) in adipose tissue (A) and liver (D); MHC II, Ba (1370822_at) in adipose tissue (B) and liver (E); MHC II, Bb (1371033_at) in adipose tissue (C) and liver (F). The Y-axis represents raw, non-normalized probe set intensities, and the x-axis animal age. Data represent means and error bars 1 SD of the mean. Closed circles = GK; open circles = WKY). * = P<0.05; ** = P<0.001.

Many MHC genes were found to be differentially regulated between the GK and WKY populations (Table S2). Most were class I MHCs, which showed no strain specific pattern of expression. However, there were only three MHC class II genes that were differentially regulated: locus Db1 (1370382_at), locus Bb (1371033_at), and locus Ba (1370822_at). As shown in Figure 8, the expression of all three is very low to absent in both adipose tissue and liver from the GK population while being reasonably high in both tissues from the WKY population. What makes these results rather interesting is not only that differential expression involves both tissues, but also that polymorphisms in these loci are associated with susceptibility to autoimmunity in the rat and with type 1 diabetes in humans [32], [33]. The chip did contain a probe set for MHC Class II Trans-Activator (*CIITA*), a transcriptional coactivator controlling the expression of MHC class II genes [34], but there was no difference in expression between WKY and GK animals.

#### Energy metabolism

**Figure 9:**
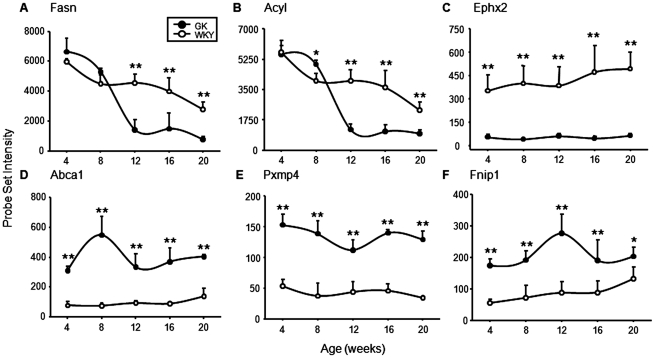
Examples of genes related to energy metabolism. Expression of probe sets related to metabolism in adipose tissue from GK and WKY rats as a function age. A) Fatty acid synthase (Fasn: 1367707); B) ATP citrate lyase (Acyl: 1367854); C) Epoxide hydrolase 2 (Ephx2: 1369663_at); D) ATP binding cassette subfamily A member 1: 1382431_at); E) Peroxisomal memebrane protein 4 (Pxmp4: 1393421_at); F) Folliculin interacting protein 1 (Fnip1: 1383894_at). The Y-axis represents raw, non-normalized probe set intensities, and the x-axis animal age. Data represent means and error bars 1 SD of the mean. Closed circles = GK; open circles = WKY). * = P<0.05; ** = P<0.001.

The lack of development of adipose tissue shown in Figure 3 suggests that the GK population may have an impaired ability to function in the cycle of lipogenesis and lipolysis that supports systemic energy metabolism. The expression of 44 probe sets representing 36 genes provide some insight into the difference in the adipose tissue mass in the two populations. Twenty genes in this functional category showed lower expression in GK adipose tissue. These included fatty acid synthase (*Fasn* – Figure 9A) and ATP citrate lysase (*Acly –* Figure 9B), both of which are important to lipogenesis. These two genes showed significantly lower expression in the GK population from 12 weeks on, paralleling the time where fat accumulation halts in these animals. Other lipogenesis-related genes with a similar temporal expression pattern include ELOVL family member 6, elongation of long chain fatty acids (*Elovl6*), transketolase (*Tkt*), butyryl Coenzyme A synthetase 1 (*Bucs1*), malic enzyme 1 (*Me1*), uncoupling protein 1 (*Ucp1*) and others (Table S2). Epoxide hydrolase 2 (EPHX2) is an important enzyme involved in the metabolic breakdown of arachidonic acid-derived eicosanoids [35]. The higher expression of *Plaa* noted previously, Figure 7F, makes the chronically lower expression of *Ephx2* in the GK population (Figure 9C) quite relevant. A similar pattern of *Ephx2* expression is seen in the liver of these animals. In contrast, 16 genes had higher expression levels in the adipose tissue of the GK population. These included ATP-binding cassette, sub-family A, member 1 (*Abca1*), Figure 9D. ABCA1 is a cholesterol efflux pump in the cellular lipid removal pathway. *Abca1* showed a very similar pattern of expression in the liver. The gene chips contained two probe sets for peroxisomal membrane protein 4 (*Pxmp4*), which may play a role in fatty acid degradation. Both probe sets showed very similar patterns of expression being more highly expressed in both the adipose tissue and the liver of the GK population (Figure 9E). Folliculin interacting protein 1 (*Fnip 1*), which we functionally classified in the signaling category, is downstream in the signaling cascade initiated by activated AMP-activated protein kinase (AMPK) [36]. AMPK serves as an energy status sensor in many types of cells [37]; in adipose tissue, activated AMPK reduces fatty acid efflux and favors local fatty acid oxidation. The expression of *Fnip1* is higher at all ages in GK relative to WKY (Figure 9F).

#### Other genes of particular interest

**Figure 10:**
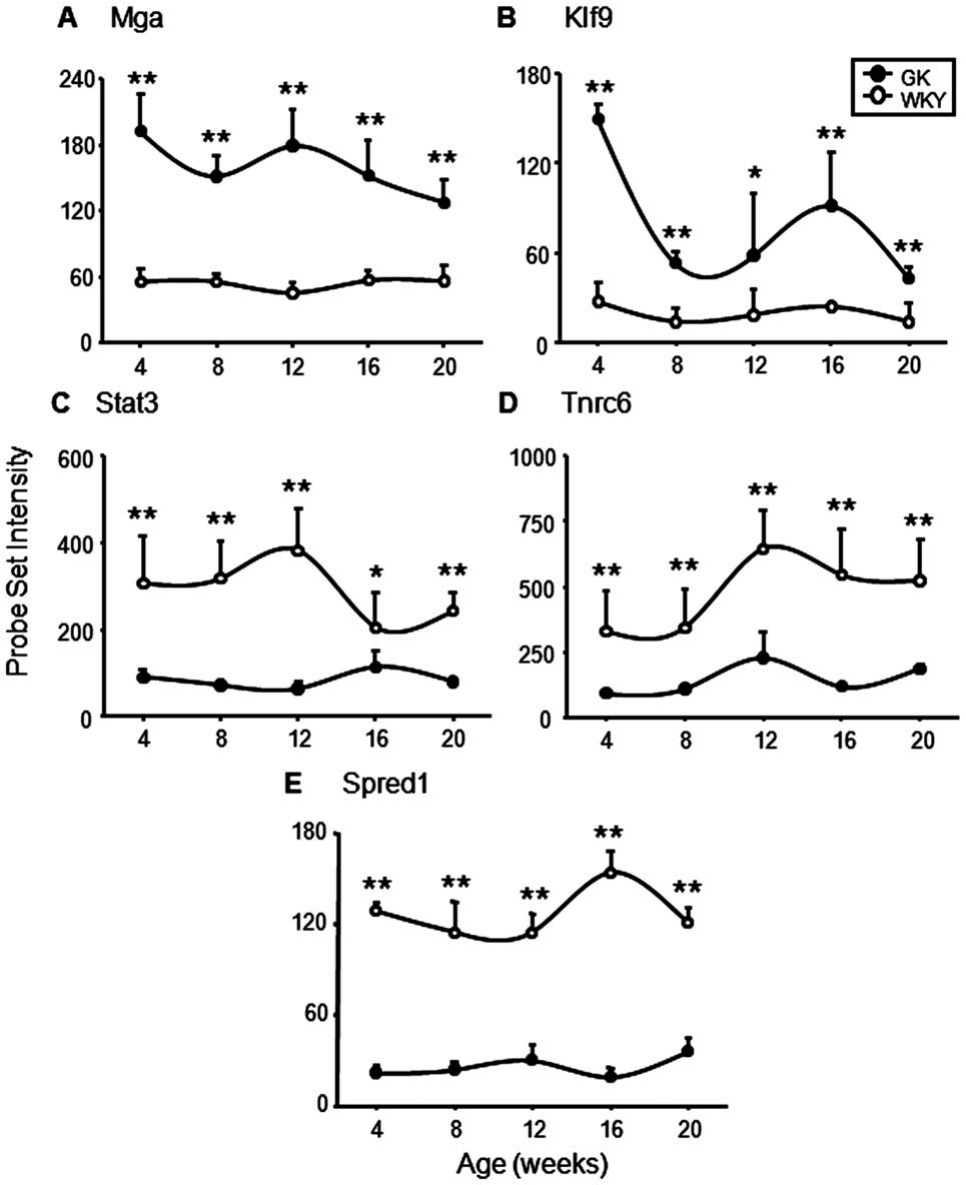
Examples of additional genes related to adipose mass and inflammation. Expression of additional genes of interest in adipose tissue from GK and WKY rats as a function age. A) MAX gene associated (Mga: 1391600_at); B) Kruppel-like factor 9 (Klf9: 1395030_at); C) Signal transducer and activator of transcription 3 (Stat3: 1370224_at); D) Trinucleotide repeat containing 6 (Trnc6: 1375664_at); E) Sprouty protein EVH1 (Spred1: 1392788_at). The Y-axis represents raw, non-normalized probe set intensities, and the x-axis animal age. Data represent means and error bars 1 SD of the mean. Closed circles = GK; open circles = WKY). * = P<0.05; ** = P<0.001.

There are many additional genes that also warrant attention because of their differential expression in GK and WKY rats. One such gene is MAX gene associated (*Mga*) whose expression is higher at all ages in the GK group (Figure 10A). MGA functions as a dual-specificity transcription factor, regulating the expression of both MAX-network and T-box family target genes and is implicated in the control of cell proliferation and differentiation [38]. Another gene whose expression is higher at all ages in the GK group is the transcription factor kruppel-like factor 9 (*Klf9*) (Figure 10B). *Klf9*, whose expression is influenced by thyroid hormone, is also involved in adipocyte differentiation [39].

In contrast, the expression of leptin-activated transcription factor, signal transducer and activator of transcription 3 (*Stat3*) is substantially higher in adipose tissue at all ages in the WKY population (Figure 10C). STAT3 is a transcription factor that is involved is a variety of cellular functions. Employing knockout experiments, it has been observed that STAT3 plays a critical role in controlling inflammation and that deletion of *Stat3* causes an exacerbated inflammatory response [40]. In addition, activation of STAT3 stimulates adipogenesis [41]. Like other differentially regulated genes, *Stat3* shows a similar pattern in the livers from these animals. It has been demonstrated that microRNA (miRNA) mediated gene silencing plays a significant role in adipocyte differentiation [42]. Trinucleotide repeat containing 6 (TNRC6) is a component of the miRNP silencing complexes [43]. Therefore, it may be significant that *Tnrc6* is substantially higher in the WKY population at all ages (Figure 10D). The potential significance of this observation is reinforced by our previous observation that TNRC6 shows almost the exact same pattern in the livers of these animals. Sprouty protein with EVH-1 domain 1 (SPRED-1) is a negative regulator of inflammation [44]. *Spred1* is also more highly expressed in both adipose tissue (Figure 10E) and liver of WKY animals.

## Discussion

Most published studies on GK rats involve animals of a single age. By using a time series from 4 weeks to 20 weeks, we observe the progression of differences between WKY control and GK animals as diabetes develops. With respect to body weight, a difference is apparent by 8 weeks of age. While not different at 4 weeks, body weights began to diverge at 8 weeks, with WKYs significantly heavier than GKs from 12 weeks onwards. This difference occurs despite the fact that GKs consume more calories per gram body weight than WKYs. Our results show that adipose tissue is a major contributor to differential weight gain in GK rats, as visceral fat accumulation halts by 12 weeks of age, and even declines somewhat at later ages (Figure 3). It is also significant that epididymal fat showed a similar pattern (Figure 3) suggesting a generalized defect in adipose development. In contrast, our previous work demonstrated that as the animals grow, the liver is larger in the GKs relative to total body weight [16]. Although the GK rat is often described as “lean” (i.e., non-obese), our results clearly demonstrate that the GK strain of rats have an age-specific failure to accumulate body fat.

Previously we conducted a time series gene array analysis of the livers from these same animals [16]. A functional analysis of the gene expression in the livers from these animals led to two major conclusions. The first was that in the liver, there is chronic natural immune activation. This chronic activation of natural immunity is apparent in both the LPS cascade response to gram negative bacteria and the interferon response cascade to viruses. The second was that in the GK population there was a disruption in lipid metabolism. In the present report we describe a similar functional gene expression analysis of WAT from the animals. The array data for genes involved in the use of energy substrates show that there is divergence between the two populations of animals that starts at 12 weeks of age, the same time when adipose tissue development diverges. For example, after 12 weeks, the expression of fatty acid synthase (*Fasn*) and ATP citrate lyase (*Acl*) are markedly higher in the WKY population (Figures 8A and B). Additional relevant genes with almost the exact same temporal pattern of expression include ELOVL family member 6, elongation of long chain fatty acids (*Elovl6*), transketolase (*Tkt*), butyryl Coenzyme A synthetase 1 *(Bucs1*), malic enzyme 1 (*Me1*), and uncoupling protein 1 (*Ucp1*) (Table S2). Taken together, the results suggest that adipose tissue in the adult GK population is incapable of carrying out the mature adipocyte function of storing triglycerides and releasing free fatty acids.

The likelihood of impaired adipocyte differentiation in the GK population is reinforced by the expression of several genes involved in transcription and translation. For example, the expression of both MAX gene associated (*Mga*) and kruppel-like factor 9 (*Klf9*) is higher at all ages in the adipose tissue of the GK population. Max is a heterodimeric partner of MYC, which has been shown to inhibit preadipocyte to adipocyte differentiation [38], [45]. Similarly, KLF9 transiently increases during preadipocyte differentiation but decreases with triglyceride accumulation [39]. Consistent with the conclusion that in the adipose tissue of the GK population the preadipocyte to mature adipocyte transition is impaired is the observation that the expression of *Stat3* is considerably higher at all ages in the adipose tissue of the WKY population. *Stat3* is activated by leptin and stimulates adipogenesis [41]. Perhaps one of the more intriguing observations relevant to transcription and translation differences between the two populations is the consistently higher expression of trinucleotide repeat containing 6 (*Tnrc6*) in WKY animals. This same difference was also observed in the livers from these animals. TNRC6 is a component of the miRNA-containing ribonucleoprotein silencing complexes [42]. Recently it has been demonstrated that miRNA mediated gene silencing plays a significant role in adipocyte differentiation [43].

Our conclusion from both the fat development data and the gene expression analysis is that in the GK population there is impairment in preadipocyte to mature adipocyte differentiation. Consistent with this conclusion is the documented decreased numbers of large mature lipid engorged adipocytes in 3–4 month old GK animals reported previously [46]. However, this study only examined the adipocyte population in adult animals, and currently a similar analysis in younger animals, before the growth transition at 12 weeks, would be of value. Likewise, no information is currently available on adipose tissue stem cell populations in these animals. However, it has previously been shown that GK rats have a reduced regenerative ability after partial pacreatectomy, suggesting a reduced stem cell population in that tissue [47].

However, the data do provide an indication of the cause of the impaired adipocyte differentiation. Although excess adipose tissue (obesity) is thought to be a cause of chronic inflammation because of the attraction of macrophages into the environment of lipid engorged adipocytes, the presence of chronic inflammation actually retards adipocyte differentiation [48]. In addition to our previously reported data showing early chronic inflammation in the liver, in the present report we provide data demonstrating the white cell count is elevated in the GK population as early as 4 weeks and remains elevated throughout the experiment (Figure 6). The gene expression data on WAT similarly indicates early inflammation. About seventy percent of genes that might be considered markers for immune cells were more highly expressed at later ages in the adipose tissue of the WKY population. The higher percentage of immune cell markers at later ages in WKY is consistent with the greater mass of adipose tissue in these control animals. However, a detailed analysis focusing on those genes that reflect inflammation suggests chronic inflammation in the adipose tissue of the GK population. For example, Figure 7D shows that the expression of *Cxcl14* is much more highly expressed in adipose tissue of the GK population as early as 4 weeks as compared to the WKY population. CXCl14 is a secreted protein involved in immunoregulatory and inflammatory processes [29] and is involved in attracting macrophages into WAT. This gene was also more highly expressed in the livers of these animals at the same age. Two other genes with a relationship to inflammation with early higher expression in the GK population are interferon-induced protein with tetratricopeptide repeats 1 (*Ifit1*) and interferon-inducible GTPase (*Iigp1*). Interferons are involved in natural immunity. Additional genes associated with inflammation such as the macrophage secreted galectins, *Lgals5* and *Lgals9* similarly suggest heightened inflammation in the GK population (Table S2). A plausible hypothesis to be formulated from the data as a whole is that early chronic inflammation impairs adipocyte maturation in the GK population. This hypothesis is supported by the earlier observation that in the rat, the number of mature adipocytes expands for about the first 12 weeks of life [12] together with our current data demonstrating that 12 weeks is the age when the GKs cease to accumulate WAT.

One potential clue to the immunological differences between the WKY and GK strains is the considerably different expression in both liver and adipose tissue of MHC class II genes Db1, Bb, Ba. The coding sequences of these genes are located very close to each other on rat chromosome 20 [32]. Polymorphisms amongst rat strains in these genes are associated with a susceptibility to developing a variety of autoimmune diseases in the rat which include type 1 diabetes, thyroditis, arthritis and autoimmune encephalomyelitis [33].

Previously we provided data that the GK population was transiently hyperinsulinemic and that plasma glucose was elevated at 4 weeks and climbed to a high hyperglycemic plateau by 12 weeks. We chose to maintain our rats in a normal fed state in order to avoid possible variations in physiological measurements induced by fasting. Studies in the literature, particularly on glucose in diabetic rats, differ in whether measurements are taken in the fed state or following varying lengths of fasting (6 hours to 18 hours, or more often a less defined “overnight fast”). Since rats are nocturnal, and approximately 90% of their food consumption occurs in the dark (i.e. overnight) period [49], [50], an overnight fast is actually more equivalent to 24 hours or more, and such animals may be in early phases of starvation [51]. For this reason, we maintained our animals in a fed state and collected samples between 1.5 and 3.5 h after lights on. Because blood glucose has a circadian rhythm [21], a measurement at any particular time under a particular condition is only relative. In contrast, HbA1c provides a very good indication of glycemic control over time. Figure 4 shows a continuous increase in glycosylated hemoglobin throughout the 20 week experimental period, demonstrating a chronic disruption of glucose homeostasis in the GK population.

In addition to insulin, glucocorticoids play a major role in regulating glucose homeostasis. Corticosterone which is the primary glucocorticoid in the rat varies with a circadian rhythm driven by light input to suprachiasmic nucleus of the hypothalamus. Plasma corticosterone peaks in the rat shortly after the beginning of the dark period and reaches a low point shortly after the beginning of the light period [20]. We measured plasma corticosterone at a time when the plasma concentration should be near its lowest point. In the control WKY population, the plasma corticosterone was relatively stable ranging between 100 and 150 ng/ml. over the 20 week experimental period. Given the constant time of sampling, the relative constancy is expected. In contrast, plasma corticosterone in the GK population was extremely high at 4 weeks (about 600 ng/ml) and declined asymptotically to a low point at 20 weeks of around 200 ng/ml. To put this into perspective, in our previous work on circadian rhythms using Wistar rats obtained from Harlan, the peak plasma concentration of corticosterone measured shortly after the beginning of the dark period was about 300 ng/ml. The results indicate that either the GK population has extremely elevated concentrations of plasma corticosterone or that their circadian rhythms are disturbed. Glucocorticoids enhance gluconeogenesis in the liver and inhibit glycerolgenesis in adipose tissue [52]. The rate limiting enzyme for both processes is phosphoenol pyruvate carboxykinase (PEPCK). In some individuals with T2DM hyperglycemia is driven by excessive gluconeogenesis in the liver. In our previous report on the livers from these animals we found elevated expression of *Pepck* in the liver only at 4 weeks. In the present study on adipose tissue we found no significant difference between GK and WKY in the expression of *Pepck* (data not shown). Given the very large differences in plasma corticosterone between the two populations, the essential lack of difference in expression of *Pepck* in both tissues is abnormal. It is relevant that disturbance of circadian rhythms can cause diabetes in a mammal [53] and that glucocorticoids cause insulin resistance [54], [55]. The possibility of a disrupted circadian rhythm in GK rats is reinforced by the recent report of Bach et al. documenting a disturbance in the pineal melatonin circadian rhythm in these animals [56].

Adipose tissue is a source of a variety of hormone-like factors (adipokines) that play a role in systemic energy flow, including leptin and adiponectin. Leptin is an adipokine associated with decreased appetite and increased metabolic rate, and plasma concentrations have been shown to increase with increasing fat mass [22]–[24]. In our study, plasma leptin concentrations in WKY animals increased through 20 weeks, as did adipose mass. In contrast, plasma leptin concentrations in the GK population increased only through 8 weeks, and even decline at 20 weeks. However, despite the fact that leptin output mirrors fat accumulation leptin levels, particularly at early ages, are higher in GK animals. Since GK animals consumed more food per gram body weight than WKYs, this suggests leptin resistance, at least in its effect on appetite suppression. Maekawa and colleagues have also previously reported increased food intake in GK rats, which they demonstrated was associated with central leptin resistance [57]. However, because the plasma concentration of leptin, like corticosterone, has a circadian rhythm, interpretation of our results must be qualified. It is relevant that plasma leptin peaks in late dark near the beginning of the light period and reaches a low point in the late light near the beginning of the dark period. Thus, this result may also reflect some disruption of circadian rhythms in the GK population.

In contrast to leptin, plasma concentrations of the insulin sensitizing hormone adiponectin are inversely related to body fat mass [25]. In both populations of animals, plasma adiponectin decreases asymptotically with age. What is unusual about these results is that at the younger ages when fat mass in both populations is similar, adiponectin concentrations are modestly higher in the GK population and this trend continues at the older ages when the WKY population has substantially greater body fat. Although adiponectin is normally insulin sensitizing, the GK population is hyperglycemic even at 4 weeks of age.

Gene arrays simply provide a snapshot of the concentration of mRNAs at one point in time. The results reflect the potential available at that time for the production of proteins. When used in a time series as we have done in this experiment, we are able to observe how that potential changes with disease development and progression. In addition, a time series design such as this greatly reduces the number of false positives that might be obtained in array studies if only two groups were compared. In fact, it is possible that our rather stringent filtering criteria may eliminate some genes of potential interest. In addition, because of the heterogeneity of the cells in WAT, we cannot directly determine if a particular message is from a particular cell type. Although we cannot directly ascertain whether steady-state concentrations of message are due to mRNA production or destruction, we can obtain insight from examining messages for proteins involved in functional processes that can either contribute to hyperglycemia or may reflect the impact of hyperglycemia.

Each animal in this study had a unique identifier and all samples are indexed to this identifier. Table S1, which provides the raw data for each individual probe set identified as differentially regulated in adipose tissue from these animals, also provides an animal code for each individual chip. Chip identification in conjunction with the GEO make all our data completely available to others. It is our intent to provide this code information for all reported measurements in all publications from our ongoing studies, whether they be gene arrays, biochemical or physiological. From a systems biology perspective this will make these studies most useful to other investigators.

## Supporting Information

Figure S1 **Glucose and insulin.** Plasma glucose (A) measured by the glucose oxidase method (Sigma, St. Louis, MO) and plasma insulin (B) measured by RIA (RI-13K Rat Insulin RIA Kit, Millipore Corporation, St. Charles, MO) in GK (closed circles) and WKY (open circles) rats as a function of age as previously described (Almon et al, J. Endocrin. 200: 331, 2009). Symbols represent mean values and error bars 1 SD of mean.
(TIF)

Table S1 **Raw Probe Set Intensities of Differentially Mined Genes**
(PDF)

Table S2 **Differentially Expressed Probe Sets by Function.**
(DOC)
