# Supplementary figures and images for: Adipose Tissue Deficiency and Chronic Inflammation in Diabetic Goto-Kakizaki Rats
Source: PLoS One. 2011 Feb 25;6(2):e17386. doi: 10.1371/journal.pone.0017386 (PMC3045458; doi:10.1371/journal.pone.0017386)

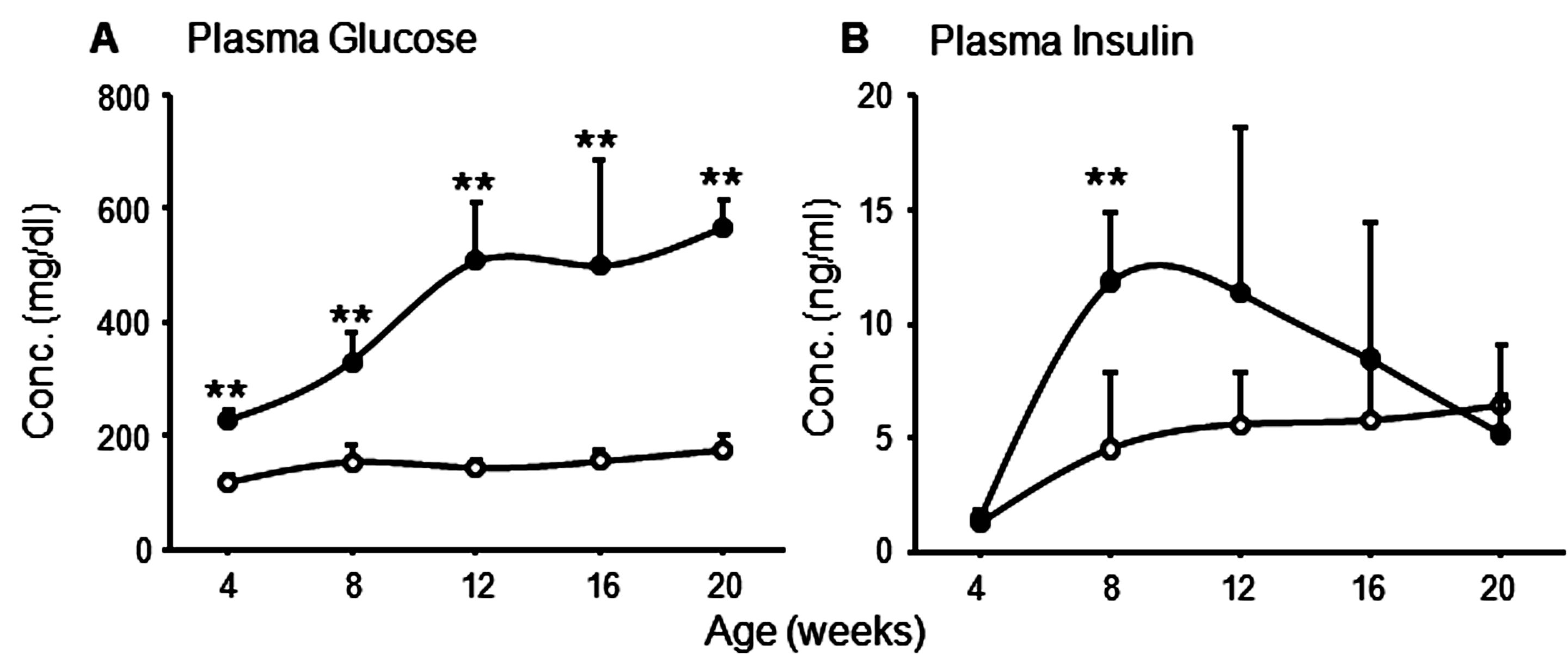

Supplement: Figure S1 — Glucose and insulin. Plasma glucose (A) measured by the glucose oxidase method (Sigma, St. Louis, MO) and plasma insulin (B) measured by RIA (RI-13K Rat Insulin RIA Kit, Millipore Corporation, St. Charles, MO) in GK (closed circles) and WKY (open circles) rats as a function of age as previously described (Almon et al, J. Endocrin. 200: 331, 2009). Symbols represent mean values and error bars 1 SD of mean. (TIF) [file pone.0017386.s001.tif]
