# Supplementary material for: Adipose Tissue Deficiency and Chronic Inflammation in Diabetic Goto-Kakizaki Rats
Source: PLoS One. 2011 Feb 25;6(2):e17386. doi: 10.1371/journal.pone.0017386 (PMC3045458; doi:10.1371/journal.pone.0017386)
Supplement: Table S1 — Raw Probe Set Intensities of Differentially Mined Genes (PDF) [file pone.0017386.s002.pdf]

On-Line Supplementary Table 1

Animal	Strain	Age (Week)	1367555_at	1367556_s_	1367572_at	1367581_a_	1367592_at	1367611_at
DD81	GK	4	7299.519	5344.2397	2254.6401	81.98485	94.27539	135.53944
DD84	GK	4	666.3398	295.02017	2564.8235	117.42667	179.10115	204.69832
DD85	GK	4	1235.545	539.8446	2123.0525	127.41376	145.92188	206.08344
DD86	GK	4	1078.1195	517.3624	159.73814	172.58006	1.89581	293.93985
DD83	GK	4	470.114	227.54967	1998.3406	92.691315	145.06076	200.46729
DD41	GK	8	298.63986	155.68231	609.66	1958.4374	18.179857	151.33661
DD42	GK	8	423.3656	204.026	260.2759	3047.0193	1.7632838	156.2452
DD43	GK	8	158.1205	95.15156	212.8247	325.74484	2.1464794	129.04672
DD44	GK	8	323.9611	147.40677	202.60274	556.4075	5.981686	139.1954
DD87	GK	8	359.52222	194.17282	253.04028	2079.6218	0.8447359	176.39021
DD2	GK	12	135.82562	68.84304	1367.4144	745.34705	2.6012166	22.699965
DD3	GK	12	579.7638	244.76334	260.11237	484.87332	1.2496649	40.73877
DD89	GK	12	334.58487	135.88914	208.31216	725.0098	1.8314785	62.974503
DD90	GK	12	1043.2478	381.25836	35.458767	483.65112	132.2237	33.359436
DD4	GK	12	457.0621	145.32199	133.08197	1020.6912	0.9322073	12.211104
DD47	GK	16	3138.8489	1584.9985	125.84225	1704.0437	0.5776801	85.31033
DD48	GK	16	4368.7305	2543.3884	1497.1777	713.17865	10.907681	13.515891
DD49	GK	16	1663.8318	732.2942	217.57663	218.565	1.8997197	32.199135
DD50	GK	16	1738.5149	793.90015	153.2639	371.4418	4.6948147	34.60935
DD46	GK	16	2547.526	1349.6285	291.42676	353.7819	0.5372487	32.03506
DD6	GK	20	434.42645	205.02405	691.1932	303.70215	11.673618	6.039344
DD7	GK	20	338.56485	168.42819	182.89998	730.1573	1.0854213	44.257664
DD8	GK	20	704.27545	331.33292	16.08352	529.07404	0.6532875	62.688515
DD5	GK	20	593.8701	212.84859	75.884476	530.81665	1.8420991	29.481192
DD9	GK	20	372.07703	162.25412	45.559288	1038.295	0.6738392	34.636055
DD103	WKY	4	1884.0204	966.62665	1244.4506	76.18285	104.64284	400.2489
DD101	WKY	4	1920.4908	867.2429	2583.2266	103.39463	559.1386	260.84647
DD102	WKY	4	4774.7256	2496.7546	1498.5721	116.50797	184.41751	335.43777
DD105	WKY	4	2086.4907	838.4517	2781.842	114.73267	765.77716	276.5266
DD106	WKY	4	4356.3613	2298.0852	2712.398	111.21937	634.6212	238.99278
DD62	WKY	8	576.1133	247.34863	1689.4015	136.11174	186.91875	106.62838
DD64	WKY	8	873.83734	418.814	469.873	249.50348	125.96957	106.10729
DD107	WKY	8	758.5589	359.79355	88.06539	73.51717	84.55697	61.58354
DD108	WKY	8	492.01633	235.61122	308.84473	67.99491	248.98917	185.88951
DD63	WKY	8	508.36563	265.35803	339.18106	63.043167	238.255	210.53049
DD23	WKY	12	1018.3304	442.01352	50.830517	269.19965	27.413431	140.80615
DD24	WKY	12	765.2783	326.05368	183.58382	187.6343	23.269306	177.6831
DD110	WKY	12	592.9707	269.15237	11.714273	133.38382	179.37474	88.9285
DD22	WKY	12	1004.6368	458.15552	253.2577	106.14761	71.51963	140.32372
DD109	WKY	12	943.98047	434.34402	22.336407	226.45374	69.112755	153.73598
DD65	WKY	16	1104.5355	490.14145	35.53811	201.763	181.98515	88.82046
DD66	WKY	16	477.15857	217.92484	79.86238	2255.412	7.9072514	127.40505
DD67	WKY	16	7354.667	5118.034	143.76317	157.35637	2.3517997	374.6653
DD69	WKY	16	1402.973	709.00287	53.699043	205.93442	116.9363	81.889435
DD68	WKY	16	2062.666	1060.1217	48.670937	113.00494	3.6668177	85.650665
DD25	WKY	20	1197.7411	596.6144	16.60009	870.5054	2.1075404	90.73158
DD28	WKY	20	910.798	483.7324	35.388184	363.2765	236.27551	67.24249
DD29	WKY	20	1028.0214	553.57385	38.27729	718.3322	434.25018	172.87224
DD27	WKY	20	974.76855	501.67975	57.595535	259.5694	2.9798918	70.256004
DD30	WKY	20	1289.0775	626.7614	5.409724	903.8498	1.0218579	90.85754

On-Line Supplementary Table 1

Animal	1367627_at	1367647_at	1367668_a_	1367707_at	1367854_at	1367928_at	1367942_at	1368000_at
DD81	755.20636	1465.5245	873.9807	6074.5728	5057.8945	1501.7949	208.942	937.3414
DD84	581.58765	41.783394	1047.7487	6551.3335	5758.2007	1278.7175	178.92075	47.421276
DD85	690.8093	68.21169	1197.6835	7127.0254	5625.3833	1320.631	184.15346	64.83306
DD86	571.48315	38.777695	1487.1392	7890.936	6692.785	32.922108	217.77762	28.427383
DD83	839.2507	30.342588	817.6255	5547.3003	4524.0576	1538.6675	193.09676	29.670198
DD41	997.2573	34.430466	1419.8838	5219.089	5160.859	304.44498	161.44356	22.240541
DD42	1032.5502	55.88331	2328.905	5496.343	5216.5547	92.30088	145.68103	26.3206
DD43	1055.6006	19.708092	929.59265	5530.8667	4808.7007	59.938004	482.84207	132.29773
DD44	1191.484	39.193405	781.6625	4876.4917	4740.9214	78.90898	292.7633	118.45905
DD87	1243.8567	33.829937	1775.8508	5336.559	4884.1577	88.558655	166.6196	36.549633
DD2	301.00485	15.443939	648.0625	698.3723	735.47424	846.5531	227.67122	81.730934
DD3	623.3808	60.60119	164.14903	1803.2782	1285.2194	144.81334	182.41841	38.928093
DD89	822.8901	18.26045	121.96111	2359.834	1667.8164	124.08175	173.44029	40.043797
DD90	629.089	93.4714	419.9182	1196.4629	1180.4031	0.5473805	142.79619	55.62967
DD4	814.491	33.908356	196.88991	985.486	1113.55	54.21808	135.95331	45.810677
DD47	889.57	288.12024	944.13	3359.8044	1804.5847	37.051186	160.25229	175.4699
DD48	483.9571	967.37946	455.23334	1155.838	821.2326	1058.8247	133.82207	353.8858
DD49	406.45386	241.35178	125.39892	993.01105	942.5546	113.7935	162.90842	137.70468
DD50	324.1364	197.18861	197.29729	1027.338	928.3731	47.767467	142.97821	170.43279
DD46	444.3709	349.12112	197.60257	874.66254	944.0982	193.90685	164.85533	212.76088
DD6	337.8661	24.832607	301.8969	442.9211	684.5016	505.79782	213.18947	53.772495
DD7	422.66586	21.011627	195.75923	679.3274	867.871	94.85483	193.96246	33.53895
DD8	510.37115	22.263123	154.8189	942.7075	1172.6488	6.315859	202.1599	29.21631
DD5	494.90012	36.568966	299.9152	880.5202	1095.6139	23.202482	208.13943	33.346535
DD9	607.1178	26.276442	205.1564	845.3099	1035.671	9.052364	216.3733	32.58933
DD103	216.09387	138.24808	1351.4974	6127.0693	6053.3086	469.68185	309.56973	195.52304
DD101	362.94022	98.68214	988.4653	6162.453	5490.175	1326.113	228.4503	98.34163
DD102	230.64519	479.70676	1401.7104	6003.989	6123.239	647.47064	351.29364	490.40607
DD105	377.85757	130.71028	1361.1091	5886.145	5361.319	1291.6631	211.93286	146.71703
DD106	376.226	404.27963	1178.8876	5608.2334	5212.3696	990.6234	253.3277	360.26456
DD62	224.35147	8.920354	388.70203	4819.541	3925.7979	726.0904	225.68364	24.850939
DD64	157.83429	24.980633	504.4121	4116.2183	3782.2441	161.77594	334.6988	57.693516
DD107	107.57042	16.99662	813.5508	3618.534	3440.0627	21.347246	525.1454	248.46492
DD108	185.0892	25.78229	815.25604	4831.76	4400.96	159.10951	462.31058	150.22766
DD63	155.09962	19.91652	779.99817	5037.8384	4486.583	161.92087	457.85245	125.68977
DD23	239.39494	18.725706	769.4245	5039.2827	4175.4575	12.446072	432.5092	51.48826
DD24	577.08417	18.144976	1031.6667	4993.4165	4760.952	77.11538	366.5643	30.410416
DD110	152.41237	9.589853	257.68088	3620.1265	3248.3481	8.661622	404.5314	59.298607
DD22	217.95705	10.481331	450.2672	4879.2344	4424.042	100.5687	423.2491	67.4475
DD109	337.87256	20.122852	444.11646	4208.363	3385.0945	10.436985	473.6167	68.587585
DD65	159.03455	16.067932	260.24167	3726.881	3362.0398	7.1196303	347.1237	38.505466
DD66	445.78174	32.257362	839.5356	4490.6235	4110.188	11.326093	395.22662	60.28636
DD67	217.57408	1910.0051	1457.3912	5340.631	5040.569	36.60256	375.5004	1114.3344
DD69	131.5365	17.497868	393.24796	3167.5664	3066.1707	15.091123	450.99997	87.1718
DD68	217.69441	141.42725	272.922	3202.2566	2521.0166	3.5020797	517.8517	227.36504
DD25	173.82968	27.165564	971.7965	2458.8413	2114.5955	3.6023316	379.38947	90.67195
DD28	241.7636	28.313251	266.25305	2856.9087	2049.1204	9.2410145	571.4479	45.736465
DD29	328.08102	23.372747	939.2444	3572.2427	3177.3662	1.2979159	388.38235	89.94495
DD27	111.4857	17.912699	1088.3385	2466.7734	2138.804	8.859243	589.744	100.27763
DD30	180.65236	36.235798	954.7939	2506.6992	2085.1077	11.681175	397.00867	84.95897

On-Line Supplementary Table 1

Animal	1368048_at	1368064_a_	1368128_at	1368145_at	1368167_at	1368304_at	1368321_at	1368335_at
DD81	722.7518	74.26097	3.171292	3.4764087	94.03865	122.61321	637.3301	490.9928
DD84	10.06244	74.12043	24.563402	30.651281	93.53027	126.29009	341.2901	9.859883
DD85	22.273487	108.20042	1.8686885	3.1753516	19.177357	159.50041	260.81546	19.505405
DD86	7.6562366	13.410315	43.019024	1.6320655	51.55996	132.7831	539.8571	17.121784
DD83	5.409511	74.495255	7.304327	2.8897069	55.434227	202.73465	271.41162	8.396073
DD41	0.8164172	152.27332	67.44744	2.576194	54.664665	212.89703	567.18066	16.677704
DD42	0.9457556	213.91774	68.64033	1.7601386	57.28403	184.1765	344.28867	2.8630395
DD43	1.12043	19.768225	100.93182	2.3099594	513.60114	161.43906	438.6868	8.428677
DD44	11.740425	25.060024	55.310814	17.145533	433.17557	169.29646	351.896	10.324244
DD87	9.729641	146.88496	107.85883	8.148836	70.01627	313.2955	606.34094	13.946005
DD2	0.7638486	38.047207	159.3261	1.1481682	276.31607	284.06613	1464.7104	1.9193712
DD3	16.757408	11.614359	228.01321	1.8644352	78.76558	335.59476	527.93036	13.050907
DD89	2.3955595	24.42907	134.36726	0.8966065	64.68336	314.48416	455.66055	2.3684564
DD90	20.762367	9.252334	155.58202	78.06702	166.16005	260.335	1037.438	9.517207
DD4	8.028825	38.8189	301.03958	4.040441	132.01106	300.71442	1496.5269	11.343261
DD47	112.25358	138.32532	132.77325	3.3444555	59.255592	187.68263	759.6769	60.47566
DD48	245.82797	81.58969	138.17374	7.608033	40.06532	201.79994	1458.7935	167.257
DD49	80.79758	17.0419	367.04932	2.519014	63.594273	202.50484	1065.7975	45.670708
DD50	91.76575	20.528175	329.2852	4.7421308	108.88184	193.13554	1784.7737	52.515175
DD46	95.52521	18.868633	232.01816	6.4792686	68.78378	279.6568	1163.496	72.75595
DD6	0.8268442	37.696125	196.2183	1.9527689	168.15059	306.60403	468.20898	3.0281148
DD7	0.9347613	6.4338923	307.3885	1.291365	100.55962	342.46133	1689.8435	7.2884026
DD8	13.139592	13.935559	249.91795	1.0525507	79.59947	314.83395	1464.5326	2.388246
DD5	2.8977234	12.409314	265.00946	4.578764	71.95036	322.90518	987.5162	11.630174
DD9	1.5885129	14.535978	377.84097	4.587891	113.51428	292.7837	1277.0565	10.065575
DD103	53.510674	36.866722	732.9285	2.8353586	133.33105	61.648228	788.01904	27.363947
DD101	43.908257	85.58198	211.68428	3.4703617	80.120575	73.093834	539.9619	50.304462
DD102	213.0092	30.254078	227.52841	9.982483	260.60876	51.832592	525.73676	172.76259
DD105	61.566288	93.343254	46.912365	11.458332	63.391342	52.264126	479.58295	54.359844
DD106	198.8751	93.376045	105.49486	2.6890495	124.14363	79.14703	382.63474	165.1127
DD62	1.8393811	12.717304	165.21786	40.955788	125.2462	55.308964	259.95557	0.9038308
DD64	2.0406353	17.546946	419.12888	66.78599	306.16653	50.400784	559.2201	10.372576
DD107	0.9328123	19.038765	239.72949	40.98023	690.85095	48.17711	401.29865	0.7978045
DD108	1.0428171	12.300408	249.99156	145.011	423.77084	65.16325	384.21597	14.780329
DD63	1.1053882	20.518663	249.36295	147.34642	404.85406	66.99039	373.73013	5.275702
DD23	1.5211728	9.482892	480.06833	28.111277	175.72983	127.68943	292.64066	4.104058
DD24	3.513082	2.8095315	657.9176	29.422213	132.88458	143.71027	395.1862	3.5311084
DD110	0.5948447	22.392834	554.07806	113.60464	234.97432	154.39307	290.82953	10.697882
DD22	0.8395089	9.344945	744.7059	37.921814	147.4388	143.4945	339.66013	3.7688155
DD109	0.7853183	12.33136	482.80237	71.13938	102.40072	55.9909	354.81177	1.1170738
DD65	1.7731179	3.437914	503.57123	112.71291	274.4203	71.87348	566.6441	5.6422663
DD66	1.3141295	93.24102	480.32672	1.4775469	93.92953	62.844658	607.575	0.7978019
DD67	663.49176	36.815613	742.9837	2.678683	59.358624	110.42431	545.7143	730.0398
DD69	4.145628	1.6846915	407.97095	97.79031	330.2382	72.569695	433.60455	20.211977
DD68	63.65963	11.226022	638.0221	1.4283706	160.56914	74.57099	483.91898	19.216692
DD25	1.1638397	92.22946	857.29443	4.157742	99.765564	69.683075	427.21954	2.370976
DD28	1.6432819	9.251738	465.711	371.33746	371.77493	62.81055	933.34863	2.9899843
DD29	1.3563819	32.948635	693.6673	392.93375	354.88782	40.944065	738.3266	11.733833
DD27	0.6892783	56.942997	443.88013	10.435717	405.01294	47.384842	463.56146	2.9732046
DD30	0.9206118	80.20251	933.2933	11.4073	95.045265	85.284256	435.13046	1.4450653

On-Line Supplementary Table 1

Animal	1368337_at	1368338_at	1368397_at	1368440_at	1368459_at	1368467_at	1368569_at	1368578_at
DD81	289.9001	64.666405	188.6106	91.17219	31.790438	144.50874	12.661428	1.8129219
DD84	221.04565	85.46277	3.2618773	62.599506	51.980408	9.1220255	2.6691616	2.3336322
DD85	42.731888	111.9361	25.592457	95.02259	52.513725	29.812096	82.17191	106.70512
DD86	32.467014	187.38821	8.595783	27.16073	83.473625	15.005347	10.709218	1.1735946
DD83	99.88903	81.903244	1.4090371	70.64071	52.854336	17.371489	14.749071	1.5989914
DD41	33.565556	170.96585	8.722071	43.548336	24.481302	21.950438	4632.627	5501.063
DD42	27.208542	195.60431	5.6237907	33.87267	46.12935	17.582893	6146.6577	6148.539
DD43	1196.1807	193.59636	5.7418556	29.614752	43.322056	9.81255	90.18877	114.51661
DD44	843.45544	71.1204	8.282434	38.718853	44.039967	18.481825	11.080767	1.6514432
DD87	37.624855	216.43756	10.462839	49.398487	69.981964	21.247828	4338.0264	5024.1064
DD2	712.8467	170.06996	5.926079	47.407387	103.07846	7.0931783	1799.9381	2764.0427
DD3	49.464275	205.69562	10.593754	34.11118	120.605	19.638832	34.471104	50.312317
DD89	43.743893	162.71281	7.7135844	56.201256	85.88822	11.218901	2.6162186	9.5522995
DD90	45.082794	191.0268	13.530329	75.41815	82.30053	16.98357	3.0903497	14.38529
DD4	33.25029	573.4374	47.56704	126.24542	102.29578	30.21258	5.588115	8.925694
DD47	41.849514	147.03528	51.223602	76.66921	93.18844	56.073128	5881.275	7182.3696
DD48	17.819805	101.31214	64.563225	57.3106	117.91116	85.662636	2902.1333	3644.6243
DD49	44.64091	143.4071	27.287477	33.858887	166.3221	30.094906	29.443182	37.691837
DD50	34.774372	228.52792	24.985233	32.152027	175.4655	39.746	3.7426372	18.372524
DD46	34.254692	82.59871	27.581507	37.14008	160.26544	42.597023	6.017493	0.7262656
DD6	472.11972	81.216064	7.0483837	48.283974	139.23384	12.92732	3.1640773	1.0067675
DD7	138.82835	121.84296	0.9512848	41.073524	150.84436	14.064523	0.8748298	0.9992673
DD8	22.278576	163.99136	3.0045917	37.427227	164.18225	7.908559	1.1663244	2.0795314
DD5	53.33149	211.8014	0.2244047	38.024555	176.16623	12.437593	2.6997383	1.53399
DD9	131.18399	335.95126	3.8523767	33.321243	148.51108	10.752923	1.6589065	0.833186
DD103	370.20047	145.319	19.726423	68.91426	69.264626	20.407213	0.3521659	1.0906729
DD101	255.72943	77.7737	18.28454	151.06578	38.74316	25.925268	1.8756707	1.1878871
DD102	698.3159	47.5848	71.31475	147.42517	58.67269	69.851234	15.146559	2.4039135
DD105	92.37921	97.19144	24.579857	140.83118	33.020687	30.018686	3.01392	0.9598963
DD106	425.19473	119.34112	79.36823	117.67726	46.598053	72.42464	0.9653171	1.8807414
DD62	188.08643	60.303272	5.5670133	241.19109	31.918566	4.877973	5.241453	7.116255
DD64	519.2864	94.06354	9.119138	145.01213	41.23551	1.5111983	12.620415	1.6990092
DD107	1588.4841	73.21988	2.88947	101.89285	38.833458	19.625349	7.247127	3.5345666
DD108	667.2176	65.33391	0.8890764	94.008705	51.489555	15.715767	6.383274	6.283968
DD63	651.8981	59.934917	5.075847	102.30704	43.877865	15.086656	5.906436	0.9720676
DD23	258.5288	42.572754	5.0775404	48.742928	47.714252	10.51933	7.9929013	6.007381
DD24	148.9145	47.82074	5.6852617	26.660295	52.2492	7.2423477	0.9108841	1.8510877
DD110	70.10374	51.140953	4.9753976	64.00803	38.78818	7.8079457	1.2411644	3.971649
DD22	19.460545	26.67276	3.4624262	56.436714	43.383343	2.6239023	8.355228	0.8474373
DD109	31.026644	48.645237	6.130781	108.16466	56.98842	5.721555	6.4779835	6.402008
DD65	200.55995	80.87957	3.5513816	80.91184	75.526245	1.2283715	8.50188	1.3093768
DD66	73.896965	88.91828	7.5583186	54.002647	58.19139	12.67058	3503.2563	4438.171
DD67	32.586346	25.637217	247.80103	98.34129	70.42449	178.00935	33.899822	35.555622
DD69	232.58504	48.76542	6.856467	50.859047	59.633816	3.8582294	12.219997	4.2600875
DD68	164.31146	67.13824	15.250486	75.1595	63.075146	19.716291	2.3686864	8.893754
DD25	60.51838	57.836067	0.293429	111.24707	80.977936	11.638601	4319.7017	4093.1077
DD28	46.936962	230.17517	7.180888	97.63917	94.45231	10.607262	21.543982	3.9995975
DD29	84.75182	47.67554	0.1819494	114.33521	68.42332	8.723432	3296.02	3770.4
DD27	960.6749	48.329712	0.5957001	60.419464	81.23684	7.1414495	3730.167	4092.8223
DD30	59.310394	57.298576	0.5388237	108.43222	109.86568	9.011086	4395.786	4085.1636

On-Line Supplementary Table 1

Animal	1368587_at	1368637_at	1368683_at	1368738_at	1368739_s_	1368762_at	1368826_at	1368872_a_
DD81	663.0927	20.577198	2.4814365	13.289753	25.355318	75.88906	545.4769	109.49532
DD84	4.221883	20.359304	1.028343	3.1733646	18.53273	54.74689	354.9271	107.53772
DD85	44.21731	33.510677	1.094843	50.265663	25.80424	6.8599253	380.88275	112.83752
DD86	13.348442	21.596542	10.789923	7.703233	5.767036	35.875904	438.2872	96.55819
DD83	7.2929754	22.498621	1.8339955	8.429836	9.069409	28.24975	327.6143	115.56422
DD41	27.938053	46.219425	12.746195	1863.7963	1449.3628	11.790928	507.19925	66.76448
DD42	84.500206	46.01476	11.249397	3658.5059	3211.241	30.128923	512.9705	64.58425
DD43	27.018799	19.385332	11.221581	37.904156	13.298955	192.72375	558.50397	63.471497
DD44	23.089495	33.404015	13.486149	10.003321	4.478846	182.17148	539.1995	77.6055
DD87	37.14045	45.004192	4.791059	2024.559	1657.7749	23.159725	506.76617	86.88008
DD2	10.826036	6.021937	28.420832	599.06775	421.15842	156.23375	456.7512	65.45137
DD3	24.966078	14.165264	44.929516	10.723003	16.162495	25.759008	623.4801	78.23732
DD89	8.422922	5.5353494	37.052235	2.7416272	3.1938314	21.381746	509.93152	71.655655
DD90	31.511526	11.950406	28.930304	1.3113116	1.1569272	26.594206	588.20667	58.36917
DD4	15.959617	18.375896	39.652027	11.125738	7.6026363	40.39153	643.93036	67.90967
DD47	277.10648	30.6559	19.484394	2396.862	1722.8784	20.59969	586.92896	77.90795
DD48	715.1747	4.5703273	34.260536	691.2763	508.8072	15.587254	541.565	90.30157
DD49	139.8207	13.487278	29.937344	30.431126	9.527108	43.84117	599.43054	61.511497
DD50	145.67332	15.086115	45.54049	3.2327912	4.374193	51.361763	636.3191	91.91565
DD46	187.49551	16.780212	46.41617	7.5075235	2.3925152	8.99695	525.64343	68.37867
DD6	11.513859	19.465113	37.732136	6.362398	19.500303	102.81564	450.59787	58.99597
DD7	7.8657613	2.2505476	45.32875	7.1787457	8.501357	57.02787	484.8385	72.73984
DD8	1.8283981	21.409279	22.932568	6.7555456	1.9798865	18.281794	486.84003	96.04888
DD5	1.4034292	4.2955203	31.164417	2.9160159	10.347198	49.634678	498.8593	84.58612
DD9	1.5504953	16.947874	34.985176	4.857217	4.366651	45.9694	565.7655	89.59653
DD103	61.96138	47.70543	1.9857705	6.1332326	3.91962	121.35973	66.323296	266.88123
DD101	89.6735	81.55028	1.5743068	2.6015162	2.4915817	56.122658	48.080524	273.28137
DD102	331.09045	38.755733	2.555838	1.0224284	1.3914195	212.07167	61.4261	301.98358
DD105	52.639664	38.670017	0.4899974	2.2927465	9.930734	50.267612	46.40425	340.50974
DD106	265.21286	118.14403	0.5248975	5.1786704	9.269126	109.76688	58.505573	313.591
DD62	1.5687625	87.27556	0.8541289	5.696248	2.0815496	46.177765	250.54143	144.89159
DD64	6.001854	67.09632	10.545517	1.1226412	2.5383348	134.44827	80.2606	234.05247
DD107	30.014875	22.202602	6.4218016	1.2253166	3.709738	444.33273	76.6305	122.98529
DD108	20.045671	48.266506	0.7060408	0.4886201	4.184276	178.76215	81.43227	100.86862
DD63	11.244562	31.034971	0.8945209	8.863817	14.005132	179.04485	85.37325	89.90502
DD23	1.6704941	47.952633	7.746751	6.1698976	4.0997415	63.541252	93.49228	222.91667
DD24	0.9471274	56.941723	9.222992	1.8918586	1.5139089	89.7582	78.64776	312.56854
DD110	0.978188	29.164505	1.2284144	0.5852014	1.4320508	55.102215	93.943924	240.8991
DD22	0.608635	50.52522	7.6014347	2.0866206	3.013612	46.60076	71.723145	98.637024
DD109	0.8195126	47.06456	13.844228	4.751982	2.2156599	40.586792	74.99756	191.47874
DD65	8.448549	44.00747	32.979134	0.8329345	9.121814	68.97428	88.524765	285.28485
DD66	9.702762	65.963905	4.2656846	1317.3234	842.75714	71.280525	92.56604	232.7065
DD67	1531.994	47.09389	7.9520516	11.402846	9.71922	124.93944	119.6145	261.96027
DD69	11.257134	28.467772	9.097939	3.31674	2.7356155	102.1514	83.4595	375.67572
DD68	94.88206	34.882828	24.899517	8.840942	2.8619492	171.10081	89.64145	312.5732
DD25	11.676512	33.76743	42.537067	1005.4726	745.9928	31.216497	86.8987	239.86673
DD28	0.7184167	36.775948	69.548904	8.6125145	8.680647	63.64453	69.11511	196.57187
DD29	17.974703	52.094353	28.779818	1064.9642	783.9778	49.134743	702.11273	205.6528
DD27	23.597866	30.310719	20.092863	1207.0114	835.82043	198.21426	363.13953	154.4772
DD30	10.793693	49.409065	27.071344	1047.6378	740.28516	16.530104	91.091835	232.80597

On-Line Supplementary Table 1

Animal	1368990_at	1369064_a_	1369067_at	1369097_s_	1369108_at	1369110_x_	1369111_at	1369171_at
DD81	83.739174	5.414654	39.774445	46.207344	2.1285708	788.77203	861.5994	46.71701
DD84	56.263084	5.8690066	37.771496	53.8148	14.72261	594.53064	16.686512	45.41062
DD85	100.04899	7.829096	41.906803	42.798847	8.934909	517.05884	28.169147	47.260128
DD86	89.12944	11.15008	44.893406	69.68394	1.400793	586.3791	16.385284	83.3354
DD83	86.15031	11.158986	30.563248	52.323696	1.8206983	523.3025	8.918026	42.40627
DD41	2911.8042	1.4381095	294.27997	73.49929	9.553959	1429.7327	2.3685362	54.348804
DD42	4120.751	8.116864	773.57477	59.490795	17.747849	2039.3754	12.132442	49.731045
DD43	151.37508	0.9707092	80.81806	52.23463	15.622512	2159.421	13.459697	31.85271
DD44	164.74902	6.543384	79.3297	52.3358	10.163265	1786.0443	10.786601	30.440754
DD87	2932.693	11.141089	205.20158	57.36053	2.914977	1570.4535	14.089105	50.12559
DD2	1282.6837	7.616652	177.64934	56.065853	5.125859	1758.4119	0.5568506	35.156727
DD3	472.0436	8.205826	60.19378	68.92077	7.0964837	1465.5663	29.2256	55.066593
DD89	427.79617	14.906868	48.627407	67.58172	9.822202	1422.3116	0.7159833	52.947876
DD90	388.54614	9.7143545	37.957066	75.99469	41.449463	1429.9847	39.871216	58.57627
DD4	375.58524	15.091843	31.36334	81.516914	1.7528807	1810.903	7.7606306	65.40306
DD47	3577.8044	14.00372	55.44079	52.411594	1.9607236	1705.7117	86.871544	61.445816
DD48	1686.2229	3.6420655	87.21072	48.25889	2.084964	1303.9646	385.89813	46.145256
DD49	639.1503	5.8428993	38.395935	59.544262	8.152246	1581.3082	77.26975	52.72558
DD50	536.4196	2.0902884	27.993185	87.7995	3.7626243	1678.8383	70.42939	76.63562
DD46	533.78735	0.6025128	36.858536	75.166115	13.976118	1599.0637	157.9398	58.983227
DD6	444.94772	1.5643791	62.18167	66.5776	6.0375495	1298.1859	2.2907076	58.344803
DD7	466.7774	0.8291294	35.528492	93.34167	4.302255	1385.001	1.0271207	63.007233
DD8	507.5565	0.6807905	27.085068	77.19572	6.495521	1052.3312	1.4820386	64.14961
DD5	621.1764	2.6748495	23.989626	60.424767	12.988694	1405.0194	14.448876	72.754944
DD9	664.6064	3.5629098	35.94653	68.066505	7.121839	1435.81	1.7369229	46.575806
DD103	73.40132	12.757452	14.648063	138.24709	8.75333	116.17346	36.736546	368.74188
DD101	70.49455	24.425282	16.515963	108.90315	11.244175	77.51706	50.600853	125.21148
DD102	96.942276	20.833067	18.701248	141.76904	0.9128553	235.3933	159.97142	276.97748
DD105	59.273033	33.48912	38.058735	117.63944	16.614985	93.5105	57.881325	245.58546
DD106	53.552654	21.346956	15.8946	116.98392	4.119011	119.01496	125.7396	134.29135
DD62	97.60556	14.922228	22.717457	108.60412	19.631735	118.8894	2.448699	242.06973
DD64	184.91367	22.366514	25.195463	119.49348	32.66604	186.41331	2.3190057	489.05984
DD107	225.89182	15.862109	51.786938	96.08447	26.194513	192.62177	0.676759	436.93567
DD108	180.65344	4.24541	18.546553	131.54466	26.375086	165.00746	2.1925368	390.65775
DD63	161.99379	14.052715	30.701668	109.33703	34.43792	181.49759	0.8626124	392.72192
DD23	142.47472	31.022173	36.607624	109.46468	12.355687	217.79364	7.339003	578.8416
DD24	208.35625	23.019577	24.340021	140.64503	12.470469	201.9182	1.7742351	309.35815
DD110	272.23618	10.839427	19.556797	116.50235	44.00538	187.52968	9.826363	356.97504
DD22	207.8384	16.30066	29.553944	111.77302	24.280209	146.1464	0.7485198	379.40054
DD109	171.38669	17.79427	9.858716	117.13275	7.5850677	190.64467	2.129211	72.27839
DD65	278.8269	19.27944	14.956653	125.68464	36.31887	188.39153	5.972441	636.74506
DD66	2447.7302	18.36106	72.608185	108.01276	9.721092	156.85765	1.4958371	577.57764
DD67	180.01128	36.13211	13.353498	113.29829	7.3104196	169.24623	1466.614	635.31866
DD69	260.2144	5.7074375	19.60907	130.33385	42.21507	216.00005	11.180977	613.1316
DD68	362.37067	25.793451	31.044464	130.84444	2.375922	183.1789	67.329506	348.074
DD25	2409.394	22.699192	28.150166	126.15137	1.8504245	252.72887	5.1230874	528.42175
DD28	335.86426	17.616627	28.609184	116.60767	75.64444	85.875694	1.623559	580.9581
DD29	2387.2412	25.148296	163.36105	126.85809	80.86636	111.55343	0.7766997	301.72397
DD27	2281.125	13.707618	70.55957	93.372765	11.842082	208.84796	3.9251568	276.94717
DD30	2299.9482	22.691145	37.316895	120.61713	13.835184	222.76341	6.6352243	600.82983

On-Line Supplementary Table 1

Animal	1369202_at	1369264_at	1369322_at	1369326_at	1369410_at	1369502_a_	1369550_at	1369580_at
DD81	245.07733	21.724403	75.69834	74.45346	26.078468	121.75211	127.63609	12.233611
DD84	232.27628	13.380151	70.15262	61.836315	12.277297	5.912501	100.00466	8.705824
DD85	232.9794	62.62988	72.91281	81.1029	30.163353	10.376532	94.98937	15.4693
DD86	238.7014	6.2025566	101.32238	6.226543	14.571496	0.5161745	14.51004	16.788507
DD83	221.14772	23.815018	76.70796	71.99573	17.963255	9.396245	131.65573	8.99815
DD41	318.43607	2459.1748	75.43717	21.591915	227.32445	12.904836	49.645573	0.8937936
DD42	295.5304	4798.3506	68.91434	9.106515	166.42372	3.855392	23.441738	9.390335
DD43	477.8351	65.80922	65.97737	9.3650255	85.611374	0.6577773	18.028868	6.563694
DD44	435.5182	11.049287	94.7892	13.562627	158.8158	12.351228	37.354355	9.756395
DD87	365.68027	3152.0159	91.51883	14.626191	61.54047	0.6571577	35.07202	5.88338
DD2	190.49255	825.37573	57.633144	30.464659	134.15607	30.555084	68.57126	1.4309052
DD3	185.12077	33.31449	63.327717	6.077711	26.892319	8.659109	22.330605	8.042682
DD89	298.61032	16.41431	77.32502	3.4331455	191.5143	0.6899982	30.791594	7.909811
DD90	211.16742	16.051693	62.522602	10.126095	193.64995	0.4530764	17.123098	11.481388
DD4	249.45222	7.827629	70.7716	4.075655	234.61543	0.6840399	21.023415	15.112892
DD47	164.46722	5224.657	56.25882	2.021469	30.859535	18.292969	2.0451071	6.5546117
DD48	121.62698	1853.5928	45.513916	39.07934	60.608433	78.91631	64.73608	8.17509
DD49	199.38687	33.3574	34.717674	5.006853	35.250587	11.830732	17.09056	2.9236352
DD50	166.65683	15.823844	47.703114	5.6793013	47.594494	17.808989	1.6956122	15.883904
DD46	178.9987	14.239893	74.10372	15.279716	136.16663	39.19344	40.43504	8.973351
DD6	163.9942	5.4374795	68.02534	19.419443	805.03107	37.108673	67.06277	1.7321545
DD7	203.04553	14.230872	52.618504	11.38538	993.54095	5.6448812	19.734186	3.0230982
DD8	117.5101	16.737555	66.182755	11.656505	1107.0105	0.2623377	3.1140497	11.352025
DD5	276.42743	13.995203	66.20327	2.42317	615.90436	1.5558665	3.0165198	11.78099
DD9	262.48447	15.442686	77.40545	1.5758123	709.29114	0.3137922	11.829628	16.215574
DD103	123.94713	6.016505	4.356667	28.0829	38.348053	22.642038	31.692623	7.5382724
DD101	35.71051	11.703557	13.612961	50.738617	32.460964	22.487026	88.30814	1.0162796
DD102	128.85777	20.374075	4.552811	26.057907	37.550877	49.531387	24.666107	3.668863
DD105	63.120155	6.5303226	41.50204	64.95732	35.44806	20.396202	54.182495	1.4478713
DD106	42.236656	4.2955203	18.844053	51.168877	33.5618	51.024364	55.681835	9.90762
DD62	109.8634	24.06856	59.063976	63.21829	145.70346	0.6231259	70.25511	7.3185935
DD64	251.50368	10.818113	2.9341018	17.771973	140.54288	3.1900463	30.208391	10.440002
DD107	11.940066	7.9259486	69.62581	3.9391477	169.62823	0.5099187	12.580496	14.175442
DD108	152.43976	17.085472	37.225777	15.434454	22.082945	0.4124042	9.751037	6.3836417
DD63	158.96521	9.982012	47.921036	3.9767594	19.05	0.5656042	13.302007	18.002275
DD23	169.69646	6.496089	19.280361	1.9361655	82.61727	0.6449747	2.5587838	10.756195
DD24	149.02115	3.914632	20.62778	2.7807317	135.43768	0.8049998	12.346878	1.3898692
DD110	150.88673	22.009424	20.543892	18.874748	93.011925	0.5067629	29.635183	1.4660958
DD22	113.67917	7.8003917	3.5055091	9.757129	47.41093	1.4867052	19.511742	4.1246977
DD109	164.3982	21.2534	6.4279118	1.5934105	27.848906	0.7433506	4.171151	9.40783
DD65	275.43967	13.908149	8.542261	2.954873	26.938866	0.6057913	0.9066727	4.3915963
DD66	131.23636	2637.6284	76.34306	2.136175	34.608814	0.4753617	9.791383	12.936584
DD67	85.04879	19.570026	12.620234	0.7043325	22.247623	177.69415	12.413174	9.450562
DD69	6.261025	17.5952	29.022488	0.7184297	40.049995	0.696319	5.4775023	5.1908503
DD68	151.97601	12.126663	3.49204	6.2910776	39.607697	3.7732415	2.1924453	1.3729657
DD25	8.924156	2010.5995	23.085314	2.6686687	35.02621	3.0679839	2.1736634	6.723899
DD28	65.3133	18.084967	41.685493	4.9961796	56.331562	5.964031	1.542293	3.9810736
DD29	151.77037	1854.3369	12.036922	3.1969433	24.087002	4.4219737	0.7455321	0.8340449
DD27	99.33479	2347.8096	27.431143	0.8600261	47.951534	0.5206428	16.246843	3.368712
DD30	9.71143	2310.42	11.065615	3.7437346	43.897957	0.7234372	2.246507	4.8631887

On-Line Supplementary Table 1

Animal	1369594_at	1369663_at	1369680_at	1369698_at	1369716_s_	1369764_at	1369773_at	1369836_at
DD81	6.9968457	42.38794	33.604652	19.44584	1152.5177	102.98866	58.976357	169.59082
DD84	2.2230392	52.501736	30.025059	48.70129	1210.8591	5.912685	59.12333	173.96553
DD85	10.713901	31.875168	31.00477	61.242413	1339.026	7.8657503	78.526436	204.50154
DD86	17.76071	85.35335	22.261623	108.8751	2021.7672	15.158857	102.83738	234.57576
DD83	21.9475	53.83728	40.744495	61.07677	1017.4965	1.4137487	104.58305	175.74452
DD41	1.5856159	44.683147	33.67381	61.926113	2767.5222	3.132999	83.69034	215.74237
DD42	1.382153	33.190807	32.917923	97.4053	2984.3584	2.8622875	85.663506	215.32951
DD43	1.3976333	29.234196	32.940594	57.906143	2409.139	8.576916	68.41426	218.08485
DD44	0.8392505	35.292847	35.07521	72.361176	1259.3627	7.2139335	93.14048	191.33676
DD87	4.358722	54.047207	36.937244	87.1321	2953.471	11.317701	101.42589	235.78247
DD2	6.268439	43.427685	33.60543	58.47672	2347.4053	12.729174	53.367382	123.88592
DD3	18.356995	84.36619	41.68431	101.74027	2538.2542	22.6179	82.07012	148.566
DD89	2.3934975	55.32418	57.06499	15.537485	2471.4517	12.317236	93.9808	128.01633
DD90	0.9267829	70.03603	55.41734	21.847637	2746.639	50.571934	74.65486	119.32593
DD4	1.8292264	48.520065	50.59194	43.051933	4660.499	31.219107	73.31458	252.8486
DD47	21.960047	26.704748	58.658012	129.03062	2700.8525	37.640064	77.73947	143.31918
DD48	17.005259	36.46592	45.673065	68.379395	1165.8004	63.66726	52.72058	95.20834
DD49	30.181658	63.49036	52.129562	127.07153	1990.2289	19.65433	72.66094	141.06927
DD50	32.568527	44.403477	50.474854	98.152824	2518.2463	27.603163	49.885067	154.07137
DD46	10.734201	58.385807	69.06576	68.46137	1315.194	44.593616	82.10052	128.72511
DD6	9.834207	77.20288	48.07857	38.396088	1228.1978	19.76691	46.673576	128.02174
DD7	2.2642832	74.74751	53.377666	44.21044	1993.219	33.60532	55.68238	128.5388
DD8	14.339042	69.781044	56.333534	81.60743	2087.9077	23.853226	58.61898	140.32555
DD5	2.4714382	58.786766	58.262142	51.534863	2684.8098	33.73497	71.59716	169.82149
DD9	5.112701	44.727585	50.888405	84.3224	3488.435	27.823038	78.769806	202.62045
DD103	6.5555563	465.41367	55.94852	166.64041	378.4131	36.89304	125.63733	40.975323
DD101	10.891221	351.608	38.818428	132.05759	171.23004	21.406614	130.75269	31.695034
DD102	18.204592	296.34625	38.35404	116.19766	277.4187	56.402447	90.46539	36.578846
DD105	19.860556	207.22563	59.904396	110.79619	299.3285	27.906433	83.29928	31.973848
DD106	15.718832	424.87305	62.31559	132.73271	205.14207	50.97299	81.06002	40.790092
DD62	15.469016	337.21896	69.09328	55.89363	374.56595	22.910212	145.30489	16.673641
DD64	20.551964	583.99396	73.317604	60.98677	601.0023	66.93863	158.02763	30.291637
DD107	10.1491	282.52682	88.35432	70.626076	377.9722	54.88072	121.96531	16.1237
DD108	17.90963	402.69577	73.989136	103.33551	297.6701	23.672285	169.681	23.005545
DD63	13.361192	381.49643	71.06594	103.83961	286.2514	34.682167	176.03752	34.630863
DD23	22.23215	324.8938	103.75947	100.60654	369.4865	99.0996	136.8871	24.353659
DD24	7.982077	288.7138	95.96532	104.65794	344.35767	59.935875	141.07854	20.98971
DD110	22.992958	597.3029	168.64365	92.37938	327.7906	88.33033	273.77484	20.95434
DD22	27.109362	350.76025	94.26069	92.68427	281.97842	96.77992	149.94212	16.909243
DD109	15.317527	350.47345	130.47736	111.28536	387.11996	151.89294	138.64728	15.444527
DD65	19.28	641.9086	193.0433	105.74126	421.5507	111.1284	216.45207	26.620659
DD66	19.076065	492.76944	119.7963	121.73486	321.44226	64.56799	137.63458	25.304392
DD67	19.271282	305.32523	54.697853	93.78729	245.20302	387.3457	56.992733	20.80466
DD69	21.341204	630.477	145.33076	103.01286	360.08807	144.31166	201.83536	12.901081
DD68	18.024807	277.61578	192.02576	85.0581	385.58798	76.91797	177.66966	21.29842
DD25	24.807487	386.73254	128.04134	117.07303	320.0659	101.20402	129.82826	15.87714
DD28	26.09063	639.82837	154.18048	134.19962	399.64035	100.492	197.69843	26.773933
DD29	37.89806	557.4118	140.45348	145.62329	253.17061	63.012783	228.46388	13.936613
DD27	29.74581	467.2267	139.4869	123.39124	398.43082	89.79988	191.55943	15.90207
DD30	33.543777	399.22668	143.98912	83.58363	344.80856	116.97657	148.56693	15.164645

On-Line Supplementary Table 1

Animal	1369866_at	1369977_at	1370027_a_	1370067_at	1370114_a_	1370115_at	1370138_at	1370176_at
DD81	13.122837	5.062713	937.4145	5675.5664	7.523042	159.13947	33.34831	670.15784
DD84	1.6640356	15.176305	12.771459	6471.687	48.95764	133.26462	4.4144096	555.86957
DD85	11.511303	23.273945	47.182167	6104.9927	11.717298	161.26347	6.879061	656.7611
DD86	10.948736	3.830705	2.3659227	7645.621	43.13006	221.3105	16.794172	456.47144
DD83	9.909673	52.415417	1.2672812	5722.6255	13.958579	151.17778	3.125597	530.4313
DD41	13.190933	47.086838	2.3974674	5130.2637	70.13612	82.342316	3.8379774	530.8978
DD42	26.306067	70.078606	3.529587	5286.273	39.44108	90.78422	9.218726	427.74554
DD43	21.64199	26.868612	2.650395	4903.63	61.293613	100.92943	217.87433	412.1434
DD44	0.9399916	65.1886	2.455608	5148.3486	57.805527	135.36664	130.66637	391.78574
DD87	15.228809	77.79127	3.6089919	5060.9995	46.019913	128.44926	3.600754	451.29907
DD2	8.611647	13.624579	2.910506	630.1725	36.93428	78.98649	70.62217	471.31006
DD3	12.789961	19.253298	17.621119	979.2825	58.94312	175.16931	3.3518171	504.45056
DD89	1.9682493	15.921208	0.9911005	1282.3009	13.080525	148.1469	3.4010494	702.609
DD90	1.7939607	128.52505	36.31625	826.80676	14.910694	81.10052	19.15427	762.22845
DD4	6.278404	11.833762	1.6086642	758.8374	25.256054	114.46719	2.772239	624.7487
DD47	14.206882	63.982452	218.1464	1269.9786	33.082714	219.91504	7.376485	440.34818
DD48	3.4933732	23.305162	410.2262	859.44806	52.83449	155.0729	3.9437084	467.18127
DD49	3.1183314	13.715027	105.82259	512.69507	56.628323	143.93362	4.5485473	391.72916
DD50	17.059122	46.881706	135.47092	438.8138	58.5259	114.40047	9.677782	535.24255
DD46	16.718636	21.409096	208.62315	515.243	37.04121	110.42563	3.9794073	455.46472
DD6	5.788453	2.9261496	4.5092287	468.16812	34.039402	81.11748	47.568832	445.69925
DD7	10.819225	10.349741	0.8317078	437.00262	8.222206	104.60478	14.755189	475.94684
DD8	15.095942	3.063482	1.5734092	589.35077	28.856943	126.05327	16.88173	407.595
DD5	7.239935	21.570288	2.2260375	540.18677	20.589586	129.31538	5.3559995	336.2976
DD9	7.576019	18.4206	2.4558828	537.16437	7.8376923	191.9143	31.8081	422.96738
DD103	25.248089	26.568491	111.81434	6621.7695	255.81496	1007.886	57.051407	370.62332
DD101	14.916329	34.66257	64.77348	6263.856	213.2966	590.777	15.513131	176.6805
DD102	19.794596	48.780437	199.32993	5488.7593	363.2851	516.4698	107.827	378.9473
DD105	25.22365	36.350395	63.771606	5627.5225	336.10577	429.49396	4.954407	190.63367
DD106	29.371872	35.208904	245.3333	5591.86	273.00842	659.6667	32.721317	159.77303
DD62	29.316023	77.837616	1.0985317	3668.1633	437.59708	362.24426	15.479818	133.07176
DD64	18.592308	174.21301	1.8528316	2983.6265	485.31604	455.69394	51.602688	268.5631
DD107	24.253958	95.66081	1.0946683	1976.8812	454.30432	355.7544	120.60932	232.65244
DD108	21.194527	209.6335	2.283949	4595.4277	425.4825	817.8231	77.38516	157.06694
DD63	16.131214	214.42111	0.9655278	4619.5073	445.36807	771.12494	78.86511	132.9127
DD23	27.574888	83.65289	2.8869827	3669.4631	462.54376	672.6828	47.68341	156.76518
DD24	23.85324	68.71833	1.0828668	3726.0986	417.57532	905.55743	16.380835	295.56424
DD110	28.481192	216.8136	0.9422705	2103.744	493.4198	777.1936	2.9779558	274.2233
DD22	20.438707	152.00398	1.2200812	2784.8333	520.5851	951.978	8.876505	266.0089
DD109	16.461918	60.974087	1.5118476	2886.7314	454.24893	668.8527	25.194918	238.5159
DD65	33.47395	197.62883	1.2553477	1641.9191	322.75677	829.1847	32.606636	115.6405
DD66	20.506277	69.02837	2.0061128	2637.7556	269.78558	1149.4941	13.364449	81.37949
DD67	24.18234	12.199116	1906.8878	4278.228	284.1692	766.0447	4.1301684	231.67508
DD69	31.137827	287.36313	1.9336305	1347.7996	334.97867	624.9657	40.498653	136.88216
DD68	16.099144	27.013866	121.41328	1418.547	407.2648	1143.4545	37.44576	253.16231
DD25	27.016499	68.9278	0.9922176	1190.4216	427.48114	640.45685	29.795591	148.1138
DD28	22.52763	307.3072	0.8716708	1277.9492	327.71	838.0647	2.8365242	122.59476
DD29	21.586964	364.6061	7.6641192	2183.6655	359.99924	645.4591	9.1776	262.5884
DD27	17.656342	31.042162	0.8266343	1178.7227	362.71872	870.3957	97.343956	224.15002
DD30	21.946548	51.72154	1.5937432	1229.7125	430.38947	665.0095	29.524021	124.57657

On-Line Supplementary Table 1

Animal	1370198_at	1370216_at	1370224_at	1370349_a_	1370350_x_	1370355_at	1370359_at	1370377_at
DD81	3139.474	56.863632	109.01998	879.5068	46.821194	7968.1577	116.33221	44.390186
DD84	3030.7156	63.172623	92.6337	18.053068	5.6896496	7916.3276	25.954254	1.1293625
DD85	2866.627	68.67192	90.166725	53.538692	5.114552	9082.921	26.908037	7.293314
DD86	133.1387	54.381996	62.71705	3.695918	10.690902	8975.387	5.4008665	1.061943
DD83	2956.71	67.548904	100.54362	6.068901	3.8520057	6493.67	25.128677	2.247819
DD41	1281.2549	25.311285	59.91668	8.36705	1.7822287	7219.0015	26.79319	0.7679682
DD42	418.15588	24.698414	66.6356	36.76202	6.109232	6786.3545	13.06152	3.2428749
DD43	461.42245	5.3370314	91.80723	12.465803	1.103334	6685.4697	13.654797	5.069824
DD44	606.41254	11.788997	79.83797	105.81406	20.295576	5912.292	9.658681	8.316967
DD87	575.0219	24.142733	63.46933	57.15525	12.732496	6116.4834	15.290377	1.9517556
DD2	2160.315	38.19765	59.107983	6.1992426	7.159935	1478.4156	45.493496	3.4281518
DD3	656.3204	29.216068	54.4115	225.86038	50.60109	4556.3706	19.84287	1.3273419
DD89	811.7991	14.518623	90.762085	11.48297	4.49117	3395.785	12.694552	3.9614587
DD90	63.701622	8.472398	61.036194	330.43033	33.5044	2718.6025	11.854123	1.1975491
DD4	309.20093	9.475903	55.50811	44.297367	8.200336	1275.6127	4.0085297	6.204731
DD47	132.12982	58.474796	152.33676	1532.9408	56.451324	5542.5103	21.984978	2.7534947
DD48	2515.1396	50.187	110.97604	3430.3691	240.88661	1881.8728	90.85296	31.652782
DD49	476.89505	23.549845	128.45491	1094.9567	53.873745	1457.4031	19.739893	2.881838
DD50	218.42577	34.86032	125.31497	1113.2286	112.08653	724.9611	13.565015	8.723138
DD46	839.9239	22.089062	54.99895	1619.1061	36.33084	908.45435	38.894424	18.93087
DD6	1760.3013	21.939007	83.60875	67.05075	9.964749	218.90099	51.595665	2.3086843
DD7	539.18726	6.99315	80.81919	14.101308	4.7966743	231.25177	19.228882	8.686037
DD8	13.636331	14.954344	81.35742	9.9517	3.0955102	765.2574	6.002737	5.727024
DD5	97.15352	22.875101	89.49	16.385855	6.816582	514.2591	8.088406	1.1610025
DD9	34.80761	14.349401	66.429634	6.605356	4.6585574	989.0574	0.5469406	1.3675896
DD103	1022.8501	38.74645	319.40195	616.5782	11.113417	6849.1396	15.70806	24.368341
DD101	2647.4355	41.796825	187.4813	30.641663	10.89765	7592.726	20.080475	23.968658
DD102	1077.5435	50.19894	369.59506	6.390294	0.5827599	7031.476	35.882587	48.74219
DD105	2559.2803	67.70931	445.40024	17.703789	5.2376447	6988.5728	22.90529	21.866789
DD106	2193.6938	61.893196	210.75484	13.710007	0.8466334	6631.5083	49.65688	32.056435
DD62	2581.4727	37.1572	204.18831	9.334879	1.262038	6704.1914	9.367047	33.508614
DD64	1109.4069	44.68044	248.14586	25.890253	3.5267344	5907.9316	21.280746	46.03697
DD107	340.34048	39.95361	349.794	12.575474	1.0655183	6376.717	8.491375	54.279533
DD108	486.63425	65.903786	392.63672	21.390718	4.4555397	5572.114	5.280445	46.04173
DD63	473.66943	39.385796	394.12033	16.734426	2.1601589	6164.193	0.8452691	58.36633
DD23	112.55531	36.681725	360.52216	5.5034285	2.7380204	5619.542	3.5791068	62.40032
DD24	295.79193	20.568583	460.94376	8.08071	0.2616001	6026.781	5.691949	70.44128
DD110	835.1425	41.506145	442.704	6.5973144	0.4871943	5765.318	10.600788	58.62017
DD22	602.45087	37.210197	425.68854	2.601716	7.4326816	6491.3906	5.7160974	56.143185
DD109	132.34473	43.542797	223.25748	12.460013	3.8958278	5448.5674	1.9722207	62.68194
DD65	150.4509	26.90732	219.6785	8.2505455	1.4423615	6185.0635	1.7493321	72.453186
DD66	46.728455	31.675362	231.0926	2.1956737	0.3714162	7626.1187	8.225806	71.67949
DD67	261.53342	21.719019	117.0992	5209.7046	327.7015	6087.7583	182.0273	157.52704
DD69	57.964912	33.711212	318.7673	85.82433	17.135664	5772.3916	3.713324	62.09329
DD68	125.98467	16.24219	136.34892	781.42365	55.743484	6365.556	14.639955	67.55
DD25	3.5917554	46.832542	222.9073	8.44316	0.3369355	4647.65	0.6039074	47.433086
DD28	23.215511	45.61319	182.49153	9.7158985	5.665282	6188.0684	4.2201324	53.617004
DD29	22.961355	55.001495	282.2609	44.44497	1.8125196	5987.331	1.0690632	70.46298
DD27	623.7014	33.95777	268.8827	11.289537	2.5325968	4857.3555	3.02278	47.748875
DD30	3.99313	27.451307	259.30252	11.690221	2.1091554	4354.674	0.3326045	59.089333

On-Line Supplementary Table 1

Animal	1370382_at	1370394_at	1370412_at	1370428_x_	1370429_at	1370463_x_	1370597_at	1370609_a_
DD81	48.50945	20.084778	1848.3267	16.743153	16.959581	467.48932	12.210869	302.68082
DD84	31.063896	22.148945	2086.1047	9.815463	1.9920418	418.77713	4.624875	309.7135
DD85	20.12734	23.391624	1460.5088	27.092999	13.964264	403.01758	13.296743	291.96
DD86	23.719833	13.442079	98.17018	16.694136	23.698418	579.9568	11.613841	485.39783
DD83	22.436806	21.224485	1820.2914	13.76208	11.986256	424.35236	11.527961	299.94482
DD41	24.52214	26.600908	348.7774	16.26315	28.964523	719.4981	10.727967	382.81586
DD42	15.822071	9.87286	107.15132	14.139582	27.743332	949.50397	15.209695	294.7954
DD43	253.43787	2972.0747	104.62684	27.762098	45.822243	981.856	18.925365	312.03824
DD44	54.20978	812.1222	94.12559	21.650024	43.45699	899.1339	15.479937	311.38928
DD87	31.507841	122.2309	98.2054	18.236317	14.572597	889.6256	8.793986	336.5312
DD2	44.24658	5696.8994	834.8418	16.680437	30.369303	1175.1466	11.909413	214.75305
DD3	54.29561	84.51005	189.1615	29.295958	29.182743	1093.987	8.065205	277.59366
DD89	35.180645	35.29181	124.68815	13.935405	22.63809	1332.4376	8.971086	429.34952
DD90	62.79836	36.216908	18.798903	14.941113	23.317532	1274.1405	14.933065	377.76724
DD4	57.748066	39.635925	63.419895	14.136256	28.82263	1269.816	21.732273	436.38562
DD47	28.76477	35.3083	70.13546	21.132984	23.985456	989.1995	3.2639446	240.79056
DD48	31.908537	36.252625	1434.8002	11.347019	23.89835	823.9809	13.57444	254.48988
DD49	68.117714	49.93822	131.686	11.294414	38.17439	1158.4276	3.6091447	266.1582
DD50	31.107462	47.76873	102.63063	12.631814	44.161278	1147.8724	15.630357	292.16754
DD46	85.69963	53.819798	181.73053	28.702671	29.809563	1089.2789	12.5392	351.37643
DD6	40.66596	3248.897	607.66693	22.52731	23.423763	1102.62	12.078197	371.7205
DD7	76.31896	150.31284	87.08107	22.518675	35.409386	1250.172	14.782926	372.7484
DD8	20.247915	46.70187	5.9549937	20.116104	30.878176	1170.7544	16.861588	562.657
DD5	17.542513	147.74393	36.681328	27.665741	36.031944	1122.8162	11.220253	379.2379
DD9	46.73708	129.6855	25.809963	15.185463	46.008003	1000.9405	6.7077365	385.75693
DD103	193.4879	11.701475	768.80334	708.85803	96.32626	54.634636	47.88786	114.49816
DD101	241.18893	3.2033296	1982.9904	8.0882845	20.217028	62.35475	32.521385	78.893394
DD102	98.15542	18.639402	1013.6354	1964.9077	142.95184	42.58712	49.096527	119.71568
DD105	58.03718	17.47147	1942.3566	372.14606	40.397182	20.624107	33.78707	93.36707
DD106	365.3462	18.50818	2242.5505	7.135829	32.261993	88.25794	25.110903	118.73089
DD62	192.45697	59.322563	1032.2601	687.3717	89.74935	191.39735	36.98446	108.40681
DD64	543.595	31.392532	256.6187	15.287657	63.264523	286.11545	39.391556	166.62901
DD107	736.45856	6675.7246	45.326393	1358.3389	276.86697	323.4862	20.040955	175.65837
DD108	399.85513	3702.9807	288.61975	1443.7523	191.70369	89.52705	45.005974	144.1155
DD63	377.26523	3862.2185	245.34738	1450.2244	225.31435	95.23497	44.351295	124.07069
DD23	435.47815	2120.9727	42.754135	1333.0781	206.19795	50.977028	23.32311	184.40225
DD24	270.37222	178.82182	109.67049	1037.499	136.0197	293.81726	37.69518	246.50397
DD110	259.66592	106.37434	23.683556	1432.1957	156.9889	374.34552	22.658407	160.70863
DD22	241.87807	120.53986	140.22098	1278.8257	141.56572	35.325867	22.501303	247.81027
DD109	246.57698	193.04323	5.4054356	1426.8347	149.21855	60.317715	27.756186	215.78696
DD65	459.3495	51.13441	33.41995	17.58134	66.10283	474.01752	23.816711	184.9039
DD66	540.827	74.57471	42.212746	7.0099974	36.523705	267.80563	28.979662	175.82913
DD67	213.66423	21.112408	61.281895	1058.2932	102.78127	257.63998	32.098602	273.4355
DD69	332.31198	278.912	36.36616	1553.0901	199.52266	416.91202	26.565357	193.26646
DD68	360.49347	1531.4114	29.103489	1442.0817	147.37181	384.76175	27.725664	176.6138
DD25	352.60678	320.64676	1.4958036	1546.9568	235.88211	42.168316	14.89757	224.31792
DD28	525.0432	57.36779	31.163784	12.878519	64.06586	279.9947	15.565102	175.64648
DD29	282.68552	67.87471	16.303986	1288.7568	141.07906	256.30447	30.492447	215.75687
DD27	587.44104	6454.569	33.45117	7.382291	65.744064	47.222443	34.674786	74.73067
DD30	296.23526	346.40045	15.810494	1488.1924	235.10051	46.93778	11.572634	229.52097

On-Line Supplementary Table 1

Animal	1370629_at	1370738_a_	1370781_a_	1370822_at	1370854_at	1370863_at	1370870_at	1370902_at
DD81	10.893495	1122.2053	2.8040156	129.62274	2098.0535	27.140669	5219.0767	18.916018
DD84	0.7390991	894.3128	1.4371092	133.76859	1878.8025	24.131899	5591.705	15.5944
DD85	2.1190057	1025.4541	1.0574682	101.3535	1636.3376	35.776264	5419.595	27.349045
DD86	1.2220194	28.230524	1.8017095	99.23092	126.84165	30.013943	6556.888	32.47691
DD83	8.216229	756.1398	1.4895102	74.07367	1684.0337	26.107197	4589.2876	31.78547
DD41	11.625828	268.80194	3.116123	104.94787	681.6783	29.76558	4763.1665	76.38475
DD42	6.508207	69.44935	3.603395	95.752045	225.41183	24.653328	4933.645	77.80708
DD43	38.52701	116.05025	0.4949392	186.5829	227.9801	24.743246	4380.881	23.864267
DD44	40.878773	120.71024	8.372205	188.89246	356.92822	39.37055	4470.3203	25.388828
DD87	2.0007777	110.99273	1.3379539	84.565865	324.90482	14.339241	4459.976	71.5954
DD2	27.395689	422.23303	7.39555	108.95634	1105.7487	27.200327	977.0003	40.36668
DD3	2.4853506	121.20126	9.39077	108.43295	299.85513	21.783203	1114.7705	30.966047
DD89	3.9525604	159.03084	14.076748	102.91917	459.43393	17.179222	1652.1132	9.947779
DD90	7.8670335	12.976526	9.605488	84.53587	130.55548	98.27231	1099.8445	23.68869
DD4	5.917551	64.98731	8.549984	77.63082	225.10422	23.986849	984.74695	21.822489
DD47	4.2283926	17.093378	24.525013	64.73942	80.52223	14.257415	1523.2869	55.275093
DD48	6.9387646	787.26294	18.884602	77.70393	1400.933	37.245472	1112.1317	45.995388
DD49	3.6607065	110.29119	27.236155	117.92144	243.55243	16.107653	568.6154	19.578018
DD50	1.1405805	29.225256	25.28761	123.44247	156.91234	98.10891	469.6766	10.359087
DD46	3.6945894	140.28539	34.86205	123.30296	421.61896	18.356243	674.54175	11.992645
DD6	25.452559	430.86838	32.19168	119.85777	943.61365	22.99392	734.8189	26.789692
DD7	10.182939	138.52235	24.659857	138.97838	270.22665	20.773998	698.88306	29.665508
DD8	0.7847412	4.2702837	34.55703	126.46056	95.58518	30.164722	764.732	18.942373
DD5	6.872251	26.336432	36.34812	139.05429	121.64034	34.083344	626.6286	22.252014
DD9	8.469616	6.7403502	39.052753	114.22398	102.00137	31.809906	624.0665	9.958897
DD103	22.334166	232.6976	9.166515	314.5549	605.3185	17.58517	5167.795	367.92065
DD101	7.9722414	770.5618	1.2062786	299.97394	1494.0284	11.972656	5292.5195	289.26138
DD102	42.903515	268.14597	0.7759189	156.7688	656.5105	19.268877	4975.3843	307.05762
DD105	8.775256	788.8741	5.6626186	100.66586	1621.618	17.464104	4781.6255	312.90857
DD106	17.047108	816.29803	10.785234	373.43137	1658.7728	19.526443	4522.9634	148.6103
DD62	5.367144	794.6549	10.406978	416.96347	1167.362	54.071976	3600.7603	384.2959
DD64	32.84375	230.11652	29.63346	1132.6405	468.41016	74.732735	2987.4868	346.87436
DD107	140.92317	88.69976	10.148503	1729.8248	161.39929	68.32975	2155.3071	213.50053
DD108	40.41803	106.17534	8.686012	794.6246	280.38138	198.6073	3916.4182	361.52695
DD63	82.92268	124.29841	3.1967373	812.35333	274.54163	220.75073	4115.0938	396.80975
DD23	23.123383	14.960482	24.53619	954.0888	99.39874	48.794506	3427.774	345.10965
DD24	5.8186827	69.16967	15.766013	637.0555	163.89418	50.448376	3662.3296	491.1512
DD110	6.453658	202.20755	27.691095	506.10266	385.81277	118.25389	2189.178	314.84784
DD22	1.9463375	95.79179	48.944096	635.8537	250.32036	69.59152	3085.4521	413.8437
DD109	3.2672322	27.588226	28.922546	527.63245	98.291046	33.277775	2576.6106	501.6573
DD65	11.054948	32.312668	34.35246	827.3554	128.63441	136.46754	1849.3934	382.18307
DD66	6.1029625	11.809084	39.015915	1228.4154	69.63194	23.031624	2887.0369	1716.2861
DD67	5.52361	52.48733	109.31178	368.11304	180.2512	18.017282	3888.5063	362.1153
DD69	14.288044	6.509701	80.05945	610.07355	103.83624	148.50078	1609.1018	372.47598
DD68	16.965645	18.233156	72.06389	794.2299	81.52509	23.830183	1629.4846	446.4691
DD25	7.5946407	0.388128	40.756413	628.3759	86.88811	21.353714	1276.8895	1243.7106
DD28	8.949905	3.24605	50.511402	1056.7246	118.39351	259.90018	1213.5448	248.04935
DD29	3.7456458	1.2402285	79.17563	478.46658	92.99242	307.58374	2309.506	1174.7759
DD27	86.667564	112.17571	57.26472	1845.26	155.88434	24.388329	1346.6583	1239.4956
DD30	12.218012	0.597731	56.96239	601.43744	88.08387	24.541107	1365.8896	1363.8325

On-Line Supplementary Table 1

Animal	1370967_at	1370971_at	1370989_at	1371033_at	1371054_at	1371119_at	1371124_a_	1371171_at
DD81	438.55765	6249.428	19.516125	9.776132	1.1867882	139.34279	63.916035	18.362747
DD84	453.1628	7432.674	38.13974	14.408937	0.8608792	116.27487	0.6789466	18.848982
DD85	35.04629	7126.229	46.999382	22.61429	0.8298994	102.34831	13.517091	12.055035
DD86	18.944931	681.56616	36.585724	13.852654	12.945113	154.09488	1.5841206	8.024973
DD83	102.10557	5755.8857	36.011883	21.082172	10.424847	117.21207	1.6103127	9.685258
DD41	83.19644	3690.0503	1.3018882	15.371787	209.58809	142.10806	3.1376908	22.644766
DD42	35.982216	1475.2891	1.0569079	16.42952	350.44443	169.99352	1.9384382	24.608675
DD43	4644.177	1571.7529	22.71851	22.550058	0.8040177	270.33063	4.69143	29.31519
DD44	3006.9949	1705.0186	27.19903	13.957618	1.9977183	207.90881	4.3159227	13.943596
DD87	159.67691	1585.8765	14.538635	6.3227997	211.76013	166.03325	1.3772947	22.295437
DD2	4066.3794	5666.1064	27.19325	11.210767	17.49325	117.49654	2.9526024	13.425478
DD3	96.065445	2352.6724	2.1566079	7.8820376	1.1036717	226.87956	5.481616	12.865294
DD89	95.42124	1712.3525	18.199316	11.18434	0.5277543	137.40707	4.8336687	18.725174
DD90	61.627888	130.26364	15.682428	18.908232	0.589039	187.39706	0.9974119	15.700363
DD4	70.65117	826.2707	5.5552664	17.111906	4.553516	279.02515	5.2170014	21.675545
DD47	65.81597	573.3554	2.0772238	13.302577	349.6655	84.72457	3.9871435	20.226643
DD48	49.032383	6657.912	18.25205	9.741941	65.59569	198.3551	32.43926	29.771946
DD49	163.42004	1711.4104	9.025622	14.190428	5.2557597	136.5935	4.3921237	18.635387
DD50	93.530655	1279.8716	2.0726397	2.925271	2.3561058	99.53888	8.258393	16.227543
DD46	70.680374	3284.5654	14.337132	9.888591	1.776323	276.21548	14.83712	15.858001
DD6	2128.3772	3204.6482	15.425773	9.236704	4.873591	163.85735	1.4643203	28.598852
DD7	405.99548	1334.9066	21.058966	10.410955	1.1292243	139.43849	1.7522192	3.919572
DD8	64.789665	29.156809	4.423007	11.078829	0.6615814	211.12604	1.629482	18.266634
DD5	125.94498	344.1506	15.664622	21.016302	3.2954812	199.37747	1.3706278	19.043
DD9	273.82385	87.6226	13.414444	5.141088	1.258472	185.82024	2.3238902	16.57821
DD103	255.41267	3108.0469	62.36729	575.6041	0.7359806	98.98845	5.8852487	64.76564
DD101	138.67616	7193.2256	55.780632	679.9707	0.7720554	40.285503	13.78683	41.135857
DD102	950.328	4554.131	80.19032	38.745834	9.81205	8.102903	14.44298	39.03199
DD105	65.88321	6961.78	76.38007	163.02757	0.6822079	52.908684	5.9178042	52.820827
DD106	549.0735	6475.11	41.386784	743.7421	2.0131412	35.135784	11.934649	66.68904
DD62	194.28653	5207.6724	41.852924	525.8701	6.290233	28.440226	4.2947536	52.675415
DD64	869.7974	2011.2301	47.56076	1705.3115	0.698786	124.59491	4.53301	64.46828
DD107	5726.584	381.91583	26.45865	1900.2306	1.246471	79.574844	1.4195155	78.44866
DD108	3021.3489	2057.148	53.236725	1277.9102	2.7117517	105.68664	0.5126896	52.58923
DD63	3169.7078	2040.0692	55.566208	1262.498	2.3594916	101.28503	0.5322998	61.48101
DD23	855.12537	294.3332	26.41728	1271.253	0.8511629	135.52791	5.1000834	69.96317
DD24	144.65007	914.6653	23.048498	918.85583	1.9418039	51.365078	4.8136744	75.12804
DD110	105.3211	958.57104	24.758665	699.37384	4.349396	41.932083	1.3689789	65.82558
DD22	62.465584	1020.9838	15.04472	984.0562	1.8190919	99.762764	0.8232422	54.29772
DD109	81.97191	159.18683	15.173286	900.9224	3.982585	110.87626	0.3613713	47.402157
DD65	177.1402	292.29822	35.11886	1697.8945	1.2239685	51.680943	10.97081	67.27993
DD66	127.88954	323.64243	11.527375	2120.6672	212.29942	104.81053	5.951581	70.070946
DD67	47.606556	965.79376	11.13593	564.8301	5.0415883	28.36274	63.563168	40.422413
DD69	348.25507	62.6914	27.620708	1069.4574	4.065397	58.030815	7.70463	40.99887
DD68	935.0137	744.19806	18.513748	1282.8025	3.6990447	53.91656	5.4740796	61.466694
DD25	270.23755	8.449969	19.11129	883.51086	223.50931	106.94559	0.109876	62.21974
DD28	62.666187	74.85728	18.274197	1750.581	0.9545595	71.88677	8.125784	61.085728
DD29	79.53543	16.548073	18.525955	849.70087	90.82778	17.791912	0.3862593	67.04983
DD27	5382.636	583.04626	38.538902	2870.2131	117.79846	190.9096	6.375128	80.522934
DD30	292.7747	5.508217	12.56453	911.02155	265.8335	104.80156	0.1103111	58.323006

On-Line Supplementary Table 1

Animal	1371209_at	1371213_at	1371248_at	1371262_at	1371339_at	1371354_at	1371442_at	1371530_at
DD81	25.017553	186.53302	37.426888	219.75021	2071.7346	2611.6418	194.54903	69.31756
DD84	15.855091	169.59892	28.545502	279.1262	2107.0972	2575.2769	178.72287	52.490128
DD85	21.49835	157.5996	28.136255	68.30299	1781.6056	2344.9954	183.71367	65.508514
DD86	5.444637	216.75647	21.32296	39.170063	118.70053	155.09468	230.36389	87.39996
DD83	14.486709	170.18227	28.61243	97.2267	1847.4784	2298.372	190.62369	59.93098
DD41	43.37147	127.11889	27.348808	62.85843	879.25946	1014.7264	178.03186	56.34559
DD42	78.647804	166.40051	20.876087	48.682434	251.97678	345.20685	182.97404	59.14892
DD43	234.96657	250.8272	22.159431	5915.5073	138.2089	215.58638	174.75716	72.331764
DD44	163.91931	213.95663	72.49401	3908.5889	161.97328	227.67928	185.9341	96.63039
DD87	51.82958	193.9673	7.630279	135.75702	215.34041	293.43726	207.7484	66.86073
DD2	172.66316	196.78185	25.173018	2728.8281	1469.0671	1988.9928	136.01093	46.886127
DD3	106.79643	245.58827	18.480013	57.78765	290.79388	463.30447	201.80104	66.027054
DD89	61.516827	187.91805	22.242115	54.7726	306.18842	371.38843	123.13292	60.872616
DD90	84.66732	153.21672	507.72003	68.036156	19.251581	46.202248	86.652794	167.14624
DD4	96.24089	159.40874	26.948925	65.27564	106.88138	202.51839	153.31317	56.031734
DD47	97.75551	189.44951	6.810877	41.160004	76.681885	171.82672	348.65457	75.15559
DD48	72.210846	173.75793	14.662756	77.30189	1409.0548	2192.636	247.42107	65.216354
DD49	109.56717	266.48248	21.150904	352.72308	122.09985	303.92978	295.74527	69.24982
DD50	115.49177	243.64742	121.18378	32.798912	109.9282	200.23521	314.2093	114.56162
DD46	143.51189	161.77731	23.733204	57.36474	310.13226	558.3085	149.74716	61.844593
DD6	111.03405	154.3911	16.675207	2330.6963	1034.8673	1341.4529	149.29204	47.32407
DD7	131.12779	171.10791	11.603737	231.8435	211.72768	293.13602	115.241	54.66622
DD8	87.83624	169.92216	20.802923	58.600773	29.0626	44.595398	156.79076	40.141968
DD5	100.16525	183.25041	33.39553	90.11473	66.9971	94.90815	220.27357	41.664944
DD9	120.46495	192.26845	20.51815	94.49068	22.91178	63.166737	233.42493	53.745815
DD103	207.91277	64.02813	10.220638	155.28189	904.13403	1203.9047	178.20628	84.10524
DD101	42.09906	33.926975	10.71894	74.402954	2425.986	3011.2744	79.09043	61.54082
DD102	125.33209	25.936409	25.090952	566.012	998.1363	1238.4459	247.3325	64.20992
DD105	44.46917	50.33987	32.43456	59.747253	2327.1553	2655.2793	71.10775	42.935307
DD106	54.122295	47.835396	16.098682	109.57983	2326.3813	2933.6943	72.444916	56.46487
DD62	40.39194	39.29129	138.6919	520.0428	1895.0239	1849.3347	122.99994	75.77596
DD64	351.05954	62.21328	342.46982	507.2064	394.4467	491.34662	141.17885	217.1589
DD107	185.0199	59.158142	157.09886	6137.639	112.36048	102.90606	66.17055	131.46576
DD108	328.13092	99.134865	576.4467	3694.1255	328.49704	451.9451	183.82393	271.51868
DD63	344.5071	80.8099	570.49036	3700.4683	321.53906	472.1979	153.49849	291.64755
DD23	485.59528	72.72919	159.39774	997.41	26.459478	59.585022	100.18873	117.29515
DD24	66.182816	50.70278	102.46203	95.46882	220.6325	319.63678	99.66834	135.48424
DD110	53.772026	56.911045	517.3623	80.468094	24.509888	45.688114	107.27818	241.68784
DD22	284.24948	54.981117	357.26263	106.11288	270.76273	412.82318	106.89764	238.2966
DD109	301.33948	65.26726	132.72372	109.0295	21.957941	32.853786	184.87631	164.25752
DD65	0.2578805	37.389244	594.41473	82.78262	31.530216	45.237164	153.55504	324.30817
DD66	387.73917	60.499023	20.953436	114.02477	56.234314	73.38782	89.521774	111.98794
DD67	40.559128	53.44023	18.086948	53.071262	83.72116	175.84071	84.47323	105.29195
DD69	98.191696	61.089138	698.31085	183.58423	35.61967	59.528576	79.05141	372.6885
DD68	88.96056	54.39357	19.680353	1959.7118	28.168318	75.15037	89.77734	85.241936
DD25	470.9301	61.429707	29.576778	465.74704	20.02076	29.974731	68.19246	97.898834
DD28	169.34485	58.541866	709.01135	85.38319	27.763687	42.871994	181.06642	357.1986
DD29	74.123215	20.389864	832.58417	93.65612	1.8501462	27.444124	86.780685	368.16394
DD27	1053.0404	99.801735	15.177546	5806.074	19.159016	50.537575	83.5965	59.90553
DD30	510.3212	48.154053	35.27734	518.41473	4.898352	39.59687	56.132923	93.23381

On-Line Supplementary Table 1

Animal	1371554_at	1371677_at	1371776_at	1371801_at	1371960_at	1372153_at	1372190_at	1372239_at
DD81	2470.2607	4.8732057	1162.8027	2401.0269	124.94414	28.964016	485.45795	116.45227
DD84	2542.9075	24.361818	999.2827	2593.1006	112.01716	28.071901	563.64087	139.31584
DD85	2414.075	20.372322	1020.9076	2287.9932	117.25462	25.843315	464.05008	141.11716
DD86	103.47188	54.425694	1469.7561	127.16547	100.47423	8.19652	10.064653	158.85173
DD83	2546.1047	43.265205	975.2665	2146.2405	105.94761	5.938563	486.17233	144.88002
DD41	1786.7286	20.61936	1587.6388	833.4941	119.95589	22.758366	77.98093	140.40941
DD42	724.7625	23.56017	1208.4292	269.21866	119.77213	15.171653	28.649616	115.53698
DD43	583.2696	16.830872	1329.6681	169.59871	149.60257	18.302916	23.486082	119.1666
DD44	789.4632	25.94785	1381.6427	161.8842	136.11255	32.072933	44.200474	107.04248
DD87	858.3139	16.315037	1271.7963	228.77037	118.38142	7.548692	34.928654	150.99998
DD2	4140.4067	13.447536	1430.7301	1946.7755	117.11467	7.5866256	258.01633	134.61246
DD3	1404.3535	24.624146	1822.3231	400.99725	112.49864	12.838554	50.815437	187.8926
DD89	1202.5253	13.724128	1797.7745	445.61145	169.9228	11.590861	45.893845	178.15718
DD90	92.35054	28.247473	2386.5327	24.917986	227.90984	79.43059	4.419869	185.85658
DD4	681.205	18.160837	1737.0809	189.49529	157.85294	3.5393896	46.90037	153.16081
DD47	507.1456	12.304512	1489.2317	88.735825	99.1912	18.95951	9.752003	171.29958
DD48	4340.211	17.289993	1415.8334	1797.8481	117.71529	18.115046	392.6909	152.00116
DD49	954.73065	19.826834	1916.7494	167.52206	114.07552	21.665443	27.259138	183.76387
DD50	621.70844	4.326711	1775.0468	123.69267	105.17155	22.849964	18.095142	172.81604
DD46	1972.3455	10.278322	1635.6598	377.176	114.89446	18.41397	75.49969	144.91025
DD6	3596.814	10.564259	1791.7063	1281.3485	150.55354	13.420407	214.56396	149.63434
DD7	1336.9005	18.950523	1924.4304	312.27515	221.41159	21.25045	58.539276	178.33504
DD8	51.974396	22.02782	2010.9641	1.2564715	190.57848	12.258883	1.3421369	158.15858
DD5	255.25195	3.5086331	1455.4264	59.080017	140.07266	27.700436	10.987355	147.73416
DD9	85.30439	12.38196	1365.8405	10.50132	107.53252	4.6289563	0.2654501	157.27704
DD103	601.84973	32.79864	34.06071	1040.3705	718.33655	16.707693	160.61145	70.129295
DD101	1612.9218	16.703663	32.455162	2539.9014	696.9772	15.056333	595.5729	69.41578
DD102	699.39874	31.491486	48.013554	1264.3218	672.0296	28.402847	141.96574	54.673336
DD105	1604.3589	28.208504	58.131966	2620.8508	600.23804	8.225363	299.0868	58.55783
DD106	1681.7432	21.903477	48.430157	2436.1992	736.20245	21.622963	579.7586	72.89496
DD62	2547.009	20.577095	65.22209	1664.6674	613.03217	39.595684	344.9527	65.43047
DD64	1051.369	39.982067	97.9597	344.2033	592.0071	100.00259	122.85225	43.014236
DD107	328.11755	32.52284	91.923225	59.913162	643.7966	60.34687	20.572609	44.692455
DD108	427.919	39.884052	47.75752	264.24313	614.5562	149.72612	17.770767	58.600636
DD63	443.0454	55.947273	59.67763	245.0255	606.34143	157.06677	16.181688	58.229675
DD23	210.96104	42.46603	77.66593	13.382362	460.6696	40.971756	10.083525	52.671852
DD24	563.4596	33.758938	90.65479	150.88301	544.6724	66.496185	15.088896	59.032055
DD110	707.56226	38.40732	98.93583	1.5910212	491.7897	172.05963	21.242258	54.0191
DD22	1130.6803	32.990128	79.88158	191.59288	444.26132	82.73021	36.186653	67.02299
DD109	163.83331	33.26969	64.95174	0.3436813	464.87402	34.10402	5.7759714	60.55286
DD65	217.74783	38.25837	94.75179	0.6461473	540.6979	161.52855	14.774326	59.237938
DD66	122.87679	34.62926	55.790962	25.459837	660.8069	26.240644	4.573941	50.852398
DD67	806.618	36.40676	67.34019	67.635475	676.3315	3.58974	26.98671	50.472294
DD69	129.01596	31.312315	73.11754	9.060476	664.93335	253.9057	1.2525389	57.028618
DD68	250.9526	25.9338	71.938	12.106891	663.22626	25.170488	3.7232585	62.587933
DD25	5.8778486	25.159634	62.694324	0.3662078	463.92398	19.982582	6.0856247	66.1057
DD28	56.884907	56.16209	74.55387	6.9470277	713.5382	274.2267	2.7090757	64.005936
DD29	44.943512	61.085682	87.98416	0.2866129	714.6706	381.1999	0.4778894	59.927364
DD27	756.8185	25.08821	61.02164	2.19603	837.45416	13.115448	36.538887	62.859295
DD30	5.0340915	31.277836	71.00741	0.448089	477.09787	12.549099	0.2157939	62.841133

On-Line Supplementary Table 1

Animal	1372297_at	1372318_at	1372345_at	1372423_at	1372481_at	1372595_at	1372641_at	1372745_at
DD81	208.97765	1816.783	77.957886	26.934534	510.9181	4129.251	993.93085	2368.5498
DD84	193.39629	2443.6218	53.25049	5.586771	585.47784	4079.392	966.3101	2586.2102
DD85	162.6321	1920.5979	48.541054	1.3212054	555.4971	3820.4382	916.1391	2288.7178
DD86	311.90704	3266.0583	77.7742	9.661264	747.86316	219.73755	67.26278	107.82749
DD83	171.42332	2136.5256	75.227196	4.24217	588.2138	3278.823	906.34894	2435.6643
DD41	155.75517	2046.4971	68.6948	2.445855	485.09885	901.43976	331.1682	1125.3325
DD42	140.86852	2364.2432	60.306263	11.152502	430.451	353.31708	94.151886	346.06726
DD43	148.21869	1810.506	66.54221	5.867588	448.7064	306.98053	133.70132	352.06107
DD44	132.56587	2130.728	61.513794	5.047074	467.005	424.36282	153.72812	467.0254
DD87	147.41841	2271.6562	87.11673	6.6552224	464.7625	454.79037	168.47327	398.01315
DD2	171.91406	105.68079	50.92652	15.859146	390.7743	1892.7236	988.32556	2071.0808
DD3	206.36487	271.65332	141.30865	15.130916	683.5909	478.4675	236.28842	458.0076
DD89	241.48991	389.4353	163.21364	9.847641	278.3625	421.43066	232.58975	510.9701
DD90	254.22208	406.24615	222.58951	61.190598	294.8321	26.07834	41.78305	43.4272
DD4	288.4999	251.65569	160.90265	13.631363	332.6229	158.1389	95.02252	229.20325
DD47	173.98755	176.56256	78.04839	23.02161	520.1063	122.18004	82.58468	141.57982
DD48	166.31721	79.667755	64.13887	10.657295	555.84534	2285.1594	1010.4141	2366.7856
DD49	234.29405	110.10671	128.00507	5.645709	636.211	235.81256	167.62674	359.54886
DD50	203.85216	92.02116	98.3074	26.74763	583.2615	188.87846	122.61935	170.61269
DD46	280.80087	94.256546	115.91303	9.779953	522.5328	413.2396	271.6766	767.4195
DD6	245.94778	49.143913	78.153595	7.253469	271.58417	760.3035	507.30872	1854.3502
DD7	274.00403	78.78291	139.17778	3.007286	259.46863	172.81606	140.1443	512.33704
DD8	282.74692	101.49258	125.71697	5.23927	256.99506	7.0863605	29.578259	18.133017
DD5	318.30402	115.71092	115.58822	11.964946	330.6869	32.93927	65.87388	99.08359
DD9	228.8708	86.2423	131.63849	14.008607	338.0006	22.006876	48.48445	30.002686
DD103	582.68994	3569.146	27.98422	8.4749975	902.37854	1554.3677	229.0553	1077.2003
DD101	407.54156	2672.8308	18.686352	2.3903124	1120.5061	4003.7886	705.5926	2752.259
DD102	394.06686	2622.8794	40.35812	1.4974148	1004.8087	2174.8594	269.21036	1232.4221
DD105	432.46423	3001.303	32.626083	3.4649024	1102.0754	4064.9475	829.13104	2487.2227
DD106	472.37842	2214.4644	28.429085	13.775414	985.2233	3398.6406	716.99744	2670.908
DD62	319.46164	1201.1865	175.34422	25.412956	983.3002	2204.7617	794.32404	1937.0022
DD64	361.50845	1105.8629	158.96902	44.262238	770.66644	760.858	286.08102	717.9689
DD107	335.09213	691.02313	147.57239	33.194885	963.1177	170.62755	66.897575	213.80673
DD108	392.77872	1844.2054	103.59185	47.350765	1171.1472	493.42758	131.9587	361.49203
DD63	386.61752	1730.8346	100.15234	50.20703	1191.5602	508.38547	137.39418	329.25626
DD23	503.24222	1659.054	197.27097	55.928284	656.3182	56.896137	39.225132	79.04985
DD24	508.57288	1617.951	144.67697	29.896013	543.79346	236.7193	80.72746	238.49951
DD110	469.20834	695.6042	174.80533	46.64609	1210.265	196.5112	213.55031	507.96048
DD22	569.74835	1173.8385	309.5348	46.111275	607.0465	382.96597	162.89182	411.52054
DD109	480.67932	1138.5192	299.65063	24.76985	1248.7098	50.45795	45.731632	111.93753
DD65	530.96704	706.23535	353.2942	68.116196	884.4852	33.053032	39.060757	87.07801
DD66	486.3837	624.1531	105.58196	14.327079	784.5008	58.12166	39.350986	38.69805
DD67	687.6886	1742.3539	99.12379	24.116491	819.5565	210.4549	86.37848	189.06601
DD69	529.45667	603.8135	213.62825	80.22982	1196.0131	22.25148	26.050253	49.83752
DD68	480.07074	401.56082	420.9286	12.291686	889.86285	93.2964	30.820452	88.57913
DD25	550.1499	374.03287	391.4434	42.48223	973.0371	19.776459	42.92013	9.873307
DD28	543.2543	303.4863	198.92583	95.01152	1000.8817	4.784375	30.56007	25.128916
DD29	602.71924	757.8504	198.40392	138.40878	896.48	3.944975	32.515877	22.940191
DD27	364.43954	341.2191	273.30533	16.563337	508.87347	67.44618	106.36186	241.42526
DD30	602.64087	379.76965	399.98502	18.896454	906.02124	9.71502	33.90379	10.980368

On-Line Supplementary Table 1

Animal	1372958_at	1373108_at	1373582_at	1373684_at	1373873_at	1373889_at	1373900_at	1374006_at
DD81	33.300957	1205.7219	86.29167	2202.6294	858.8126	62.738518	20.771217	71.698814
DD84	29.555325	1517.9896	89.4471	2145.5784	715.3883	44.70951	5.907953	52.28657
DD85	41.01536	1397.2397	93.17911	1897.8656	785.3957	53.741974	31.162718	60.92188
DD86	40.27585	145.00381	123.34773	170.46605	80.16183	70.8896	12.234963	42.051342
DD83	38.269417	1403.2126	86.42306	2043.1936	705.5447	42.461964	16.650822	56.52365
DD41	25.334143	952.5407	63.55394	1172.7021	511.6652	116.93083	10.792039	62.51375
DD42	44.28561	396.53687	74.92414	472.66898	242.52557	102.09135	2.6739008	62.84768
DD43	31.031769	380.13956	78.80846	581.8025	232.90152	105.65438	7.846166	57.512478
DD44	30.76258	423.71783	72.005745	678.34753	296.51196	143.05736	43.1327	48.573704
DD87	35.593555	603.71735	87.46077	445.9168	180.7624	96.41609	5.2082524	50.335976
DD2	38.12194	2721.1184	76.322876	1350.3958	1722.696	133.60927	1.3874369	78.61121
DD3	33.6762	618.30994	109.83891	337.176	487.63312	155.27618	11.818351	100.4608
DD89	25.888433	667.77344	99.246346	496.7716	458.54437	186.31107	12.486628	81.5226
DD90	15.787748	106.63651	93.47968	130.86266	82.26716	128.64337	211.03708	82.4424
DD4	19.757395	220.05235	83.057335	307.09753	256.34933	147.80756	40.096996	88.65945
DD47	45.632423	243.5127	81.24467	107.78079	224.5355	152.96794	30.817589	97.33975
DD48	44.626175	2677.9868	87.29128	1412.9116	2447.3289	109.89747	6.798027	96.26813
DD49	51.49283	532.7565	107.1345	264.47366	328.44955	140.94676	24.772156	106.62535
DD50	59.08264	253.69212	129.20235	173.60477	235.59023	127.05154	65.838585	93.249916
DD46	27.639622	1009.0676	95.62626	459.611	521.3514	109.67412	2.0280557	103.42969
DD6	38.4251	2107.8987	70.45242	1152.0569	1056.3396	106.76441	7.829552	92.23843
DD7	30.944681	534.0159	76.67904	327.84457	326.05463	181.60596	11.633121	108.55826
DD8	27.645103	139.51163	78.57471	63.352337	36.752735	126.96018	2.4617593	122.42931
DD5	59.120117	192.64029	65.48102	217.43431	147.87877	165.94907	15.857978	136.44395
DD9	38.553017	114.85013	75.79317	83.473656	49.538006	183.54489	23.877268	117.42714
DD103	85.61009	356.99414	29.957144	660.0099	123.60745	163.84897	32.034504	133.21971
DD101	75.3928	1277.94	43.537266	1898.2491	571.8295	109.25127	20.64545	136.39618
DD102	79.21804	419.15836	41.02053	728.7246	193.13235	172.12555	33.19785	138.67117
DD105	62.21636	927.3417	43.161705	1831.7043	355.5421	102.89318	6.1756916	115.71391
DD106	63.82974	955.63055	42.167442	1910.5879	363.3361	89.94533	4.2519507	145.94838
DD62	41.843517	1612.288	25.703028	1661.1969	578.55237	312.29425	47.34683	137.95975
DD64	57.34146	812.72845	33.223568	825.3764	282.8174	381.1794	144.73515	202.36406
DD107	54.51385	234.38441	23.826496	244.75517	115.85853	393.76932	57.421165	188.59361
DD108	81.04028	244.80914	36.409588	313.01953	125.03008	347.90436	206.78984	127.34814
DD63	70.4136	233.99721	43.108303	312.3594	117.96527	363.88458	206.85345	115.58122
DD23	57.179317	214.64868	28.988256	116.41518	101.75513	593.62476	72.358315	181.75903
DD24	77.42914	355.12036	22.574022	289.59976	149.28267	618.94257	58.18195	188.77402
DD110	64.39395	774.1495	18.414656	636.3485	331.85913	470.2532	190.59883	237.47855
DD22	64.83668	800.724	24.581835	353.2166	338.92603	737.56683	125.29462	256.8361
DD109	70.170044	249.27734	48.385036	138.5095	113.15257	572.9774	64.773796	249.21327
DD65	72.873116	293.67178	35.255592	122.82622	131.36543	618.5502	241.2035	254.70882
DD66	105.48363	109.97059	38.3508	83.84089	69.403534	726.83044	21.158905	176.21532
DD67	87.125015	551.873	36.09067	207.96121	200.1865	420.4715	20.19456	361.76526
DD69	78.42148	147.0245	35.302307	61.196243	57.945484	569.2254	288.60413	276.67883
DD68	105.61389	215.69945	29.661667	134.3535	91.941185	598.97833	30.941442	250.41206
DD25	76.38811	154.69456	41.02875	80.64508	16.914059	510.53574	21.70371	277.84323
DD28	83.05897	135.98206	43.892784	54.96436	43.453857	618.9046	348.9699	263.41406
DD29	97.45684	110.16523	39.328575	85.75752	32.24479	569.6183	403.43967	175.23175
DD27	85.8927	501.7063	31.38192	258.36493	253.69861	389.83966	10.791205	202.13132
DD30	77.74235	155.60608	46.471394	44.03511	8.58689	566.7055	19.565956	294.4008

On-Line Supplementary Table 1

Animal	1374015_at	1374049_at	1374099_at	1374160_at	1374207_at	1374248_at	1374276_at	1374283_at
DD81	219.54367	781.6749	2274.7363	551.8128	122.44959	4253.88	1.9723289	12.146656
DD84	249.00992	920.4247	2048.3462	623.0391	118.44615	4115.6084	7.8029246	0.9185274
DD85	219.38904	731.8656	1970.5886	720.8097	122.81188	3853.5276	13.048782	11.462825
DD86	30.133541	17.204605	99.168945	978.822	149.88055	186.93874	26.20896	1.0468806
DD83	187.81119	733.3246	1711.2058	609.21405	110.00894	3457.6787	6.8848743	5.9150257
DD41	27.387342	194.22305	521.94025	706.88025	129.75104	1684.8564	12.82318	1.7071089
DD42	19.57739	42.613888	218.37518	813.8876	110.11712	706.38666	17.669838	9.710982
DD43	23.274986	16.826633	211.04729	728.87494	108.56615	597.94055	11.730178	17.93488
DD44	24.311348	29.933475	257.98846	355.0987	117.88213	679.06946	12.957383	2.92919
DD87	25.913437	40.091442	248.47076	874.94684	121.38417	728.6047	15.927112	8.412374
DD2	18.36818	267.30447	1316.1122	837.83325	167.27614	3031.8193	13.898413	9.159735
DD3	15.993862	43.115864	322.7867	807.7	169.5043	836.3583	19.520578	12.713948
DD89	24.199783	47.95095	375.06845	744.44714	166.88036	869.1521	20.628609	18.518446
DD90	26.294336	0.7239184	41.76684	907.14465	243.00111	65.708534	14.362155	1.4717296
DD4	23.557762	4.830126	145.59483	1894.9801	299.8109	424.59912	10.208026	1.7575189
DD47	18.363993	30.528982	101.88272	680.8659	171.97165	275.92294	22.945827	15.804173
DD48	22.576431	457.64194	1780.9064	528.2026	169.29907	3238.0586	22.59398	19.342867
DD49	18.068201	40.90674	215.9338	834.4461	219.36029	576.0362	15.23622	18.811436
DD50	22.321688	3.3549814	173.61148	1004.3505	242.18378	252.18881	24.415134	17.944418
DD46	15.860563	36.49658	365.22064	518.22015	234.5974	973.025	8.665051	1.6526337
DD6	15.91662	108.43634	716.828	517.8313	201.1273	1808.1243	15.807023	20.377377
DD7	21.435558	33.479088	232.54985	641.7396	274.12415	558.4734	24.206713	1.0031613
DD8	15.02693	0.5964441	21.032057	1094.8889	251.04703	5.0831475	15.00918	5.9985943
DD5	18.482927	4.030989	40.774807	1057.684	242.93526	115.78804	24.84811	1.7771848
DD9	19.96494	1.5646733	29.59735	1840.193	195.84747	7.7905235	19.451025	7.7779565
DD103	123.8866	409.06973	486.54965	206.21182	66.705444	1506.1865	43.163773	52.14931
DD101	405.43976	1143.0945	1206.2173	277.34317	73.8156	3786.4133	49.401283	34.943886
DD102	265.973	482.52487	603.766	181.97389	112.51731	1682.0453	37.59983	46.05552
DD105	305.76282	1179.233	1466.8899	264.5129	82.23555	3686.0796	31.113422	40.262806
DD106	396.40158	1105.6129	1301.5729	308.35114	88.22291	3058.1252	48.307312	42.455536
DD62	36.070652	429.01672	1401.8704	205.67438	88.63635	2995.5688	10.293092	8.209971
DD64	39.740746	74.37135	385.34418	196.28029	90.04891	1013.2922	58.09687	9.041293
DD107	40.004074	2.9718866	103.16416	173.96811	87.83307	371.2629	48.90028	15.927826
DD108	39.479416	108.58347	254.96428	148.32169	69.50105	584.74817	41.27227	33.795357
DD63	39.099304	86.6415	225.75565	149.00706	78.564415	658.2532	37.989693	20.676298
DD23	41.473827	2.0715487	52.501747	136.57103	91.86797	93.62627	58.24221	41.865128
DD24	51.74498	16.920317	106.41838	146.9416	93.92805	344.32193	128.71388	57.805588
DD110	41.562595	1.8948119	168.52696	156.12744	78.811844	452.91052	105.47985	49.578056
DD22	67.36272	28.373032	226.41339	140.8757	81.112976	708.6764	131.00229	49.58437
DD109	51.802	1.1489882	38.5222	98.315254	85.54455	82.52312	89.48051	99.380714
DD65	54.177784	1.7252609	32.20351	175.48834	88.15675	94.40271	99.00396	5.3596883
DD66	48.48237	2.4255857	31.624208	132.94223	90.49579	155.53465	62.654408	3.4062307
DD67	52.105713	2.7057307	87.959526	61.200726	68.50331	333.87903	46.06803	0.863142
DD69	50.685394	3.7976081	15.275773	123.04414	78.632675	24.761307	63.884937	9.7547865
DD68	61.77266	1.8553807	49.14314	106.9981	81.71657	136.87708	59.101368	10.716254
DD25	51.989452	2.8286397	6.252771	122.10974	94.45891	65.83563	41.72715	86.22268
DD28	49.555626	1.2401788	6.862174	135.92674	76.28413	5.616671	82.19191	10.542796
DD29	43.743263	3.9537559	8.630584	92.95951	76.03883	9.986455	78.89672	4.307807
DD27	52.464306	4.0505614	99.12777	105.21099	76.53987	270.32877	33.025658	18.66491
DD30	51.252815	3.107813	8.00173	98.69037	102.31418	90.59225	43.157276	77.77169

On-Line Supplementary Table 1

Animal	1374330_at	1374345_at	1374558_at	1374583_at	1374630_at	1374699_at	1374710_at	1374828_at
DD81	62.014004	14.798513	35.453243	54.815865	1.4312601	11.427988	1364.9783	186.96169
DD84	53.933052	14.149699	35.849335	53.718567	2.5476549	16.137936	1199.7445	134.97195
DD85	83.04645	18.294277	38.952805	58.943195	2.527412	10.489649	1209.1753	153.46594
DD86	104.05497	1.7184258	49.371387	44.466583	12.289653	6.265151	71.07995	182.77957
DD83	75.6369	9.313067	38.35354	48.738533	1.2246183	7.4960194	1087.6528	152.33302
DD41	135.72452	8.539203	16.807346	49.364952	3.4272776	19.384	332.22153	184.71829
DD42	126.79417	5.079962	19.550505	61.56027	19.244354	17.565653	127.50389	181.78545
DD43	124.0205	2.3938904	41.993317	50.014816	3.856374	10.546864	127.65042	176.00876
DD44	52.54973	30.362488	27.579416	54.078373	10.709765	16.928562	198.36519	179.98436
DD87	133.05614	5.7160892	37.809635	55.335293	11.276357	19.247402	129.45416	173.19424
DD2	72.42439	0.9941194	28.796434	59.514008	12.610839	1.8766891	639.11365	152.23259
DD3	105.21649	4.1112294	50.093357	47.693317	18.964485	9.780029	135.41275	146.81369
DD89	102.31748	3.1663568	26.401524	50.946186	24.547506	7.305706	168.11263	157.5212
DD90	119.79215	85.59758	37.974533	71.86312	20.150787	38.29249	59.51358	149.12581
DD4	324.57672	3.6969817	30.038273	56.653576	17.132296	11.547675	97.404366	153.80289
DD47	83.50619	3.2104294	66.02939	61.572495	27.780775	11.996659	59.84211	158.88664
DD48	45.728516	7.7048817	64.33445	37.91227	21.832077	8.395068	620.24896	146.4164
DD49	75.838554	14.174551	71.06764	59.403652	41.70171	5.9138002	129.94408	162.9514
DD50	105.91441	33.895145	67.05857	44.589783	23.434555	6.567242	93.44315	163.08179
DD46	47.834515	13.498164	28.80573	43.74462	13.590998	6.028925	197.70644	158.49295
DD6	59.53425	4.6536655	16.624704	58.8773	12.654212	8.7763	427.4208	127.21383
DD7	91.66324	1.9989526	37.023525	49.14672	19.669542	5.339221	146.96686	181.62314
DD8	94.652275	2.5004816	35.857464	44.001648	6.0968604	9.484369	72.51106	154.57867
DD5	111.2011	4.3978443	37.807354	57.884644	33.364727	2.037212	85.574974	183.83133
DD9	191.55283	11.799848	41.161926	63.41192	24.202	7.1557865	56.979893	159.91336
DD103	90.567856	13.445783	93.58149	74.50677	5.634697	16.791586	450.2865	39.65091
DD101	27.003105	5.8545704	116.40213	122.90465	15.086216	7.034481	1059.7754	117.22519
DD102	43.818542	2.9682205	139.83206	128.56284	12.348279	8.078379	472.9932	141.9118
DD105	71.4405	5.3904614	104.14667	47.28113	2.2542953	11.842615	1228.74	118.28786
DD106	59.28439	2.715009	102.08422	117.75702	2.0132022	6.3562274	1029.2755	32.490715
DD62	36.137512	44.523987	66.85234	132.70212	13.328992	17.398273	723.5222	101.78553
DD64	31.469667	73.678024	107.27123	102.27561	42.596397	38.249218	268.9538	134.47307
DD107	24.455393	51.436604	108.78912	144.63358	13.587	17.269192	94.6438	58.215057
DD108	41.09697	82.53597	132.76953	153.57996	22.69069	37.202885	126.38054	70.196075
DD63	35.660137	102.49323	125.63469	146.7946	39.02454	51.49476	125.5074	76.073395
DD23	29.175488	51.76698	180.0002	127.47192	21.1682	23.292534	54.546307	89.95272
DD24	27.372557	45.69522	140.89815	146.61337	31.69105	22.497206	76.72655	93.90712
DD110	29.0142	98.187775	141.96646	147.71481	44.01939	42.33319	164.26103	44.42209
DD22	20.076834	81.40368	153.57259	144.05124	56.11237	19.452816	109.45358	27.629845
DD109	21.12389	45.66255	178.44597	170.47314	45.981228	13.385836	55.175888	108.36476
DD65	17.935608	99.30744	143.13596	88.1576	30.637983	43.54941	56.391075	135.52676
DD66	38.26608	4.6189847	99.81498	191.58908	29.957657	23.288286	32.231766	35.53918
DD67	20.806807	11.853909	151.96024	96.5842	34.0159	15.149382	89.80265	28.9935
DD69	25.713606	97.92348	255.29266	124.61398	61.9715	48.147854	45.527485	185.26329
DD68	17.88004	3.128416	176.05756	204.33466	30.853004	10.859686	49.723297	54.824512
DD25	22.244158	19.465685	220.3699	185.6069	23.85725	7.4598875	46.171345	131.665
DD28	25.071737	87.95987	364.25638	181.1938	60.624	79.94995	48.34455	108.20802
DD29	25.716187	128.34947	318.97806	163.6438	57.613632	93.41427	30.40675	106.80039
DD27	13.355249	2.4445188	244.1194	105.09199	15.659719	2.9465334	104.82452	124.89235
DD30	24.443117	17.98576	207.466	226.33662	25.28976	7.3375463	41.699387	137.71266

On-Line Supplementary Table 1

Animal	1375040_at	1375092_at	1375098_at	1375655_at	1375664_at	1375699_at	1375842_at	1375877_at
DD81	28.887026	676.3868	0.8884521	296.0333	93.81745	206.94139	84.611725	0.7880592
DD84	44.477386	618.1717	6.5772104	300.219	90.7268	227.9412	49.364227	6.377423
DD85	61.235428	574.8807	1.0833827	282.22833	85.330055	210.19623	97.561	6.7511144
DD86	69.187675	24.556143	1.8220319	324.0646	96.52887	242.86317	127.08575	6.144017
DD83	40.383442	508.29578	5.502968	267.99628	97.75188	186.67409	81.5342	7.101103
DD41	73.75332	208.52136	0.9715405	307.17725	122.47663	271.79636	193.48328	17.595686
DD42	95.81365	68.2913	9.116257	306.60422	112.57684	282.79947	131.5001	36.041054
DD43	83.99079	57.59793	0.9648386	407.1845	89.55387	242.85663	139.21164	9.807566
DD44	39.085903	91.51043	0.4584649	429.7857	107.78479	255.70485	79.18847	2.1059668
DD87	112.12243	105.86743	10.099607	265.55	122.42223	267.52402	171.98672	25.142122
DD2	70.84518	356.78	0.4210496	353.2012	116.54299	221.99854	88.46078	8.531604
DD3	100.51365	80.30755	8.049832	296.76825	151.29996	236.91498	95.69543	8.829145
DD89	88.41311	122.74978	0.5907341	317.29056	249.18884	341.5339	98.02931	4.3735595
DD90	114.10654	10.716768	4.099288	352.2545	368.87085	346.55222	110.50343	9.150525
DD4	351.69788	19.854427	5.8320293	325.98706	259.16223	241.81802	380.57516	3.7702904
DD47	58.66404	25.60465	8.155954	250.84132	119.70661	149.93793	99.32663	26.602802
DD48	34.351814	390.19724	0.97032	248.50404	113.23026	223.36034	59.45862	11.595055
DD49	56.38495	53.68895	1.3950839	294.8109	111.04887	219.73457	97.0111	8.913887
DD50	99.44799	47.055454	5.736164	309.3807	124.5602	249.43695	184.87413	10.558011
DD46	51.926987	81.18095	3.9330454	298.59506	124.70594	254.64685	79.77842	2.747436
DD6	42.53336	196.71715	6.1018085	335.477	166.99931	211.51707	72.60909	4.015079
DD7	59.390427	58.111088	0.8723542	332.4324	203.05142	241.62843	92.58953	8.172175
DD8	67.27521	4.0847383	0.3935954	308.3432	195.63637	203.80573	142.06412	4.249928
DD5	87.21761	29.806782	6.117588	311.0877	200.71465	151.4487	105.86049	11.147433
DD9	158.1027	6.564377	2.3993409	335.771	173.44737	156.10753	234.91498	4.9979444
DD103	44.90402	118.24583	1.6677114	154.0179	314.126	488.825	152.94489	2.0192716
DD101	38.95278	450.377	0.8743622	111.56461	477.6447	308.52316	97.96648	1.1534743
DD102	38.906	143.91512	9.557468	135.8974	329.73846	413.76892	73.87994	1.1171716
DD105	29.436172	419.42404	10.510792	125.72398	440.3316	378.59036	72.28339	7.5280066
DD106	39.044075	448.94055	9.041759	172.77747	83.32347	338.22015	109.3486	9.110805
DD62	31.049824	395.77823	0.9753233	152.422	99.30567	407.41486	54.71018	1.0593507
DD64	48.76608	122.33651	10.848677	169.94858	440.56424	482.41214	108.7772	7.7169957
DD107	40.116924	52.83118	7.1894145	339.29324	465.90753	477.54562	71.25707	5.1288576
DD108	31.66722	60.910683	8.455114	165.2393	352.66336	442.51788	63.12829	6.076763
DD63	32.46098	44.764595	1.7365947	167.1667	356.86227	454.2283	53.06696	2.56365
DD23	34.294403	18.435898	3.2962914	174.2264	458.68018	513.3773	43.94688	5.1751113
DD24	50.41159	26.689339	4.8254304	241.14792	728.57715	502.35947	36.083656	5.8740034
DD110	39.426155	115.77521	0.6548436	235.99812	786.8214	478.1776	49.028603	5.9840264
DD22	32.28294	76.41991	4.923715	183.64362	730.9073	510.33063	38.25099	3.96215
DD109	41.797497	27.43986	9.665506	121.50826	528.8574	465.3044	30.275509	11.182033
DD65	55.687878	19.832108	5.762925	247.71759	465.92664	478.18942	79.84875	3.4972866
DD66	36.052944	2.5651255	464.0217	174.56049	855.1802	524.2781	56.05192	48.499767
DD67	32.14082	52.481487	16.11231	114.89655	421.01053	352.01562	29.138071	1.9682847
DD69	39.76559	17.663263	7.0554028	159.64748	496.18066	512.2259	52.914562	8.771227
DD68	37.007248	22.63367	12.265206	154.72096	486.37262	552.05457	51.054157	1.4610254
DD25	32.701035	3.6221476	7.0056787	122.70248	474.17633	486.07977	60.465576	21.703043
DD28	40.347908	14.573016	1899.3713	150.782	427.95755	431.87393	50.612606	9.630022
DD29	35.780235	14.774385	4.113089	149.52376	791.9387	429.873	36.944126	22.3841
DD27	24.102129	33.753376	5.105545	237.50484	410.79578	415.92624	53.106106	21.624786
DD30	30.827288	4.287894	1.251871	124.20696	515.12823	458.82355	48.17243	22.884947

On-Line Supplementary Table 1

Animal	1375891_at	1375909_at	1375924_at	1375961_at	1375996_at	1376062_at	1376106_at	1376129_at
DD81	61.216297	420.52124	10.472637	38.844353	65.00787	17.294989	79.73309	97.3262
DD84	59.47233	393.01364	10.80652	42.388268	35.82801	18.433775	70.54	100.32716
DD85	83.7835	421.29297	3.756018	63.40902	21.872686	9.633507	99.06916	79.65863
DD86	92.18983	430.90103	26.604437	47.256397	8.575863	14.610642	163.70367	77.92387
DD83	61.223446	445.57687	5.0088773	53.551556	18.719852	20.746902	86.71878	82.87047
DD41	3.5120556	397.43192	19.930809	693.00055	21.792528	20.367088	186.10971	112.53314
DD42	30.657434	381.38254	16.903786	1584.7756	14.198923	9.442838	364.9974	101.90543
DD43	22.445133	314.66766	21.575512	43.534904	265.1227	14.336452	92.93138	101.79939
DD44	8.530052	349.50354	17.190912	37.65488	179.3351	17.293772	64.51076	129.5686
DD87	30.458374	430.2076	16.41304	765.4773	30.68224	10.329956	382.23877	125.94496
DD2	26.875673	364.64426	12.667749	274.0638	168.20868	17.96655	144.12125	105.05789
DD3	51.026318	409.07373	19.425972	125.71819	33.57309	26.47044	116.3956	96.95945
DD89	14.748192	399.99463	16.151491	116.94003	40.25971	15.464973	124.04756	198.0116
DD90	22.064537	361.31836	28.439875	101.03592	39.829514	70.806	164.98456	300.31912
DD4	6.1586657	333.40042	17.27129	114.07105	34.21923	19.980051	163.64241	173.87411
DD47	135.37624	416.10214	22.029894	928.1628	29.691782	32.8354	303.72177	91.07245
DD48	92.18268	399.81674	19.067516	484.47562	23.187017	21.267712	138.24335	126.51805
DD49	94.74005	365.0079	13.249257	193.06209	21.709393	33.344326	151.5029	114.43745
DD50	99.76015	375.27386	22.489584	180.64641	25.322903	34.460632	121.68676	98.4022
DD46	23.41711	408.3307	19.149302	194.4529	35.150608	28.474205	120.39139	121.49743
DD6	12.168208	350.34625	16.015343	153.54898	88.558205	14.957214	118.18619	183.79851
DD7	27.685654	403.16223	28.689264	143.3216	41.291367	18.628136	160.57103	209.54527
DD8	25.66171	423.60242	25.16442	201.26053	29.721102	17.95753	124.61414	154.8065
DD5	9.202857	469.69452	33.713078	189.89781	19.914005	32.546776	137.0023	142.10446
DD9	32.164757	438.2401	23.447525	135.39723	43.986153	27.97034	142.72447	123.90089
DD103	88.57864	188.26839	1.7688501	41.33336	78.58123	15.672026	24.368826	171.53613
DD101	68.52386	172.34239	6.1776195	29.022438	37.045563	7.4463677	42.2567	197.93425
DD102	103.34888	161.41878	10.876674	32.155403	161.00742	18.609364	23.900497	178.79538
DD105	59.04393	175.65141	8.671425	23.165253	24.762241	4.521328	47.355072	144.57704
DD106	47.198387	173.14355	8.343636	25.29094	56.13957	22.450916	60.16554	173.4281
DD62	20.393452	206.39198	8.634763	98.55892	40.32051	22.036057	31.1671	308.53104
DD64	39.78802	202.39442	7.10862	367.8432	94.50964	48.51794	23.598253	195.13898
DD107	40.117878	152.4402	13.609823	217.54582	339.115	31.297556	44.76549	329.9269
DD108	71.482635	166.67819	7.437049	129.37401	142.99081	54.92839	26.43921	280.91382
DD63	78.45218	145.7753	15.55477	118.63478	141.08897	64.16453	33.421135	264.0368
DD23	54.27904	213.50735	6.6118097	248.33115	105.24602	38.47344	39.583023	155.61142
DD24	43.815548	202.25537	4.1851983	161.72603	56.029236	41.144825	36.232876	364.33072
DD110	61.352024	213.63362	3.614748	329.48355	35.901306	69.476234	36.008858	235.53915
DD22	56.74119	228.22543	8.33603	319.95145	28.191788	62.33639	38.06361	340.17535
DD109	62.400234	226.67464	3.1521323	368.8823	39.902126	30.089064	55.839115	211.7524
DD65	46.4626	222.37987	9.603621	418.2974	46.15047	75.89261	69.19703	127.2904
DD66	65.91936	218.63504	0.8370155	673.4334	40.734814	24.09606	117.99448	245.3126
DD67	74.34873	202.2721	11.186335	595.07263	22.390306	13.791765	42.300404	98.68894
DD69	68.92953	173.88242	6.7322583	550.07825	72.20285	82.27584	55.208202	295.3348
DD68	68.874954	228.57391	7.450664	589.9841	89.791084	43.588142	72.79384	271.21616
DD25	52.69525	219.5931	1.2491723	1091.4418	41.150616	45.147697	144.67851	178.43527
DD28	83.97927	201.32315	7.6782365	410.94693	23.797947	70.24002	72.36137	239.1375
DD29	103.57295	188.91946	0.6907484	831.7717	30.869778	108.97547	133.31418	122.16676
DD27	73.56994	172.40654	2.945932	974.30927	210.58179	41.87476	148.04512	238.38568
DD30	61.901524	200.95872	11.327949	1102.1162	36.696934	46.032978	147.9165	270.96777

On-Line Supplementary Table 1

Animal	1376197_at	1376226_at	1376292_at	1376327_at	1376332_at	1376373_at	1376550_at	1376562_at
DD81	220.35803	4.705381	13.821012	80.09823	13.257265	4.2215605	24.110798	42.851242
DD84	126.14263	16.749193	7.7645392	46.07799	1.5505835	12.314858	32.397747	42.841305
DD85	58.13759	18.13481	2.1750932	14.16271	23.333017	11.022081	42.187836	27.322065
DD86	43.126297	35.979374	0.8325663	21.119556	17.446383	2.5300486	43.514904	1.8694453
DD83	57.954155	20.303547	6.145066	6.0136347	10.580277	11.120993	30.195162	36.20168
DD41	62.77349	30.206709	4.962299	21.580471	3.5399756	14.165342	22.185553	17.329466
DD42	59.58653	26.98287	10.908245	19.152012	9.513914	3.519472	28.139326	14.114695
DD43	1362.9166	39.14953	18.505335	534.6941	3.1680465	0.9290776	22.391132	31.48621
DD44	868.0015	35.55088	14.234918	404.12665	2.3665695	6.996091	23.977772	32.757065
DD87	91.75007	26.759228	6.1134443	16.660496	21.150146	8.073434	25.176533	18.150705
DD2	607.01996	32.114227	11.118183	354.8316	3.0761616	0.6409676	26.12559	41.723717
DD3	92.595726	33.23149	11.743326	30.152208	2.9095874	5.3847623	33.590973	18.516209
DD89	66.47438	27.195524	7.3706374	28.4624	10.193448	2.6320841	19.888956	17.106697
DD90	147.34908	39.292324	44.208035	36.33228	13.910355	9.179703	42.288677	11.038143
DD4	116.1777	33.601006	14.198609	56.32615	2.5705323	0.2420919	30.495909	13.46902
DD47	107.69265	34.609196	6.6864753	21.376915	13.810938	5.104061	14.743245	16.57617
DD48	87.79907	25.222242	1.2008531	11.986643	9.698516	3.815001	41.173576	18.310055
DD49	109.67145	28.966242	2.6979804	12.568029	5.2993655	2.7358737	49.38512	11.407753
DD50	117.58463	31.088446	16.1684	25.972498	16.57477	1.4359628	39.93108	8.538183
DD46	84.148445	42.79598	3.5804963	25.950762	5.5883236	3.5357907	36.74127	13.644731
DD6	377.3357	37.42617	11.228689	200.03659	13.994962	6.110359	33.649788	24.0672
DD7	148.15982	31.835146	6.683097	55.88211	2.7347414	5.770948	53.329506	20.335024
DD8	85.229065	42.923927	7.812295	39.18376	1.4070636	5.510215	18.369469	12.787257
DD5	77.78311	43.764156	8.636112	43.232246	14.395373	3.5113587	29.24101	4.791836
DD9	167.2765	44.1386	0.6161139	67.63367	12.113396	1.195863	39.11446	12.321078
DD103	324.08713	82.883125	0.9958074	111.58452	47.855045	7.841076	10.242041	37.14235
DD101	142.08221	12.330596	6.8002014	36.543343	33.163822	4.6926184	10.284698	48.097088
DD102	738.20374	94.09234	8.506757	249.88683	42.53067	0.7000268	12.750515	46.043873
DD105	113.21873	59.482983	1.5717093	22.091047	55.588886	7.4987955	17.338348	58.60701
DD106	219.43306	24.693153	2.2242982	73.311874	34.965176	8.466038	2.3978708	40.27531
DD62	115.65966	31.724632	17.470633	58.028805	25.88601	3.511052	11.790703	42.095776
DD64	210.76364	85.65677	32.43155	115.91718	30.06836	4.4535885	16.265926	36.25666
DD107	874.1932	167.55992	26.056484	628.30743	45.8898	6.9133406	9.377637	54.55271
DD108	592.26044	60.96916	45.800297	265.53607	48.290108	8.065675	11.516083	37.750866
DD63	633.04034	56.136337	60.89874	256.5193	48.914597	10.547927	17.230253	29.678865
DD23	273.68665	137.93715	17.56634	139.88954	53.772346	9.250157	12.52829	40.94099
DD24	146.33582	214.26718	15.587781	49.934765	41.06772	11.793098	10.875246	42.440907
DD110	116.05792	38.06515	38.80951	35.6314	61.01811	11.882035	0.7097693	33.18508
DD22	51.027077	141.84747	25.540148	16.82292	36.41906	11.305111	17.604166	38.049232
DD109	81.81762	119.69637	17.657843	22.373016	43.8376	11.450398	5.084611	36.395725
DD65	225.49022	32.12915	61.05028	64.89207	42.099083	11.497717	14.689871	57.94991
DD66	111.27473	205.43199	11.632116	60.470375	49.82934	7.2530413	10.866358	35.71015
DD67	93.04228	146.96817	8.439649	19.778976	47.034576	15.011733	0.8489491	22.06908
DD69	269.81183	41.59816	50.409664	95.05295	51.302036	16.969471	28.558685	39.265194
DD68	281.99933	159.46893	5.154195	97.8756	46.035046	10.980509	0.8455685	44.943302
DD25	107.64812	149.39278	12.845471	41.506668	40.702026	12.289706	1.301038	41.93404
DD28	98.01894	174.86786	89.64674	24.270128	37.7253	9.760806	2.214498	53.473885
DD29	94.837006	301.69983	110.84506	9.981225	50.033478	5.874744	9.28403	34.650433
DD27	909.8506	51.05332	3.4551468	370.03473	35.8119	9.181583	9.346323	44.513733
DD30	110.0527	152.51157	10.796168	45.256634	52.02128	14.467394	0.3217158	41.592106

On-Line Supplementary Table 1

Animal	1376635_at	1376748_at	1376840_at	1376875_at	1376929_at	1377011_at	1377097_at	1377334_at
DD81	340.72614	163.55098	22.474277	37.88642	890.8077	107.46181	22.917242	888.2334
DD84	392.3608	122.55193	24.385286	44.29253	640.7135	120.53169	18.77391	953.14307
DD85	355.93915	83.091064	13.083505	33.19677	572.85156	132.64282	20.884584	673.7418
DD86	637.9947	8.313029	20.333685	43.708057	65.97403	102.20908	31.186016	606.631
DD83	396.47705	112.40691	21.33823	39.018726	614.24	136.8431	20.197596	728.4362
DD41	304.5291	47.43621	16.0097	20.004324	308.93237	117.92675	17.227879	696.62946
DD42	324.92792	3.788084	11.650243	32.158485	100.6321	95.20453	9.388574	751.25684
DD43	311.9477	4.094202	32.48591	46.582386	88.045586	100.02461	28.594936	3680.9216
DD44	363.49545	23.429678	16.272903	54.440884	94.29573	97.23517	26.40219	2683.211
DD87	359.28912	8.825026	16.359346	34.150146	86.61785	105.6248	13.706565	899.7114
DD2	48.230507	73.14145	14.927917	42.75104	462.06592	142.60088	22.24627	2135.808
DD3	117.88958	30.19134	16.438358	32.25989	137.85858	183.85297	21.93502	1421.5955
DD89	98.40767	35.439392	28.711452	64.67209	117.49471	171.9701	19.442472	1765.4531
DD90	92.33852	1.6955867	54.40859	54.609577	25.490513	289.448	17.186245	1721.7885
DD4	86.22547	9.792455	22.125994	42.850903	82.51723	235.63501	16.74785	1498.9265
DD47	156.0274	15.92381	13.176291	46.948334	52.140484	112.69573	15.476131	1086.1139
DD48	74.604454	124.85538	21.831968	49.380924	452.94904	156.14778	20.847315	952.44464
DD49	93.53353	19.444225	17.024645	52.37816	139.68614	216.20763	33.24688	1234.3553
DD50	86.53675	9.1189165	25.6619	30.75036	74.8814	180.9973	28.218277	1171.6398
DD46	74.06922	27.842722	22.848013	47.960148	145.80066	284.00836	19.81145	1171.551
DD6	41.549786	62.489185	18.831076	49.89391	244.31718	135.42586	26.21101	1807.5077
DD7	78.7377	14.320251	22.027422	61.59257	108.50395	144.52567	29.15803	1655.274
DD8	107.84268	3.7739003	20.632656	51.532665	30.50285	230.54756	17.322138	991.7727
DD5	66.28294	17.895481	16.991962	48.779694	40.00365	161.89664	21.026669	1235.3983
DD9	80.29259	0.4279544	14.726851	48.45179	49.413773	229.98079	32.43082	1388.0482
DD103	777.14734	28.064999	161.88734	84.034195	250.10529	328.77945	29.19025	472.90942
DD101	556.41016	112.94701	135.04243	76.960686	891.4147	449.53143	23.98011	12.206336
DD102	669.8683	40.908287	174.52377	59.76505	253.14433	383.57904	32.92546	1811.4877
DD105	530.1201	93.94501	173.11221	74.3968	632.79285	117.37525	23.52872	145.29704
DD106	574.7545	82.546036	144.24168	60.826824	612.94775	384.93802	33.731457	16.36032
DD62	238.04333	104.99065	109.70037	83.79889	498.61395	131.8681	17.49389	522.41504
DD64	278.4068	54.030098	95.20081	85.36402	136.91043	456.97476	33.622593	24.179005
DD107	160.96713	13.687505	200.22284	131.14279	79.0883	373.5005	26.923233	1872.3021
DD108	423.90253	17.9679	199.68916	90.88349	130.86945	198.451	35.942535	1105.161
DD63	388.02673	19.238188	218.28218	76.42142	163.322	169.82433	43.361275	1421.7603
DD23	362.7379	4.745536	122.87866	101.24217	39.259853	358.05786	44.57208	983.68
DD24	376.79855	17.058262	122.71839	84.30846	107.84078	445.53726	44.006954	797.0076
DD110	216.73552	48.58358	167.48868	87.0743	34.8072	120.91184	56.232857	702.7936
DD22	345.4085	33.11179	93.607	107.43592	63.651714	505.1044	55.549976	748.603
DD109	389.1929	4.777438	174.2779	94.94457	28.959768	112.93273	66.292435	836.6143
DD65	257.69577	5.1001687	186.1824	97.40631	25.57379	178.2752	86.16165	14.152724
DD66	273.6463	11.118997	198.3508	71.825455	35.80678	403.62048	33.348312	17.181932
DD67	914.3721	19.538382	122.2561	101.20215	49.882492	125.06715	90.772736	500.05286
DD69	261.22867	6.7965527	170.88182	98.769226	26.14943	516.6494	57.44955	859.8029
DD68	216.69456	1.3429825	176.5286	98.47819	30.231031	130.2171	90.83901	1231.197
DD25	256.39178	2.1701539	281.91467	97.97363	40.40376	606.24603	82.04443	864.33276
DD28	242.2166	1.3768711	130.78328	75.43165	28.9645	179.03534	80.78853	27.453842
DD29	421.23438	0.7507635	169.28922	102.46491	33.66353	141.55005	54.06024	762.0169
DD27	149.28957	28.430977	244.62874	76.236694	45.472973	566.11365	46.377403	28.665539
DD30	242.14989	2.4962595	306.9966	96.77408	27.13423	617.0676	82.67346	899.2306

On-Line Supplementary Table 1

Animal	1377407_at	1377448_at	1377452_at	1377529_at	1377610_at	1377778_at	1377797_at	1377873_at
DD81	45.213448	1085.931	21.170628	26.8461	818.05444	28.1295	47.441963	29.28551
DD84	45.255646	1011.528	56.194378	29.635231	745.0359	29.591187	29.773457	34.294147
DD85	34.46477	854.82336	31.57363	15.813751	868.0702	16.764116	26.600096	35.522026
DD86	34.009243	22.365805	107.31434	5.7699847	49.38662	26.298954	48.164246	40.35908
DD83	59.962723	986.46796	34.50466	21.384495	784.61	22.995546	61.15559	31.441631
DD41	37.336384	222.52777	78.55561	20.185875	224.84613	32.846825	54.346382	22.75419
DD42	51.6441	101.59418	107.83521	17.036205	73.93454	13.380857	65.180824	6.899324
DD43	31.089066	112.38657	101.85384	11.70013	77.02178	18.433788	102.06622	9.849332
DD44	31.018284	151.79025	68.18441	11.302959	82.91257	19.06359	62.930557	18.948307
DD87	38.982212	120.68553	113.7189	20.509106	72.03528	29.889687	61.017563	12.54614
DD2	13.161197	789.52295	50.98043	13.618668	553.78955	7.297407	46.234615	14.167609
DD3	47.864845	118.04878	104.90567	3.074117	139.00291	9.998133	70.2692	14.238274
DD89	33.789757	140.89586	14.646762	10.588279	134.24348	26.604652	33.243332	12.000692
DD90	34.83964	16.186558	4.5436406	8.091455	4.802735	22.737617	25.560099	10.082875
DD4	31.086836	42.63877	3.1796646	6.340289	64.32739	23.34709	51.573162	5.175006
DD47	25.680214	50.894943	97.7107	30.830807	37.807716	21.64917	57.363483	8.230666
DD48	37.25383	874.2303	124.13738	21.995243	772.01715	18.355572	74.63879	4.279249
DD49	21.985882	136.58574	66.92176	16.415771	90.4341	14.023132	65.21606	7.639784
DD50	42.905487	61.197155	71.99979	10.3747	59.968422	23.677553	17.561678	3.224211
DD46	33.874825	170.36981	66.76319	14.688025	147.04666	17.121973	45.29626	8.845749
DD6	41.594246	508.6747	35.681396	23.122984	296.97998	25.776772	49.149822	7.355419
DD7	31.725506	126.51601	17.630106	24.61459	114.95203	14.748446	10.492655	4.9321923
DD8	45.9251	11.709612	9.094885	6.028859	1.2390554	6.2385283	21.238754	8.857527
DD5	43.744186	16.748676	34.0114	8.090505	37.413273	3.373125	25.76014	9.235822
DD9	36.161755	13.618305	22.663033	7.631395	11.920181	9.5975485	47.59471	4.5678234
DD103	117.95752	272.93762	168.4048	8.68729	289.09503	157.88855	114.6711	5.7805257
DD101	31.066355	1086.4598	171.67624	13.552077	512.60223	171.3976	76.67531	5.8824754
DD102	118.70125	395.35892	149.51128	5.0326786	377.39713	94.00669	128.37875	1.9718826
DD105	103.38375	888.5733	91.706474	11.271757	709.73553	180.65065	80.7334	3.607509
DD106	133.36247	836.82983	399.70355	16.2176	751.5637	151.13313	97.89968	0.990096
DD62	117.34807	885.1924	44.987984	11.886886	408.2147	51.96363	58.72005	3.169334
DD64	72.57328	329.5026	14.254261	6.108902	143.59529	129.48726	70.00013	6.985617
DD107	60.837627	90.11551	12.268162	4.832347	33.38998	76.63438	100.15712	3.1361673
DD108	86.74447	154.6244	184.32219	1.1357937	90.10848	99.51348	94.99433	3.2814841
DD63	71.372185	138.99501	186.15454	8.042352	81.96635	103.88818	93.48619	6.349558
DD23	103.54949	37.026566	259.58264	6.878398	27.038355	111.86844	131.52977	9.28938
DD24	80.202934	47.7093	79.37288	5.936342	42.446415	116.0239	109.75508	2.6284618
DD110	49.32938	218.91016	234.45084	5.1802487	36.42033	166.15161	95.98209	13.866056
DD22	75.442406	149.28291	79.32635	4.785104	72.23764	82.91879	114.91207	4.7047753
DD109	118.29895	34.480038	142.68112	10.840031	8.313312	157.64548	77.82387	2.6034126
DD65	121.14849	30.730364	152.82771	0.4144976	19.816322	155.14288	80.8985	16.92767
DD66	63.04342	10.771364	328.92767	13.001437	2.5324109	68.150444	97.99553	1.1793861
DD67	155.34245	80.86741	130.20471	5.1925592	32.328537	128.08177	122.00676	6.3491564
DD69	83.23736	24.085546	100.18007	5.9692006	9.742001	180.4242	88.65325	11.774416
DD68	60.753387	51.457195	305.79474	6.7829547	14.376216	162.57812	141.50621	6.332847
DD25	158.2374	16.109875	47.44905	41.863827	6.1097465	107.5131	72.37149	0.755061
DD28	70.19015	14.111699	27.475166	6.8098807	18.22694	109.02982	65.9506	11.599694
DD29	61.219868	33.09598	195.92049	8.068472	3.4972465	80.97171	71.20673	5.5206614
DD27	85.681465	151.59767	28.856647	22.489273	26.043015	99.4126	85.37664	2.5811918
DD30	178.755	12.128045	54.722446	31.876001	1.9261359	129.71185	79.15522	2.8945699

On-Line Supplementary Table 1

Animal	1377950_at	1377960_at	1378062_at	1378193_at	1378265_at	1378413_at	1378458_at	1378541_at
DD81	227.36276	95.4981	127.89334	41.779247	62.662113	72.01186	28.83663	13.972801
DD84	187.10347	86.94011	125.30358	22.244236	77.89171	50.470566	36.42253	23.95927
DD85	185.28679	108.72528	152.45291	21.081472	94.70025	35.22391	28.868303	20.03099
DD86	218.84291	83.95812	218.7933	20.485006	102.20533	38.931545	33.7649	29.532766
DD83	159.29825	98.98238	184.81589	34.771343	51.57321	32.466076	19.526058	17.627241
DD41	396.34656	92.560104	141.24216	48.634174	34.731964	68.02113	34.493587	24.000942
DD42	319.77792	86.96242	109.94019	42.146965	42.039364	87.86752	39.501205	28.594294
DD43	650.03864	127.72525	115.93324	94.30106	64.56816	264.97134	19.542803	31.841581
DD44	554.1185	124.92356	101.39339	107.31287	42.905704	160.82242	27.753733	30.061525
DD87	365.56665	102.91821	113.98691	43.843502	60.84258	65.369225	35.482746	25.08087
DD2	301.72543	90.7676	152.64024	47.501427	36.32543	119.98907	29.899788	21.399979
DD3	364.21872	102.5891	174.59604	32.58937	66.020134	29.712484	26.490643	20.13972
DD89	488.58328	116.28899	120.29885	65.091995	41.237015	61.654816	32.64615	33.461964
DD90	447.59067	123.97904	167.86774	37.30878	55.346138	53.946114	28.368233	34.1858
DD4	463.7929	108.34782	150.4694	58.88923	59.450794	49.23439	28.993572	37.055
DD47	254.22318	115.69727	177.88467	52.924335	59.443005	60.39262	27.736744	25.729355
DD48	238.16154	93.9679	126.57703	34.98511	78.721664	49.788345	46.42929	25.84302
DD49	392.83173	87.28364	194.87177	62.794693	112.3316	38.86968	21.633074	26.167242
DD50	380.6052	86.80835	158.93803	44.20608	125.31207	40.225895	54.465214	22.84948
DD46	384.21762	108.74545	171.31142	49.974754	62.928032	27.915525	20.67731	39.81776
DD6	385.23572	128.95242	163.68964	74.608086	48.288456	103.06089	27.63969	38.835934
DD7	414.7145	111.91729	192.3103	50.645847	53.56405	57.269646	28.70411	37.136646
DD8	338.5676	102.23799	174.15947	52.759342	53.077896	38.649803	16.674955	39.95196
DD5	462.09363	85.04477	192.85905	73.3275	51.805782	33.87798	13.572399	26.463066
DD9	462.6172	121.98016	187.28363	77.36176	51.522907	31.902214	13.971766	31.99715
DD103	5.4973574	77.23164	114.91619	63.419735	23.921244	197.36511	63.162415	56.4735
DD101	42.738846	28.220108	81.24009	41.638382	17.93418	76.487114	61.572174	39.581047
DD102	4.2975492	113.69188	102.3259	92.726364	25.192148	276.19897	42.831707	62.74861
DD105	1.3335333	65.006935	121.21754	46.545433	24.575787	104.15032	88.88239	47.056107
DD106	70.935585	74.82192	62.433846	50.616634	23.795553	153.6878	56.61697	48.398567
DD62	0.5104904	58.038605	41.31383	61.54784	9.381909	76.98574	62.36969	64.33494
DD64	7.7060666	18.222603	52.902496	106.16657	4.9303875	147.08925	52.08253	73.57669
DD107	0.2877844	84.244286	49.620148	112.60235	14.245113	592.3319	52.20708	98.55914
DD108	1.0040067	75.468475	102.24826	122.5789	30.001528	281.50867	74.947014	65.300964
DD63	3.4860213	72.42815	96.72149	116.94888	17.094421	272.35336	57.128532	48.41309
DD23	5.201583	48.71355	52.873867	108.60115	8.331293	249.3774	66.04189	87.54504
DD24	3.0500696	55.707897	85.95297	124.83476	24.765207	129.70178	96.039925	73.04026
DD110	534.27686	35.886383	73.37205	98.17687	25.112318	85.4458	90.004074	74.01722
DD22	0.3207114	22.390387	67.84302	153.0377	14.591993	96.81802	64.33537	83.0002
DD109	451.5664	79.862656	118.40387	127.08751	22.776196	126.98145	55.539917	82.122765
DD65	3.8091683	79.76458	69.92435	111.44528	4.84316	131.40004	82.85142	86.421814
DD66	6.046464	68.481094	98.31415	85.72198	12.290486	165.5903	68.12145	76.15238
DD67	154.3044	25.592457	101.72527	70.98061	11.487391	121.95512	31.814837	74.51171
DD69	6.4704037	37.2522	85.24684	150.5455	17.64777	153.26163	100.31957	78.38509
DD68	4.0536933	79.0677	121.10792	153.46634	13.041662	218.9921	85.06299	75.32453
DD25	5.8896	62.67211	52.7206	97.72577	32.70275	189.32819	49.141945	83.47364
DD28	0.1659488	73.05247	59.793682	86.47836	13.970328	103.62758	52.405346	90.2226
DD29	337.7004	35.024986	75.863594	72.49266	24.119944	228.38295	90.73492	97.41459
DD27	2.3803227	36.203064	101.48709	96.70467	15.394295	456.96484	69.68031	84.20631
DD30	7.4785366	78.35753	67.16521	75.66276	24.8387	209.57149	86.48756	86.7676

On-Line Supplementary Table 1

Animal	1378624_at	1378857_at
DD81	15.942635	693.4064
DD84	10.0024	668.49854
DD85	13.324475	507.97534
DD86	15.313739	25.69307
DD83	12.567055	543.2777
DD41	7.491917	117.39526
DD42	0.4738059	43.10943
DD43	5.5398216	35.332344
DD44	14.83097	40.79278
DD87	3.2241585	43.893135
DD2	4.6081314	93.61188
DD3	1.003641	19.668192
DD89	7.6104865	39.340115
DD90	5.9493275	1.5125262
DD4	6.8623257	3.035798
DD47	4.1617513	9.171505
DD48	2.6170297	92.15557
DD49	0.7238777	7.5832405
DD50	10.151987	1.3894027
DD46	6.0427	26.773869
DD6	6.397991	75.31905
DD7	8.23366	24.580761
DD8	7.422525	3.2584043
DD5	0.6517307	2.6491215
DD9	2.0814838	0.8129515
DD103	11.035327	207.66135
DD101	12.327878	827.8903
DD102	8.750454	263.4083
DD105	18.524921	825.70105
DD106	9.693994	769.25806
DD62	8.577123	212.89822
DD64	8.492681	42.606197
DD107	5.226492	12.770739
DD108	13.618688	49.621952
DD63	10.705621	46.914806
DD23	12.282795	1.2971915
DD24	5.2915673	3.0644817
DD110	12.010509	14.025675
DD22	5.4420266	14.981571
DD109	2.075757	3.0096374
DD65	9.95688	0.6671365
DD66	6.459505	0.7291841
DD67	10.307988	10.744406
DD69	14.705979	1.4731816
DD68	8.560702	0.8773408
DD25	7.92068	0.793695
DD28	11.716383	5.6348815
DD29	16.0194	1.4355551
DD27	12.237954	1.1437944
DD30	2.5914237	5.287386

On-Line Supplementary Table 1

Animal	Strain	Age (Week)	1378866_at	1379047_at	1379065_at	1379249_at	1379306_at	1379357_at
DD81	GK	4	216.57748	14.09488	139.04906	143.49739	31.78368	25.91941
DD84	GK	4	162.9292	22.792614	247.06468	101.11397	23.736177	16.929941
DD85	GK	4	177.8752	25.301643	296.6658	151.24066	39.837452	18.603348
DD86	GK	4	252.12863	6.247877	402.8229	112.30605	21.76271	17.30886
DD83	GK	4	173.06815	15.075417	422.55194	106.65116	35.67002	8.645662
DD41	GK	8	156.88684	14.060219	1259.1942	159.90193	38.335056	21.269035
DD42	GK	8	127.62418	17.096855	1336.5802	125.10185	35.493538	30.429073
DD43	GK	8	239.66591	31.711643	1109.8612	157.44493	29.267773	20.914423
DD44	GK	8	222.2948	35.8319	1376.8634	147.63486	37.745056	35.04271
DD87	GK	8	187.46065	11.13376	1369.3239	131.45488	31.154259	22.16203
DD2	GK	12	224.81566	29.499243	22.534422	141.5476	25.431786	26.90237
DD3	GK	12	207.19019	14.475289	63.608624	128.9641	24.710304	27.86698
DD89	GK	12	215.5316	25.457344	145.34091	165.4861	33.251	21.658894
DD90	GK	12	286.10095	28.834745	39.95731	228.22462	25.169857	22.645323
DD4	GK	12	239.3867	22.710869	8.508041	137.22331	41.024006	25.41431
DD47	GK	16	174.89742	22.457521	39.94804	86.11666	25.13328	21.658333
DD48	GK	16	213.06767	4.8083453	40.945705	149.23637	28.58548	23.719734
DD49	GK	16	267.80252	1.5220444	9.649074	145.8619	28.02417	40.863804
DD50	GK	16	315.0281	25.78024	9.682814	168.5283	20.560383	7.24461
DD46	GK	16	249.91135	9.479395	33.514412	172.16035	36.837414	28.649794
DD6	GK	20	230.15808	15.6508	31.64539	205.93152	34.280476	38.88217
DD7	GK	20	262.40125	16.109783	31.500929	235.39659	32.524815	23.770418
DD8	GK	20	182.17833	19.16104	31.088362	224.28206	41.452263	17.600039
DD5	GK	20	155.18152	23.787601	8.216477	158.34618	33.539864	41.94868
DD9	GK	20	149.5382	19.297035	50.10477	165.20563	39.702644	32.819897
DD103	WKY	4	23.04029	3.0791621	979.438	510.6882	78.6623	106.53368
DD101	WKY	4	15.509005	11.640488	652.72424	445.4661	73.17796	32.24569
DD102	WKY	4	114.09622	11.622966	861.38495	480.13092	94.809364	62.49067
DD105	WKY	4	125.67761	3.209631	740.18146	429.70752	84.33163	37.856136
DD106	WKY	4	72.10547	9.083941	417.75436	488.57837	110.14094	52.833115
DD62	WKY	8	94.35365	3.7143013	699.22015	121.24556	23.994133	43.137405
DD64	WKY	8	136.46805	14.430355	228.06516	406.11234	102.7482	149.01112
DD107	WKY	8	183.433	24.313175	302.99744	880.0271	80.14424	105.38184
DD108	WKY	8	48.470673	4.956789	1426.8688	551.4821	81.16871	136.01886
DD63	WKY	8	46.43896	5.525375	1536.7473	537.99634	80.8613	140.367
DD23	WKY	12	146.80486	8.426561	339.36935	353.4412	91.6351	191.77223
DD24	WKY	12	149.93396	10.374113	936.21936	661.7275	76.522804	75.99961
DD110	WKY	12	20.723516	2.2750053	382.84113	401.64307	70.1144	64.82366
DD22	WKY	12	122.62582	14.219832	364.439	323.45602	70.94883	139.71727
DD109	WKY	12	124.67114	9.078858	245.08165	476.56754	67.085335	160.3316
DD65	WKY	16	26.203938	8.764291	344.34552	579.2944	61.55111	24.009626
DD66	WKY	16	112.41459	16.73672	331.51572	851.25586	82.94023	120.49623
DD67	WKY	16	111.33791	3.1736908	215.61115	517.70233	76.873344	58.581055
DD69	WKY	16	184.804	21.517124	258.92767	167.54846	88.50704	89.32252
DD68	WKY	16	182.13809	16.574812	353.8233	513.6559	79.122	90.46323
DD25	WKY	20	130.3917	4.2472134	213.08994	1014.2094	79.25354	188.32562
DD28	WKY	20	13.892318	5.5375624	165.12817	1097.5444	77.56616	111.45085
DD29	WKY	20	143.57687	4.894602	278.63223	1066.3209	89.89418	67.64153
DD27	WKY	20	142.05084	3.9297547	164.31848	206.383	60.44164	321.15955
DD30	WKY	20	98.67194	16.202272	175.50133	1113.5756	95.65932	189.90805

On-Line Supplementary Table 1

Animal	1379382_at	1379390_at	1379433_at	1379435_at	1379446_at	1379496_at	1379513_at	1379534_at
DD81	38.473286	1.9846549	508.41888	1.79027	203.04552	34.040268	20.099918	530.6727
DD84	18.533783	21.091076	585.86536	2.102541	140.65625	28.572931	12.778921	489.02655
DD85	44.173218	9.567259	691.9707	3.89548	4.500837	24.018576	12.967302	532.728
DD86	37.43899	13.165875	1098.9451	3.8747275	6.6956134	36.993126	10.800236	23.426794
DD83	35.64996	20.967838	703.11383	1.405391	65.316	21.612793	6.8350396	566.2924
DD41	68.352264	5.4470706	881.92084	3.4140556	10.098872	38.810463	13.026587	137.3891
DD42	48.25959	5.975343	839.934	6.7166953	5.45103	32.8235	4.052103	45.64076
DD43	52.576534	61.21314	886.8748	2.2529728	799.44714	93.53953	11.756859	74.488235
DD44	73.1584	36.255753	698.6658	2.2846048	606.8496	68.73415	14.90038	60.730373
DD87	73.92373	15.032867	819.95435	2.3524606	10.955077	55.374134	0.912178	69.86182
DD2	266.27612	44.06374	708.28906	1.7965397	613.4061	68.07916	10.103339	427.00696
DD3	332.8552	14.391015	1001.1733	2.2687852	22.317093	62.437828	5.8356504	97.819496
DD89	312.1671	3.1314256	911.6458	5.4909797	32.268517	44.018124	6.504897	122.87183
DD90	381.64392	19.97664	1264.7372	10.333883	16.970703	33.285347	61.619396	17.880028
DD4	378.0365	27.782055	1150.8466	1.5102488	17.560246	18.995268	18.136166	52.90264
DD47	268.1324	16.904308	785.4803	2.079871	1.6429814	66.48687	1.4834764	25.940332
DD48	249.4001	2.3299932	561.35834	1.6158453	3.2070746	56.004513	3.6137624	637.2117
DD49	393.2734	4.214742	928.1902	0.8542578	2.8237345	71.41616	10.112524	93.31251
DD50	355.084	20.651775	1080.688	3.8861449	0.8193286	86.20275	27.704763	31.875172
DD46	503.0914	9.5325365	935.2078	8.289278	7.1547136	57.363464	6.8274655	174.5601
DD6	417.28915	23.780376	702.36194	2.0797439	327.06088	69.84576	3.229038	399.17795
DD7	481.40762	4.421379	948.6749	7.524789	250.5637	36.3345	3.8443177	111.81343
DD8	516.07367	19.375933	891.396	1.3304543	7.0515685	63.372383	8.641016	10.041458
DD5	430.62152	13.160689	874.98254	9.024703	17.499601	43.577065	5.356796	22.665705
DD9	515.35187	15.991823	949.84875	0.8681207	53.47809	65.97457	1.2719452	14.041069
DD103	28.286873	65.62461	409.51047	65.80753	154.57433	60.145992	0.7562471	149.76474
DD101	21.246885	35.79568	227.75935	85.043686	70.417564	34.8399	1.1185157	549.68933
DD102	19.879128	82.30315	311.96005	55.010136	379.40295	98.03591	11.837919	189.6215
DD105	28.233978	32.76414	286.8305	65.426895	27.392065	46.116295	4.447656	427.27753
DD106	32.331184	45.30906	286.95108	81.77304	113.01373	41.44904	11.33667	569.84973
DD62	66.957664	22.609592	278.12006	94.447784	80.18041	118.7217	19.325274	433.8616
DD64	82.28093	51.446423	355.87085	118.00573	290.1809	187.12717	44.118008	202.27151
DD107	49.81871	73.49592	371.616	91.75258	1202.8805	258.88068	31.184015	48.3864
DD108	57.228382	51.568684	390.12354	65.04285	378.14478	158.27429	46.10407	67.012634
DD63	59.339096	76.44165	416.44717	62.149796	370.57242	182.27501	57.5206	71.22756
DD23	90.07518	33.50766	439.22757	59.47452	162.38194	236.42404	18.75558	20.73398
DD24	76.043304	39.366547	531.7733	74.92825	51.48511	148.67445	16.874706	40.69405
DD110	152.72365	48.905636	431.53662	124.94346	29.991774	195.38365	39.282684	178.95914
DD22	95.37282	30.45415	426.2365	2.0108378	9.585913	164.0154	30.337833	99.75129
DD109	90.27267	35.113155	485.30682	92.55757	7.94679	197.8775	10.60685	37.597717
DD65	104.02585	62.199295	565.95715	112.10268	61.456116	169.88033	61.342266	33.967052
DD66	120.64587	24.511768	420.3106	61.927242	37.138542	161.45956	7.2781425	14.137775
DD67	46.46789	27.370686	412.3196	86.95778	7.139239	136.49033	2.1155171	55.86376
DD69	69.62963	53.50443	448.2931	136.04678	103.45017	182.18658	56.000454	22.366596
DD68	190.68292	30.92168	500.62024	65.12868	149.73477	178.0566	4.999044	30.539316
DD25	175.78008	21.743849	552.6215	55.722126	23.052322	199.36	4.5976343	5.2842345
DD28	236.20055	52.490482	405.2171	108.74831	3.1678607	146.46141	78.35443	13.378054
DD29	82.37442	57.293564	453.07336	68.66416	11.487139	156.47185	99.771255	10.221441
DD27	143.00337	49.663734	377.76993	59.185024	729.33167	237.32358	1.589112	96.4774
DD30	182.10167	48.584435	573.07465	59.32914	24.703634	219.07059	7.7044983	7.933592

On-Line Supplementary Table 1

Animal	1379615_at	1379653_a_	1379707_at	1379735_at	1379747_at	1379818_at	1379846_at	1379859_at
DD81	49.28945	18.79434	321.38278	1.706468	9.600008	277.81577	11.112171	124.96268
DD84	53.55416	15.710455	314.5022	0.6185045	15.375875	209.51291	8.395637	125.51489
DD85	71.72651	3.3716712	293.9722	10.488965	2.145491	167.2615	25.77174	72.6235
DD86	81.86231	4.3933706	26.119886	4.070006	16.56805	240.49	31.446592	124.60747
DD83	57.130424	5.8962526	322.24597	4.7002907	7.1303043	171.81552	25.889187	108.73473
DD41	64.25733	4.3693886	103.62252	1.2254754	3.0218053	339.3163	22.740164	121.29223
DD42	59.69191	1.0628829	52.853962	0.9158874	5.2121964	314.40637	26.38644	141.77936
DD43	81.194374	164.66327	59.011276	7.6902075	5.8016706	1037.1351	28.285156	156.02798
DD44	75.26771	115.08102	67.18769	1.2583004	11.032095	821.13007	32.496315	204.48547
DD87	63.480682	7.417016	40.000797	2.3873796	7.9857492	293.04626	25.130518	134.73676
DD2	49.592712	64.757545	256.87408	1.5473586	8.232898	696.46045	41.519253	162.16681
DD3	41.74699	11.370495	55.394245	5.966751	4.4627213	491.1168	40.23972	105.92541
DD89	65.90748	12.654651	64.19019	0.4980145	6.9747033	432.9248	38.6448	138.98044
DD90	57.168095	14.744666	27.285744	6.4703145	4.2208133	583.0371	78.816986	261.6804
DD4	40.660866	17.15926	29.670715	0.6752257	6.0523577	562.26	47.730354	179.71472
DD47	44.50013	7.630531	40.878292	2.882572	1.8538159	500.18793	28.507889	99.735504
DD48	37.09426	6.622724	295.6961	5.304326	1.4696676	408.13428	27.551764	84.228455
DD49	53.351048	10.574287	62.315002	0.4174176	6.337233	733.5602	36.31143	109.43937
DD50	61.851673	10.661531	41.61854	1.2946067	0.5504241	641.40875	45.055527	87.9614
DD46	50.36666	6.4022026	86.80926	4.7876554	3.6237013	680.2478	82.32586	149.72559
DD6	47.376137	48.641457	194.24583	0.854274	5.3879466	697.496	76.11743	173.68365
DD7	44.307327	7.5110927	71.24239	3.995576	5.0486913	703.94763	79.22276	168.01009
DD8	47.37926	14.53154	1.5834637	7.934921	5.8181047	695.7543	84.14556	133.32956
DD5	32.07285	19.776518	19.938757	6.569863	7.664943	619.15125	67.1127	98.42277
DD9	46.620255	15.478641	1.9380575	9.497534	7.824864	616.2787	50.71467	100.55552
DD103	287.49402	41.77478	62.931152	16.521976	227.0447	117.95776	2.2848954	10.292839
DD101	44.109818	14.480494	280.37585	19.838856	115.01166	21.881481	7.6533937	7.028191
DD102	260.023	62.982216	75.29564	20.019823	56.427322	183.9832	4.9631224	82.97515
DD105	154.80435	17.805733	220.80551	18.055891	97.11147	27.41205	10.118807	64.039856
DD106	45.7064	28.960773	218.51434	23.405493	100.03282	25.269869	7.843337	15.677495
DD62	142.91652	9.617669	302.67178	19.194529	31.379627	64.576355	1.508957	88.33252
DD64	60.088375	15.642616	166.32024	29.42424	26.281332	74.25588	6.7739544	105.5332
DD107	139.63876	127.48951	49.800945	23.80781	19.100014	289.50742	1.8557181	180.37154
DD108	261.74704	66.398346	51.09783	15.51358	43.352703	138.68376	9.129569	59.03132
DD63	226.05147	67.467255	41.116856	21.484507	53.281025	175.95319	8.318415	37.958668
DD23	97.89624	48.53195	27.05766	16.153847	21.490175	171.42226	9.117668	76.31339
DD24	208.08784	32.30368	37.901604	18.757647	21.933872	82.98654	4.094286	73.728806
DD110	63.85361	13.114394	110.74121	19.660923	10.960152	100.75961	4.01006	12.638591
DD22	225.43236	11.774216	79.21269	18.254229	12.843803	73.2829	5.654891	60.720882
DD109	170.02647	18.38482	23.286724	9.606694	24.918049	83.55168	7.2630153	100.32526
DD65	52.831448	22.15352	33.323093	23.19192	14.09107	48.957966	20.924496	89.679214
DD66	45.694195	9.178518	15.250068	22.915077	62.98267	68.67392	11.061153	72.45131
DD67	232.55289	41.999123	59.880238	17.515955	31.013666	55.82212	9.705092	162.21931
DD69	148.27287	19.604929	11.713882	27.698545	12.086821	144.18716	18.926905	91.24989
DD68	188.99533	31.37594	23.55262	17.700981	10.341064	108.51332	10.214508	164.81032
DD25	222.40807	14.728965	1.6200521	13.243192	64.8761	121.42122	3.2384512	5.3852143
DD28	156.36314	10.100742	14.320026	29.814543	16.46352	75.67885	15.246895	70.492455
DD29	164.78261	12.686551	1.9542868	19.721708	45.395866	78.06821	12.913546	9.580683
DD27	169.61317	85.97558	75.25761	28.488827	15.539544	308.6822	8.010607	239.11719
DD30	207.03583	7.746723	2.3290665	13.516159	75.38757	137.63013	4.012896	2.3328183

On-Line Supplementary Table 1

Animal	1379890_at	1379994_at	1380047_at	1380094_a_	1380123_at	1380165_at	1380167_at	1380180_at
DD81	266.15094	7.9683394	13.518465	151.39459	31.073708	78.83821	45.92523	61.906853
DD84	212.7737	8.3778305	3.9353528	119.61633	29.220657	75.467804	42.41777	52.086433
DD85	259.59396	5.6473684	7.869146	120.98786	29.684343	93.78928	31.498497	45.16723
DD86	726.9687	3.218743	5.1276083	109.32719	26.21052	86.570335	31.833014	12.647151
DD83	214.7393	9.470219	9.676078	103.33568	11.343607	79.19049	31.07641	47.387318
DD41	295.19315	5.5114865	13.919681	84.36013	49.12184	67.26335	65.52613	17.525364
DD42	300.99243	3.217625	8.743135	59.8659	29.84054	59.263885	150.27423	13.574964
DD43	315.17703	9.177954	9.669471	82.77385	33.121822	101.86108	118.20986	54.884407
DD44	226.14973	3.9142933	20.078154	106.21358	10.761457	88.103424	63.074745	39.266937
DD87	306.472	4.081264	17.844748	92.37586	23.482456	67.132195	60.432125	16.041395
DD2	258.07672	0.3865557	6.340075	105.40653	2.6558774	82.877174	82.08059	38.336945
DD3	481.4078	0.4818326	18.294535	92.221695	14.348982	73.26195	54.125607	20.583511
DD89	310.9448	7.6118965	18.424324	141.89722	8.930904	62.38332	44.033257	23.652533
DD90	523.0001	9.80994	12.955476	278.33234	20.387922	75.99715	44.03626	39.805286
DD4	423.4513	4.532348	19.172527	149.16365	13.538817	72.72027	41.526237	35.062325
DD47	380.1361	6.4575877	12.40114	101.21832	33.305946	47.426353	68.525955	22.840092
DD48	269.41714	1.0613847	12.440362	109.65366	15.401838	65.81198	67.62683	27.097391
DD49	655.42737	6.8989987	6.3229785	132.47542	6.424278	85.19285	59.41767	21.533138
DD50	589.1519	4.041112	14.011942	117.07939	6.182472	71.40188	50.24786	18.30383
DD46	378.82193	7.305668	11.544204	102.14521	13.466049	83.423645	48.56941	22.179197
DD6	274.0545	12.767554	6.8656807	119.3391	5.49445	87.26095	57.060623	45.901115
DD7	399.62344	6.1984015	10.855352	164.51717	13.014356	75.32473	33.254112	31.013817
DD8	471.33008	11.383187	11.849358	113.85343	12.256583	68.05062	39.84512	28.878618
DD5	385.0262	9.538768	17.079943	128.69865	16.070528	68.40636	24.552662	29.483833
DD9	410.49374	3.2871838	7.6900024	129.32489	11.990426	78.58296	36.523754	37.65327
DD103	107.65572	12.306978	15.827635	9.899291	44.471745	2.1609526	11.789725	41.837692
DD101	82.09339	9.632337	6.7165923	20.952845	16.918156	6.235278	7.2840962	70.31031
DD102	106.9701	8.670922	18.692305	11.762614	39.632214	1.7537652	4.879327	61.77693
DD105	119.49316	14.139418	18.5708	7.1543736	44.753937	44.18077	12.211106	84.848076
DD106	92.465385	14.265096	13.709699	13.2339	30.181253	13.297493	6.049372	65.278755
DD62	54.470078	7.219295	14.051358	9.68167	29.758327	16.34313	16.249172	40.48289
DD64	128.77756	13.642512	19.617601	9.935235	34.3355	51.553844	17.722841	52.70317
DD107	77.09127	21.55756	24.787264	14.29209	25.671864	5.067237	24.871658	69.21135
DD108	107.8763	17.669657	19.008924	7.953302	29.480211	2.1753435	25.710306	50.015835
DD63	118.58734	15.079108	23.091442	10.34785	32.982777	9.109113	19.575884	50.000324
DD23	145.02681	10.21769	27.09048	13.249493	26.337616	41.54172	6.63061	67.26744
DD24	126.90581	16.167034	21.230436	5.564157	15.840821	39.821033	23.141441	57.697323
DD110	145.71507	10.106897	38.95389	4.1372805	26.146969	12.156561	11.444457	54.781773
DD22	106.77789	8.144004	18.404514	8.376308	29.104805	8.429322	7.4442215	55.01567
DD109	153.1504	10.210946	37.408363	10.120918	40.59177	43.746456	10.404183	44.662773
DD65	183.98251	15.807016	56.92739	12.798576	57.924614	7.078444	19.689516	81.56472
DD66	109.86778	16.438614	33.37421	9.272022	41.439804	13.634954	53.069614	55.244385
DD67	76.101135	5.914347	10.112629	7.1092906	24.571852	78.44612	6.8294134	34.341022
DD69	114.15703	9.249759	51.39669	15.814636	47.24019	50.88985	22.036364	69.72518
DD68	105.08106	13.391505	33.638435	10.538996	20.754202	46.617386	15.984667	71.47899
DD25	186.86235	9.06888	25.707666	11.508841	105.52746	72.31145	39.460964	68.90204
DD28	266.08698	16.899088	22.864712	14.708984	46.58853	10.7182	5.081267	55.801926
DD29	162.84003	10.050128	23.450174	10.126996	80.816025	36.800495	20.77076	72.08345
DD27	104.48496	16.477087	54.67955	17.15052	36.67679	42.127552	24.62752	71.35673
DD30	163.33087	13.440912	32.355656	12.794211	108.1222	68.59055	33.550095	83.24164

On-Line Supplementary Table 1

Animal	1380209_at	1380264_at	1380365_at	1380407_at	1380418_at	1380433_at	1380531_at	1380632_at
DD81	1.7496107	5.1126375	14.671067	495.5676	34.55218	5.164336	12.785837	71.383484
DD84	5.3927474	6.2605467	15.788689	571.8886	37.456818	4.641401	22.131279	76.35998
DD85	9.024675	4.845281	14.266012	544.0044	34.80852	5.983472	10.71228	80.06891
DD86	2.57429	11.210011	23.265915	620.28827	55.64107	7.0009	13.099484	28.535284
DD83	14.477452	14.819656	13.751223	553.8269	36.488205	5.062176	17.07593	62.71037
DD41	39.468098	11.377824	26.291153	582.1303	48.045216	11.9343	4.601891	16.95069
DD42	53.36914	12.28754	43.9638	634.9617	54.988377	32.76981	16.23065	6.201479
DD43	17.949146	8.56985	20.009459	482.49045	43.090527	12.616179	18.6733	18.670282
DD44	18.781683	12.554391	22.171059	583.2602	33.56877	6.165774	15.203774	32.192116
DD87	35.406338	7.643551	42.361588	620.3	48.901253	9.762717	8.914371	15.91643
DD2	14.288829	9.716376	13.472934	440.67865	27.406067	13.250566	13.001643	43.05616
DD3	17.285025	18.685514	20.943403	584.52704	47.578224	5.708545	12.562333	19.439608
DD89	17.581959	2.4727883	14.800403	567.44183	45.80842	47.071213	19.289877	11.926855
DD90	11.916328	16.11149	10.438966	622.5663	60.371826	87.73181	18.308212	1.6260202
DD4	9.802794	5.2656918	21.845938	655.91437	55.225086	39.04975	18.815628	1.9862597
DD47	43.860847	9.859349	17.70078	605.4243	44.864506	25.023064	1.864021	10.055318
DD48	24.707525	1.8233678	14.618537	510.72153	41.491993	14.700693	19.180197	48.437767
DD49	21.72007	4.073132	14.875543	586.63214	60.865692	20.940237	16.793346	15.220035
DD50	15.15016	2.5431585	21.011223	537.70935	70.77725	34.87806	11.274776	27.668232
DD46	26.699558	10.962041	12.802629	566.4969	49.897465	15.885999	10.345845	16.99725
DD6	26.204401	14.824682	10.105655	478.53546	43.182213	27.416967	4.7791963	31.758049
DD7	21.998072	12.614636	9.699104	504.3697	59.053307	53.541748	6.477522	9.041265
DD8	24.26023	12.039613	20.332386	498.67062	49.27055	38.715034	7.150833	1.0120907
DD5	28.936642	2.1483598	18.739576	479.5488	54.268208	15.041309	13.977207	11.27438
DD9	25.57575	9.345586	15.497843	627.8224	59.76076	32.67793	12.8473	7.6130896
DD103	11.367468	17.437582	81.26041	245.57079	16.89443	27.065819	37.686428	5.89449
DD101	7.503114	8.60309	70.32263	286.91312	16.233896	24.732462	34.568207	69.278046
DD102	12.008615	18.520624	79.84817	259.78696	17.809	51.340454	22.83991	19.715023
DD105	12.339125	17.759365	69.681206	258.24283	8.412666	25.418709	13.104658	66.310585
DD106	15.004377	18.76636	52.709385	425.7398	25.445755	48.490654	33.46179	57.309963
DD62	25.997097	22.353737	64.599625	298.08807	10.477735	60.055954	30.51311	85.314354
DD64	23.726053	13.788053	63.027428	502.3723	22.936926	42.499893	29.424229	25.295918
DD107	26.88656	20.659338	55.387146	233.51573	21.480036	36.28807	15.017475	16.781565
DD108	15.173862	22.15666	85.369995	301.39404	33.95274	33.16932	48.223278	23.83577
DD63	23.377497	24.793348	74.211655	308.29425	28.193548	37.408016	48.634785	8.5786915
DD23	35.84844	27.628931	66.32192	446.80783	27.437328	66.65175	22.22871	8.446021
DD24	30.187687	28.866936	85.01759	278.9575	28.865543	51.598892	43.200535	14.023908
DD110	50.096115	35.06646	49.998665	277.87387	43.791496	39.00736	23.624249	30.593147
DD22	27.943928	31.019949	61.317127	317.6775	27.518177	46.407772	36.14976	21.902636
DD109	73.262505	25.981586	77.34184	558.2972	29.261015	22.869331	8.681729	1.9467472
DD65	34.96095	26.604546	73.33737	217.6714	22.109646	89.95048	13.27419	22.678865
DD66	57.309147	35.215454	83.28911	255.86855	33.626305	85.76755	26.537745	6.1595693
DD67	44.955708	14.138061	63.33544	458.4544	21.175436	79.99217	20.739815	14.345587
DD69	43.033077	19.223587	62.471523	243.16841	25.984478	63.697792	31.856373	4.1599393
DD68	67.15948	17.613539	61.56642	489.86737	38.357014	103.43423	18.567179	3.5247676
DD25	55.98112	31.732906	75.6092	458.8141	38.49491	35.063274	16.922401	2.6073446
DD28	50.663055	25.047379	67.31494	471.7174	43.42342	118.16025	20.53755	6.613292
DD29	53.14919	24.881018	102.62927	308.04382	25.566729	103.90939	37.01222	2.387689
DD27	37.771313	39.980118	42.857285	411.79086	32.45383	65.216576	27.48454	15.025774
DD30	48.058594	28.398252	75.446	453.18134	51.882	29.452171	3.0718338	2.8850293

On-Line Supplementary Table 1

Animal	1380651_at	1380670_at	1380701_at	1380782_at	1380804_at	1380835_at	1380880_at	1380956_at
DD81	11.109551	27.82902	67.44545	9.027239	34.480343	7.70153	53.999855	5.761428
DD84	21.658203	15.709728	64.520004	2.7773948	26.20688	31.0755	40.747635	15.666629
DD85	5.9314885	4.823118	76.43383	15.690584	36.302135	9.574965	55.214638	25.05863
DD86	17.154255	27.319494	72.37514	23.344732	23.517735	13.195299	98.410194	23.556568
DD83	11.834753	17.468868	65.90656	12.637644	18.128944	10.700822	75.816055	13.524632
DD41	21.910034	5.390009	43.454464	21.303461	20.559767	16.77435	30.413683	19.330328
DD42	19.83681	16.208601	43.721474	16.522974	25.623108	11.058555	20.621765	22.844814
DD43	26.17666	33.67818	65.55041	19.235403	15.737206	20.442614	27.319733	9.383684
DD44	22.815968	30.695356	51.367992	14.535332	19.22034	15.714503	31.998032	10.629011
DD87	25.786568	12.181796	54.0429	12.697925	25.424902	18.51768	47.30045	2.6228542
DD2	13.163697	16.466852	57.55405	11.937555	13.184359	4.356226	24.193579	3.6029189
DD3	27.311798	13.295941	53.5415	12.413694	25.402958	20.337923	31.160131	11.837338
DD89	0.6801447	31.823309	82.917114	12.401722	34.997673	7.476212	39.27026	6.256071
DD90	1.5046214	22.704836	78.81298	12.681067	26.971972	7.288	142.8594	24.368017
DD4	0.5172142	11.978905	56.514736	16.758993	17.95635	16.721727	307.29514	7.1403804
DD47	9.964245	9.112156	34.535927	13.957548	31.63252	18.904453	55.525696	1.7822723
DD48	19.774803	17.203348	66.60195	25.837683	27.250418	15.633919	55.878212	8.888534
DD49	14.083025	9.250646	41.36424	6.396054	34.100937	12.159923	140.0957	21.494648
DD50	16.103432	30.913622	71.266785	3.770451	9.050051	4.779073	138.24846	4.0524507
DD46	19.577213	23.392658	61.260006	7.740022	18.032358	6.415254	188.49878	16.37648
DD6	15.169222	27.288483	45.907757	20.996305	16.279417	10.961534	200.91815	19.045132
DD7	5.779364	28.905333	47.765594	17.542082	23.01425	1.541064	145.2937	9.758621
DD8	7.3020163	14.567225	42.290478	20.842234	17.860619	2.068273	175.83647	11.339013
DD5	6.186767	3.1950607	56.86888	17.636847	14.598004	3.6067352	128.81883	2.5744982
DD9	8.672494	13.262612	26.28292	10.050333	31.682934	1.6348469	86.07586	8.71834
DD103	48.306072	35.711086	102.21712	48.550346	27.62353	22.73341	6.0800204	24.04979
DD101	104.67741	30.437807	128.32281	20.571144	45.25444	25.900482	8.363686	16.082289
DD102	55.66217	61.327637	80.34133	43.206825	27.572205	39.532536	4.809251	21.981949
DD105	58.77203	37.023453	109.76964	40.981922	28.786478	27.355673	6.0836897	24.51127
DD106	44.004032	24.680237	171.5715	16.222263	39.853863	29.032883	3.4534712	11.292109
DD62	141.95087	35.08256	107.086	46.057896	22.102627	17.745184	7.365265	53.610664
DD64	102.13468	45.53007	103.91832	20.959133	35.633144	17.151785	20.351057	59.695477
DD107	59.60882	82.46396	112.33141	32.417675	22.501574	20.798302	9.619321	39.547707
DD108	48.990242	63.94943	102.10606	32.660583	24.587671	32.12215	14.088317	31.047453
DD63	55.223354	47.8445	99.82213	23.253235	30.001333	27.124893	7.4188204	21.684536
DD23	77.03414	56.417854	103.86511	28.920185	39.89781	34.46234	9.209587	57.420593
DD24	80.946045	50.863277	93.6679	28.758734	65.20802	28.33506	24.337809	76.97672
DD110	30.796274	65.02789	172.1948	28.119719	54.04726	15.074348	17.398481	96.85982
DD22	92.370285	39.493507	122.20855	26.654903	77.73394	18.061367	16.81866	111.64796
DD109	36.61847	69.93196	106.47196	27.225449	37.95197	19.114035	9.124029	66.546005
DD65	62.651894	47.931072	149.47925	36.543392	76.306694	25.807398	20.237083	113.90721
DD66	23.646673	31.9648	74.00578	39.015743	81.81286	6.0727615	10.178237	43.35281
DD67	71.73886	41.280533	71.109085	19.843187	80.05592	11.26735	16.847004	26.28617
DD69	95.0822	74.85921	65.649796	43.969074	89.53717	19.228275	26.670746	31.297705
DD68	99.765045	67.67581	90.0787	20.888927	167.12761	31.322817	17.53995	50.697872
DD25	14.600825	46.535545	119.97178	27.286596	53.75705	21.284512	27.3786	48.03872
DD28	93.549675	49.368885	52.61027	31.880861	74.2499	16.519117	46.339386	3.3697011
DD29	104.68504	53.17484	117.48059	26.826323	100.92532	8.619874	38.27972	84.36957
DD27	17.952446	66.327736	149.25764	16.144869	46.02293	27.588917	16.059067	65.7488
DD30	8.884704	55.157948	122.7691	24.157864	53.483646	21.312834	17.9351	51.801945

On-Line Supplementary Table 1

Animal	1381166_at	1381218_at	1381327_a_	1381328_at	1381386_at	1381407_at	1381414_at	1381503_at
DD81	5.482881	34.278885	79.59333	30.159124	18.647816	29.498793	50.285767	44.270714
DD84	11.540105	17.832577	52.440083	31.904726	11.10119	27.293764	59.182003	37.87864
DD85	16.179092	28.926985	2.53103	6.688246	15.314143	29.910673	60.310936	33.52385
DD86	9.119807	28.8919	4.0492334	0.0828424	11.28996	52.64328	67.50652	42.903076
DD83	1.3006485	21.72765	22.620665	12.339249	11.262363	20.413456	60.698296	35.61026
DD41	11.323699	26.27588	0.5809293	3.7157776	11.931787	27.362179	73.38292	30.980244
DD42	9.110862	20.82984	0.2125229	1.9937389	2.3748722	19.046162	46.709255	34.91039
DD43	9.643717	30.784052	367.50015	200.60674	7.2721457	42.454704	99.159355	27.15584
DD44	2.9275591	38.409706	239.85713	124.90151	12.936573	18.477196	78.53945	36.989597
DD87	3.0295012	26.189833	1.8441513	4.187878	15.242118	30.622566	54.961792	31.414856
DD2	10.239922	17.17757	234.09761	61.501945	7.5042267	25.89819	75.47567	29.022392
DD3	12.073731	25.261757	8.785153	4.24927	8.966627	25.406784	63.031357	21.017138
DD89	0.9519455	25.78653	8.796907	9.279542	12.093907	24.76037	59.136864	27.382608
DD90	15.169866	27.273842	8.966405	5.235152	16.487463	36.51767	94.93445	24.732916
DD4	6.4567876	37.971115	1.8548193	0.2379751	13.016808	43.3408	72.03384	15.765439
DD47	5.314415	20.715597	0.956185	0.5860874	9.087222	23.887394	54.730446	33.694653
DD48	7.9111266	25.724674	0.3289851	3.120988	6.9760666	32.766953	98.684715	35.82342
DD49	0.6285997	24.154587	1.9583668	5.6953745	3.7942963	55.912903	61.78096	29.412155
DD50	6.738114	22.17569	8.522388	2.4659493	7.6549344	44.16109	58.988865	31.778814
DD46	14.714959	19.928902	0.741558	3.9123545	4.6978354	41.73924	84.782	27.636442
DD6	19.05105	25.275045	155.0795	98.58933	3.9414525	46.857452	97.736176	31.603214
DD7	12.948133	23.189554	100.29336	64.19373	5.2413235	38.638035	93.66775	21.476377
DD8	18.209297	26.41698	2.188058	1.5433054	11.020862	32.953377	69.145454	16.153246
DD5	8.532827	24.375832	16.767231	3.371933	7.573519	31.68292	40.702415	2.684552
DD9	10.623297	19.473295	15.491671	8.4900055	9.556525	35.393314	55.423775	12.85551
DD103	8.537241	129.63138	40.428814	36.394806	31.836866	3.7376056	24.183735	62.070217
DD101	6.656771	59.692425	18.063343	21.301268	50.96776	14.046931	27.172783	92.52875
DD102	0.9351669	78.18744	166.90627	85.931	37.367218	5.846229	18.492188	70.877
DD105	10.654848	87.081375	12.467104	7.6942296	39.137974	15.906126	22.06462	105.94342
DD106	5.8956876	46.416378	40.95125	23.454514	39.24958	16.294119	28.634457	67.73977
DD62	4.499256	71.18154	29.831022	14.011817	33.340725	32.331715	31.21801	74.904526
DD64	2.1225812	95.533356	104.83089	44.287334	10.883711	14.007362	34.075947	70.66454
DD107	9.667293	80.642395	407.33994	170.22075	65.101	2.1941147	35.088284	59.18564
DD108	1.2590567	109.34265	146.79158	76.90212	48.16813	2.9359205	39.764286	64.02709
DD63	5.0051203	120.10406	152.07895	55.436405	54.35509	1.1789663	18.22843	73.47561
DD23	8.170747	88.722824	75.044716	44.953953	37.308765	39.02677	24.117733	40.74036
DD24	3.5217001	75.99936	23.565943	12.26362	48.199512	27.899399	33.105824	38.048733
DD110	1.4034951	131.87866	9.414199	3.5182018	41.44662	19.496202	23.585396	43.10203
DD22	6.3978686	63.18686	0.286092	1.033113	28.925835	11.54006	19.885366	49.089825
DD109	1.2151672	76.42138	3.551355	2.1499624	63.18713	39.165646	9.442034	55.653587
DD65	10.199776	99.03509	16.767534	8.943074	7.814898	14.572899	24.258024	42.318512
DD66	14.525972	97.62857	6.0944643	7.621929	38.342133	20.16733	19.685349	60.37178
DD67	10.855735	76.326515	0.1471538	2.046882	59.30506	16.479046	16.090395	57.206238
DD69	3.5976171	134.06195	28.181526	9.809047	10.699773	26.599297	28.42813	56.579285
DD68	1.0808418	142.11676	47.536236	21.239763	32.466778	24.376274	26.229652	54.42733
DD25	7.37635	92.33799	18.510057	2.9106133	44.41282	39.204227	29.646688	72.52996
DD28	10.77141	117.30181	3.9294007	2.9181004	34.92934	3.166802	35.62489	74.72283
DD29	2.8762293	123.85294	3.2280738	0.3020214	39.271202	2.5083206	30.066387	89.59998
DD27	2.3903952	31.879177	248.1731	108.35451	10.016034	45.057808	41.052597	81.434105
DD30	10.899961	121.83976	5.823593	8.100742	39.719624	39.451954	20.977093	59.651623

On-Line Supplementary Table 1

Animal	1381508_at	1381574_at	1381590_at	1381620_at	1381626_at	1381747_at	1381795_at	1381990_at
DD81	27.352324	317.2849	36.70092	6.3587623	24.22457	18.751179	22.946272	169.55421
DD84	21.054731	337.799	17.022831	13.162776	31.210094	16.789288	32.34009	152.7359
DD85	15.999145	369.89847	36.719803	3.3614335	39.598755	10.302308	34.90233	123.03928
DD86	12.421844	578.1279	38.087776	1.992927	34.27198	26.511963	11.257291	139.27583
DD83	17.717789	338.53845	23.351158	5.835768	30.12851	14.098858	20.0608	116.36198
DD41	23.464352	591.03094	10.81997	18.403128	29.255548	24.048744	20.841444	176.23283
DD42	22.340677	524.66315	24.432611	6.5704966	40.05381	16.930424	13.056372	131.57619
DD43	15.379465	435.94113	31.44488	2.9450119	18.787996	6.71197	9.383333	159.212
DD44	16.547188	529.43097	22.118309	6.349111	40.5757	6.433049	4.0492897	154.0005
DD87	19.315783	584.45374	25.528751	2.4027214	27.802603	19.34651	2.9455564	120.35117
DD2	12.123848	618.8478	17.517677	4.8591514	19.934011	7.5128183	22.929531	108.84072
DD3	17.545992	823.8355	17.381384	6.022383	31.248096	11.200994	11.827975	110.40201
DD89	13.942255	870.77954	24.34536	11.53827	26.23863	15.727831	17.19572	246.30588
DD90	14.520741	1094.8718	22.664335	12.859751	31.958792	21.466394	19.10047	272.37726
DD4	13.51246	970.3679	8.901511	7.653878	23.679762	6.6046	10.956806	172.64406
DD47	17.125402	776.7372	23.257689	11.332446	18.119192	11.466285	5.8104486	111.58392
DD48	11.817143	653.4623	25.945097	11.871996	27.737272	27.02775	29.294628	151.5853
DD49	11.577781	857.2899	20.439135	7.2379274	22.11284	38.24515	9.756196	139.12508
DD50	11.359251	748.14386	31.083403	5.3633738	23.784286	46.283764	24.888494	172.50731
DD46	7.9292645	901.4732	30.300798	8.11645	22.10364	17.223219	11.631186	123.43039
DD6	18.20847	726.9568	19.36226	11.128886	20.912247	20.836306	30.351252	161.24182
DD7	12.50821	929.6093	20.911356	9.894869	30.836876	21.498116	33.84633	197.46817
DD8	10.331909	1110.508	16.93041	10.362405	22.625677	21.956156	20.458221	182.60019
DD5	20.054544	1023.4498	27.131638	7.5829034	23.54637	17.77072	22.279913	130.39174
DD9	25.347227	824.0274	20.756735	2.1500685	21.226774	19.186197	14.428657	118.96715
DD103	54.016846	81.16743	59.930893	0.8688897	14.965277	13.505321	17.00083	110.19007
DD101	84.015564	55.716805	46.87511	5.854327	18.512865	0.5175787	19.655817	74.939644
DD102	70.20938	89.15289	53.036465	0.3348173	14.885976	9.429221	24.500206	52.241
DD105	88.89062	67.85785	62.61883	11.250844	12.617696	11.322321	24.847488	53.817398
DD106	77.199234	80.14011	45.14345	1.1576772	8.836766	7.3949423	26.21011	80.618576
DD62	10.668504	452.35745	34.775246	1.0098277	4.7931886	8.387719	20.697594	58.942814
DD64	49.934685	195.24414	39.40235	0.8504978	0.672111	16.889936	11.239169	79.9201
DD107	48.41718	584.08386	37.627914	2.550704	4.5439277	8.962978	12.040994	70.86242
DD108	40.67019	244.14041	40.300827	0.8433908	9.21735	3.274791	5.590964	67.64529
DD63	41.918686	217.68661	47.024467	7.7450733	11.321533	7.955058	21.680058	66.98109
DD23	28.110456	174.74706	35.886196	10.431901	3.8509848	5.2455096	2.043714	64.42523
DD24	61.180096	164.67967	36.571625	8.839432	7.8023024	5.6900673	1.9240822	79.427666
DD110	38.997074	446.17896	52.86322	0.9161767	15.392774	12.110699	12.400615	54.87688
DD22	65.17594	185.7412	35.443592	11.059652	10.028647	17.055374	1.4717786	76.25807
DD109	44.096176	217.57997	51.043106	2.6052747	0.5764744	14.821459	1.5788465	32.44618
DD65	50.88164	167.85736	52.385033	0.6430005	1.7056912	21.564997	1.8405119	45.92654
DD66	67.99353	544.3301	34.75607	1.0078261	12.965095	9.615751	10.837013	130.94472
DD67	43.160046	191.32301	38.99833	4.0387263	1.0791632	5.321224	5.624956	84.4123
DD69	42.24032	168.44989	60.66541	2.1190097	8.74956	13.80351	5.964943	93.424065
DD68	33.147366	607.9123	52.701427	0.4972437	8.863064	12.573611	1.3979703	113.52725
DD25	61.603016	194.98497	57.49093	1.1523545	6.776746	14.491267	9.645596	52.49121
DD28	35.73462	223.62958	49.699	0.9566517	0.4415899	11.78732	16.17017	94.43636
DD29	39.520737	382.62982	55.22154	5.7095838	3.1288626	8.250374	12.356529	85.68482
DD27	10.895127	117.64221	32.101917	0.6911087	8.007876	2.1221962	1.058379	63.31802
DD30	57.873535	185.78282	41.82288	7.862712	6.6129684	19.722242	4.3852243	43.769554

On-Line Supplementary Table 1

Animal	1382163_at	1382171_at	1382205_at	1382291_at	1382368_at	1382389_at	1382401_at	1382431_at
DD81	16.448118	433.07538	117.17185	2.9241786	206.25998	1.8650762	318.04633	330.16525
DD84	26.581718	338.34317	104.94379	2.3910866	191.02466	11.094681	301.40195	330.83612
DD85	2.3038096	368.49237	82.035736	17.618298	178.68529	11.894051	272.16965	304.17575
DD86	10.895188	422.0866	2.165179	2.5057755	209.62914	21.586515	34.983154	327.3316
DD83	8.780077	364.02148	96.85752	3.2603583	173.27419	1.3824726	279.1461	257.81485
DD41	24.993643	399.01874	26.454138	2.2801335	271.35297	4.7946873	54.72539	539.3096
DD42	25.40464	343.49893	15.884725	1.1022989	210.50203	12.109839	39.259514	763.2181
DD43	92.74797	356.61984	8.5608	7.727951	192.46213	15.953797	58.745464	467.32816
DD44	45.359367	444.3986	20.006855	4.275584	177.68837	5.3184266	50.26866	501.27798
DD87	44.2786	373.85635	16.114687	5.064946	213.06032	12.946183	27.719837	469.19034
DD2	34.02507	413.59427	80.97122	5.882101	196.47641	16.440895	99.83903	460.67126
DD3	14.766943	388.06204	18.274	0.4937312	253.84207	8.284904	36.940254	233.91254
DD89	9.089101	597.20605	21.677042	1.8181248	418.566	15.989927	52.75393	289.6499
DD90	56.504993	980.84326	4.77103	1.4676106	397.7217	23.12574	13.398527	378.72012
DD4	16.088642	700.5948	8.938725	1.8048614	407.5013	4.4855876	4.408647	312.56665
DD47	34.628613	310.3648	6.8927426	1.9716529	133.04588	26.215248	2.9772549	522.00696
DD48	14.975139	422.05902	115.67679	1.2043904	334.2703	11.808891	141.39899	279.58954
DD49	18.237722	520.1726	20.871664	6.2274113	277.2475	21.102428	38.33308	381.7739
DD50	58.208385	379.09436	0.8234934	2.1440687	353.9769	13.359472	13.048743	324.35504
DD46	7.653417	454.81638	17.065182	1.6614977	344.40585	12.582949	31.178894	340.23117
DD6	21.601786	481.5106	68.86346	4.045339	289.45984	17.141014	57.2556	391.3716
DD7	21.148514	604.28156	13.867766	3.6551616	331.9456	13.960728	19.533072	419.93387
DD8	8.999616	468.48538	2.7748828	5.793734	349.25778	1.6918603	2.351877	421.459
DD5	12.351481	347.6917	5.7172127	3.3300335	138.81358	1.5107018	14.788429	391.0282
DD9	22.335073	430.75424	7.236951	1.8975325	222.4536	10.191162	9.962054	398.1055
DD103	25.200733	234.11421	27.304256	15.198753	102.77486	1.7402556	81.39803	75.18008
DD101	13.242695	423.3252	84.769905	20.006502	53.8662	8.5936575	160.26424	58.65672
DD102	26.811453	353.17313	47.446865	21.081215	50.171276	5.1760974	94.84209	115.79257
DD105	7.264065	245.8954	97.47743	16.156067	75.885185	3.0789442	206.65164	57.083157
DD106	20.249357	286.73267	97.897415	9.249005	132.26907	4.4153023	138.84277	82.72442
DD62	23.824287	268.91452	67.54021	12.157132	98.90418	10.440431	114.40274	50.5498
DD64	34.82096	274.6731	42.533974	29.385277	85.228836	8.697116	66.98465	56.380398
DD107	59.641003	114.57365	10.986536	11.670992	172.33125	11.911028	22.962845	82.28459
DD108	69.586395	347.2505	7.548495	12.796398	105.48734	15.920609	22.94538	93.69082
DD63	99.30538	343.01483	12.356993	7.4384217	105.276	2.6140888	35.194916	86.75203
DD23	30.084208	114.46542	0.7058505	5.124383	145.20015	5.948015	5.0285625	109.69762
DD24	27.00767	231.90045	3.1571212	12.595053	214.08894	6.7813487	16.431217	96.977455
DD110	56.15472	222.67767	28.015003	15.964822	118.72825	14.097574	31.17928	80.26396
DD22	23.62095	195.03926	19.827667	0.9256596	146.94627	10.302881	20.618734	75.875946
DD109	26.186272	121.57961	5.585549	10.686419	184.94427	3.473713	2.9758391	99.41271
DD65	72.63726	305.43536	4.8373704	10.497466	222.52074	9.884951	3.8452244	66.75631
DD66	54.95315	137.46523	4.333931	13.634333	244.4351	18.650276	3.9506109	88.85371
DD67	11.779861	158.22534	9.652577	9.817379	163.68544	0.7355698	12.8691	101.61039
DD69	73.659935	144.21776	7.827137	13.412157	136.23425	11.819778	1.0623885	96.06514
DD68	22.050283	434.71817	12.536173	10.913204	327.2481	3.378199	3.2238128	85.543564
DD25	42.081085	123.7575	0.5320889	11.031177	77.63011	5.95442	2.5272179	164.70238
DD28	102.6909	532.9192	0.8453097	13.742356	249.73955	22.038433	1.1110386	68.37942
DD29	112.60396	215.38922	0.3532263	10.355516	152.5811	32.91867	0.9259735	101.32713
DD27	24.37602	184.82118	15.483789	10.744483	226.95859	17.385956	18.219936	144.8515
DD30	47.39523	139.7443	0.3648628	6.841244	69.46215	16.628002	1.2162216	210.1619

On-Line Supplementary Table 1

Animal	1382437_at	1382462_at	1382467_at	1382524_at	1382539_at	1382618_at	1382648_at	1382678_at
DD81	1.4571129	303.10345	16.650671	60.56428	22.328152	36.195564	28.031908	50.646866
DD84	6.8171167	328.07593	18.445532	60.422634	21.48321	51.12633	15.435369	4.271509
DD85	2.7092984	319.23413	22.6484	53.667477	22.361586	50.04763	30.145742	12.101614
DD86	16.35803	370.0286	21.697876	58.56077	18.534071	75.97159	41.266663	2.1546946
DD83	2.0072644	312.22318	12.022584	63.14072	18.909925	40.92459	32.495922	11.177495
DD41	7.873515	294.69208	40.38332	59.049152	15.753402	109.99561	32.606598	7.0067964
DD42	2.3375235	292.887	48.93665	55.592583	26.152466	79.31273	33.040802	13.132651
DD43	17.190376	233.24666	6.381911	57.84033	10.071972	98.48941	29.727312	20.165934
DD44	2.1709132	261.7655	15.190329	53.537994	12.029929	51.72066	18.530895	1.0054475
DD87	4.135189	306.13	34.424236	59.953197	16.036835	87.22126	26.57224	5.1577826
DD2	1.4017525	131.15448	8.661305	34.575806	16.982422	45.24172	22.332006	7.1751595
DD3	2.0086145	170.38359	9.834822	51.947567	9.385459	60.37266	22.024717	15.486924
DD89	1.5332648	146.97458	10.397407	37.482098	20.997997	65.9941	8.151684	11.676875
DD90	1.7180517	129.50269	12.529864	31.22138	27.255287	51.334175	24.160877	1.0392191
DD4	2.7826319	117.96429	15.741629	24.306763	21.9554	183.62546	10.536209	1.5066516
DD47	2.8420734	138.55872	43.105545	45.421185	12.61344	31.115223	19.777193	8.350158
DD48	10.78201	113.29057	10.746494	60.151806	18.660425	42.489643	37.3284	52.499744
DD49	5.632961	86.851944	10.043876	47.896076	11.207281	34.429173	34.92531	4.646404
DD50	5.9782734	108.13962	9.289443	58.43398	6.045709	35.32572	26.07119	2.4713225
DD46	1.7268156	180.88402	13.57856	66.509575	18.11896	34.531136	26.662643	13.654304
DD6	1.6606569	88.60341	14.324109	54.19832	35.28388	8.265041	21.230793	4.028605
DD7	0.9465383	90.97761	19.070189	46.888916	31.26141	46.455544	21.814283	4.4088044
DD8	3.2078676	106.46624	9.331018	37.725758	23.752996	44.6406	13.574087	2.7841873
DD5	15.098919	103.90461	14.573793	15.879183	26.427666	61.105747	17.0142	6.138942
DD9	2.3798273	97.684906	9.051336	15.796566	22.289984	117.41467	22.823114	4.687117
DD103	24.580717	862.0639	5.050516	85.053154	12.210335	72.68873	13.719763	1.044087
DD101	12.254751	643.5778	8.914701	112.51985	9.071438	33.647503	1.0997046	8.339776
DD102	54.3836	518.74274	9.288165	105.11035	6.1595926	26.516994	5.658768	16.475697
DD105	18.463953	484.7011	9.842547	118.31046	14.419399	47.55782	4.3321996	3.311171
DD106	3.4427173	454.40283	10.315118	138.04803	8.670673	32.49413	4.0614657	9.208792
DD62	34.88928	233.4815	7.3082404	102.82573	16.685902	18.689453	2.7897897	1.3728858
DD64	38.869225	302.03	6.5476985	57.603886	13.865454	19.768312	2.9927168	1.1860485
DD107	60.2344	177.67096	11.101166	143.43771	12.724249	29.433048	9.993584	2.0999355
DD108	27.433268	403.4314	10.04495	161.86519	5.9843264	30.978333	10.494841	1.3320503
DD63	52.14811	409.47885	14.18924	156.38466	5.9936247	19.990166	10.145078	0.5935645
DD23	27.344812	303.346	11.662492	109.58855	10.19766	20.175583	5.6089907	2.7250628
DD24	54.803173	666.34924	0.8362114	182.28375	17.164116	22.24377	6.8028407	1.3306551
DD110	58.807514	296.6073	3.535712	121.76511	9.7017975	7.319151	9.312378	1.5041794
DD22	28.49579	386.76758	4.353336	169.66455	3.1388109	1.3494496	9.231849	0.271768
DD109	40.64257	336.83157	3.511886	75.66033	10.207208	18.193695	9.917796	6.443246
DD65	62.89732	290.80173	6.99221	69.04453	0.8172514	22.931408	12.570135	0.9189845
DD66	35.29347	457.4823	60.046158	127.32027	2.977927	15.768566	8.876796	0.6370922
DD67	37.699852	1106.8411	10.410674	70.61123	3.2234256	10.833024	10.321543	84.485176
DD69	69.81639	311.5479	6.4897017	64.48873	4.996297	22.396246	9.370989	1.2500125
DD68	68.62337	374.90054	4.7963786	58.504414	13.646255	24.470867	7.533812	5.376213
DD25	48.694283	203.4631	33.880642	40.907402	8.912686	5.04876	12.908645	0.1284479
DD28	34.080406	200.20705	2.1747174	126.25463	13.162762	22.842268	3.1290843	0.8441508
DD29	36.756657	337.59283	25.920647	139.0697	12.057162	3.6039684	5.2578287	0.6696315
DD27	8.298435	255.02289	32.026154	53.43963	7.2925577	19.367765	2.6400752	0.5648001
DD30	46.40923	259.7159	21.009274	46.799324	4.615652	11.94611	10.472934	0.0872739

On-Line Supplementary Table 1

Animal	1382778_at	1382809_at	1382848_at	1382907_at	1382926_s_	1382936_at	1382950_at	1382966_at
DD81	378.4768	26.882025	12.072198	171.66719	13.796774	89.50842	204.26817	3.7212157
DD84	378.7568	41.659786	8.241761	226.59152	3.4371006	124.45155	233.13972	3.2005124
DD85	413.89813	8.336936	10.590804	270.5351	10.894903	121.85567	203.4439	14.149485
DD86	337.94037	23.8417	7.731932	474.3134	6.082965	126.15472	253.24626	2.2821863
DD83	252.6367	1.8574666	0.812849	176.49779	22.469347	119.02271	203.00461	2.5305445
DD41	341.7716	20.027431	4.6161385	395.1079	19.416311	129.56818	658.2736	201.40356
DD42	239.83603	23.844706	7.5386715	553.5858	5.704825	133.08998	435.45856	411.16632
DD43	347.6722	23.432356	0.9400568	495.36664	66.00313	117.28832	598.9933	17.458656
DD44	511.71506	15.986155	4.551515	215.32838	57.351604	98.01062	615.645	4.179281
DD87	394.6602	36.91746	4.825397	452.46854	22.653044	129.9816	437.78384	190.30222
DD2	534.035	18.322115	7.5912995	372.51688	34.10557	121.74137	435.54022	83.31369
DD3	287.8652	24.62942	6.9936647	337.56827	26.30747	115.14125	727.7877	2.0076354
DD89	405.87564	0.9396349	0.7694005	252.47464	19.128397	122.27705	495.97397	16.140488
DD90	766.4468	7.3543854	47.158455	356.39685	22.069925	124.85337	425.1949	15.67865
DD4	524.5664	14.610398	3.7851381	931.78815	22.382883	181.19124	416.62628	6.6081176
DD47	218.13557	40.453247	13.033146	274.79547	20.38491	124.23308	351.46167	277.3962
DD48	365.97952	42.932613	9.572561	229.76527	17.763748	217.89154	348.91678	101.31228
DD49	335.9071	45.935	5.868965	298.80334	17.857811	176.12132	709.54675	3.6445668
DD50	356.8106	43.714317	15.759988	424.61267	24.306744	239.90636	653.01465	12.847805
DD46	370.54556	31.281094	6.6498833	166.08795	20.491821	190.79857	546.97705	7.0893974
DD6	447.8191	18.317877	6.7136765	154.59566	20.559801	194.40512	482.19092	16.08777
DD7	552.2976	1.0150816	7.1405535	217.34068	24.882427	202.83344	359.3253	1.6643463
DD8	389.72107	27.611336	2.4089277	251.31685	10.146545	157.4485	592.76044	3.3298285
DD5	417.88046	27.81805	3.0378873	241.25842	15.135568	121.64629	617.77106	4.73364
DD9	507.66403	17.22471	4.39174	444.84222	26.002628	179.65347	651.13873	15.483695
DD103	181.66147	65.775566	2.845012	363.9747	64.68645	71.42003	373.37103	5.326304
DD101	433.733	61.564663	0.5838519	217.60785	24.47812	35.56375	256.54114	14.641686
DD102	185.99648	83.64378	3.4200618	161.12991	142.12688	80.064926	381.73297	2.062813
DD105	145.71805	39.03465	7.2659917	294.15826	29.40779	71.780235	184.02985	3.9338303
DD106	211.15826	110.00826	8.780035	273.31107	60.961597	72.07401	205.63274	8.161843
DD62	162.39256	28.013716	18.128984	130.01651	82.58862	52.14745	1068.0336	3.8814778
DD64	171.371	41.840015	32.19995	269.91965	130.06407	61.689213	1437.5657	18.568525
DD107	270.3398	19.272768	28.990816	321.39395	204.64839	76.353134	1543.1147	2.2991233
DD108	189.82892	29.67476	49.006016	197.6592	162.96748	72.64098	1105.009	4.305646
DD63	139.4809	28.483887	34.43507	210.16454	157.08214	80.97272	1104.8997	8.86259
DD23	77.96181	94.32382	19.210709	120.35895	99.18162	65.58459	1250.26	2.49908
DD24	184.78798	38.335136	19.246655	110.12744	123.6056	65.869804	1389.5013	10.408944
DD110	231.19627	42.941566	45.06425	130.1685	100.09254	91.66223	1879.93	3.2375388
DD22	187.44753	48.29012	31.086098	82.247116	119.01161	42.55424	1358.0613	2.5060015
DD109	164.81854	13.524332	21.371334	132.90123	75.36328	90.72405	1560.7954	3.716949
DD65	289.09818	18.442764	56.836212	166.62437	151.69313	103.19122	1216.266	1.0085161
DD66	151.63316	24.065388	1.5859969	152.49861	172.97762	56.249615	960.52704	166.6676
DD67	63.375813	25.867147	14.651654	53.669052	15.129037	63.893925	982.3609	3.5045507
DD69	146.02734	36.26407	54.153976	136.326	171.59756	84.99355	1223.1803	15.815143
DD68	140.89177	57.444664	8.298059	156.96838	90.19143	39.65215	1456.3015	2.6262195
DD25	236.01468	15.633816	8.30698	188.47719	78.07016	96.28003	1176.0068	205.52264
DD28	66.11498	28.89028	87.70991	265.04193	63.84082	78.37152	1032.8303	3.09258
DD29	262.20004	39.165466	84.38117	214.68217	131.04587	100.57613	662.64197	84.72592
DD27	154.77863	58.453125	9.853763	327.34293	122.93407	58.659164	1040.4777	189.50992
DD30	194.59215	3.660551	2.9239614	198.8922	88.54185	114.61947	1172.0568	223.77083

On-Line Supplementary Table 1

Animal	1382984_at	1382987_at	1382999_at	1383058_at	1383117_at	1383163_at	1383211_at	1383272_at
DD81	35.447395	13.139798	19.11922	81.758896	413.50363	71.66538	23.201973	160.43433
DD84	54.306847	7.2626715	2.7566469	59.794228	565.1714	87.75528	19.020985	133.60254
DD85	35.25043	18.94354	1.7815773	52.08068	546.227	32.203804	15.813625	145.2329
DD86	50.52601	20.842676	0.7126713	29.878178	402.72415	16.134983	17.986454	2.1676896
DD83	46.052223	3.2039764	0.7464922	46.717567	389.34277	22.692846	20.48541	151.79323
DD41	36.67474	13.041579	1.5047971	28.261497	284.99527	35.815704	510.1731	124.11039
DD42	54.364464	24.670609	4.19008	27.18323	280.77542	9.595967	892.7654	40.428097
DD43	57.158054	42.11981	79.19901	52.104294	328.1481	1521.1875	22.46749	40.599926
DD44	48.89172	18.712317	56.56526	33.638176	283.78036	651.29047	15.985156	51.673244
DD87	54.1017	25.547937	4.294128	44.69178	321.2692	75.52103	507.3014	46.74091
DD2	34.436665	18.433266	62.47318	60.54945	330.88855	864.8946	117.09009	232.11493
DD3	37.711304	15.283725	9.194295	31.768118	336.0139	72.48537	19.521805	66.590675
DD89	24.56174	27.233322	14.495765	27.974716	315.82327	57.197437	18.756063	80.67323
DD90	21.182207	40.461323	0.7647196	42.49385	330.47556	60.106087	20.717262	2.3269114
DD4	24.791979	22.133455	14.770697	38.826767	236.69565	71.850006	27.965178	50.725155
DD47	38.678017	7.5137463	0.6260203	71.76013	291.52307	61.515007	613.1573	26.804716
DD48	36.15504	10.255084	0.9313016	102.3805	442.8265	40.929485	229.00652	218.50832
DD49	40.72941	1.8481896	3.96706	58.909763	375.85657	235.58804	11.66426	49.330242
DD50	47.322735	6.28117	1.7200634	54.521915	331.9943	89.7338	23.583673	26.408382
DD46	49.691936	17.825365	1.1538656	51.53733	305.20224	55.65365	22.180826	104.59076
DD6	32.167133	16.114801	49.249607	86.29949	256.18484	736.7435	12.628411	230.25258
DD7	24.736551	14.54179	10.364269	74.970665	243.40694	164.27896	30.274397	72.03898
DD8	32.64509	2.8131404	5.119846	48.437668	244.28758	64.21601	23.7464	10.298324
DD5	35.09328	4.8507147	3.4226472	54.80232	218.99594	88.724174	17.608643	24.791595
DD9	34.180916	11.536473	7.2707963	58.149544	216.22934	148.052	20.96335	2.479545
DD103	61.614796	30.588604	69.165245	108.23305	101.38952	37.374542	12.86058	12.569154
DD101	50.51097	26.260868	15.323677	227.1534	68.06775	70.718796	16.376158	84.65695
DD102	73.86367	40.899704	101.54758	173.57951	76.17136	305.91193	20.543964	25.36918
DD105	77.032036	29.754402	4.6950603	234.04181	89.90712	17.074408	15.092151	79.528915
DD106	67.31388	42.531227	33.164265	224.81523	75.910515	95.83872	17.954704	106.16248
DD62	63.070923	27.523476	22.330387	98.44397	50.248493	91.8051	8.312113	234.21576
DD64	85.45924	31.786726	75.9999	114.70529	45.988533	139.40248	12.498038	126.22347
DD107	73.708855	82.60868	231.65433	111.48432	38.864185	1811.6545	19.002466	33.286633
DD108	88.99076	55.088276	83.31032	95.60717	62.82616	1344.4785	17.962906	13.298162
DD63	90.1747	51.887722	87.4396	92.6519	75.96884	1460.9933	13.860526	23.954063
DD23	77.22199	31.936413	55.914536	59.444942	67.10455	275.84912	14.257754	9.10884
DD24	76.07626	38.479164	21.160185	82.263565	53.279408	95.34678	16.780582	34.0595
DD110	66.63452	28.262438	13.95621	75.99786	54.7552	44.02186	13.744637	84.99435
DD22	61.49004	21.713772	5.951049	67.64327	49.90211	77.09596	13.215939	70.11654
DD109	68.75256	19.893053	7.1126637	88.722565	54.376816	64.002625	12.448751	9.453894
DD65	95.13507	31.001493	28.670664	116.50165	54.606495	144.55148	11.459188	9.641504
DD66	82.72217	46.13736	9.626936	106.11134	62.922432	102.569	359.7839	12.137645
DD67	80.39293	22.01335	12.318818	89.37233	89.41627	32.104053	14.148327	40.3598
DD69	85.904305	45.15596	47.192127	114.27206	48.671715	198.88295	17.351448	1.8791227
DD68	80.86475	60.278873	42.417137	69.59152	56.442707	434.32236	14.35447	6.7469416
DD25	66.39751	28.777216	18.217419	153.67941	70.316284	331.68808	381.09692	2.0126574
DD28	66.23267	47.4408	1.6404067	97.25278	54.14173	88.80613	14.133974	2.3533316
DD29	70.099724	44.009693	5.3835926	154.0328	60.20741	61.94884	132.71802	7.538136
DD27	71.80022	81.221565	128.23705	196.88528	55.8476	2816.914	213.75165	50.357365
DD30	67.06536	22.78538	10.67598	157.28519	47.802	349.9219	307.4389	2.4837534

On-Line Supplementary Table 1

Animal	1383322_at	1383407_at	1383422_at	1383449_at	1383510_at	1383516_at	1383577_at	1383672_at
DD81	37.456055	60.568546	3.2238998	18.78418	452.03674	291.03522	187.2963	368.53857
DD84	15.805313	67.42578	1.0622139	11.07428	269.5308	321.77664	182.04054	372.2031
DD85	17.007738	27.163654	0.3807773	16.407972	236.92299	375.31305	164.27638	277.2282
DD86	15.245049	28.118898	0.7115842	25.160883	221.73422	525.19086	141.01422	14.698135
DD83	20.877497	30.172915	1.1056995	14.741488	412.19766	331.2718	186.04385	339.27145
DD41	83.0592	30.402576	2.016837	16.229471	411.13528	425.50906	173.0281	161.80307
DD42	104.53897	31.134205	17.059393	20.869091	363.43945	317.66666	164.13452	32.76054
DD43	10.241588	449.9268	48.24729	26.921156	269.57135	755.67896	113.79813	44.528225
DD44	19.509218	372.3129	27.36614	17.48426	377.6566	623.37665	151.61346	58.452583
DD87	118.20009	40.01069	7.3527713	17.775822	238.269	397.32285	142.01709	56.47348
DD2	38.23495	262.68637	25.885815	15.917677	375.46347	784.0246	134.4016	274.43582
DD3	14.101709	87.982994	0.8968197	14.640026	182.05003	781.41455	130.53395	52.958206
DD89	14.454319	101.80245	0.84209	12.161501	403.5036	799.0628	164.03096	57.646194
DD90	3.9266944	69.26955	5.6123796	17.728428	538.0844	1341.4025	201.01048	15.181271
DD4	19.254702	86.995346	0.748692	9.14677	361.59714	1251.3085	169.53377	38.629555
DD47	109.46306	60.053883	8.779439	8.368619	202.61629	412.63388	107.16795	15.36665
DD48	50.98629	46.023674	1.1769977	11.330275	335.5508	515.8742	127.87638	178.10878
DD49	14.793749	50.751026	1.1775587	8.415457	208.98433	888.19543	109.35777	42.21203
DD50	27.378426	53.156063	11.793834	15.385077	232.61073	985.4296	102.13553	30.223885
DD46	19.0003	50.838223	3.835412	15.11157	311.7812	890.27124	134.26273	70.80249
DD6	20.43343	142.65645	19.856724	9.121088	385.49097	899.01807	170.98438	193.69756
DD7	19.869278	101.1233	12.749295	11.590788	382.255	1245.8293	178.68054	68.68465
DD8	11.833734	31.498323	5.9898214	13.994117	312.3675	923.1331	157.56741	10.397639
DD5	10.615403	67.14261	1.5439869	9.286515	152.3934	795.4628	117.10365	34.2988
DD9	3.2170208	77.822975	2.1706672	7.1213484	202.44373	983.8277	137.5788	13.027033
DD103	26.770224	79.79181	9.123534	43.578148	17.040045	259.85565	77.31652	115.12224
DD101	31.326593	42.660236	8.152616	43.688564	16.939306	152.33073	78.81302	232.90797
DD102	13.345337	226.9411	36.120796	86.951836	17.808718	276.1274	53.42747	53.66922
DD105	16.70459	30.831816	0.9991445	43.859818	26.956038	256.1599	65.46246	228.72765
DD106	39.71306	58.892834	8.00813	51.00209	15.363048	241.58614	43.29007	242.18094
DD62	16.993073	52.593475	6.979202	61.56151	25.046144	336.85202	72.79058	147.68532
DD64	15.112412	110.41351	20.348259	31.69253	25.992096	354.03763	36.167995	62.54509
DD107	26.99581	481.45636	65.72689	98.08092	17.600306	463.9038	92.280945	31.080132
DD108	17.639744	230.05392	31.704597	39.96292	22.443579	312.1133	62.485355	34.743324
DD63	24.632618	222.19482	28.270176	45.940514	21.496298	314.5955	84.540504	29.647285
DD23	16.861212	134.33139	9.885236	38.80459	15.859733	444.14606	64.34915	14.615464
DD24	4.066977	101.17306	5.9084	74.812126	21.404	475.44266	63.761124	27.524033
DD110	22.170128	56.60338	4.4225945	76.60499	21.899345	426.92227	71.961296	52.24496
DD22	1.1380599	70.26014	0.9124625	27.606075	12.771683	290.38617	78.178795	36.279778
DD109	10.931139	61.590378	5.860494	26.144302	14.656721	363.39148	45.352497	19.259197
DD65	9.326091	112.07978	6.6374397	45.05357	22.807415	471.37338	38.623753	13.90904
DD66	90.87826	115.53574	13.173518	24.601707	17.232687	331.3551	66.65111	16.058655
DD67	3.5741355	38.89131	0.8572253	56.352745	15.998616	281.923	85.01861	50.634132
DD69	4.2890787	127.31439	15.930176	71.97464	19.936861	436.046	22.45083	17.868935
DD68	13.865819	146.28891	15.436234	94.33206	85.40036	442.11484	66.18704	23.391512
DD25	92.111664	61.862083	15.822051	26.147005	14.805502	453.57388	20.481707	8.8221655
DD28	4.660184	58.499836	7.8665886	32.203915	22.90701	466.5269	57.87023	11.382237
DD29	76.14089	67.18786	17.439962	84.865265	15.490206	497.34866	53.19386	13.338104
DD27	77.20402	346.63095	59.427258	19.241362	13.473743	469.14896	32.060436	43.160866
DD30	81.1126	74.9034	15.653248	38.156418	15.806382	433.15717	21.76817	10.372116

On-Line Supplementary Table 1

Animal	1383802_at	1383836_at	1383864_at	1383875_at	1383894_at	1383895_at	1384031_at	1384033_at
DD81	36.42745	882.10187	0.8666019	22.998503	208.64609	21.748785	11.901987	435.6034
DD84	35.783524	684.1323	9.382653	32.465427	173.16797	16.838934	7.132949	417.88727
DD85	40.88079	1079.7727	12.457436	22.390491	171.37161	16.229387	15.063765	354.65225
DD86	44.850857	975.1622	6.785803	30.46268	147.2996	20.325935	10.692735	12.890237
DD83	35.816757	814.98303	10.638107	40.413845	169.50986	12.911462	18.098202	373.90634
DD41	25.78179	669.38684	14.938775	39.059887	219.64928	20.577028	6.448865	71.45241
DD42	42.069862	618.8295	7.147593	54.604046	162.94073	26.55076	4.4286985	49.00299
DD43	55.387283	574.02277	10.192595	31.08035	171.59224	29.793669	11.154456	68.3036
DD44	48.930744	633.7963	8.15929	99.95871	178.96288	29.10009	10.975058	74.155525
DD87	31.459745	714.6267	3.912793	44.7198	224.59756	29.153536	13.586575	67.48282
DD2	45.448303	826.11096	10.691696	37.609398	221.76607	31.7776	5.7870326	275.87018
DD3	38.73378	1023.5554	7.5573993	58.382523	273.47128	45.96825	19.805084	88.26216
DD89	30.44994	1095.0309	10.55031	141.19542	325.4974	49.58735	7.775274	62.712498
DD90	45.089577	663.03894	7.607789	351.43112	349.76434	72.85175	9.737699	16.775476
DD4	34.916332	586.9636	9.473556	53.958786	215.35344	71.888885	10.635006	31.20024
DD47	37.861103	852.2567	9.853949	60.199097	111.2992	31.19885	11.330939	16.409382
DD48	31.15709	764.70685	10.809692	65.14817	224.8585	48.031048	14.87817	483.36725
DD49	39.647846	1402.2286	12.448829	125.08204	158.39223	72.98878	3.8400357	55.08617
DD50	42.556347	1238.4165	2.0411956	93.77402	178.51125	93.94988	20.512903	35.835274
DD46	38.659126	1304.9889	12.369887	49.782005	279.06198	79.482216	1.4230012	90.67025
DD6	45.92523	1253.712	13.581952	62.799206	243.93053	89.04683	15.452201	175.75284
DD7	34.043633	886.88074	10.850668	94.46237	224.26274	96.76735	4.894109	44.905315
DD8	41.286476	1588.0663	10.802912	24.044743	194.14362	91.1835	9.341396	1.3375375
DD5	36.227165	1576.6726	8.943183	61.459785	173.11661	125.45379	0.9437965	14.207173
DD9	28.549776	1038.8855	4.274218	147.5796	179.68411	90.204735	8.730758	2.5423048
DD103	11.390669	1939.7653	13.725447	81.98343	66.06884	14.640757	9.257389	102.56397
DD101	10.279351	1370.1044	9.325393	88.02568	50.824753	6.0287395	23.629803	277.28818
DD102	29.04978	1774.5236	20.070587	33.8411	44.123722	4.6378303	9.953268	144.05803
DD105	20.06235	1300.3414	13.342462	18.419243	68.45137	7.943529	15.857759	359.49738
DD106	17.738262	1197.3383	18.905127	36.99576	41.749516	17.10108	24.199211	267.0742
DD62	17.346895	846.34235	23.429964	124.38318	69.79126	23.050697	15.743485	226.89903
DD64	25.219524	1794.8022	16.664995	274.2043	138.48273	28.975817	15.421759	80.45882
DD107	28.4192	1511.0085	23.816647	141.28601	55.08303	25.47708	8.573802	38.38188
DD108	29.955471	1670.5498	18.121273	240.16132	51.466347	13.660045	18.761436	71.38561
DD63	33.997887	1743.5364	19.666883	263.85638	40.759026	22.320074	18.879566	68.42496
DD23	26.454582	1574.9601	24.80234	117.83978	95.1217	13.949672	11.25776	22.547379
DD24	9.746446	1959.877	22.827557	156.81635	72.28178	20.570223	10.501558	43.016617
DD110	23.85624	2117.965	26.649527	350.32666	65.60648	48.726875	13.369146	58.13156
DD22	15.620056	2284.6409	18.675463	311.90527	54.53557	6.4859533	11.332474	52.50348
DD109	24.148516	1515.5946	21.896358	231.21794	147.2973	19.277706	16.525957	11.324307
DD65	16.352509	2123.9329	15.546551	317.6622	71.354294	46.382645	5.9906006	24.925507
DD66	15.716556	1803.0768	11.747843	93.28739	50.268616	39.402554	11.897993	15.67939
DD67	18.850157	3717.0823	17.599176	88.108505	112.13191	11.604635	21.760199	45.66086
DD69	14.409504	2917.7341	12.698823	392.59134	69.44785	47.909405	17.969446	6.4314523
DD68	23.07231	2265.8198	16.142025	119.61051	139.89742	65.32264	14.006606	13.783745
DD25	22.707544	2082.9734	17.438494	102.88054	143.15115	42.29099	10.807653	2.2876084
DD28	17.483284	1621.0377	11.616898	386.1672	133.21082	72.233025	17.743414	1.1808585
DD29	20.589336	1485.6963	24.19376	456.04453	68.54111	50.318058	11.9839	1.6491705
DD27	24.311508	2049.93	9.681223	35.933243	144.70026	63.609287	12.610169	31.789185
DD30	25.600847	2106.4229	15.589811	106.65969	170.50284	29.337208	6.735996	0.9175709

On-Line Supplementary Table 1

Animal	1384054_at	1384058_at	1384073_at	1384104_at	1384218_at	1384219_at	1384236_at	1384247_at
DD81	78.78849	149.68246	31.178747	0.3129679	159.54494	47.572544	7.952817	51.76655
DD84	36.130775	97.93208	21.317247	3.0859785	294.05463	37.680374	1.2812641	59.017513
DD85	73.68016	98.76922	7.1947308	0.3569553	3.2025087	43.764233	1.7822357	61.71497
DD86	126.69919	135.53804	24.943134	7.458739	26.85546	56.75376	9.277378	1.2396225
DD83	76.27491	97.893394	18.214657	1.0529557	25.833338	40.647175	7.711979	65.58245
DD41	49.97599	106.79451	4.5340466	4.9854007	10.133859	62.610855	5.232713	38.22142
DD42	28.725073	94.222885	28.817398	6.7061386	1.980746	62.75963	3.0735161	13.578126
DD43	39.78592	65.62401	5.7709556	0.3645163	535.09576	62.818756	3.3976083	15.910597
DD44	44.431007	106.19785	22.928137	1.5008419	1227.9801	63.96779	15.398554	17.305332
DD87	45.55101	108.5397	16.447296	0.5811263	30.55088	73.78474	3.5212748	23.210331
DD2	45.166058	85.91647	88.75675	5.889238	369.0443	51.297604	2.5281253	34.113194
DD3	32.723946	122.35724	45.63963	0.7215876	12.717567	86.12126	0.2409123	9.631326
DD89	67.17494	150.1579	49.69531	6.201421	10.978576	60.54415	1.8726826	15.415879
DD90	94.53569	226.11942	60.831173	8.566368	7.875093	82.43392	86.889175	3.3678544
DD4	256.77298	170.0106	55.572487	0.1792912	5.107882	66.67314	5.278913	4.299522
DD47	63.335083	117.06554	29.840153	3.884869	3.819183	68.705956	22.271006	5.5628595
DD48	76.92171	113.54352	40.653313	6.9684553	4.3185024	51.76436	13.34226	52.4675
DD49	98.77912	145.08125	40.18668	0.5454775	97.93391	60.132824	4.3884854	3.1259148
DD50	159.5812	203.5728	16.72082	0.415464	10.64396	78.19268	12.096391	1.2800632
DD46	194.27495	135.43939	49.030754	8.169489	5.056364	74.18633	0.6673277	14.813223
DD6	136.84291	150.84361	82.34221	6.718746	679.1341	53.06228	0.2893157	30.738678
DD7	84.24767	229.70111	50.656845	2.898238	37.832348	57.15779	0.494247	15.071913
DD8	137.5606	130.15446	92.079384	4.68853	4.317248	59.879906	1.9067222	0.9691622
DD5	140.4933	158.47844	50.260975	10.320096	23.1596	74.558784	0.8102402	0.3899829
DD9	82.91807	125.33482	45.447037	6.0839634	16.338785	60.318855	1.4265842	3.190895
DD103	1.6338702	14.978666	86.254906	27.858831	17.93591	100.06081	0.2809559	28.778751
DD101	7.6307726	3.5359204	78.884834	10.25825	110.47207	119.5776	1.5850554	45.25368
DD102	6.4641585	12.343298	60.058193	36.87446	286.9548	141.41403	0.1200455	25.477045
DD105	1.8968786	12.612821	52.142952	17.793749	10.746143	87.13403	3.6782262	57.716003
DD106	1.0524873	7.4623456	64.00024	13.440738	354.63254	131.16069	4.7564163	45.64805
DD62	1.2720121	13.373376	161.47903	18.57178	48.91705	79.91038	28.852804	63.96301
DD64	2.5002525	14.953993	74.36351	27.367052	54.137653	113.42938	56.90636	30.45163
DD107	2.6391003	14.607498	58.204918	27.324686	411.41614	141.53531	40.347275	10.510469
DD108	9.414083	17.394064	72.884476	27.211786	407.1909	158.22963	89.153305	6.6302333
DD63	13.769287	15.434545	63.214035	20.070364	417.42337	166.02383	67.51802	7.140939
DD23	19.064157	14.586664	98.58379	30.883827	53.80883	172.2395	22.975792	0.6346219
DD24	27.149086	15.354255	98.911125	21.501627	31.896193	93.788445	20.960682	3.3902643
DD110	14.910524	9.874142	122.34751	40.81424	6.785999	110.12141	64.83039	22.094007
DD22	18.372551	4.228516	168.13297	20.698431	10.243045	129.79634	43.24292	10.313004
DD109	26.255663	4.1197343	108.55591	19.264565	15.865228	144.46725	28.58654	0.4473691
DD65	4.5354757	16.122433	65.96491	29.314857	20.836214	177.04375	98.22802	1.1342648
DD66	7.41475	39.4898	71.221725	25.059025	23.999245	209.2039	22.657698	1.0854887
DD67	28.03851	9.440202	296.7763	28.826092	1.1746782	122.28754	4.1709557	0.6741021
DD69	41.026104	7.9817753	85.80235	28.089258	32.273994	153.13766	96.05949	2.2470946
DD68	35.480057	12.03259	58.8348	18.635817	52.775402	166.16273	1.0536548	1.1884363
DD25	43.229855	58.664383	66.97182	24.749601	42.495644	188.12666	11.12012	12.460935
DD28	35.09518	16.049622	64.09758	32.361744	2.7784846	193.9699	152.1709	0.3273626
DD29	40.14055	18.43724	79.92136	30.735378	16.96703	124.29206	142.59868	2.0783823
DD27	18.404696	11.039452	51.48056	27.6607	583.84186	183.10962	22.032076	7.536756
DD30	45.210808	65.16825	125.29757	31.421665	67.518814	196.16443	8.871855	0.2127357

On-Line Supplementary Table 1

Animal	1384271_at	1384334_at	1384499_at	1384598_at	1384667_x_	1384695_at	1384717_at	1384794_at
DD81	224.69872	499.0103	162.09688	11.441714	8.177705	43.526634	185.14119	14.728679
DD84	176.09306	3.7122276	59.60132	11.659829	5.818498	42.76733	193.0264	18.251173
DD85	22.322056	38.754284	71.20195	19.05531	4.1374345	34.59323	252.67044	6.2308364
DD86	12.798795	24.427475	84.00974	18.416513	9.4728775	43.971497	274.80893	19.531626
DD83	56.99942	10.76565	59.700226	14.775135	2.3554149	37.459003	256.3571	22.279581
DD41	15.879125	5.323457	50.743984	31.774143	1.6317654	29.011105	229.52748	21.037727
DD42	12.098992	9.108824	49.432693	19.642763	8.690076	20.81357	198.21552	15.400886
DD43	1064.5167	13.925743	50.308353	24.053623	7.942235	27.56979	261.32486	13.982671
DD44	686.6891	18.875946	42.355377	15.968912	2.3503926	64.86871	293.385	19.346518
DD87	16.74476	3.8702984	47.758045	30.71496	7.7737684	23.249866	224.32574	13.014454
DD2	488.0389	2.9384058	34.40259	21.205048	10.123688	12.262542	332.30634	10.348235
DD3	25.65896	33.901737	44.961025	33.61274	12.123452	31.344347	284.61075	19.800966
DD89	29.937284	4.956337	62.7815	17.238142	17.526144	31.540384	312.36856	22.23619
DD90	39.469955	45.115303	65.810486	22.357244	19.906921	350.44263	276.1898	19.037216
DD4	30.201868	8.28634	78.803925	73.19497	20.565252	36.57913	337.89822	7.683222
DD47	3.0624912	140.01472	59.535927	8.279798	12.953089	20.277542	210.95172	11.291801
DD48	9.077263	439.73334	75.54437	20.01915	10.418533	28.433458	207.73894	7.3776793
DD49	6.683915	96.42642	70.064476	16.89173	9.947259	22.378405	318.16458	10.728417
DD50	1.9354928	133.80817	69.35355	20.540108	28.720722	70.26115	267.57513	19.556599
DD46	22.216265	218.60103	65.87201	8.071155	18.940714	41.863697	385.74988	20.246273
DD6	211.77716	8.378312	42.941967	13.458728	19.483889	32.545418	393.59973	13.940956
DD7	82.11488	1.9483067	58.71608	17.576143	20.543573	30.774683	457.14868	13.884451
DD8	14.617864	7.032131	44.412914	32.2563	8.536968	32.4365	329.93524	18.043541
DD5	19.485016	7.1586223	65.226006	34.42216	14.109444	48.04619	329.48175	19.618454
DD9	39.486397	6.151426	63.29943	35.159206	13.303913	44.188972	416.99554	15.689683
DD103	179.97185	57.765755	127.46065	9.212783	1.7661183	23.602427	17.479307	51.55674
DD101	74.035286	37.34306	17.08776	22.132978	16.821156	26.040508	18.268538	38.057873
DD102	474.94232	186.19576	137.37723	0.8647467	18.960297	25.827929	148.08304	67.99795
DD105	49.047077	57.044376	99.51375	13.721392	17.585032	41.037373	17.908133	52.321457
DD106	184.65091	209.1336	146.92621	19.388153	20.882929	50.18996	101.38958	41.162247
DD62	76.142136	5.174609	145.93703	11.177123	21.362885	122.048	203.48924	40.204327
DD64	301.1146	3.9338343	107.8706	8.937004	21.746288	292.27353	30.129707	64.01063
DD107	1126.1818	9.137418	96.47065	7.744482	16.679586	127.17088	390.9423	36.151752
DD108	527.9653	8.040107	120.45264	5.130876	20.968456	389.7086	77.71481	65.07183
DD63	517.42395	7.237521	127.3783	9.701301	22.736961	373.28793	85.429344	56.879986
DD23	185.32277	3.9228609	132.26503	5.279521	24.4651	107.84206	140.85493	52.020756
DD24	61.43296	10.416463	111.59827	10.598282	41.197742	101.21734	22.349928	31.323149
DD110	52.476204	5.3044515	28.445755	13.449549	31.683157	371.70316	28.95138	36.579487
DD22	10.228896	3.4647515	102.3205	6.682562	19.713223	253.89584	25.67384	31.031248
DD109	20.412086	1.0151244	143.97998	13.1068	24.660559	118.75464	30.527336	42.628784
DD65	75.79863	6.441452	201.8621	6.3719873	46.197857	381.40823	141.4331	14.203291
DD66	45.843563	4.2633667	64.294876	15.492919	43.13222	24.754448	250.38698	51.999817
DD67	17.971043	1076.541	383.64566	8.588823	33.718037	22.000322	21.734682	50.48224
DD69	112.16258	7.2814217	104.83182	9.37401	37.007267	464.37497	22.576504	52.904034
DD68	157.27072	54.018856	132.80429	19.824627	40.426876	27.462261	171.75897	41.577534
DD25	54.514496	10.227098	193.06178	12.341222	40.502514	39.595898	167.27559	51.281193
DD28	13.899791	8.271513	120.47507	9.274485	49.787357	533.0168	148.07408	46.71852
DD29	19.156645	6.7595634	73.5727	8.172922	46.931576	628.4543	155.7897	51.880104
DD27	976.8417	0.5961548	124.12035	11.702637	30.024357	20.312231	135.36263	44.713326
DD30	51.57582	1.9724579	139.92935	12.9439	44.00773	40.008434	172.22449	32.93964

On-Line Supplementary Table 1

Animal	1384818_at	1384836_at	1384944_at	1385058_at	1385096_at	1385120_at	1385202_at	1385216_at
DD81	73.003525	10.20223	23.356396	4.790579	4.951897	8.775352	5.1367097	14.445085
DD84	56.159763	10.190693	14.131808	2.5389614	14.958825	1.6617844	10.810127	2.4723141
DD85	43.68511	15.44134	1.3494054	0.2477202	12.394455	0.807048	2.0188942	9.408316
DD86	45.300243	14.528951	2.5885127	2.2679305	14.404098	1.0499367	16.016844	4.5141897
DD83	57.513264	12.079949	8.7948675	1.8586357	12.454171	2.1384048	3.2126994	1.1255678
DD41	53.351227	11.042054	7.7099085	0.72209	8.665378	0.7753996	0.5944781	0.6351045
DD42	51.67885	9.493955	10.627806	5.4252524	11.05957	11.315537	1.0918678	0.6062967
DD43	39.85467	9.949238	277.1401	2.1057198	6.160779	2.3386693	8.835794	84.30457
DD44	42.24416	13.638862	256.99866	3.5648425	11.986212	8.422955	1.7142096	47.223255
DD87	38.505913	14.116037	15.55586	5.679838	11.606087	2.9280136	3.496031	11.941081
DD2	35.67451	10.382031	125.96263	0.6566628	12.109427	14.267357	2.3294652	34.393364
DD3	29.908844	14.31773	14.689733	2.4808352	11.996752	1.1338278	5.4974637	8.913982
DD89	20.602825	8.47776	18.44374	3.3401322	13.175128	11.442667	0.8625816	1.023129
DD90	15.939597	17.767044	19.703947	74.64381	16.059607	32.534977	7.9497843	5.5630784
DD4	29.929312	12.689482	31.517353	47.778587	14.550589	5.769608	1.1933951	10.992476
DD47	42.945705	13.445871	10.69128	13.098844	3.0654292	9.215034	0.9414095	0.8099683
DD48	41.444267	16.636621	12.451259	0.2296344	9.02983	5.8392777	1.7081971	4.7824545
DD49	47.84855	10.132306	14.954118	0.8744064	8.948068	3.0150454	1.3894931	1.7304626
DD50	46.97151	14.61655	5.4552374	12.414022	6.9909415	3.2975366	3.26154	6.0306325
DD46	39.961906	17.02789	15.352713	4.426294	13.556373	6.1576552	3.0622642	11.272517
DD6	43.50337	16.618671	96.990654	4.9822106	15.716394	1.5330628	1.4191608	28.293142
DD7	30.447226	13.069553	45.9756	0.4132312	17.546524	6.8312573	0.7662669	8.301351
DD8	33.454227	12.764915	18.711456	5.243681	13.398098	0.501658	1.2042984	9.327405
DD5	41.513878	17.805725	19.003954	3.778091	11.327045	2.3002727	1.3090402	6.3238835
DD9	35.30828	20.808685	49.164818	2.62433	10.299184	7.206206	0.8520103	7.520685
DD103	55.541264	12.52111	65.42028	6.19337	8.695919	1.85714	14.8146	4.2878613
DD101	42.3548	8.522892	17.291199	5.186045	5.490532	1.0326627	1.5969131	9.892884
DD102	56.08798	16.022154	130.71898	3.146742	0.7851082	5.023601	31.348082	20.37431
DD105	42.33985	16.025253	8.722076	5.4888062	6.6386485	5.259493	14.051733	8.6998415
DD106	34.921112	14.938613	26.397627	0.7482318	5.878049	9.42841	11.764951	16.135817
DD62	56.775043	16.209848	23.573486	3.08142	6.5541277	12.488035	1.6992494	16.243996
DD64	92.147095	40.58833	61.588734	17.349365	12.50708	37.77669	10.67312	17.73405
DD107	59.599247	12.373074	230.83821	13.346223	11.625362	16.335346	15.795954	66.71995
DD108	116.80866	24.62016	129.85458	42.568752	8.86803	38.496098	10.366323	21.72154
DD63	98.246605	41.397297	108.2037	41.89622	8.090876	39.711296	10.513771	29.605778
DD23	49.15824	40.913406	77.19905	6.9970393	10.784937	19.93643	9.713233	21.351606
DD24	26.59903	28.111889	22.533388	12.142116	9.944758	18.325642	0.9761728	9.820201
DD110	61.177742	35.584244	12.077137	31.940094	3.0134263	45.466957	2.100607	13.109159
DD22	57.372692	37.93322	3.0100176	25.464792	0.8287789	14.209901	0.9430889	4.700464
DD109	52.702957	40.413834	11.715565	10.537631	11.584171	10.201582	1.3832096	7.2849746
DD65	68.54725	34.52194	35.97615	41.23824	6.410668	33.45236	1.4621634	16.129553
DD66	47.83685	11.996026	13.175037	1.796287	4.5031757	2.0138173	1.9273157	3.6933248
DD67	42.552586	33.43458	5.8230824	3.146745	10.930999	4.2239776	1.0798781	3.9495006
DD69	61.301086	46.241257	44.726685	45.86291	9.3662195	52.3083	2.867615	24.31669
DD68	32.57071	21.301956	49.02826	1.3196603	6.214764	9.564942	4.742272	11.426929
DD25	59.372368	33.88063	15.552051	2.8182728	11.028108	2.370425	4.7555614	11.404964
DD28	153.07022	46.57412	6.942975	92.81483	8.973592	67.67949	4.2637296	6.5690074
DD29	163.76846	66.83693	15.962267	85.701706	2.8937588	54.030357	0.9374048	2.925969
DD27	38.01647	24.801395	135.0796	0.6409721	0.9393166	4.032497	14.571754	33.92841
DD30	74.95125	22.020807	16.84316	4.1720676	11.353345	0.9354599	1.4723935	10.102964

On-Line Supplementary Table 1

Animal	1385225_at	1385343_at	1385381_at	1385395_at	1385464_at	1385465_at	1385609_at	1385635_at
DD81	10.176106	1.7594084	10.198904	26.407784	6.113408	10.877353	19.311848	67.116554
DD84	17.515736	9.666337	23.932741	27.82083	5.863777	13.423722	21.333387	8.897007
DD85	17.578173	0.2839916	27.128136	6.524067	1.093822	10.018595	11.543245	19.70115
DD86	9.598008	9.086849	25.140202	34.080765	5.1033792	9.389158	11.607494	16.19849
DD83	13.765688	7.2837543	26.63619	9.753785	2.648168	13.213724	27.901484	12.091909
DD41	11.292261	7.7230687	36.13151	30.188074	1.8176051	10.245729	1.9476938	30.234888
DD42	18.392782	3.1016788	23.648483	45.45871	0.7449638	16.493263	3.2425098	13.499723
DD43	32.844032	6.4977226	34.553745	20.533268	1.420753	13.599915	16.544964	34.345398
DD44	18.82917	10.587003	23.124557	27.267178	2.0533564	22.964777	28.967909	44.457756
DD87	24.873272	4.133021	57.899117	36.776783	1.5827123	15.186216	15.713255	25.06553
DD2	1.3747331	1.9643561	16.458044	22.153883	0.7277904	18.79562	3.0898712	86.57211
DD3	18.643652	14.818501	24.632082	29.190268	7.126079	36.12234	14.589693	86.90057
DD89	20.557396	8.4653425	34.818363	36.480366	0.3491259	35.443718	19.355293	79.500336
DD90	25.197618	11.301127	32.869503	18.006561	20.360226	30.557865	44.187233	78.943306
DD4	23.35185	6.11505	43.322212	5.757258	0.4656636	45.23958	30.434237	107.72363
DD47	35.633766	13.695073	20.757816	27.186047	1.3012314	20.423588	15.031524	92.99761
DD48	20.018343	2.1130385	34.812794	12.781132	0.4646953	23.433226	9.80605	66.69256
DD49	8.318097	10.84046	49.222992	22.947157	0.3107961	20.564741	9.328659	63.687084
DD50	2.0579035	14.476742	31.408264	10.329088	6.204302	23.57786	54.443382	33.026028
DD46	7.143227	5.3448772	47.183903	28.073498	0.8713915	33.132614	9.431255	56.65606
DD6	1.4400431	7.5435677	43.133263	41.803295	3.6135476	27.461243	14.594751	57.33872
DD7	2.7441578	8.334277	41.48194	42.053516	5.3041534	42.604385	12.996728	93.28279
DD8	3.3205812	3.8843045	33.580853	24.757101	1.6593978	29.032482	3.4334774	55.923065
DD5	5.9767265	7.107566	26.672453	11.339349	0.4846374	25.735893	4.687002	95.855705
DD9	1.6736324	6.6139045	35.208527	6.848449	5.3285766	24.833193	1.1140844	127.88217
DD103	31.905918	8.123959	10.441393	139.31166	0.518353	45.365273	2.693438	11.293151
DD101	30.364777	1.1996255	13.084964	71.33998	0.7222524	36.496452	15.610336	9.37082
DD102	28.298498	12.430769	23.212973	112.01453	6.7975535	87.26087	20.968077	15.280196
DD105	26.725199	4.506953	20.499834	103.6233	0.4439768	46.65039	13.493709	12.994578
DD106	23.264421	6.998939	41.556175	71.32533	0.4651691	37.405087	4.365237	12.571335
DD62	15.395938	9.986559	16.488308	63.202244	3.7992902	13.808946	14.667922	9.825193
DD64	32.92337	16.490952	11.75196	98.02091	16.79677	145.22163	58.573956	14.107777
DD107	23.68926	8.928761	16.663143	71.38134	10.087916	24.21118	39.563683	24.05832
DD108	46.13872	7.148034	16.422731	93.992584	21.704453	71.61846	92.69957	10.637581
DD63	49.914627	8.944295	16.228714	105.25433	27.594816	75.27781	86.60085	13.138596
DD23	22.94973	11.671731	19.1365	64.493004	12.149553	108.21046	17.726149	19.028753
DD24	44.434963	12.888732	16.29463	75.701454	6.6725454	28.667068	17.930435	23.300266
DD110	43.914886	16.743046	11.242206	70.62931	23.792881	152.23701	100.58641	8.404898
DD22	26.262537	14.523572	9.571837	66.74906	5.733934	22.500168	65.87686	12.704266
DD109	34.610302	19.901491	14.234485	77.94594	2.0751674	174.27893	29.086151	9.402116
DD65	18.705383	18.998241	22.05191	74.866035	31.589088	228.69453	119.46733	43.235626
DD66	39.000183	13.671209	25.587887	55.879803	5.729414	147.89166	13.662695	119.86514
DD67	16.124104	26.789686	13.358306	99.34758	0.7778839	113.00674	0.9506913	36.684467
DD69	14.2382	14.04983	15.704542	63.632183	37.270725	332.51062	153.32045	48.312077
DD68	24.716768	15.784039	13.610057	77.90277	1.1060722	195.35075	8.851183	29.584309
DD25	16.843262	20.806461	23.316782	54.44618	1.0445613	176.28055	17.170555	44.71905
DD28	29.31455	19.984074	15.671986	63.059376	46.556923	189.55708	192.39267	64.567154
DD29	16.472435	6.1708474	15.253985	88.67206	57.980198	156.77603	220.17957	90.63993
DD27	19.285873	1.4901353	23.993095	84.56671	0.4552768	180.4192	11.226734	15.235827
DD30	27.998789	23.355286	28.045683	45.95203	0.7545033	191.38036	4.529666	37.235493

On-Line Supplementary Table 1

Animal	1385647_at	1385700_at	1385751_at	1385799_at	1385871_at	1385892_at	1385974_at	1386128_at
DD81	0.7281456	86.946106	94.88844	14.378259	261.5104	1.5736775	32.650738	54.064365
DD84	14.092065	52.585423	103.08489	7.020568	269.65466	3.788518	35.840565	190.3487
DD85	13.552236	2.2921548	109.6664	19.605438	256.8409	4.4099255	32.56521	104.15151
DD86	10.393466	6.263536	82.110664	11.757012	174.12953	1.8090162	48.89356	245.78091
DD83	4.9639225	31.498476	94.02439	10.956628	183.99149	4.3792815	31.367085	130.05095
DD41	3.762889	3.6663337	90.2482	10.644898	283.01578	9.422801	10.100478	249.40369
DD42	1.5043839	8.960134	64.8431	12.487226	262.26758	2.7820745	18.695385	252.22574
DD43	2.269463	443.82657	87.529945	17.221006	378.9145	5.800826	39.099922	313.18286
DD44	0.9897531	318.27747	100.15637	10.960014	267.42334	2.16521	33.878468	237.22792
DD87	2.3389804	15.779242	80.05498	15.464485	317.2139	11.788567	30.639708	285.01202
DD2	7.331532	371.5165	214.63397	11.307623	265.8006	0.6121579	27.713146	110.8057
DD3	1.1056317	14.008772	217.1258	8.132534	181.7332	2.1552162	31.058956	354.25705
DD89	4.6550155	26.682184	145.18828	10.423438	320.55487	1.8745668	19.395695	57.669193
DD90	1.7557876	19.986486	232.53798	12.003951	501.0975	14.686845	51.84443	24.785273
DD4	13.423844	9.948964	326.2738	13.909714	407.71176	16.238684	30.544603	14.318731
DD47	4.8678527	8.685014	145.73648	12.742548	167.27545	3.2251587	25.05317	278.33572
DD48	2.0434582	4.307193	202.9884	5.155277	304.27563	8.331147	28.097498	322.29736
DD49	0.9072599	5.0129786	289.3038	16.774734	172.36855	8.155875	41.001095	266.72977
DD50	5.794986	2.631735	286.97873	6.712376	184.44244	5.085161	31.068068	218.3266
DD46	1.3268358	14.893594	309.92957	13.442871	268.2214	10.585463	25.930897	177.39812
DD6	1.0094904	178.40138	279.03085	10.615908	396.1541	15.813027	37.802227	83.89545
DD7	4.6731806	131.53912	315.21082	10.181842	384.62463	1.7408276	38.024364	33.527348
DD8	1.3789932	15.097178	327.82382	14.30617	395.1785	5.3645186	27.075153	40.846138
DD5	1.2057309	14.746615	252.52942	8.837382	244.01567	21.449577	28.977575	29.112886
DD9	1.9610854	33.7985	304.3797	24.038292	314.08035	10.38542	31.270044	27.301
DD103	23.06177	56.302483	68.79292	8.889282	692.3265	29.369915	10.814036	581.1198
DD101	5.83447	34.844242	100.86954	3.5414495	749.87634	4.0097346	11.90771	465.5011
DD102	14.201475	161.79958	76.00607	6.515522	890.82556	26.39981	31.762463	378.1778
DD105	16.465097	13.645271	96.242065	9.094979	713.92285	1.7192125	23.56974	386.5131
DD106	7.236883	44.30724	118.17452	6.172731	880.50366	60.34691	14.759595	844.7928
DD62	30.325317	44.246124	88.342186	0.8079768	790.4464	31.396091	10.20335	92.854164
DD64	46.014545	141.03491	112.95376	4.553607	719.6676	39.54167	18.678034	82.298195
DD107	27.264324	607.0285	103.45033	0.3566204	806.8289	29.620316	11.646468	47.217377
DD108	34.2531	168.6287	83.06405	9.158652	639.8038	20.835283	14.579007	681.94763
DD63	35.248222	168.38278	80.06101	6.8500996	606.99066	18.471035	21.914104	678.5595
DD23	53.683598	85.2869	120.66317	3.5166419	588.40063	24.55425	14.083647	920.6531
DD24	74.81771	28.969225	100.20834	0.3089519	571.18945	30.710093	14.565347	219.14786
DD110	41.679676	24.13824	91.21588	5.047064	657.4147	2.8212311	13.926922	779.7115
DD22	75.661705	2.9397159	113.08997	1.1852696	484.78275	9.827415	5.576391	199.69435
DD109	44.76886	11.157028	114.12876	0.1016489	642.2475	107.47112	12.730937	413.63763
DD65	24.357044	31.690413	136.95325	3.9034126	644.4353	40.87512	23.064505	560.5962
DD66	37.980404	30.466976	117.95139	0.4753574	457.6429	16.905102	2.8919616	1121.0718
DD67	60.262295	8.837925	78.22002	0.1468094	617.48895	32.07955	7.917006	392.01193
DD69	43.984276	47.136646	152.79964	0.8962281	670.8006	48.479313	14.02619	266.29318
DD68	54.424484	77.921524	134.51671	8.865995	605.22345	53.39816	2.759074	952.4785
DD25	44.752968	15.453937	124.54449	0.3754902	633.616	56.288292	14.044	122.00816
DD28	1.1846275	2.352524	137.88197	2.9289114	579.0905	62.20271	20.559343	52.748085
DD29	37.000965	10.454953	122.67436	2.0690114	684.4913	62.168976	18.995722	582.6884
DD27	35.373043	335.5375	104.40466	2.543291	892.445	40.086327	10.622924	80.67941
DD30	57.163445	11.709011	122.35603	2.294838	698.4532	74.53542	4.8335466	128.81721

On-Line Supplementary Table 1

Animal	1386160_at	1386333_at	1386383_at	1386466_at	1386621_at	1386679_at	1386697_at	1386770_x_
DD81	12.648684	192.85216	1.2658082	8.127407	72.24549	4.0337834	17.893131	33.28958
DD84	15.666427	107.13039	1.5288243	11.167679	116.55946	3.5102181	15.82014	41.218628
DD85	22.736273	135.13153	1.7425674	16.470089	85.855644	4.4296145	18.77131	36.85885
DD86	32.394306	2.0358474	1.6237874	15.420771	66.044365	1.0078424	21.960533	36.558018
DD83	11.569451	104.758	2.2773798	11.054244	104.95204	1.2108549	27.64257	19.39832
DD41	9.131828	28.771091	0.9974388	9.293172	112.21847	3.0571377	28.458649	46.436214
DD42	13.233408	3.084239	1.9166169	10.005176	132.84607	8.671206	15.23958	31.935871
DD43	14.276521	2.2091963	4.488447	10.696567	153.49513	47.13135	26.914911	47.4619
DD44	15.698424	1.6740954	1.5463766	19.241978	114.31641	38.963177	18.78266	37.035473
DD87	13.851777	4.21495	5.456924	13.0091	94.68048	9.531057	23.447952	44.44541
DD2	39.058437	60.498363	7.406116	10.750584	108.36046	24.991198	10.008438	27.046167
DD3	37.721878	1.5955731	0.701255	12.943584	101.76324	1.6853299	12.197155	43.167664
DD89	33.345043	10.112576	13.311207	7.9336467	84.48854	1.8289456	19.189308	44.16845
DD90	43.237194	1.2338938	8.0490465	17.35382	92.46089	44.462864	18.62724	50.48072
DD4	50.84366	2.3001072	1.4999967	13.812347	72.8625	3.1579769	15.249316	55.607838
DD47	20.288141	1.1440403	1.431144	9.90173	69.605446	1.3461933	13.990463	29.85107
DD48	27.542595	75.16426	4.5851107	11.157475	96.127235	5.208819	7.3830476	25.753164
DD49	48.98774	1.9240803	0.4100556	9.981307	86.87661	1.7750052	14.093061	26.181143
DD50	46.2085	22.084734	5.4850426	5.7636867	98.33509	9.360569	4.8368616	14.573232
DD46	52.685143	12.375495	7.5355268	10.452127	126.6909	5.477522	13.324688	36.618233
DD6	52.335026	65.02057	8.126006	16.445189	106.01388	16.283884	12.523752	44.599
DD7	57.301037	13.941334	5.2846737	16.118103	85.75377	0.9529017	17.862637	47.78012
DD8	74.469315	1.9123929	6.780876	14.023923	122.74403	1.2698791	19.337961	39.91282
DD5	45.553955	11.111468	2.9397368	13.411916	67.03181	2.6669989	14.974295	35.639805
DD9	52.901287	1.1009103	7.121213	12.424516	83.6341	7.1498194	16.56754	38.448734
DD103	10.527886	69.13926	4.4326706	34.907013	18.164663	5.183763	13.393544	0.9692277
DD101	8.443837	128.78415	4.492325	21.231253	18.725718	1.9103193	5.5801334	4.9393277
DD102	17.980442	29.390017	2.4897537	37.86744	59.86366	27.980755	10.487357	5.3411117
DD105	11.552411	96.89042	8.683104	27.372469	56.03495	2.1897147	14.109478	4.550787
DD106	10.185602	88.71817	2.2593575	24.24935	23.537056	6.663671	9.848415	3.2780118
DD62	10.198148	132.78592	1.9287483	17.498709	87.47266	25.259066	9.9453125	19.803091
DD64	17.717072	57.107307	5.22283	23.255714	105.2358	50.59297	15.500045	6.246556
DD107	13.580507	11.27096	9.753572	43.419712	75.78477	52.59354	10.331465	32.696075
DD108	10.486752	2.265766	2.0732594	38.413456	19.13143	72.500656	11.011723	14.829187
DD63	20.385603	3.4885628	1.7792767	34.019703	17.59807	71.42894	12.073734	14.812701
DD23	31.855103	1.1530036	1.0475874	27.051962	57.463333	29.508926	15.140946	7.2232647
DD24	12.688882	2.375648	0.9817762	22.451538	72.66816	18.915314	13.261098	26.844568
DD110	14.023376	36.15822	3.1012125	26.540432	14.535955	46.635704	9.440404	0.6793681
DD22	14.573003	7.6784606	0.5742374	20.836403	58.941967	31.354177	4.749703	2.4405336
DD109	10.291269	1.7286826	1.0564786	14.521485	11.668581	11.441728	5.733323	7.730714
DD65	21.065403	1.6530503	7.9514694	22.31114	9.115386	69.47336	11.515581	3.2042062
DD66	7.0625935	1.395252	91.41627	13.625931	45.279236	9.826013	1.586704	34.705677
DD67	21.204397	2.5847628	11.162303	9.661236	54.044666	1.4342017	1.1280661	5.2270336
DD69	22.586355	1.4882798	10.72056	26.56476	63.487335	59.864807	7.971315	10.240842
DD68	15.021412	1.4020389	6.5958023	17.71269	22.58848	16.777672	3.3072941	0.3640335
DD25	32.197884	1.7556897	135.73262	24.581354	51.144085	2.0678256	4.1612825	9.711846
DD28	15.483645	1.6571943	12.399295	24.57214	63.542545	110.65881	8.029183	18.699772
DD29	25.077845	3.4224045	167.1016	33.422962	13.964311	103.09489	6.311138	4.4002175
DD27	10.294287	12.654609	331.00632	32.957714	149.88925	37.781452	8.271623	8.042681
DD30	25.224588	0.864109	129.0929	15.112763	39.637802	12.676739	2.1363487	9.49601

On-Line Supplementary Table 1

Animal	1386855_at	1386859_at	1386869_at	1386873_at	1386889_at	1386941_at	1386947_at	1386969_at
DD81	38.700546	3321.0679	166.72902	1999.541	131.94547	413.22192	46.623486	26.153677
DD84	53.58171	3623.2014	306.4751	2201.2273	188.61246	481.49997	10.908229	30.845749
DD85	48.744076	3296.0188	224.65598	1897.6097	247.49301	408.9698	43.425594	22.721819
DD86	35.833908	4710.167	220.81038	92.42821	253.44069	278.25638	60.00256	40.342075
DD83	58.506687	3054.2178	150.17245	1974.7953	162.77783	380.14288	5.287868	29.734854
DD41	31.390905	2927.6672	51.389256	914.93225	218.44774	155.90372	5.340833	36.462826
DD42	29.848036	3249.8633	64.62681	262.56387	528.5431	159.7378	1.7822931	49.813595
DD43	46.13952	2890.8855	96.61193	139.44157	147.12236	191.83694	20.856045	25.712755
DD44	32.033302	2566.1108	107.42476	161.33884	127.19625	153.5705	26.515034	33.73328
DD87	26.24039	3189.262	118.10754	187.10979	331.77246	161.28333	11.352476	66.913345
DD2	28.801043	796.67755	70.79551	1453.6056	113.83362	160.05399	22.691637	14.731303
DD3	43.12212	1238.5284	121.3456	309.83786	28.90653	265.49603	3.491903	25.540909
DD89	67.85523	1231.522	43.766556	321.95453	17.595743	153.5576	1.2309368	26.851814
DD90	96.74405	1133.272	501.11307	36.988564	39.057705	115.59288	108.28944	39.21546
DD4	54.55678	1002.9166	63.887184	129.2355	24.558884	164.73792	16.48887	39.49823
DD47	35.63143	2098.594	58.91209	86.36469	237.20615	301.82977	8.247027	55.435238
DD48	45.279324	990.13324	218.99759	1244.9242	66.79028	472.2336	11.786688	30.849974
DD49	27.185368	1050.4426	137.69295	135.53838	15.842877	362.49542	4.15806	37.686802
DD50	34.472023	977.09576	203.23679	105.83669	37.37935	347.86554	22.987282	39.908703
DD46	41.048874	927.1817	72.85192	372.58514	36.104298	169.6418	13.444899	47.87197
DD6	47.529133	688.80316	66.40299	1165.8151	51.152504	126.13139	4.89427	29.385805
DD7	60.392914	775.8437	134.80225	238.8533	26.47352	125.44009	11.559337	30.168425
DD8	37.33716	1049.8313	63.645557	22.3544	27.95208	115.80543	6.084624	42.89824
DD5	34.0843	1130.1517	57.69467	45.336742	39.68965	144.60175	11.191583	48.456604
DD9	35.437763	968.1499	77.76129	39.563305	35.687145	141.10924	2.0688045	36.238426
DD103	218.21152	4206.0107	286.9679	981.2369	264.6422	440.91748	31.142849	60.398777
DD101	312.41452	4049.6885	166.63618	2248.1428	174.73532	694.3739	13.019275	51.14909
DD102	165.88925	4150.087	301.11847	1020.8904	254.9567	498.76672	41.699078	109.34804
DD105	193.99623	3981.1458	290.98248	2102.603	229.09035	865.80084	27.843267	58.580555
DD106	298.7234	3745.671	276.9047	2157.2612	155.65572	619.4843	29.099749	59.42306
DD62	22.149326	2195.785	320.58942	1736.1498	63.39531	207.9117	29.449976	55.74178
DD64	31.753437	2476.7896	493.51944	439.27078	93.22326	384.8203	65.52703	61.14099
DD107	209.14592	1750.3892	376.0316	72.33273	136.94174	247.63065	51.228867	57.192966
DD108	282.43747	2979.0513	747.86194	301.60672	177.33533	410.14032	110.2493	54.945824
DD63	280.80154	3101.4963	781.92426	299.47723	165.03018	375.07602	89.60181	56.845497
DD23	160.87717	2782.4822	158.37894	44.11616	119.87642	282.3376	36.887386	74.98033
DD24	196.55481	2587.6387	137.02318	209.96062	190.89992	367.88025	50.6033	68.801056
DD110	309.12213	1894.7765	508.86014	38.559902	47.76559	419.69348	62.64947	60.81837
DD22	179.6446	2880.145	249.2588	278.92328	69.23688	421.16345	55.446724	69.66254
DD109	321.22415	2385.422	281.27502	23.98188	70.01532	447.15857	32.816193	90.13058
DD65	290.58383	2494.266	493.90683	41.758064	36.12546	414.38303	100.92715	70.45616
DD66	193.3625	2596.7976	63.63943	53.704697	138.80211	328.7877	13.39625	80.8088
DD67	163.63838	4185.954	157.63997	84.30685	253.80833	349.7643	51.540802	99.60133
DD69	219.19902	2174.161	434.87387	53.53569	50.965984	357.77942	74.84758	52.490166
DD68	352.05695	1923.4247	49.344143	32.16368	42.682697	306.40097	2.009609	64.97879
DD25	168.95062	2510.0715	130.89812	40.167736	140.21426	342.41766	18.50794	100.63613
DD28	235.3197	2003.5239	909.4714	32.912983	61.664185	460.60242	145.77307	83.05406
DD29	358.37354	2517.3806	1006.4047	35.046688	212.94484	352.57993	180.5885	67.499115
DD27	37.0885	1704.5419	80.22005	33.09592	237.13446	354.52313	12.112456	68.080376
DD30	180.04674	2719.094	112.98959	27.136417	150.64577	308.09195	1.1806095	90.68913

On-Line Supplementary Table 1

Animal	1386993_at	1387025_at	1387027_a_	1387033_at	1387063_at	1387065_at	1387082_at	1387100_at
DD81	4918.377	24.566174	460.1181	2556.068	65.14988	282.59805	500.39008	1.9977851
DD84	5572.701	12.9287	384.15768	3087.1335	81.76462	251.54463	3.790116	2.0963767
DD85	5319.7495	20.819426	442.87183	2139.6619	68.60418	240.29263	40.962406	18.494473
DD86	251.29102	23.213629	656.5476	2969.5676	88.806915	0.7190942	17.54493	1.0957737
DD83	4523.113	9.200297	369.33353	1669.8293	70.86631	294.2617	2.6989102	1.8179289
DD41	1576.4177	79.9891	826.32886	2183.7063	48.358414	89.74969	13.593804	1.3508877
DD42	483.80676	112.11198	828.1249	973.5315	58.190845	25.168797	22.291512	4.1156163
DD43	332.4311	10.906636	936.6108	833.17786	44.14632	43.788372	1.8053969	4.6029286
DD44	317.95383	3.152493	733.5372	395.3787	64.279	38.09846	3.8953445	20.092007
DD87	410.92056	97.47997	901.3764	1619.0404	60.55301	19.655874	8.713349	5.6838703
DD2	3442.2573	19.947475	664.7388	57.771507	58.1618	167.53737	1.8850305	6.551963
DD3	912.6281	13.934378	730.2271	359.82007	64.60571	24.85459	3.7933981	8.076782
DD89	613.06213	6.937406	695.51154	168.4896	51.9318	44.505863	1.2819086	1.1465094
DD90	33.747707	12.416018	647.1835	478.97064	45.499092	0.936899	13.355004	99.65732
DD4	263.59827	12.958864	1889.7863	246.19601	56.840183	2.863905	4.428126	24.34895
DD47	187.04454	64.845215	551.18524	623.69885	164.16815	0.9040739	112.27929	14.826896
DD48	3737.774	31.041485	415.5299	140.8216	114.7084	213.69041	361.79007	0.6941333
DD49	426.80658	11.652976	560.7346	7.734426	98.29369	29.81139	78.15065	3.8252044
DD50	217.73236	5.6728826	683.0557	6.003388	117.39368	2.4936967	77.3048	11.371389
DD46	851.94696	4.2394443	511.12103	18.356747	38.347622	79.59242	113.18396	5.1873856
DD6	2060.2314	10.492997	518.09595	8.370589	62.22805	180.84639	4.994356	5.7531776
DD7	464.232	8.504986	606.5615	11.411118	59.071384	40.2966	1.7442888	1.4901689
DD8	2.3153315	3.3171542	586.0505	34.71556	48.95981	1.6889201	3.7583017	1.1330439
DD5	75.18849	6.151595	821.6498	23.354532	63.13592	1.8876481	2.182053	4.099992
DD9	32.248543	17.976812	1079.5127	26.625656	71.602	0.5051656	1.5898548	1.1288342
DD103	2630.8618	3.365735	256.32108	1758.4558	110.99877	47.141235	58.948692	1.3836604
DD101	5133.058	15.528183	133.03297	742.5213	105.43987	165.63652	43.125275	1.0820336
DD102	2833.9612	6.12785	223.05128	2072.2432	114.12813	69.23702	201.89835	3.0674617
DD105	4921.5566	2.8512273	152.27228	2027.1362	103.05103	109.59817	45.54928	2.0358005
DD106	4524.686	10.304789	133.07794	606.0021	110.4971	111.70795	226.27007	3.6265323
DD62	3132.5312	14.60427	233.05975	1380.2861	103.33648	144.3629	9.036858	22.054403
DD64	881.8843	9.840002	420.98178	1416.8557	126.97546	89.296074	1.3909459	86.30082
DD107	82.62689	4.246986	338.894	587.65497	156.74205	18.000254	2.892618	43.876026
DD108	863.95386	7.5959415	301.35382	566.4263	153.23569	11.196737	4.9842987	113.12333
DD63	926.7213	6.219427	262.58115	532.1272	141.17238	18.416836	3.0154333	127.74485
DD23	25.024849	3.012285	311.22015	1242.2904	125.29937	1.896065	10.001365	34.409054
DD24	474.44235	13.833365	292.95752	210.43652	112.33006	8.324988	0.8839567	37.832977
DD110	10.174487	4.9809175	300.36044	375.65683	121.84079	50.079674	1.2508365	136.98286
DD22	578.86554	1.2808068	298.74628	878.5077	124.18482	39.22946	1.2997437	70.50057
DD109	3.6768298	10.068725	336.9193	624.9012	107.97846	11.923242	2.747248	22.015226
DD65	30.672087	9.567457	285.6039	1357.8425	105.78174	3.208565	3.0703092	132.47752
DD66	66.16905	42.70224	262.3769	772.7157	159.19409	0.876803	2.4691868	19.89546
DD67	257.30093	13.729261	256.09006	99.2033	140.70018	28.552528	580.9469	0.6108608
DD69	57.038097	11.429302	316.42035	1252.0583	132.93259	0.6833491	3.1075482	158.04128
DD68	40.55929	6.2673864	325.4259	129.47058	123.6016	0.472451	42.215664	2.63013
DD25	2.4448678	56.796104	306.81622	2342.8616	147.46822	0.5076324	4.2888174	1.8782295
DD28	19.069305	4.710059	254.62021	649.13605	114.14794	1.022204	4.4833	268.79837
DD29	2.0398784	42.08589	221.49731	1965.4585	135.61296	0.7423969	3.82557	305.01215
DD27	30.67806	28.70872	293.88812	800.1411	170.77354	45.092102	1.7245978	1.3608098
DD30	2.4588375	62.955055	310.48935	2648.8958	162.74388	1.4162076	1.8776199	10.0773

On-Line Supplementary Table 1

Animal	1387118_at	1387139_at	1387146_a_	1387174_a_	1387305_s_	1387313_at	1387391_at	1387459_at
DD81	97.569855	25.625692	202.98312	162.45338	8.451385	235.75008	0.6890488	11.630143
DD84	7.683199	0.4471524	201.91129	167.26726	1.1482352	290.98343	3.9965727	10.562231
DD85	1.6791669	127.68619	212.09416	195.68654	23.146523	287.11835	1.7443252	9.397301
DD86	6.0586157	22.484318	343.4285	458.8618	2.1273727	262.12415	10.084901	3.1294668
DD83	1.0231774	4.1617355	216.19736	337.92963	1.3356583	271.784	12.229086	8.725115
DD41	0.9828355	1349.7041	257.16104	1878.8123	2167.2375	157.41368	3.9630494	11.339376
DD42	5.102498	2919.3096	294.71863	3524.5095	3817.485	128.95612	16.557753	7.027351
DD43	8.492786	20.061243	200.524	143.63437	27.907486	128.98203	13.997759	84.40063
DD44	11.200522	41.13261	225.09721	206.81004	0.8272488	147.57748	10.541204	59.71494
DD87	3.7112927	1302.975	254.14801	2486.9392	2223.282	212.90335	2.150626	8.653687
DD2	1.3793751	277.30344	307.80246	702.70624	673.90875	370.133	10.01331	35.97335
DD3	10.882303	26.924583	380.41827	68.1622	7.178878	234.3177	10.96847	11.556033
DD89	5.334937	7.197121	377.49408	69.09308	1.8414189	188.43016	1.4049636	10.850205
DD90	13.72291	14.6086	458.9969	73.902885	1.3559313	195.82117	7.7197695	20.727184
DD4	10.173242	323.53802	453.34955	66.83359	5.439054	196.8614	1.4075966	12.255778
DD47	65.90442	1653.9409	300.01624	2562.6067	2631.0781	183.08641	4.113598	8.220753
DD48	162.10008	383.40045	272.64706	886.9824	1055.9263	413.04877	3.1100223	3.5288093
DD49	43.835175	14.094914	381.61746	37.682243	19.992298	198.6439	7.782482	7.142849
DD50	45.905083	16.485106	418.29532	59.720383	1.8919123	190.5669	16.661966	12.644444
DD46	85.662674	25.409903	378.3019	26.619612	1.3249848	324.98007	6.517876	15.524255
DD6	3.2504237	0.9911135	348.1153	4.769307	6.5273232	367.72632	5.8486266	37.992645
DD7	3.899526	9.658237	446.5818	34.09276	0.8089554	307.24466	13.503406	23.060642
DD8	3.8773904	3.6003997	427.11597	27.423088	2.2425892	195.77025	3.7304518	10.3678
DD5	7.1519837	17.475447	380.68262	22.943897	3.7638798	206.6861	2.3877783	21.356901
DD9	0.7631157	2.9142318	431.46158	33.84592	1.3074621	233.08495	5.9000797	16.13711
DD103	17.275322	6.2906733	174.20737	249.05421	2.5347767	137.6532	36.414116	16.227285
DD101	12.387644	8.054844	86.05465	282.963	0.5625665	155.80223	10.886127	9.163687
DD102	84.129845	5.2962637	126.95839	164.94385	5.7409415	113.81129	33.182186	41.330273
DD105	30.674683	24.137554	155.65558	191.02055	0.623172	126.95767	57.146675	5.708637
DD106	66.576546	2.9135485	94.88629	253.7009	1.638633	210.26933	23.673542	21.906197
DD62	6.550469	6.3213563	72.09347	276.3543	1.543068	158.13893	18.033525	22.733164
DD64	0.5192622	4.128705	200.3624	135.4615	0.9967432	112.61934	23.270538	30.2252
DD107	4.6698036	1.4972173	169.61432	102.86746	1.2853678	45.573055	20.934683	106.7036
DD108	3.484185	3.659874	161.16661	258.94598	3.3224616	60.970722	45.0833	54.069588
DD63	2.1124616	1.3895329	167.24474	275.5363	2.6873415	65.711044	48.19857	48.47334
DD23	0.9559545	1.3148783	199.79503	245.41916	0.4364366	57.1601	15.103491	34.470932
DD24	0.3393497	1.5656052	238.6405	195.63951	0.982561	109.48773	34.841324	19.109745
DD110	5.1935883	5.53984	213.94778	134.49063	1.1947646	93.820984	28.324812	17.416668
DD22	5.583175	1.0973403	184.40231	142.78532	0.8430206	76.59507	41.454147	17.42043
DD109	4.548344	2.2627087	144.61697	150.14299	2.7279687	61.973785	30.724752	12.370905
DD65	4.959632	13.256929	151.88283	44.211544	1.7400827	55.369175	53.540714	22.645153
DD66	6.565239	994.7071	219.48172	1323.175	1587.9795	26.347105	87.91404	11.185599
DD67	210.58028	72.714966	112.24497	147.4758	10.959302	71.78649	8.239857	10.109778
DD69	1.6509804	0.598982	234.81143	64.04432	2.2950315	59.488865	59.33666	20.869064
DD68	21.86644	11.645082	148.6188	152.88211	0.9904225	39.602367	25.66619	25.94036
DD25	7.050374	1115.2551	160.43619	1077.6953	1178.6101	47.47054	13.55771	19.07665
DD28	6.9804707	7.079855	137.7496	47.536892	1.6285399	70.19208	32.248363	16.96138
DD29	11.20216	773.2068	218.68623	1051.247	1349.7012	60.46087	33.644363	17.092785
DD27	11.932569	800.07605	188.02383	1257.875	1511.4264	66.93675	31.069529	68.22068
DD30	0.6835501	1186.3823	161.63605	1064.614	1385.1531	44.963566	2.3580928	12.27033

On-Line Supplementary Table 1

Animal	1387472_at	1387508_at	1387656_at	1387704_at	1387710_at	1387749_at	1387768_at	1387796_at
DD81	57.535583	170.08553	153.03766	31.379671	363.72546	112.27349	3570.9387	357.7609
DD84	28.499365	3.1805542	134.30272	26.40799	343.32455	95.96057	4159.102	390.8307
DD85	7.3125257	23.569052	255.32225	27.702791	479.497	2.2275496	3478.9	549.62695
DD86	14.962449	4.4435616	406.73883	20.57491	532.7407	2.5652976	210.86409	1090.6991
DD83	15.377603	11.148948	144.19572	35.09108	631.2711	28.854574	3240.1604	375.47095
DD41	3.6462772	2.589901	249.82196	25.117632	379.27487	2.4212096	1342.4735	403.73215
DD42	17.74874	10.561497	259.37198	11.36523	476.30325	1.6715745	524.6767	431.86606
DD43	460.23657	13.093636	273.63315	4.756722	373.5866	476.34747	489.69086	365.2599
DD44	368.23978	9.127663	100.78969	22.521383	601.22125	340.76013	517.3041	202.37465
DD87	25.36959	20.72046	300.51138	5.537617	611.40216	19.004358	505.52188	511.71487
DD2	249.56398	10.133612	157.46487	13.628917	124.88068	299.1401	3072.5745	248.18452
DD3	24.576239	13.484242	230.53433	15.928125	216.0006	3.682774	984.1158	329.68634
DD89	21.925955	9.675217	117.72688	15.357212	342.14264	18.359577	654.216	198.8887
DD90	44.751213	14.224417	123.66081	3.0045712	117.5695	11.671016	48.22508	259.5032
DD4	53.28426	6.282097	489.06476	9.331683	104.28974	10.999036	277.96494	967.75885
DD47	38.727997	67.759254	223.05466	14.579886	436.47754	1.2329686	288.25302	209.93753
DD48	15.340375	128.39438	118.28291	13.99078	157.30258	3.638929	4123.332	186.04474
DD49	35.36405	31.324635	245.15079	22.680307	178.99199	17.351788	449.917	288.02267
DD50	7.931153	48.127983	279.7649	16.009546	125.10259	4.7599945	423.86197	369.61484
DD46	20.11056	96.4888	85.44133	20.614492	243.82455	7.091842	979.211	75.13333
DD6	143.49782	9.127827	76.47827	24.074375	131.41649	143.30028	2039.0696	120.46744
DD7	62.126095	4.385348	107.19624	13.030996	155.44984	64.26302	549.04944	206.80138
DD8	33.615368	3.3916173	146.73119	23.323792	167.00327	2.0801983	18.684937	218.36821
DD5	49.534214	5.715665	206.68938	23.128244	140.7398	12.022636	129.44803	337.00217
DD9	64.391624	9.735276	352.0943	24.200768	158.28183	39.400566	55.420105	512.78925
DD103	85.494865	12.910868	382.23724	13.076813	432.08093	81.313065	1996.448	2027.1464
DD101	51.036552	9.603017	242.61739	15.369008	419.32025	60.699253	4449.3604	1443.6514
DD102	310.29294	47.664894	173.6299	23.160038	408.0006	228.35036	2322.6792	857.78925
DD105	24.625507	29.008589	235.89667	26.379566	348.91074	15.767229	4412.9756	1276.2644
DD106	78.40677	61.062363	197.5571	15.544581	142.73938	81.35053	3939.953	1254.6411
DD62	59.731815	1.9594294	73.09879	52.211365	459.79367	46.32022	2422.3062	140.74908
DD64	90.64057	6.972977	133.45152	61.316	431.07657	162.72717	1299.1447	203.73973
DD107	343.77988	6.2615986	105.33717	50.496174	298.3029	668.8818	238.80171	251.70323
DD108	200.47798	7.320915	144.80727	43.90979	856.0502	259.5904	887.6671	308.2023
DD63	212.32643	13.73505	141.68506	45.26019	806.42096	270.7692	856.36615	308.96545
DD23	125.9019	1.2476919	43.748592	37.452873	375.90643	119.0397	145.72198	79.61051
DD24	47.28395	1.3645283	71.32636	31.004637	516.92395	27.254295	596.44745	96.23372
DD110	33.540554	6.309642	81.37418	63.79233	529.49274	12.251431	199.55763	145.89821
DD22	15.577018	8.09835	43.51205	31.485777	479.31287	2.3295233	886.90955	72.06451
DD109	28.361275	3.8128417	42.283585	41.84073	460.93066	1.5705575	72.26374	80.46005
DD65	73.03749	11.884635	97.79154	17.955275	196.3008	33.618286	178.697	107.67237
DD66	30.70174	0.5870353	79.03365	20.280985	508.2798	25.53062	127.78696	162.11479
DD67	12.232834	270.44534	16.397888	26.987717	411.08353	3.2235205	700.93994	19.769018
DD69	77.60088	1.0457472	52.39061	38.3365	325.3542	57.992947	94.37673	75.54754
DD68	96.58193	19.150003	43.860714	55.344944	569.56573	83.99892	171.38991	61.199474
DD25	31.707096	1.7506195	72.22539	26.434025	234.37555	24.5349	41.937893	98.14378
DD28	22.708548	1.5482768	152.75656	51.683353	418.774	2.7424376	51.1384	197.68626
DD29	27.179964	1.1228856	92.1089	46.98935	678.7308	8.178913	16.742075	130.47655
DD27	226.6015	2.303381	60.846283	53.80723	446.53705	465.6219	296.87418	79.05319
DD30	20.646416	6.3268814	67.25111	30.797184	246.29865	29.79843	36.19491	82.05702

On-Line Supplementary Table 1

Animal	1387808_at	1387827_x_	1387839_at	1387902_a_	1387906_a_	1387938_at	1387992_at	1388046_at
DD81	109.85633	0.2174647	20.415903	824.207	219.46371	1.6170665	42.559616	5.170314
DD84	137.18085	0.8588801	9.265862	921.33777	210.90804	0.6689181	32.764523	6.423269
DD85	113.05302	1.4895493	17.486624	57.192856	202.40805	7.731152	42.074696	7.818098
DD86	169.93396	5.0160904	19.544176	41.882217	272.69586	6.7469053	35.65861	7.1043277
DD83	107.2542	3.7851303	14.724697	178.43294	136.33192	0.923772	75.29899	10.277965
DD41	106.57216	2.7707994	29.210562	139.88663	242.32184	41.0548	85.64596	29.25317
DD42	110.32131	0.720453	11.760749	72.00056	124.68369	105.16412	84.88817	27.418703
DD43	144.9312	2.5279272	46.4811	5397.7563	159.41011	0.8726434	77.88368	56.901756
DD44	149.03922	5.5557942	39.02772	4395.9727	97.57892	6.86469	117.22895	65.06596
DD87	115.3454	2.1443038	23.806158	484.59464	172.99606	71.478714	112.00539	22.138975
DD2	104.52771	3.4713328	29.927927	5228.1597	108.47871	19.592047	75.31417	31.632349
DD3	91.81336	3.3827586	26.958698	361.16763	91.98897	0.8909901	89.684746	42.181557
DD89	111.97215	0.0527581	26.552683	354.56323	86.73184	2.3629854	130.11452	42.290176
DD90	100.71118	3.017044	16.730547	251.59883	117.32281	13.62464	85.94701	19.957317
DD4	113.60073	2.3470674	24.3187	220.21736	74.173355	2.3046556	109.11082	25.91343
DD47	125.29264	4.9016967	31.33251	194.26018	95.84126	71.45461	134.64984	10.294142
DD48	77.24427	0.2455923	23.16047	139.63481	122.6723	33.565277	72.76407	25.653538
DD49	104.83124	0.0273368	30.488068	1260.9532	59.80611	4.3574867	105.86002	40.366383
DD50	131.12625	2.3053055	39.52343	367.0777	118.83582	4.340007	100.8806	31.87315
DD46	123.83166	4.1918263	34.8294	288.12653	116.99873	1.6456292	165.9568	34.74546
DD6	115.39803	5.1850996	44.77457	4982.828	88.128624	1.6472824	81.14365	34.861168
DD7	130.11736	1.5762961	28.960522	1631.2314	108.01842	3.4519565	101.86958	24.15458
DD8	130.54535	0.9056808	26.495794	270.08994	65.609535	6.5317154	78.583824	23.107744
DD5	146.99126	2.7594721	19.1189	864.3669	39.05657	17.044651	109.51107	7.8685117
DD9	119.84676	0.0221085	25.987455	1222.827	50.071995	0.5512793	104.7496	4.054409
DD103	58.771854	3.1678288	73.65696	552.7142	47.216022	18.38181	44.208374	28.738298
DD101	33.446327	4.275697	19.900469	548.30194	45.793247	5.7988524	35.78833	52.549416
DD102	67.73113	6.4114738	32.75925	1818.98	42.449383	9.9244375	13.126189	79.570564
DD105	40.067806	5.974773	32.58544	84.278435	41.13875	2.8842645	4.6985097	37.07178
DD106	34.93005	3.7862096	27.19364	867.1227	51.06756	1.2165787	5.0341687	23.585066
DD62	41.109203	5.084622	10.533593	1362.9712	29.173046	0.5438913	59.94074	62.011456
DD64	55.964188	4.15738	73.415565	1623.5165	30.557716	3.704556	111.48562	71.827156
DD107	91.10833	6.1761312	25.681175	5559.856	27.564503	1.3462244	46.209488	89.46942
DD108	84.647095	7.134626	108.6617	4560.7705	25.57022	7.1341844	21.508354	75.03887
DD63	90.56218	6.777625	110.59319	5177.5547	29.931427	7.2584286	16.7312	73.18056
DD23	68.85995	7.7935457	119.20773	3191.577	51.05064	14.883188	68.742744	61.470898
DD24	59.62746	5.5603895	15.748925	797.7076	38.13579	7.5382004	92.9329	52.985886
DD110	48.739166	5.414454	23.081673	475.876	36.20231	2.0727248	3.7212162	51.593857
DD22	67.16687	5.954063	81.20389	541.54877	67.777054	2.361741	217.55774	69.7963
DD109	54.70031	0.0252763	91.21626	584.1937	120.34384	10.514357	119.48221	93.1139
DD65	76.53875	3.7609823	3.013065	634.57855	26.975368	18.717766	4.1230636	70.38636
DD66	62.66408	0.4452377	100.86541	604.44794	27.37391	63.172554	200.73721	59.577892
DD67	39.819286	3.0975318	26.375067	71.37735	71.985954	1.9578905	83.8552	80.42879
DD69	59.718647	3.3145745	26.614098	1571.436	28.017447	14.878304	114.87633	89.468956
DD68	61.257088	2.6169882	14.992391	3673.8845	67.91526	11.163528	221.41936	82.89033
DD25	48.94147	5.2077255	149.4478	2557.1372	37.351223	82.90446	4.650079	65.08399
DD28	64.07905	3.9632394	89.656136	183.78543	18.041504	25.997879	25.520569	89.204506
DD29	62.55902	2.7979383	21.456324	302.01413	31.820496	47.98674	83.70555	61.335434
DD27	64.968796	6.330682	261.4364	5895.2725	39.358776	51.55107	68.753624	68.62941
DD30	45.915638	2.486439	160.92554	2597.748	51.63364	60.563267	4.8223534	58.140297

On-Line Supplementary Table 1

Animal	1388056_at	1388071_x_at
DD81	3.2505007	599.92944
DD84	1.2797594	532.18567
DD85	7.180577	532.046
DD86	7.810616	615.3638
DD83	4.7477183	490.46097
DD41	1.3984989	723.63165
DD42	6.8736563	813.7779
DD43	15.69651	1157.8948
DD44	3.1828759	925.0807
DD87	5.110799	830.5114
DD2	0.8705483	854.40857
DD3	1.1415375	963.4561
DD89	2.2723217	694.5008
DD90	2.4133034	682.2966
DD4	8.545933	939.45355
DD47	7.9123025	784.3435
DD48	6.628512	881.1711
DD49	10.534639	1137.0812
DD50	10.367904	1222.5288
DD46	2.234524	949.9395
DD6	5.0387135	891.4545
DD7	1.3641329	860.6582
DD8	5.060523	848.6588
DD5	14.631377	899.76483
DD9	2.0147102	953.5076
DD103	23.154612	2872.1194
DD101	8.725255	2285.0815
DD102	9.7373085	808.9451
DD105	0.6658021	1529.4438
DD106	12.850105	1948.7455
DD62	19.335962	2671.5369
DD64	2.875245	4443.63
DD107	32.961456	3901.693
DD108	29.501148	3446.8196
DD63	27.23431	3689.5332
DD23	7.9946156	3383.3003
DD24	15.344646	3157.9302
DD110	18.20041	3639.6174
DD22	11.182065	4096.4263
DD109	23.434288	3711.0476
DD65	1.4751681	4587.611
DD66	3.6486716	4846.166
DD67	20.241476	3220.7866
DD69	7.8760424	4154.589
DD68	27.994755	3782.0361
DD25	24.700464	3714.4478
DD28	13.896221	4573.7593
DD29	4.010978	3458.6265
DD27	3.7070415	4685.384
DD30	28.13021	3504.3647

On-Line Supplementary Table 1

Animal	Strain	Age (Week)	1388072_at	1388101_at	1388102_at	1388108_at	1388139_at	1388155_at
DD81	GK	4	29.6669	29.204266	238.3114	2494.176	5982.6294	86.38284
DD84	GK	4	60.424713	19.365566	282.8189	3258.3662	5931.591	40.897564
DD85	GK	4	46.036644	22.625242	260.30685	2756.6294	5851.8604	25.715601
DD86	GK	4	37.37327	3.483685	392.35043	4611.179	441.76837	20.698713
DD83	GK	4	47.958214	23.644728	303.82578	2732.5342	4603.9116	17.037008
DD41	GK	8	18.474083	18.087646	481.85272	2893.044	2132.431	22.002779
DD42	GK	8	30.446075	11.318294	598.556	3357.882	743.1884	21.931818
DD43	GK	8	2.877744	8.848504	327.38156	2488.799	817.07324	117.858
DD44	GK	8	16.481936	19.24204	401.4388	2752	773.20667	117.27507
DD87	GK	8	34.63204	1.4805354	500.48114	2836.7146	751.5029	21.677097
DD2	GK	12	12.916053	4.6241074	162.45995	198.9148	3564.907	62.33066
DD3	GK	12	1.9155805	12.35033	169.7989	380.98676	1010.8391	20.861322
DD89	GK	12	1.408467	7.3187895	230.21544	541.0633	941.5873	32.22995
DD90	GK	12	0.9021858	9.021306	208.93236	599.6738	30.83586	205.9069
DD4	GK	12	1.3931257	20.109236	185.21928	376.67328	462.48288	23.772257
DD47	GK	16	36.53271	23.236198	380.2889	337.18192	171.83319	29.455412
DD48	GK	16	39.92112	14.598277	165.73334	190.93402	3458.6968	54.879314
DD49	GK	16	8.437958	12.412204	138.57703	162.53883	381.5733	38.908855
DD50	GK	16	45.462524	9.99631	138.26596	244.6225	341.3579	72.402374
DD46	GK	16	8.344169	6.6486754	145.86101	168.93066	912.64484	32.7475
DD6	GK	20	2.4821503	9.063289	115.63525	116.64785	2294.866	34.983738
DD7	GK	20	8.014337	2.1346114	161.93982	154.56935	665.2843	29.574911
DD8	GK	20	1.6209633	14.091447	164.54233	176.86569	29.628994	16.541069
DD5	GK	20	4.8298774	10.079407	167.76146	138.41591	123.87054	18.236837
DD9	GK	20	1.8302585	13.953515	182.7792	175.16302	50.616875	29.936125
DD103	WKY	4	4.6277866	14.889602	440.2974	4838.641	2369.9597	65.62099
DD101	WKY	4	37.914062	22.11727	588.3148	3372.3667	4831.61	41.989124
DD102	WKY	4	1.4886111	22.253342	519.611	3643.0134	2619.395	92.12386
DD105	WKY	4	40.329533	40.32936	409.0023	4106.9824	5025.845	31.64571
DD106	WKY	4	9.517521	28.384665	439.9196	2789.7952	4451.2144	46.626877
DD62	WKY	8	1.6267914	19.813671	346.36893	1505.2833	4057.5732	85.087296
DD64	WKY	8	1.9489342	17.077444	370.77414	1398.8822	1474.2253	211.87558
DD107	WKY	8	1.5124195	10.578075	343.04868	831.9793	315.60617	154.22102
DD108	WKY	8	1.2256659	38.334267	364.92776	2214.591	565.534	283.88156
DD63	WKY	8	1.4580394	40.044907	362.07083	2257.4026	568.3046	290.0535
DD23	WKY	12	1.3651987	18.772413	388.6196	1953.4369	177.49315	90.33391
DD24	WKY	12	1.6123575	25.101698	620.4156	2170.615	406.34094	67.690285
DD110	WKY	12	1.3052773	23.237398	333.1009	899.00104	69.89819	236.37674
DD22	WKY	12	1.179324	22.745094	503.4999	1464.762	1025.9008	180.54329
DD109	WKY	12	3.7601967	26.747103	543.06824	1437.699	17.960663	87.12291
DD65	WKY	16	1.1215447	29.543613	464.45157	885.6775	135.80867	293.46548
DD66	WKY	16	8.381688	36.467094	579.23206	1062.2257	47.86111	23.547855
DD67	WKY	16	23.70158	8.764109	479.07294	2683.6885	110.58972	99.06212
DD69	WKY	16	1.444008	31.735464	337.05304	891.3421	33.24102	351.2131
DD68	WKY	16	0.8335915	38.283222	446.78143	738.00726	212.65625	43.73858
DD25	WKY	20	12.717579	34.92853	373.78275	535.7605	7.2118382	30.425726
DD28	WKY	20	1.9781736	36.187897	393.3798	430.0646	27.536333	331.97607
DD29	WKY	20	16.162329	48.81106	460.15985	1087.3341	8.26593	398.91104
DD27	WKY	20	23.059492	34.418106	319.5704	462.82358	31.089895	60.230072
DD30	WKY	20	16.885775	28.26841	368.9263	585.45844	7.648305	28.771236

On-Line Supplementary Table 1

Animal	1388157_at	1388166_at	1388181_at	1388199_at	1388200_at	1388202_at	1388203_x_	1388233_at
DD81	68.1476	509.74747	53.002262	8.913445	3149.92	10.463224	39.68743	176.58606
DD84	95.68772	394.38257	63.544415	10.356187	3737.4006	9.718919	82.39809	44.34774
DD85	77.53406	83.528404	7.6270094	24.638695	3375.6304	18.623087	59.257317	24.489668
DD86	136.30806	98.92627	2.060919	9.41884	208.37878	18.733042	80.1471	59.948895
DD83	81.09882	186.12381	14.498325	1.456831	2679.357	22.483374	82.157104	37.609844
DD41	101.04396	160.23083	1.5422108	1.3654542	1170.6992	20.535461	66.72813	42.482025
DD42	92.94637	187.0589	4.025698	1.3637809	371.3204	28.009485	91.89077	53.14069
DD43	116.18472	2428.046	1395.9102	7.967271	288.3814	21.343143	109.47548	44.122032
DD44	86.140755	2168.8792	292.46933	30.03039	275.2815	18.44885	98.59581	49.5544
DD87	77.943886	175.44504	2.8004036	8.153376	255.33672	19.988781	83.9185	42.716164
DD2	69.51699	1684.921	236.66272	10.03726	1764.2836	4.7792435	72.98562	58.885315
DD3	115.94846	117.92477	3.6839807	2.1808243	446.91803	22.106026	57.7412	313.53412
DD89	41.791164	134.21457	2.104965	2.9782896	250.09367	17.863096	57.131874	66.70832
DD90	31.74568	101.7501	10.391715	124.77301	4.207918	9.655708	50.858253	38.947643
DD4	26.588858	137.06778	9.206545	103.85393	104.45551	27.140875	62.29952	64.70885
DD47	159.17126	73.03194	1.5609016	27.166637	140.43954	8.736011	51.42379	99.991905
DD48	125.48076	95.01501	2.5107172	2.105628	2173.939	3.5837083	88.873764	148.31732
DD49	121.17793	568.44666	9.779574	13.522625	295.36487	9.155128	74.95673	112.31742
DD50	141.16658	101.2002	6.0482707	44.197567	211.93604	14.966529	41.52644	578.7357
DD46	84.7395	69.78139	4.8537292	1.2693598	436.09598	12.291178	52.924706	47.392117
DD6	53.038834	1035.8022	88.319916	8.488699	1044.3889	14.295108	72.73272	39.61551
DD7	25.938496	429.36896	45.270405	10.210179	207.42032	12.407455	59.96819	30.805988
DD8	66.44623	68.94787	6.7868886	1.4747071	2.928465	14.427221	82.64326	32.653645
DD5	52.112377	124.51832	10.104587	5.102879	52.335457	1.5659823	63.294174	31.46445
DD9	56.4791	312.2365	25.40102	5.674725	5.916914	9.464841	47.60198	25.198175
DD103	192.31433	427.33725	45.889362	1.9403406	1828.0059	75.97086	181.63197	39.895275
DD101	205.63733	251.52771	31.172129	4.514087	3931.84	54.047157	160.88466	44.685818
DD102	233.55782	1174.5236	165.1113	8.662132	2131.254	36.94727	92.28491	135.35397
DD105	155.49077	131.265	13.971509	5.7861176	3646.8457	58.570568	112.07831	185.26483
DD106	184.15126	322.29517	44.731453	8.80931	3527.7769	57.826992	214.02464	136.64862
DD62	87.56146	478.97656	41.258347	38.548153	2131.6028	76.5142	139.29095	166.79897
DD64	145.52664	702.37115	119.21789	95.97456	475.14282	139.08134	173.87926	65.56037
DD107	86.81365	3266.9326	547.73615	57.2274	89.60069	113.09544	212.54861	90.79564
DD108	174.36641	1835.2983	193.47466	137.72733	586.41034	126.49748	184.49237	292.72485
DD63	180.54163	1888.1487	196.01535	141.84924	624.78156	143.23654	246.03535	322.4428
DD23	172.8391	756.90564	109.33595	47.52893	29.3438	103.15686	142.343	186.19388
DD24	168.6599	270.52527	9.694503	46.64098	326.28653	101.13882	188.35632	176.30312
DD110	190.73369	123.35813	15.729384	144.66739	1.8825238	100.36981	216.54233	58.100502
DD22	227.8269	127.52165	0.842281	96.88544	431.66818	100.42783	99.497025	230.98608
DD109	123.61639	116.79395	1.3391407	39.393047	1.4541621	92.50955	109.72363	21.89102
DD65	95.095085	322.88696	18.077717	168.12427	5.3791456	134.98839	170.10957	146.39142
DD66	142.9137	274.54337	3.75222	9.997331	37.664097	146.41516	184.08638	31.399746
DD67	115.54204	130.88588	1.717905	13.992215	152.32904	101.10486	118.39217	83.97443
DD69	187.30667	589.83386	31.272558	195.18213	28.699526	99.438126	142.3286	294.31396
DD68	208.94623	513.91736	73.8769	14.832006	53.917522	91.37924	138.0136	140.91338
DD25	86.59635	696.79297	10.019767	19.10267	2.2761521	94.02013	111.17735	67.06908
DD28	181.15895	114.09709	13.510775	244.96829	38.153072	95.43804	88.03746	59.96099
DD29	173.91447	119.2552	16.445223	303.8914	4.016517	79.16546	211.35274	48.55447
DD27	192.3122	3308.0225	421.23737	9.517658	13.383834	126.14539	163.09904	76.43952
DD30	91.10457	712.33795	16.498293	3.8596103	2.5050988	81.85387	72.05565	61.190716

On-Line Supplementary Table 1

Animal	1388255_x_	1388272_at	1388275_at	1388277_at	1388396_at	1388433_at	1388451_at	1388485_at
DD81	25.751293	7.660189	129.67807	32.673462	74.50264	20.436726	1399.0626	218.06094
DD84	6.3329115	49.458855	56.451103	16.555208	93.23287	42.937843	1398.2534	238.1165
DD85	19.80752	2.74999	1.8349807	15.859158	80.079124	38.49521	1434.7449	184.35278
DD86	7.7912064	10.758132	17.186054	23.620296	68.62809	40.66063	108.05615	273.96463
DD83	11.454116	5.3358264	20.250864	21.043242	85.173325	48.243267	1307.9052	259.16385
DD41	29.99256	24.486715	34.180958	15.843298	96.18086	33.218807	683.63763	334.34448
DD42	43.040432	4.846747	40.46744	7.167012	100.79515	32.255184	496.4161	546.67816
DD43	165.76698	4298.739	1002.6086	164.68593	142.96977	39.424747	185.93391	123.42319
DD44	189.00137	2213.0527	902.3653	158.64896	79.857864	65.61418	257.6551	140.48372
DD87	70.05618	95.74803	51.363422	16.952114	107.83752	37.564743	382.2964	314.8197
DD2	176.66772	3045.2244	618.0751	128.74173	64.51846	40.57331	879.5967	261.37256
DD3	83.497154	54.313396	52.88768	17.116947	96.88624	51.776543	208.61575	236.77048
DD89	71.224915	40.34147	70.48652	23.977709	35.359856	58.437263	318.72256	264.6406
DD90	74.99201	22.064098	56.961002	20.854485	33.80893	191.8288	73.192825	260.20355
DD4	39.81006	12.047142	121.29899	14.553777	29.577736	38.858955	116.38778	250.56264
DD47	83.023476	20.116516	53.228962	19.325373	97.54878	82.94158	160.00868	487.42346
DD48	63.021313	15.554704	40.228794	11.881401	150.04732	79.6854	804.5246	341.0682
DD49	67.53164	77.83682	50.688248	21.92434	116.63258	103.99374	184.45872	299.33273
DD50	78.3681	28.584684	51.896603	2.159582	92.71793	153.71922	111.25099	266.55966
DD46	95.63596	21.584425	50.361233	12.171533	72.86406	49.62466	317.18338	205.16609
DD6	85.08981	2259.6384	383.89957	115.35087	72.7076	41.314873	773.291	221.03014
DD7	90.7895	225.00209	98.57076	41.43386	30.196957	39.825417	223.25606	280.0556
DD8	54.333076	35.76573	56.864376	11.535024	52.934937	25.3556	61.954105	310.66452
DD5	61.684803	75.26627	64.557495	23.618374	49.0168	39.092342	92.06154	313.32776
DD9	47.06701	109.09866	167.42995	29.412327	47.539684	69.32342	64.757576	339.2205
DD103	152.68385	6.3345404	184.30959	67.43006	191.76015	107.36075	473.78333	61.317703
DD101	32.576443	0.8409426	81.14415	41.125427	244.61055	100.5437	1396.0624	76.77078
DD102	124.175	96.63734	441.91223	157.43326	201.41391	42.196888	549.6526	55.00973
DD105	38.50754	9.565443	53.3166	30.07401	135.97	21.982008	1359.1123	106.53488
DD106	45.445618	114.64031	159.66576	80.78467	264.6365	68.89861	1114.317	91.566536
DD62	34.43714	237.45995	148.722	35.675766	130.69418	143.57983	911.0277	63.61076
DD64	275.48593	459.80063	246.64735	123.54055	203.53915	265.1531	431.6988	111.43526
DD107	160.0573	4080.8997	1015.1713	1687.6322	183.77258	165.16739	117.47775	31.468946
DD108	285.49487	2175.3452	429.25113	484.202	129.30962	518.6772	169.92908	71.74806
DD63	325.35468	2196.9907	432.81363	493.03543	142.00668	520.0344	164.08014	67.276245
DD23	367.4471	243.52339	270.21356	283.44107	241.95154	110.52895	75.9353	106.86369
DD24	58.900974	58.385723	99.2174	41.72648	241.08878	146.4052	94.849915	72.705154
DD110	70.46609	114.49901	86.05193	19.758236	295.90054	356.18164	251.09445	58.4565
DD22	225.3817	133.99176	32.312744	13.363123	213.57831	304.90576	201.30267	68.15769
DD109	198.50269	76.652916	39.079563	25.868172	100.06487	159.75397	86.0472	65.106705
DD65	0.3595839	178.81706	103.80109	44.727055	215.1023	492.544	55.976727	72.23433
DD66	242.1429	109.62388	59.0255	32.56986	254.15688	91.17556	145.20422	101.16666
DD67	50.450245	12.913012	27.240732	22.149734	73.19655	71.296715	91.19459	69.61982
DD69	89.04818	538.64325	138.11409	53.923748	173.1155	543.38104	54.867718	69.41742
DD68	81.33696	1098.8927	208.58647	166.13252	159.79732	63.565395	66.9743	62.53545
DD25	307.19955	773.41705	94.334045	59.097805	231.0154	72.56174	105.05417	236.30255
DD28	129.5393	45.455223	34.82588	25.267632	136.41936	673.5506	63.750828	65.606804
DD29	62.630127	52.10162	34.72655	27.557669	85.74383	707.57666	80.01511	129.00064
DD27	503.91693	4067.9346	634.9739	927.4074	243.29167	33.31495	182.36456	107.4548
DD30	284.23013	838.7488	79.93051	69.089455	232.50453	72.56284	120.92613	246.47243

On-Line Supplementary Table 1

Animal	1388578_at	1388603_a_	1388613_at	1388741_at	1389007_at	1389118_s_	1389142_at	1389160_at
DD81	283.5053	1514.2506	1039.5537	2967.7705	226.97412	18.160051	517.0155	106.09127
DD84	276.3762	1721.7025	1122.229	2745.2825	197.43643	8.070124	610.64703	118.07014
DD85	235.32848	1457.5045	1034.1149	2597.4185	208.88223	15.435855	547.1413	117.50073
DD86	252.62553	1670.8298	1110.7877	98.730774	251.35522	14.376817	1047.3761	241.2283
DD83	263.69162	1924.0999	1189.8481	2313.137	218.2744	14.448441	620.81165	67.57293
DD41	252.17267	1335.52	1137.7407	1072.0276	189.29453	15.895382	648.0244	284.76987
DD42	222.68335	1283.9174	1059.3862	270.2814	228.53105	11.676843	610.63947	247.37627
DD43	172.68884	988.79724	919.8065	371.33624	253.40488	12.723228	574.18634	179.99097
DD44	236.71114	1152.0409	866.5573	395.7207	263.29803	22.966726	690.778	118.3785
DD87	245.53978	1300.3196	1138.5607	390.12924	340.49768	14.165691	767.9171	238.03302
DD2	226.98193	1319.5714	1113.2567	1997.255	167.40894	6.8429675	520.33325	187.84198
DD3	176.50822	1095.8586	909.27344	561.8562	324.42444	13.901639	749.23846	172.79704
DD89	220.1651	1181.7216	962.07495	680.2214	286.98035	12.457792	709.93036	181.73764
DD90	241.25922	1111.6232	957.5684	83.77769	166.50781	88.08513	756.577	192.37993
DD4	203.34485	1141.8164	1541.7325	274.99088	198.25955	14.301406	666.5465	839.6304
DD47	154.23581	1191.8761	812.4985	127.41149	425.29117	10.707785	641.1588	123.61726
DD48	190.04013	1390.1112	849.8243	2411.2715	315.0003	9.464258	596.8461	102.42142
DD49	161.32845	1025.2593	749.19257	348.35202	299.9997	20.196852	746.4712	173.34012
DD50	185.26195	1064.3302	873.5385	225.01337	333.87332	25.802538	682.6797	202.63855
DD46	202.52557	1108.4025	853.6833	721.88885	231.05682	4.687737	696.7811	72.18564
DD6	227.65323	1371.622	1037.986	1640.5046	170.92065	6.9092474	520.61414	98.29673
DD7	244.93512	1124.1963	1046.9792	592.35767	157.74153	13.943371	596.70233	157.98332
DD8	225.24052	1154.9198	1016.7866	27.039433	214.61855	15.640429	642.89246	178.1985
DD5	214.61717	1089.8893	1069.1611	122.74406	253.18864	9.281021	683.30896	259.32812
DD9	188.5503	1071.3069	1259.213	39.687386	265.17404	13.582237	617.1095	368.8684
DD103	93.22446	758.59625	617.09644	570.35004	80.78312	15.213846	235.32375	322.75497
DD101	102.73147	729.03064	676.7913	1709.4939	130.4754	1.2328882	206.03015	185.76123
DD102	79.67215	614.3097	417.6477	700.26337	48.34466	10.132207	259.15216	96.04352
DD105	110.34522	604.1075	577.8544	1924.0531	124.07715	10.849793	266.51093	172.07253
DD106	96.378265	755.5097	688.12994	1511.0743	87.70757	6.3885555	255.2608	197.75256
DD62	130.90916	496.41348	590.6835	1888.2152	91.99774	30.848719	334.23715	109.57069
DD64	198.99072	759.8964	756.0864	899.31213	72.23593	61.697395	275.88647	161.10095
DD107	97.9664	290.99573	373.29044	219.16707	52.340878	40.82751	232.20181	115.88487
DD108	95.55662	735.7736	572.0612	335.7031	126.83474	118.63918	335.86423	58.598
DD63	100.95719	772.2487	560.9668	308.31403	117.69865	105.34251	291.0817	82.82323
DD23	154.6019	762.20215	531.4542	113.3508	143.68419	36.917747	355.97162	57.398563
DD24	173.6312	478.42288	414.66095	207.2917	161.4879	37.286934	359.85504	78.087524
DD110	114.89041	344.63306	410.0021	632.3791	98.497345	79.75233	320.82098	78.51555
DD22	180.54103	417.93472	365.62595	438.17764	160.4557	58.930416	363.15912	45.283623
DD109	118.37733	376.71613	414.877	132.50429	186.81216	19.5049	417.52454	60.109486
DD65	194.95512	446.7431	395.89517	134.57582	185.69727	132.6594	445.1995	94.48356
DD66	120.12086	430.76273	410.38654	41.597385	132.67047	22.30253	334.8812	62.585472
DD67	148.07832	413.47113	377.69034	222.14029	114.04527	6.4663925	458.45865	8.530969
DD69	113.33314	400.7864	330.05142	57.776657	114.37037	127.52277	431.12173	57.653946
DD68	117.56285	410.93802	369.21713	90.7545	148.88779	9.428461	409.4762	46.32511
DD25	121.62164	646.1363	542.1032	6.5417914	161.96411	15.742757	428.3892	52.28117
DD28	97.67978	438.16425	443.13968	5.8642354	146.0443	155.48529	414.4873	82.927055
DD29	100.50057	354.36996	328.8114	14.887004	162.73921	187.63568	354.0951	59.21162
DD27	109.04578	455.03714	370.4131	286.6582	89.681625	7.5471883	290.68143	49.051395
DD30	121.70098	616.485	550.12964	5.6252017	195.35385	6.320074	396.9221	80.19132

On-Line Supplementary Table 1

Animal	1389177_at	1389270_x_	1389369_at	1389425_at	1389436_at	1389486_at	1389725_at	1389734_x_
DD81	7.3429494	322.88553	53.146755	32.61078	1089.5074	300.2659	53.27266	93.50381
DD84	23.260096	38.10766	50.407383	35.34022	1066.9637	336.2393	45.422436	92.89433
DD85	3.2169976	68.71	66.12181	24.1123	1036.3951	452.6972	48.14656	77.84124
DD86	2.4259892	30.623676	70.15238	29.837292	103.20264	744.46967	44.776085	130.5813
DD83	17.962858	32.537224	55.468758	25.129549	959.61414	323.97324	30.950565	87.937904
DD41	11.561288	26.373802	46.939144	31.95976	774.73364	871.9596	10.957129	164.28786
DD42	15.046025	39.439957	43.884476	59.584404	236.38823	963.7298	34.179157	156.86302
DD43	14.718123	18.634558	51.339157	176.4595	226.97261	783.80536	4.4356236	129.98363
DD44	25.112358	85.68588	53.925858	137.54007	242.00783	384.5457	15.396009	117.19347
DD87	13.779921	64.36491	49.52604	39.95503	261.9972	861.9084	29.312786	156.7014
DD2	3.9950302	12.682241	42.87996	121.03117	1264.8654	706.4659	6.6408286	140.2212
DD3	6.5541606	161.96729	29.082602	42.154213	301.9534	661.2465	17.716578	216.3208
DD89	6.55897	27.147907	27.73409	23.96951	371.47684	627.2597	5.1930995	253.04071
DD90	24.929905	179.33298	54.761314	26.30489	71.177574	763.1359	11.086619	307.70782
DD4	18.529184	38.752434	42.41486	24.5646	191.09383	2162.8633	2.4316566	354.08493
DD47	9.73278	358.88263	42.533405	34.698257	135.29474	488.67368	22.780119	203.05226
DD48	1.7974005	955.3876	31.421381	37.450195	1233.4316	300.20993	23.400656	226.22704
DD49	1.0122446	288.84674	37.784023	32.68767	252.24516	505.1067	20.698675	278.15784
DD50	20.4145	361.45407	56.18309	54.894783	142.97534	632.30505	4.247066	343.92593
DD46	8.029888	499.5948	40.009365	26.247389	498.46857	331.04413	16.018076	284.1135
DD6	6.2066417	68.31667	52.169834	69.99373	1058.2798	362.98666	10.905397	290.9181
DD7	6.6554275	11.429791	38.963745	38.185596	340.3825	606.0028	2.6400938	273.4538
DD8	6.0099707	14.275005	28.730852	40.070812	44.071938	798.2824	14.498049	345.89706
DD5	17.21525	22.454472	69.26558	37.00825	128.41965	699.9949	3.938152	257.19974
DD9	6.616782	16.38144	39.99663	40.50741	46.22047	1363.3734	4.024544	289.23535
DD103	1.5291473	296.59943	82.06799	54.068806	223.12634	715.3408	81.24378	734.07336
DD101	7.3142095	25.989761	134.46365	65.36055	522.26086	465.92746	64.01844	117.0146
DD102	1.6668907	9.099696	89.0183	151.17946	212.9985	325.10956	81.7907	1214.8763
DD105	2.808739	26.540764	97.98259	64.50985	306.65103	561.4117	97.69879	574.1523
DD106	2.0870478	19.76072	113.69646	76.06925	343.8092	507.15366	73.428345	134.02672
DD62	8.035783	27.30775	83.18442	67.85933	834.4905	310.88065	54.475445	712.43225
DD64	47.983795	35.20258	106.2168	109.24679	143.95331	559.0992	50.578243	377.48907
DD107	13.620411	21.570263	93.01744	228.78752	142.69635	596.0835	50.030632	998.32465
DD108	28.7874	23.113045	88.70527	155.20123	153.48477	375.4721	82.21886	804.9029
DD63	52.24455	21.99092	88.35089	153.20633	164.34012	373.38443	77.463615	752.30524
DD23	33.645885	12.635447	80.44818	91.489265	91.8599	239.98958	69.60912	944.491
DD24	20.941652	27.740108	74.68944	54.38618	201.30661	259.0098	73.77848	957.58234
DD110	38.05573	7.7090983	74.273636	61.212452	161.88982	314.19437	44.83152	911.14667
DD22	34.45913	17.913849	97.29742	48.84571	214.37718	175.38847	48.619984	876.4804
DD109	30.393076	18.225872	84.437325	48.848793	50.71337	204.2314	74.19599	840.7368
DD65	38.67563	19.03121	109.619	49.31314	100.0081	326.76047	41.292957	692.49603
DD66	37.43998	26.896122	138.18169	50.774837	68.26685	261.619	50.46227	342.10782
DD67	22.97058	3415.6846	75.509415	35.654358	109.04868	90.261154	97.4559	887.51807
DD69	56.906174	72.419624	91.56606	61.493015	73.82259	254.37878	23.238081	1111.1664
DD68	19.081478	383.4914	89.62142	56.13493	99.771675	236.43336	67.778854	1036.4424
DD25	9.413042	11.684831	103.33543	55.34214	41.03419	308.05814	46.729996	931.2977
DD28	64.98032	11.640443	119.93487	53.283115	51.96114	424.77524	28.263483	281.79727
DD29	76.56584	40.55692	99.59495	44.25783	29.756752	288.1885	37.735783	839.44965
DD27	5.5082464	9.843381	175.31183	154.98149	128.20033	436.98828	63.77897	72.39897
DD30	17.780619	13.781117	86.01897	50.691906	67.67532	298.4502	45.650463	950.96796

On-Line Supplementary Table 1

Animal	1389905_at	1389935_at	1389996_at	1390050_at	1390100_s_	1390235_at	1390481_a_	1390491_at
DD81	418.77914	69.20902	91.446846	37.753044	625.8602	40.28384	32.610455	81.11755
DD84	327.68735	65.51787	99.39265	46.150677	429.17526	41.17097	26.049105	60.252064
DD85	256.59818	84.80705	66.857414	32.483017	391.86002	31.880606	31.027733	72.94919
DD86	202.61508	63.450554	132.35986	34.714733	425.32858	20.809334	10.81983	72.628525
DD83	263.65433	55.161663	88.41195	30.902002	363.7076	47.59774	36.004005	56.00872
DD41	347.01602	37.435497	112.96759	27.82006	380.99594	20.958906	17.913458	45.59892
DD42	182.06203	65.54333	98.57671	33.787106	445.89194	17.706207	7.976518	39.98762
DD43	214.77617	71.467735	128.89667	156.82814	519.0374	14.204851	46.262444	32.832638
DD44	235.38046	61.64244	109.45996	133.29652	549.453	17.56094	29.475744	47.104908
DD87	232.52985	67.941696	83.92053	32.703136	368.4914	15.152172	16.34455	35.719185
DD2	260.09113	58.37623	74.03113	88.950905	517.7852	17.097467	3.7789974	39.264694
DD3	273.00363	58.61931	112.76656	32.402164	299.86325	17.282167	9.017593	52.069973
DD89	584.37665	59.65745	88.57901	31.720491	560.82306	17.726515	7.981023	57.161003
DD90	764.6644	40.518787	153.81322	55.631763	720.1836	26.055538	15.691388	59.10309
DD4	575.6721	67.9651	118.45689	37.813274	385.44815	32.52032	2.9054465	37.4014
DD47	213.60281	89.98021	97.742226	27.055758	432.8008	11.767064	9.636308	38.9663
DD48	371.7222	73.881454	90.50762	18.630863	405.21912	13.963288	1.7743573	43.3512
DD49	390.73398	73.29912	99.2741	44.395847	418.32162	17.882483	3.5532782	31.685123
DD50	371.2852	93.09016	91.4295	22.424347	446.5953	35.180946	1.5130055	57.423016
DD46	387.36664	41.695076	87.71784	35.990623	410.2709	11.346961	1.4016873	51.261425
DD6	476.31964	42.92291	111.53558	65.642	457.72186	27.539629	2.6936748	45.87321
DD7	721.88153	42.621876	132.99161	49.493263	613.4471	22.636536	1.6700476	40.766895
DD8	516.2948	53.02794	97.39752	31.199472	379.1328	19.757254	3.9065437	37.919975
DD5	362.6926	49.067616	88.940475	30.96835	264.69785	32.34119	2.0421264	40.693554
DD9	433.18857	61.290688	92.50265	47.46671	279.76932	28.355066	3.3436053	38.232773
DD103	176.44402	11.633572	89.02831	69.88262	192.43036	51.069298	29.620955	11.345482
DD101	273.06055	4.7272635	60.561237	59.812263	272.0088	68.45572	24.008247	23.425589
DD102	141.24806	24.772907	56.194614	168.52457	208.90573	45.913975	23.355398	22.703947
DD105	195.18748	16.994661	55.57792	51.556145	196.87933	49.6111	30.713171	27.287785
DD106	306.05722	21.889622	89.98374	65.446266	196.0961	57.266525	35.83611	7.8723564
DD62	226.45355	5.5705223	56.657917	57.73171	182.62314	39.5736	4.9805408	20.937723
DD64	235.43912	18.802427	64.9617	92.11859	189.74142	34.556274	4.027046	12.970262
DD107	129.3189	6.0922585	40.451134	239.62524	232.4042	37.016277	37.37658	31.354742
DD108	174.44653	29.52977	41.218204	130.79066	238.30836	47.018787	21.453613	21.749573
DD63	156.2083	21.659122	34.95765	126.9204	231.36032	42.361607	30.852932	21.69942
DD23	116.40087	30.600107	35.30825	76.927986	118.78195	47.505592	20.242558	14.125883
DD24	200.3076	15.039276	35.08723	61.769917	94.16898	61.912647	18.425737	19.842642
DD110	184.77678	24.780436	68.38826	59.66765	125.67376	46.837982	13.572778	17.326073
DD22	130.29752	17.57194	21.978598	70.902275	121.4987	41.435917	3.7173388	21.740795
DD109	167.07817	14.462087	95.45646	65.16166	198.99773	53.1135	11.205921	32.27253
DD65	190.2994	23.861277	42.823048	87.7122	125.54062	29.582817	11.333993	44.530796
DD66	130.61517	25.57453	29.776543	65.26359	187.22952	40.681343	15.314974	38.52784
DD67	126.21844	24.143553	23.859358	67.96302	133.90398	49.47262	16.042538	52.09482
DD69	208.86885	15.209031	78.06886	108.43816	173.6528	39.026108	2.8631148	52.25777
DD68	180.67584	21.768261	87.53029	77.353294	143.11981	52.895023	5.2002997	30.56938
DD25	221.31851	7.301617	82.722824	61.70619	244.30885	42.816795	1.2010379	25.051601
DD28	178.72661	34.561485	91.19153	97.43656	166.87202	58.361366	10.659738	14.256693
DD29	208.57109	8.633107	96.96134	113.06966	130.37874	58.184147	2.320344	15.492233
DD27	131.92265	9.683222	51.261414	162.94484	273.4208	49.239594	14.041892	43.179802
DD30	166.57343	15.947011	90.4803	64.10899	257.62283	44.003887	9.888668	23.866142

On-Line Supplementary Table 1

Animal	1390554_at	1390562_s_	1390585_at	1390654_at	1390722_at	1390790_a_	1390813_at	1390835_at
DD81	93.55976	43.54369	95.382774	14.962533	32.330498	26.72567	37.573	6.3155384
DD84	89.33949	40.75355	70.704254	19.377028	23.406416	23.049759	36.10826	5.1334467
DD85	72.35002	43.353573	88.59656	24.613771	33.186604	21.25994	45.369034	8.363069
DD86	25.810415	32.54567	105.63245	37.88943	15.714033	0.5914656	14.400331	2.241137
DD83	105.33097	27.762487	99.14672	15.274619	35.671894	17.913452	36.50992	6.586068
DD41	171.57484	82.81321	127.71876	26.438498	30.242346	28.071638	30.89188	7.4625316
DD42	509.451	36.26063	141.49922	9.483605	19.588938	29.582613	27.22216	1.4931742
DD43	16.47444	103.66509	116.08051	22.04781	24.200657	29.084366	32.22862	2.6048908
DD44	11.664357	72.35044	138.892	41.453598	28.82383	23.527555	32.571114	2.0107145
DD87	231.03018	39.685535	155.14258	21.700783	27.792385	6.8813686	34.416737	1.6564548
DD2	104.72901	68.33067	159.1057	33.127625	21.8058	23.992834	31.203962	11.136775
DD3	20.371723	54.32266	240.4733	46.40869	44.87327	1.1185949	14.87365	8.713248
DD89	28.187284	66.374664	201.71915	34.5932	49.591183	1.1364663	41.800587	1.9020067
DD90	15.649834	111.98628	289.86713	38.600163	106.18095	12.1895	21.06318	2.2521722
DD4	20.818834	78.784424	304.9097	57.137093	75.96208	1.3303857	21.917336	21.873617
DD47	224.82831	39.981243	149.53601	28.1077	25.757034	23.143745	37.40268	27.770512
DD48	213.34383	44.931786	121.00056	6.868053	22.00218	12.9869	62.816303	0.676718
DD49	17.808207	66.88078	181.32254	43.036537	17.329876	3.1105056	17.574759	0.9053345
DD50	17.485796	64.612175	211.48021	37.33538	40.125786	8.903944	21.568127	3.1714246
DD46	14.819908	83.88578	184.94765	38.52762	30.396221	8.257604	26.898827	2.4818585
DD6	42.531445	104.12508	158.33653	22.783888	45.818096	19.773708	42.276413	5.5126386
DD7	16.071636	122.87119	220.73657	24.262623	69.85159	5.069882	22.88412	4.668799
DD8	5.3703413	86.45867	178.67372	30.81077	64.18768	4.774729	22.34496	7.440042
DD5	7.672872	92.54286	167.10292	38.812824	62.574406	9.646975	15.945924	3.0191371
DD9	8.478736	61.794086	169.74472	36.21084	55.797535	4.6514435	17.758966	2.9159632
DD103	42.09374	33.185627	61.262737	46.699234	20.384266	25.264275	98.35329	26.250374
DD101	61.313145	1.5873342	41.019787	52.44125	1217.7903	34.560173	148.7614	64.257164
DD102	30.090687	107.55101	48.642895	48.7955	37.43089	22.054827	60.72597	17.69064
DD105	79.73329	20.107983	78.724304	42.860966	1069.305	46.296753	178.03874	40.620605
DD106	60.059418	4.7083645	64.60971	43.48199	1251.8683	30.98922	146.17519	22.080988
DD62	31.056517	50.614784	102.13435	56.422234	531.64246	27.39481	64.5389	40.102745
DD64	8.023216	13.533958	73.62702	63.915268	507.85153	14.454891	56.479492	15.091441
DD107	8.27196	119.3887	105.64874	55.216408	537.41846	21.548258	49.183483	18.358633
DD108	11.697271	53.739666	92.986855	78.62879	591.0933	12.615301	59.52798	18.401693
DD63	12.673678	63.65232	89.468346	68.61061	581.616	18.494202	65.90615	22.90901
DD23	9.780327	73.03545	142.30754	69.10884	412.13486	18.841274	58.026222	20.693785
DD24	9.657	50.70169	119.00269	67.917816	505.63742	30.632854	48.703587	18.985037
DD110	4.0764556	52.428337	78.57897	69.3256	929.00604	26.126062	80.72196	24.268757
DD22	11.364732	77.59559	90.90244	70.61741	499.07153	19.038675	55.361645	18.407015
DD109	4.350529	51.034233	101.59946	78.48912	25.777029	11.704866	63.389366	4.418582
DD65	9.450093	4.962208	52.10872	89.25417	670.5264	10.7024	47.148685	22.172865
DD66	164.665	1.4118996	85.22063	71.56927	639.63367	16.44526	22.332384	19.299648
DD67	9.179291	29.039341	53.347355	55.451252	647.2147	27.769657	26.1265	6.657087
DD69	7.4814725	55.73968	54.715908	81.60714	49.74278	13.206544	43.854618	17.970854
DD68	4.7716637	49.881947	64.54013	66.89224	1186.5564	2.043322	61.233562	11.277585
DD25	204.10364	70.11851	125.56442	72.12474	670.04553	30.694008	51.532246	14.327083
DD28	0.9117357	15.307098	72.37261	90.7688	1217.2067	10.805862	17.716995	26.994967
DD29	166.82777	35.83912	61.334503	75.09182	37.01568	17.32784	61.75241	26.127113
DD27	200.1567	17.059467	92.099724	58.87889	667.9319	32.410336	41.76668	14.143616
DD30	176.0184	68.14574	90.12292	78.37675	695.99615	21.964668	65.50517	6.4017572

On-Line Supplementary Table 1

Animal	1390890_at	1390929_at	1390971_at	1390979_at	1391011_at	1391018_at	1391072_at	1391089_at
DD81	19.349216	64.07749	2.1499503	56.312057	11.611056	61.19445	84.34809	38.26976
DD84	13.965431	49.768024	1.149528	58.571358	0.8808553	41.43377	64.63889	34.157608
DD85	9.808538	49.689144	1.0237054	57.790325	13.665177	77.49092	11.708229	50.43584
DD86	1.4196655	81.45918	1.0341065	42.54454	15.028234	165.9233	1.9272811	35.28667
DD83	5.2516356	38.247253	0.5274577	35.389313	5.405404	59.610058	29.428183	41.586205
DD41	0.7624636	59.292145	0.9508781	56.6208	1.3231213	194.55212	7.494144	58.754925
DD42	4.468961	49.077114	10.700004	44.92289	17.014889	185.34065	10.100632	78.42216
DD43	111.6094	55.5969	3.8158426	41.29049	3.0682185	140.81903	308.4104	70.60023
DD44	73.329865	50.52313	1.5830482	48.673588	1.3709698	78.10058	230.08125	61.911766
DD87	4.1852007	50.30873	0.4705469	41.943806	4.164548	184.98563	10.560061	77.372375
DD2	62.835995	45.373196	0.9404234	32.039593	6.6952906	119.52853	133.7457	17.71722
DD3	1.3958582	70.87568	2.9201384	42.239555	5.24905	128.18382	15.652926	43.77799
DD89	10.691752	105.4945	4.022881	40.858635	3.6248229	140.77194	8.319941	67.0211
DD90	15.268215	102.92854	11.655056	39.897552	5.5607977	169.63725	16.210505	70.747
DD4	1.8797548	65.79978	7.2529044	30.393835	1.703331	508.12457	26.167936	52.33293
DD47	8.846289	55.47382	5.2441287	37.081116	10.817819	78.83676	4.689885	36.585346
DD48	0.6347429	65.26714	6.2647543	37.958035	9.580544	61.974594	3.1048284	24.998186
DD49	7.5247936	80.32928	11.407709	35.89927	4.773969	77.25956	9.237877	32.256786
DD50	5.6467166	99.33626	20.78068	49.02721	1.4045025	116.24241	1.5325934	24.166636
DD46	5.52616	65.778206	13.802532	48.88564	4.7633104	63.41326	6.4110465	22.085213
DD6	20.53297	51.047432	1.3120826	46.98377	7.7947507	76.844734	67.35801	14.118012
DD7	17.80872	64.217415	12.775381	48.9717	9.164577	115.93171	37.405094	24.93014
DD8	5.1816726	42.89553	9.544438	50.559635	7.492666	120.72678	11.879921	24.34136
DD5	8.795037	41.631992	8.198602	47.923	20.469595	155.0098	18.670254	21.391027
DD9	12.637174	41.149586	8.671559	40.48635	1.0311503	250.02396	13.627473	24.367037
DD103	45.639515	38.26176	0.4217697	16.483871	19.29104	150.96179	46.1261	45.789295
DD101	12.487616	26.150496	2.9667857	18.535995	1.5010289	106.45854	40.03422	30.808155
DD102	72.666245	16.88936	3.100249	18.338404	25.495646	61.977585	114.43045	14.542331
DD105	2.686025	40.915684	3.0717247	8.7361965	21.53587	92.756454	17.0279	17.16792
DD106	27.574205	45.834793	2.99482	3.0313604	28.996756	111.44836	50.39833	32.446823
DD62	10.33414	41.358944	0.957368	30.301702	10.680877	75.424614	36.819572	18.050014
DD64	29.110014	15.952127	0.3872555	19.583008	13.529516	128.17212	78.79838	12.836765
DD107	72.847946	56.31061	1.6228771	19.674051	13.995125	88.24292	272.79575	8.636948
DD108	40.261887	27.202118	0.9687663	10.265981	19.965162	68.79366	133.58968	21.617561
DD63	49.751038	27.958042	1.4615486	10.796925	25.420635	69.95434	147.35713	24.539507
DD23	29.214258	55.651672	3.6093688	18.475042	22.171793	50.661705	72.93975	21.227705
DD24	2.4396384	42.09529	1.045169	13.268192	18.80826	46.769573	13.587668	39.24905
DD110	7.2431192	42.42156	1.0314043	21.458233	30.067434	61.847706	10.722442	17.321217
DD22	0.7905391	33.72012	1.077418	10.913136	9.007641	28.44084	1.1122047	21.11966
DD109	1.8071305	108.69466	1.0464311	12.799747	27.427927	43.65191	15.85964	26.467371
DD65	14.422249	21.295347	1.3112057	28.33173	19.20082	72.29275	27.80681	15.576011
DD66	2.0359538	17.950994	3.414151	19.227604	7.703085	73.83405	11.596595	22.308378
DD67	4.3424973	42.959064	0.8671883	27.56083	31.530277	19.445618	8.337364	6.5167084
DD69	24.096527	27.694784	0.6046194	23.327377	17.97111	60.580013	31.578318	18.721886
DD68	22.463327	89.21438	0.5732398	14.351273	8.166801	40.01948	48.747746	12.130178
DD25	9.216137	98.70472	3.456725	20.304668	31.478802	54.957203	20.575817	12.871821
DD28	7.0104103	8.230694	1.789074	17.136621	29.57752	60.747894	15.000289	13.489121
DD29	7.1217175	20.573854	12.583549	16.080942	27.52713	41.12726	2.8268754	14.180288
DD27	130.66814	2.9248056	3.305826	29.458862	22.285213	42.737816	205.45288	12.804614
DD30	2.2809792	86.53636	1.2030239	19.147673	23.83171	60.566833	16.958956	7.83466

On-Line Supplementary Table 1

Animal	1391138_at	1391207_at	1391222_at	1391258_at	1391266_at	1391295_at	1391305_at	1391417_at
DD81	29.065523	5.544226	30.972042	3.1053917	27.053839	14.144192	5.2279115	126.14356
DD84	36.76776	18.848503	17.876064	3.1834908	30.478504	20.440905	5.212442	10.937783
DD85	23.77811	24.237715	3.4891474	11.670619	42.020218	26.1019	8.056071	16.212969
DD86	43.99391	9.636246	5.8131146	2.513555	8.883405	17.460619	12.413914	2.014931
DD83	18.267006	13.929042	17.461548	3.5402	17.463217	11.00292	8.245495	5.5443306
DD41	26.226027	14.883661	1.5536826	8.673344	22.58818	42.728287	0.6853306	6.1259365
DD42	40.490704	2.4796655	10.886703	3.0669339	31.616405	34.627075	1.1146758	6.386685
DD43	69.118126	6.7208405	20.972986	20.863768	59.017967	15.57299	1.1686666	1.2617004
DD44	45.71762	5.7745605	0.8720242	2.3347197	40.665436	17.093267	2.9906871	4.055756
DD87	35.54907	7.892242	11.633416	9.897609	31.552267	59.676064	0.754448	7.449493
DD2	40.237587	11.204384	13.215055	3.1139283	39.81095	13.226613	1.2981515	7.791279
DD3	36.161476	12.285654	11.460773	5.69472	30.676384	20.027988	1.590073	6.484906
DD89	49.98243	14.730338	27.811705	8.950208	22.75999	16.952436	8.842042	5.029288
DD90	75.63183	17.97722	24.85395	2.1461275	34.569134	16.343067	6.502438	12.153791
DD4	63.375767	2.518593	17.913013	3.548563	20.301794	14.164888	2.550701	34.692467
DD47	12.247305	4.9496536	32.813797	12.750231	21.000479	16.21775	3.697639	54.503025
DD48	40.457542	15.907506	60.557613	1.8056169	24.451387	32.5358	4.4954476	104.47624
DD49	27.50334	28.53839	46.163513	6.345184	20.928406	10.851691	0.9497453	27.277136
DD50	65.28582	21.30933	36.86341	35.489197	37.149082	27.648441	0.6875618	30.032701
DD46	45.77704	11.772345	17.906416	2.9093902	22.703306	23.155247	3.2631426	35.77721
DD6	58.979748	6.268652	50.544693	3.2296703	31.44423	20.01839	3.5015285	3.4229498
DD7	66.892815	11.612366	39.05912	5.288996	29.99835	15.560972	1.5990759	2.2257898
DD8	42.26253	4.834948	22.355494	2.8755643	25.168503	14.965479	8.450243	1.5121219
DD5	15.234602	7.6800246	27.611717	2.6989791	21.849167	19.285154	4.9651136	10.558643
DD9	22.321192	11.265754	27.533472	3.4220567	25.006653	14.704988	4.337129	1.3652052
DD103	6.382845	11.625609	39.543133	37.987953	19.636698	6.796765	2.7996004	15.435981
DD101	9.850886	29.243202	29.71965	2.9205043	5.1201415	7.90097	8.758987	15.441952
DD102	7.262974	20.770756	42.710884	70.53205	24.292864	6.903146	3.5070012	40.733418
DD105	2.7884486	26.440899	25.003258	20.042505	4.3606033	10.393073	6.0762362	12.905303
DD106	2.1379387	19.040432	34.6236	2.8400323	4.440633	3.9890263	4.4894805	35.065666
DD62	21.59364	24.28914	23.268341	38.289845	6.5151176	8.725898	25.110308	6.559923
DD64	24.663343	36.957783	12.903554	9.575471	23.274223	11.53082	26.006584	1.3463862
DD107	5.451374	22.909708	14.938199	76.700615	42.54986	11.84245	15.579562	0.9601452
DD108	11.470058	55.188473	23.897636	55.15033	6.1945286	1.9927014	5.2670374	6.764002
DD63	3.388958	55.75765	40.6658	47.456203	6.5320344	18.797247	4.9796176	2.0982132
DD23	17.194487	15.529313	50.14981	57.756123	18.053644	8.899202	22.974905	0.2917265
DD24	17.85945	45.19422	61.551266	45.58328	4.302986	11.420781	9.978773	6.4317846
DD110	31.018776	80.67024	60.988163	57.559544	13.466763	2.1892755	8.350056	0.2660932
DD22	2.8109767	33.642693	49.23797	36.656223	10.478224	11.027368	32.687492	0.7102539
DD109	17.085968	34.84099	49.82717	39.602863	28.644375	10.860665	36.68369	3.1998887
DD65	30.089512	45.89258	8.987831	2.8725393	10.677142	22.706692	30.905325	0.7646413
DD66	1.5859176	48.94256	20.111357	3.3825533	3.3696506	13.298834	9.760797	11.536803
DD67	6.5282145	17.526129	9.005267	31.763102	14.705734	10.553043	4.133262	254.82542
DD69	37.230923	32.898365	12.163478	41.01564	6.169918	13.421318	12.070928	6.2466426
DD68	2.1358929	50.71986	24.337528	44.85248	17.513435	10.210009	65.93989	19.062515
DD25	23.792805	47.52767	38.864876	57.81422	23.947351	10.329663	65.88082	0.9025715
DD28	19.924551	35.996597	25.29433	3.1510115	2.3476057	17.430456	6.2034726	6.558944
DD29	14.721167	99.04074	23.555525	48.281044	16.397982	24.405146	47.418907	0.5240541
DD27	4.8346467	80.30332	34.206802	7.452174	21.144123	6.061003	47.201782	0.4709263
DD30	16.270065	42.3209	46.29586	60.334167	25.999765	17.051352	64.83088	4.575387

On-Line Supplementary Table 1

Animal	1391509_at	1391512_at	1391552_at	1391575_at	1391600_at	1391612_at	1391635_at	1391697_at
DD81	7.422478	18.400122	499.3535	83.46605	196.78087	88.91549	121.87036	19.96203
DD84	4.00435	10.784018	421.64154	146.63666	185.90648	76.13189	110.49117	21.825134
DD85	20.263424	13.427074	414.84152	104.90868	193.18219	12.496528	184.78725	25.967773
DD86	15.875033	10.564629	359.68277	165.99779	240.43611	18.457695	182.11823	23.215311
DD83	5.859921	11.215121	493.61823	125.85289	147.10872	36.79504	125.9969	25.031939
DD41	13.540526	15.0445	305.783	134.72368	155.45705	13.797674	93.6735	17.17656
DD42	8.144284	7.915321	359.66806	95.66824	149.90892	12.396894	109.14963	25.562914
DD43	18.977669	13.194419	372.51663	114.45644	177.67621	310.84726	80.68331	23.1988
DD44	25.418123	15.143739	414.92935	93.64886	151.81865	225.54987	77.03765	21.649239
DD87	12.130785	13.665831	383.22623	141.6195	123.00945	16.481823	123.15395	21.207954
DD2	14.35987	14.715123	375.4243	29.027699	146.19585	243.52298	110.74265	21.38921
DD3	12.780403	12.493692	288.60275	47.93614	180.1615	17.031017	205.95071	21.652346
DD89	9.922056	32.643658	404.74716	43.591724	212.62144	24.61612	234.67125	14.633872
DD90	128.09807	23.696363	496.09305	58.65927	211.1744	17.106339	326.59384	23.816946
DD4	23.204819	24.822374	280.71106	61.95672	148.40993	8.568034	249.65776	12.156518
DD47	9.47231	12.260123	353.2505	56.020683	141.27863	10.313405	180.90129	13.726278
DD48	4.4314866	12.885751	406.23935	35.734848	123.71623	10.971592	162.47943	20.696342
DD49	13.674642	4.257744	282.34247	36.79304	151.91656	7.267126	195.5093	34.710423
DD50	49.938732	14.315305	243.25508	46.43469	206.39081	13.950528	232.53156	16.578753
DD46	2.6099784	9.286769	304.80548	23.552006	137.4726	7.419981	164.39587	24.040968
DD6	4.4743624	17.561533	372.8735	38.92526	123.91225	135.50725	120.65408	23.967615
DD7	1.4009268	22.030518	297.75125	35.263897	162.42612	70.017235	179.2811	20.903664
DD8	5.0735693	17.260044	265.61923	34.669945	125.95732	11.298893	132.18233	21.365728
DD5	10.714051	26.535044	249.19827	39.015358	123.45779	16.064203	121.83118	22.967571
DD9	1.7395344	13.809255	241.87259	29.777718	104.0729	27.922274	101.07296	16.864933
DD103	1.6871902	8.879023	35.306995	179.696	61.180668	48.06927	44.33121	61.014957
DD101	3.4543784	2.2443266	175.59966	81.156975	44.736683	10.966227	37.27387	50.210064
DD102	1.958876	1.2751756	45.9382	149.35677	70.09197	103.66183	70.53028	76.68617
DD105	3.7486079	9.307973	154.38547	109.25674	60.201206	22.160896	54.065735	70.2591
DD106	7.910025	11.347977	31.332457	70.17567	43.83186	18.149546	44.066544	47.06532
DD62	43.10948	8.223862	137.03476	101.94016	43.720154	10.519301	49.60472	32.967552
DD64	93.04909	3.2992525	137.1541	127.54566	58.48139	61.326927	50.077255	66.12539
DD107	55.478897	6.0396686	132.10817	95.23898	62.28112	32.296207	49.716427	85.61563
DD108	132.25351	5.176312	59.42046	211.95828	55.329033	8.975359	63.030552	69.90181
DD63	132.48576	7.891442	52.569447	220.13069	60.94112	14.031286	59.193768	57.697224
DD23	57.20253	9.387172	144.82486	119.25385	41.01446	48.47847	56.873425	70.97388
DD24	45.933258	3.6476355	118.64781	147.58472	33.7823	14.650229	56.157047	51.489876
DD110	166.4385	12.140031	130.96617	129.59686	58.111664	4.562616	70.62975	50.650166
DD22	126.49445	7.7303123	202.43306	99.393654	52.03852	5.211697	55.583107	20.381466
DD109	56.673042	5.833337	283.33768	141.2288	42.473377	11.880054	55.01753	30.371483
DD65	151.02783	7.906045	24.48506	124.62254	63.236485	9.134572	50.68461	61.07327
DD66	4.872947	11.219819	163.36186	145.73567	50.090107	11.107826	61.104816	51.45772
DD67	12.800751	6.7290397	297.0568	70.88196	50.634518	11.546989	60.90263	49.972775
DD69	151.32018	7.3306236	30.498838	95.00994	69.70755	22.002506	62.221607	79.98634
DD68	14.657002	7.7377567	229.7956	45.5436	52.67351	14.828315	52.67358	84.70939
DD25	14.094617	4.154176	321.38885	53.23321	51.141872	4.9412656	81.160706	67.72799
DD28	253.4186	9.58529	262.28674	74.79869	72.8906	13.562572	58.58428	71.3331
DD29	232.77852	2.7671602	296.52808	91.961586	71.97889	6.0720515	83.3231	44.94904
DD27	20.563477	10.469859	248.67168	91.167114	40.531788	121.05888	60.469093	61.65958
DD30	17.717508	3.5061905	364.1334	69.94722	47.49922	4.1394024	80.70869	66.47337

On-Line Supplementary Table 1

Animal	1391757_at	1391793_at	1391875_at	1391925_at	1391948_at	1391977_at	1391990_at	1392026_at
DD81	91.27702	10.667394	20.851124	129.2919	4.9878507	11.855837	0.67038	78.12333
DD84	69.77329	11.139839	6.0132046	127.32848	3.5832207	2.5893707	5.766339	61.87056
DD85	80.97536	9.780237	19.2784	38.807373	1.3723801	1.6745466	7.534939	67.678474
DD86	97.07797	7.5176134	16.598057	29.529291	1.2965026	0.3458915	1.1673483	87.179695
DD83	65.65478	5.1767287	12.317742	38.297398	1.5599213	0.5795075	10.251471	60.000946
DD41	107.41756	5.267208	7.437261	25.495575	2.6086197	3.7137742	11.192531	94.47262
DD42	76.26953	7.8828793	10.953368	41.554478	7.848931	1.7016066	4.8513546	110.94549
DD43	51.524582	1.4584204	9.814622	862.9158	95.73414	1.126168	3.533941	96.35229
DD44	70.71964	5.0302234	7.411008	527.12964	81.680786	1.8643795	2.5620925	52.476116
DD87	62.482635	9.466402	1.2655448	45.86036	5.9473896	0.6123574	7.005247	105.91234
DD2	62.976006	4.7922077	0.6082757	255.371	33.893612	0.3657738	5.7843995	104.03832
DD3	66.18741	6.755731	9.267614	43.439804	4.4961467	5.7204676	5.5092483	80.68182
DD89	120.02433	2.4368646	8.275419	16.6658	12.77017	0.5005462	3.5137799	60.032936
DD90	116.52882	9.211481	9.109145	23.684633	3.0436306	1.9397678	4.7219763	73.79993
DD4	69.855865	5.8298016	10.04788	40.543446	5.2353415	2.279273	9.127249	97.411835
DD47	44.572865	6.494308	5.019569	39.366875	3.4615803	1.9199998	3.4108443	135.7546
DD48	52.906643	8.267399	6.618689	40.800213	2.5763626	6.6279426	0.7984372	147.12024
DD49	78.23146	7.12768	8.519059	44.918552	2.5915785	1.1437942	5.729755	118.11386
DD50	66.119545	6.6592765	0.6597017	63.229195	7.021721	7.371274	9.82802	130.0956
DD46	105.12841	8.123987	7.6763554	18.114128	12.315895	2.4897878	8.491005	87.47404
DD6	101.81709	8.46911	8.30154	154.34712	40.089138	0.4495684	5.8982053	73.555916
DD7	96.46133	13.394252	7.176906	54.07891	14.815458	0.7173243	4.1002145	92.854965
DD8	141.33968	7.961749	5.5124664	27.011065	3.1721065	6.070656	6.0806704	55.481487
DD5	92.86458	0.2553099	14.121887	37.614113	4.021175	7.8923306	1.2791886	57.59118
DD9	79.43253	3.4255836	9.8437605	54.97707	25.070211	0.9856499	0.6612644	59.636948
DD103	130.86897	4.308533	5.7878866	150.46684	20.894701	60.6692	10.586943	34.622173
DD101	107.86447	3.9093328	13.514051	65.37395	2.94665	41.74798	13.076283	25.325802
DD102	128.61809	1.4064524	9.6288595	408.23236	52.840652	48.357204	20.691313	30.250496
DD105	182.31396	7.768472	14.428753	44.93769	9.250002	33.45369	16.520449	38.742428
DD106	148.3448	3.3282113	13.033811	140.37856	14.996806	42.638943	11.813204	34.907093
DD62	231.3451	4.5993876	18.090479	75.79347	10.392418	42.773354	14.495534	17.944223
DD64	273.852	4.260981	23.61727	137.09482	27.41747	41.483925	4.0801764	25.246138
DD107	249.95941	4.287532	16.130068	421.6875	78.46804	101.13274	22.977844	30.997025
DD108	177.97748	2.1244657	24.5823	306.35205	54.57376	50.68226	15.180017	28.35859
DD63	174.95702	5.4207177	23.224955	305.45612	55.450974	48.108265	18.944193	23.961136
DD23	178.69054	4.701482	19.927515	121.74206	56.841763	73.07283	15.116375	26.776253
DD24	237.8109	2.3300686	17.708447	44.54126	17.80878	7.3004084	7.938181	33.665833
DD110	343.57352	8.088371	19.0522	54.209255	15.070413	98.10772	13.244885	31.704977
DD22	178.77437	5.4033227	15.003003	17.089474	2.3730357	3.4051516	15.224745	26.533903
DD109	182.713	4.1086507	13.275667	39.782215	21.455769	114.10001	10.373612	42.18072
DD65	256.1618	4.9290433	15.319305	75.32768	16.255865	112.22754	17.32365	43.57799
DD66	121.53874	5.2820773	19.432484	64.94008	2.185941	38.093452	11.94634	45.792316
DD67	105.2775	3.2909985	13.809513	10.523146	1.8391854	93.887665	9.499672	21.290066
DD69	241.70221	2.7489424	10.746727	78.176506	29.779638	60.144268	13.555285	47.361507
DD68	218.89355	0.428173	13.207109	147.35895	18.202808	105.94477	9.674651	38.839954
DD25	177.63992	4.0707483	12.391156	57.975037	26.367115	50.60499	15.027841	51.424427
DD28	185.65965	2.384825	14.761408	31.959473	10.262932	87.865456	28.420494	36.973267
DD29	199.17738	4.4515443	14.130857	45.515987	5.104622	1.0425174	10.872712	51.208927
DD27	183.8967	2.9670122	16.250177	427.41907	55.471447	47.355267	8.437583	45.75101
DD30	168.84814	4.5642567	9.336858	46.957253	15.55398	36.28244	17.237421	44.59028

On-Line Supplementary Table 1

Animal	1392037_at	1392064_at	1392074_at	1392171_at	1392233_at	1392304_at	1392382_at	1392392_at
DD81	24.07774	14.289741	46.083424	76.41283	19.091997	36.47775	150.0988	146.24147
DD84	21.194967	13.169362	50.986893	55.908463	27.164778	26.884954	145.13539	124.71595
DD85	15.637417	1.7464106	29.499645	24.599028	0.4911122	26.12682	144.81645	108.18698
DD86	30.834116	2.199204	61.770657	27.401217	2.2849305	26.297407	49.322197	185.9506
DD83	28.300505	16.114902	32.82767	25.626713	14.001148	23.463514	135.08351	101.36531
DD41	20.425508	24.395224	40.62483	24.803127	6.899833	33.891083	65.963295	107.25607
DD42	23.44183	29.719492	44.967484	24.044594	11.95799	26.750645	43.649033	100.15746
DD43	38.30553	13.86622	31.20656	168.87122	169.08347	16.885622	52.141144	183.69106
DD44	35.409103	15.803988	39.540432	112.01769	121.82714	22.875013	44.048454	151.55243
DD87	24.100016	22.448626	48.138737	29.341156	6.217786	23.927467	52.514496	106.30917
DD2	26.971048	1.6291945	60.129726	101.8789	103.70081	27.58649	62.224	161.05614
DD3	21.924696	13.380837	59.377007	39.752	15.519626	23.637312	44.63698	203.12225
DD89	15.590181	19.720592	75.13272	35.722946	13.452008	26.115784	79.35427	119.71969
DD90	11.770097	1.7736794	68.24374	33.607708	11.088899	34.70606	65.98528	130.23036
DD4	26.399624	9.211089	62.306335	48.661026	5.623513	30.604952	67.09781	125.07764
DD47	10.259686	7.789893	48.006016	30.06874	0.691899	31.745764	29.76139	127.56513
DD48	26.588295	15.655429	74.6363	22.160524	5.06181	43.960953	53.64211	99.80154
DD49	24.789717	9.312259	54.322666	36.389645	6.7780557	47.951183	43.657646	192.43196
DD50	22.24029	2.8288028	81.22094	46.689217	5.7264786	41.161926	45.16834	211.1267
DD46	7.514866	8.737097	60.415764	25.774647	0.8410508	30.240307	66.83375	141.39299
DD6	24.536509	14.031454	79.98828	77.10353	43.29107	46.855858	61.782394	165.18251
DD7	20.595255	10.647531	82.2827	53.832882	36.48116	35.5406	55.801506	155.33089
DD8	17.77081	16.978289	56.74095	28.70995	0.7080975	42.657738	42.189064	151.8372
DD5	27.69681	20.69428	45.685013	42.42078	0.4607171	39.652256	32.537838	139.04625
DD9	17.207912	10.817723	44.776443	44.965992	11.196288	33.05879	40.892593	150.48824
DD103	67.03677	17.299635	108.27126	80.26107	30.947449	1.0199223	47.106583	71.96395
DD101	25.064083	15.577541	145.26253	56.248604	2.7297761	1.3971733	40.624367	55.27596
DD102	66.54602	13.005927	83.0604	145.5275	70.878105	9.982012	26.376211	93.0573
DD105	41.273758	10.517346	109.29665	34.85493	4.0905495	3.7812526	51.595657	89.62756
DD106	28.888575	13.733754	153.02872	68.66836	13.898759	7.1031027	59.457836	68.67001
DD62	21.9911	10.218943	105.2514	37.51804	13.690398	9.188175	30.561111	59.498047
DD64	64.333046	6.418077	84.32826	63.615948	59.913403	2.4901373	25.023935	78.36178
DD107	117.92357	12.88596	58.639675	134.85663	212.03024	5.3310747	20.812218	111.17831
DD108	67.29314	14.197382	83.65633	65.94041	72.96255	1.4114673	25.978275	85.41846
DD63	64.98161	14.885742	96.15209	88.853935	86.15542	1.5172877	17.639181	86.34822
DD23	52.884567	15.523919	208.48561	65.62509	32.960342	2.3520997	21.622618	150.2492
DD24	15.332666	15.255124	196.50569	45.089336	13.546423	2.0982966	24.868938	85.37688
DD110	44.312386	3.8545964	273.6896	33.74518	5.6734324	7.34523	30.939148	87.76501
DD22	13.255809	11.312804	154.20746	46.589397	0.6984148	3.7995014	25.428047	69.98718
DD109	18.698235	17.152473	187.49817	32.89614	9.616981	6.6104836	26.854525	10.250692
DD65	73.2792	16.150385	135.2461	45.28761	9.003582	14.652808	19.66808	125.5329
DD66	72.72549	26.506056	109.70532	74.85558	8.395591	1.0259043	22.790922	7.0406294
DD67	33.862812	19.441368	48.480125	66.85856	5.605801	4.387476	14.425843	4.336233
DD69	51.816425	9.160432	208.0759	54.84462	28.720598	5.351326	22.074883	11.503762
DD68	72.05413	16.9389	124.97389	72.68502	35.201622	10.698219	19.903736	78.5618
DD25	50.69877	18.578367	183.26125	49.690845	0.934796	3.7105896	24.245203	194.37408
DD28	92.393295	16.651363	164.6659	72.94288	1.1634055	8.136871	15.872469	67.54068
DD29	86.06988	14.670146	179.37791	32.10467	1.2712357	9.758368	19.24567	7.559364
DD27	91.20901	10.677718	108.19537	139.14595	128.75531	10.083419	8.54814	78.36438
DD30	52.14686	11.292884	215.67935	54.414253	4.9763093	12.184855	14.013083	176.37299

On-Line Supplementary Table 1

Animal	1392504_at	1392539_at	1392598_at	1392678_a_	1392731_at	1392767_at	1392776_at	1392788_at
DD81	254.63646	93.68078	668.3939	33.481735	41.07141	148.06107	8.248733	27.777584
DD84	254.37927	165.6749	635.9076	47.40871	26.494453	101.79177	7.7098	24.142517
DD85	249.37024	99.13447	629.9718	25.290766	34.637554	120.39764	14.356048	21.011908
DD86	192.00044	197.06749	675.6994	17.014685	52.65828	120.5817	11.710424	14.746203
DD83	265.67783	123.75898	681.6783	32.70299	40.44026	143.91576	13.410041	20.669083
DD41	242.24915	175.60379	748.7055	3.8991795	46.419735	78.19287	12.972421	23.491348
DD42	209.57677	208.40677	770.9472	3.5585022	47.671085	96.944016	11.216303	23.517565
DD43	201.71199	222.51097	720.5175	25.209	86.78592	161.54901	13.58975	28.909029
DD44	217.87225	181.73462	734.22217	29.103956	63.539948	139.1818	2.9237232	27.586966
DD87	229.93915	202.2407	674.87476	6.8378925	36.452	107.47057	21.05355	16.083256
DD2	219.64433	101.37057	637.9339	18.226213	50.853874	141.4019	3.8434567	17.311535
DD3	209.30281	224.66866	608.35065	10.437479	62.688385	117.7615	13.257693	22.724491
DD89	210.45308	84.660095	1003.9241	3.8713536	40.782997	316.46298	8.818059	31.215845
DD90	227.1111	81.228615	1377.7467	9.894213	26.844204	581.35065	11.011223	44.70222
DD4	207.03204	72.49787	1045.6171	1.0695748	47.868828	301.99808	12.179236	34.07864
DD47	153.54073	159.12065	544.9106	2.8184924	74.21515	101.00697	8.6107235	24.652567
DD48	214.2821	184.88838	610.26215	16.382631	61.389713	122.75655	11.622673	12.745049
DD49	157.15369	181.05061	613.3554	12.705688	79.4333	127.25748	13.221586	25.68622
DD50	144.84001	172.48701	589.0421	2.2109442	96.97657	125.29887	9.319507	15.596066
DD46	200.92316	121.09789	652.60785	3.1621106	61.697308	116.50234	11.302923	16.801657
DD6	238.41438	72.531166	754.9348	3.0420897	64.93885	155.89908	7.538545	39.21612
DD7	210.97702	64.95102	948.04926	1.870422	56.33328	264.9836	7.7062855	31.276169
DD8	210.14513	73.712364	768.388	1.8283355	58.120914	201.11476	8.178134	33.657413
DD5	189.24667	64.0658	733.25366	4.528401	58.51371	195.6086	3.0461426	49.686348
DD9	194.39423	64.97682	747.1697	1.4244349	61.82551	188.87689	9.48215	25.154673
DD103	89.75539	429.1967	409.7076	75.47272	36.148872	13.651808	2.1062696	128.08775
DD101	101.79508	314.81787	366.49072	67.67153	12.923409	7.9200535	2.4554913	129.71269
DD102	70.44932	419.4164	370.75125	80.57433	59.302757	19.451624	10.543622	136.19553
DD105	99.7944	388.31238	323.13956	100.3597	15.575861	21.496002	9.500665	119.88792
DD106	109.55061	476.49686	302.18832	51.055878	2.8204703	90.57231	11.57398	126.41713
DD62	153.00974	209.9144	349.66113	40.79664	3.2721374	21.248194	21.59599	121.55396
DD64	115.27687	221.43184	320.60614	44.261402	35.94007	23.565922	15.899321	131.72456
DD107	96.927246	261.11493	340.8029	33.20706	35.95057	110.41267	18.204224	133.1497
DD108	95.65787	473.402	395.90155	53.478016	49.409885	41.82378	25.299822	90.933395
DD63	117.25085	461.80243	386.4075	50.21103	43.132023	37.145756	40.416664	93.856186
DD23	103.09728	494.98355	300.99203	29.097715	48.290085	41.36699	20.262672	105.94733
DD24	119.1791	320.74014	380.18796	44.730022	29.194029	34.736973	28.384983	119.41718
DD110	114.11735	350.36185	423.7194	22.313692	27.117945	69.42328	46.823112	130.04228
DD22	112.89111	309.9611	322.67737	57.245293	28.165468	52.627598	29.445871	97.658165
DD109	112.94788	479.0504	217.24611	17.91828	26.765461	16.094181	23.673025	117.61567
DD65	76.15589	270.10385	534.0637	18.993412	35.466293	31.01693	68.65355	152.73334
DD66	103.12199	317.32584	597.8938	29.150053	26.320026	91.1032	54.452854	140.14638
DD67	114.89423	439.0816	384.7288	4.3116455	42.570236	19.640898	4.6292763	143.81017
DD69	86.9529	273.30865	416.76352	17.850422	41.18859	32.27245	37.893116	176.52751
DD68	106.43562	314.1514	425.3581	25.939552	37.746284	24.77113	55.73816	156.30861
DD25	71.582016	379.86954	240.09785	18.927746	46.775906	7.182301	42.71717	109.63612
DD28	88.957985	205.05174	415.124	18.775513	46.38697	21.962255	17.958801	126.18809
DD29	104.47688	254.95601	442.75577	24.966997	57.77763	30.699408	28.567757	108.84567
DD27	99.50053	198.55333	646.99084	36.723305	42.221447	11.631193	50.021793	130.50067
DD30	86.0118	431.04803	236.57375	26.34398	57.700016	2.6206062	30.369125	126.29643

On-Line Supplementary Table 1

Animal	1392794_at	1392860_at	1392954_at	1392969_at	1392999_at	1393018_at	1393108_at	1393139_at
DD81	23.500519	4.227903	17.213713	52.18178	8.693839	8.969577	18.387726	198.44067
DD84	19.557598	10.145608	15.579674	19.235788	13.152424	17.744051	13.939793	5.360323
DD85	1.9426124	8.307154	19.934956	41.44574	13.353668	19.037455	12.942666	15.873702
DD86	15.282852	6.588321	21.631283	1.341484	8.215132	17.94755	7.2935414	8.965426
DD83	18.246195	8.864556	13.98509	9.974744	16.601788	11.473846	12.517955	2.238398
DD41	24.061226	11.385891	7.019827	1.6316289	20.371136	18.487001	12.848338	13.695179
DD42	20.78289	14.865023	21.85172	9.363317	21.543674	10.434818	12.833067	15.289088
DD43	51.673298	17.369432	54.485134	13.424887	25.140305	19.723314	26.27384	0.6954138
DD44	67.245804	11.909503	46.55784	11.223825	33.82255	9.929101	21.737848	1.1356798
DD87	31.060501	9.350936	27.983519	14.071569	21.344038	17.090912	10.978757	1.8559395
DD2	44.65787	9.942919	35.456642	11.567087	44.04911	28.65871	9.4466095	7.919985
DD3	49.982597	5.57232	33.299747	8.979112	52.560432	51.15173	10.947239	8.965776
DD89	63.051147	3.9732583	23.042046	13.742039	47.8553	39.842793	8.309045	0.6679502
DD90	61.316174	6.4196486	29.904545	18.411123	70.87001	86.7808	12.031776	3.8323593
DD4	67.1955	7.1906433	40.415714	46.83836	88.84193	91.373856	23.521118	1.5038031
DD47	46.046352	5.558884	33.08234	62.453682	27.049774	43.2261	19.683723	56.446682
DD48	35.00785	1.5568023	33.488304	40.793995	41.933247	44.915554	13.781536	160.19693
DD49	27.751604	2.872252	40.81173	20.478117	90.58684	52.83568	8.108671	22.81049
DD50	22.942522	6.1049175	37.118755	20.454226	56.614098	50.84177	38.426323	39.263393
DD46	37.789307	6.9075	15.629184	24.675735	61.75796	46.12637	15.389644	40.033478
DD6	45.37653	4.7898865	25.287094	15.390237	46.4459	45.460575	19.390942	1.8569405
DD7	56.79393	4.679401	35.32661	14.803221	71.72748	71.63543	11.200156	1.3168103
DD8	58.558544	1.3091385	22.434162	8.892782	72.5488	60.994694	12.539531	6.3134217
DD5	55.045685	16.208105	33.91226	15.783156	63.824863	71.38754	18.399807	0.8202604
DD9	73.78264	6.4383383	34.537678	17.179676	94.390236	51.905983	11.89799	1.1088419
DD103	10.424414	82.442245	148.2484	7.3982186	6.9935465	2.8105934	206.35043	18.319933
DD101	9.622718	76.700195	27.57257	12.154986	10.148685	9.259502	160.7276	21.744198
DD102	14.842227	98.34422	318.41516	33.081615	17.743214	2.5035753	19.682398	115.728
DD105	8.648248	79.26605	129.90129	31.334303	12.222376	11.497988	100.96883	33.948692
DD106	2.8264134	69.63462	29.178345	37.937775	17.30605	12.857166	196.08548	100.67699
DD62	22.921703	15.146711	140.73169	11.240003	23.626545	22.158875	162.7644	1.0973413
DD64	20.342773	46.650394	44.595825	8.763202	31.052689	14.298645	619.8547	0.712059
DD107	20.612528	141.61028	362.70114	3.3510702	60.258396	17.71614	399.4574	1.3441261
DD108	19.487473	88.94789	222.50845	13.242169	20.668018	11.951603	345.16605	1.4767655
DD63	15.68475	72.429924	224.32549	3.140841	20.412624	15.937761	356.116	1.0460936
DD23	29.513512	84.67487	244.3766	3.2738426	21.520475	17.619312	401.24173	7.2492657
DD24	19.685024	10.688467	236.21988	12.095635	17.536049	21.094997	335.28702	0.3389632
DD110	12.973697	64.89305	228.07986	0.9136754	34.430767	18.296795	377.51166	0.8422838
DD22	24.960407	45.03397	193.95593	6.8754873	10.000542	17.848537	321.80872	1.9150116
DD109	28.032171	41.41304	216.97443	9.57068	20.307394	18.293863	401.84827	0.6077722
DD65	16.083218	3.7615738	58.251133	12.355525	34.81416	26.319057	711.8353	0.4966263
DD66	33.955585	71.503555	45.94703	0.4386188	29.933556	5.8721104	788.4233	3.5468974
DD67	15.600875	49.558537	142.57506	128.33458	4.922331	13.185894	254.6742	269.09967
DD69	24.534664	50.66915	283.69623	14.984981	14.030863	17.601994	400.59805	0.9786498
DD68	26.671066	13.082278	239.35278	16.791267	13.666497	53.132465	371.07745	17.353321
DD25	29.250917	81.669525	257.7465	7.4922833	27.360344	61.215996	415.84198	2.560428
DD28	34.02141	39.83314	35.03775	3.779816	41.773567	30.445642	463.01407	1.5221152
DD29	26.104101	34.123734	223.7659	2.2922204	16.973698	14.896527	271.2817	1.2774827
DD27	9.17381	95.87008	39.856953	7.627627	39.40523	46.566437	639.51697	1.510596
DD30	21.321014	79.63344	253.69504	9.923052	16.693665	49.66673	399.97687	4.430686

On-Line Supplementary Table 1

Animal	1393187_at	1393259_at	1393297_at	1393338_at	1393421_at	1393440_at	1393469_at	1393476_at
DD81	154.25829	64.69483	65.6722	351.11182	128.82802	4.8712997	34.740116	73.728134
DD84	112.7757	81.04323	60.234165	301.6415	160.51534	10.525452	38.272118	69.33347
DD85	18.001118	91.36079	6.801443	275.46466	147.40431	8.117008	44.60919	62.465942
DD86	33.76241	45.657753	1.9955007	34.61312	175.44426	10.716386	58.51478	34.65782
DD83	39.061176	77.34067	29.706131	271.7748	153.88278	13.3209	32.71207	69.29993
DD41	41.978817	36.363174	0.8342134	73.66581	122.77355	7.0232606	120.32231	41.57594
DD42	32.478653	20.89358	8.302631	128.12515	144.65875	11.923512	73.99776	43.324284
DD43	1267.6023	44.390446	579.3597	22.121635	132.69746	10.466199	57.652157	69.0248
DD44	973.7194	23.875868	369.32877	31.09845	121.50432	8.66535	71.04515	43.06191
DD87	44.82199	34.487804	16.038012	115.76701	172.31972	11.526921	54.122284	60.742218
DD2	659.43384	3.5477152	332.16907	103.73629	89.8144	7.4267044	82.827225	39.500854
DD3	59.655056	75.306854	18.507153	38.239048	113.04393	5.3905616	99.95235	57.860104
DD89	52.12941	45.98966	19.89371	44.671894	121.23498	1.1152729	161.98494	37.199875
DD90	66.582886	68.35941	15.699932	12.526423	99.80614	0.1793667	167.45953	34.900047
DD4	95.3441	62.34459	13.181631	26.852205	134.1226	5.4665365	87.63822	37.21114
DD47	38.978474	48.982872	4.4955106	21.734446	131.76492	3.6809735	62.14545	32.36508
DD48	33.73376	115.81168	0.606852	173.967	147.44205	2.6651063	60.64414	65.68896
DD49	42.939537	71.84425	13.678949	29.280851	142.1547	0.6828396	145.77263	58.424866
DD50	69.76483	92.63174	11.200122	10.09185	141.07474	0.2208931	115.82512	75.89615
DD46	50.72315	49.713146	5.962671	32.88486	138.47192	4.3831234	162.76923	40.608765
DD6	331.8858	58.5512	177.98074	73.70203	115.25056	5.0494466	152.9885	42.797287
DD7	166.50313	57.559322	78.20758	23.160555	135.17345	4.0725307	167.96112	36.17099
DD8	48.055897	48.25518	7.8831024	3.5229735	121.38637	0.705826	184.71452	29.00252
DD5	68.96815	49.286148	16.443233	12.957033	124.63942	10.941065	186.30788	34.559483
DD9	115.4127	65.95561	27.56835	11.949905	150.23215	3.8071783	182.26065	40.201584
DD103	177.58894	128.29782	68.932014	76.455	53.32661	4.2359896	40.193638	9.902086
DD101	80.21185	165.22212	35.12564	263.25357	62.86773	0.2543229	19.22503	11.467349
DD102	419.2257	117.22054	171.1583	93.74212	46.436287	5.180026	35.86359	22.307142
DD105	44.2037	169.63922	10.363442	247.1092	64.54673	5.2670064	42.11833	23.947214
DD106	128.09828	184.7862	36.47494	267.9984	40.657337	4.897077	29.551317	16.533655
DD62	110.89666	116.84347	33.242966	116.32835	38.52977	3.0580776	80.59514	13.510047
DD64	274.85745	90.367256	106.598	29.314095	11.083605	5.6821103	132.45155	16.442682
DD107	1053.6543	81.51553	1014.8901	12.688438	24.765177	7.221637	458.1095	19.50048
DD108	465.8735	125.36642	322.54657	33.292004	61.993332	0.8322187	196.50066	15.640474
DD63	455.30872	114.49005	314.40277	45.888172	51.285427	4.6841073	193.29318	8.85861
DD23	262.28146	109.15714	90.793045	17.142141	37.416225	5.02616	196.15982	6.0411897
DD24	82.913704	109.21912	12.369421	18.947363	62.146416	4.8309045	396.51572	7.4386425
DD110	77.967186	112.08512	12.741122	26.017021	17.816406	1.8864903	650.2359	3.257686
DD22	32.76467	98.76043	4.7246647	19.704182	53.44477	5.8339324	290.91437	3.0067635
DD109	63.250004	139.41219	4.786385	12.266515	47.72588	3.1664221	354.55365	8.4630785
DD65	106.24833	163.53102	23.906044	4.4909525	41.907375	0.2608171	435.61603	1.8853941
DD66	65.818535	146.12592	14.433538	50.22979	53.908184	6.8025656	331.62903	8.291767
DD67	29.989618	132.1168	9.856224	17.419338	55.626595	2.2959542	230.24469	11.001645
DD69	157.92139	180.5755	52.959213	6.0956426	50.639683	3.587507	317.0923	9.327842
DD68	209.35355	180.50513	120.54368	7.0118537	27.770933	5.3386745	554.99896	8.056635
DD25	87.40998	128.36142	24.835087	15.942461	38.862087	8.427165	280.3718	25.047504
DD28	18.583635	157.45575	11.69027	2.8284445	34.317924	2.4755723	281.70508	12.256373
DD29	44.29966	127.5696	13.974002	54.32155	31.604544	4.766349	349.45834	15.463079
DD27	662.874	135.5197	562.77496	40.63304	32.671486	8.77564	201.73254	7.248549
DD30	98.294846	137.00131	32.243923	14.982433	33.936077	7.5773134	290.49045	12.755088

On-Line Supplementary Table 1

Animal	1393627_at	1393657_at	1393689_at	1393751_at	1393783_at	1393791_at	1393809_at	1393821_at
DD81	21.985703	25.424433	53.174164	13.174177	1.2275274	2.1835077	39.655502	26.986609
DD84	14.396528	57.01156	68.87207	3.3563788	6.6134486	0.45359796	54.885693	14.616665
DD85	31.305462	13.992202	68.415855	16.480175	0.31150585	0.61382115	24.951601	12.622514
DD86	12.71932	62.50004	48.049335	8.879385	3.5079794	0.19054037	37.226524	13.579329
DD83	13.164439	58.08964	68.61914	12.76961	7.9211226	0.62427807	37.408127	21.051912
DD41	864.40314	52.613235	71.365486	21.428625	6.723668	1.0583423	12.769724	91.03533
DD42	1573.2076	42.20243	68.35291	18.530495	0.23910116	0.6508048	44.10738	142.25569
DD43	39.432537	74.813576	56.13158	17.829786	1.4857919	25.517702	62.868977	22.421873
DD44	17.154257	81.64621	71.04114	8.237326	7.984741	23.993795	46.810688	19.934628
DD87	998.6121	55.597908	51.229683	13.088806	7.875544	0.5225379	46.632187	93.00032
DD2	234.98802	42.78208	44.815556	14.990066	0.1875375	9.7398205	35.096794	35.496696
DD3	17.865866	71.51885	43.04809	25.64498	12.502524	0.893998	48.899033	6.0475855
DD89	4.4057164	8.342756	43.81766	22.687864	7.4571767	2.4550602	65.0801	12.98383
DD90	7.666987	16.009893	40.719887	30.010809	10.582395	2.4945467	51.347244	9.930537
DD4	10.679448	13.079708	35.564983	24.516518	12.143406	3.108506	8.835679	27.723845
DD47	848.93646	74.10225	33.6954	15.796664	9.6953535	0.6790534	52.192276	65.08375
DD48	442.26398	64.175026	53.756462	24.636883	3.0593455	4.4556036	68.68659	59.55308
DD49	18.719288	55.24551	34.363224	23.641497	9.178161	5.1224833	65.81061	12.727612
DD50	2.9237785	61.37762	41.393627	32.250355	13.091866	0.9436721	57.033424	5.0270796
DD46	4.5459337	44.89321	48.71161	18.176992	5.684416	3.7407448	38.71498	15.187686
DD6	3.2761118	37.052822	51.752266	22.491297	0.8096928	12.687392	62.254074	18.972868
DD7	13.684056	15.324721	50.07195	24.653036	2.2116535	7.628396	55.11087	12.132839
DD8	6.784232	37.89219	59.2715	16.862759	8.731184	2.8800793	50.556396	9.069532
DD5	6.63575	19.674416	72.69554	14.478155	9.963643	12.857739	47.594563	24.671476
DD9	2.4507966	22.268188	54.210255	25.826487	13.46018	6.1003575	51.141594	16.523943
DD103	16.578632	97.740074	124.16154	12.391359	4.9873753	5.862152	118.33072	1.8158249
DD101	19.633234	77.32448	121.64233	1.2790619	4.0653615	5.2648015	79.90823	13.513215
DD102	18.680418	101.67597	90.28638	1.124091	7.876439	16.805567	107.43118	6.108522
DD105	11.395697	64.19307	130.30077	9.019626	7.1229033	3.70787	96.72688	8.759044
DD106	16.326422	89.23133	127.54236	9.866758	10.837816	8.71506	104.12495	2.754577
DD62	8.160513	63.45742	121.63136	11.584005	0.6977411	3.1118174	82.16033	16.57991
DD64	3.9796038	70.18627	120.33856	6.3650937	8.889118	8.569018	75.671776	21.413881
DD107	16.958681	47.76328	60.10152	5.4105396	5.6908646	31.921675	72.89279	10.709824
DD108	11.697441	63.151497	81.55616	8.189352	8.162065	14.039565	115.26147	18.650112
DD63	11.645289	87.059715	71.28222	15.737347	4.5782113	11.970676	102.294426	18.509727
DD23	15.27989	110.00399	114.28185	4.291631	1.6237671	8.6452055	142.61954	15.813347
DD24	4.0150404	99.12418	117.46893	8.857183	0.72735494	7.9267273	170.74393	12.335155
DD110	8.757016	75.6756	87.105995	2.94213	4.4622097	5.287885	131.9769	13.1607
DD22	3.1524055	76.07219	87.07443	4.591653	0.7208034	8.049032	136.31818	3.7724273
DD109	8.437035	78.30445	88.318344	13.840146	6.3409715	4.3965597	143.75565	17.83116
DD65	1.3451519	47.180813	91.50005	4.672152	3.1499152	9.520222	84.76102	3.4222791
DD66	699.8584	55.457317	60.552258	5.9179034	2.2243595	2.8315172	72.69069	89.60007
DD67	14.163077	52.770275	111.64531	8.2698965	5.0861664	4.173488	59.516705	8.163639
DD69	6.0582657	65.37237	60.524075	16.245028	1.1134251	6.453751	83.13637	5.547777
DD68	10.028191	75.81513	105.83306	5.8698573	1.6444892	3.1306968	83.68896	9.449828
DD25	822.2623	38.927135	100.76857	8.846188	0.66557086	9.032979	132.89476	70.04408
DD28	8.370841	76.81969	67.82561	12.338456	2.758247	6.0727596	78.50616	20.5602
DD29	646.0213	64.42077	59.984097	9.716888	4.229771	0.2801045	90.17188	48.897533
DD27	710.32086	68.50961	92.22584	7.408522	2.372292	17.203268	92.696365	87.26671
DD30	840.2276	63.578354	87.00496	16.949938	1.6426747	7.1269884	143.45667	58.140488

On-Line Supplementary Table 1

Animal	1393842_at	1393910_at	1393926_at	1393929_at	1393945_at	1394020_at	1394097_at	1394401_at
DD81	120.61871	53.310722	59.774597	51.55293	2.343112	169.89703	9.226711	1093.9833
DD84	127.02508	25.173811	48.043938	42.76788	0.2255352	191.78406	10.381697	1417.4429
DD85	112.63537	29.088255	8.172857	3.6421986	1.0278343	217.04085	2.313446	989.17725
DD86	125.1336	28.703999	4.1828237	2.375569	5.2108026	293.70776	23.330082	2670.6697
DD83	142.38866	34.452732	18.173609	6.5679574	5.196304	190.25697	2.3973918	1041.1049
DD41	125.05028	80.47398	21.69778	4.0728564	4.3523107	199.92285	18.13734	1505.5576
DD42	90.88131	63.056854	20.18353	4.33432	0.201847	193.60243	17.299076	2122.994
DD43	106.85032	53.00478	269.01913	179.11407	0.4393719	155.2702	16.51243	1476.1675
DD44	104.86344	50.165607	205.84386	91.02834	0.5858058	176.41664	19.897194	1511.7231
DD87	123.337166	70.37199	20.634964	2.7999008	0.4221086	186.27824	19.963976	1768.9324
DD2	108.08774	67.17122	166.55289	102.8941	1.0640285	138.96938	32.682705	50.175724
DD3	92.984604	100.88275	39.519283	3.7504756	1.8142052	159.66418	44.75454	141.50111
DD89	112.620316	288.48523	27.159143	9.839694	0.1477379	176.32524	67.1864	350.2293
DD90	116.19408	208.81706	27.407076	12.136567	6.443992	197.35526	43.443253	386.88358
DD4	96.86949	253.51419	23.351286	2.5949132	4.367812	170.78162	55.11395	244.95107
DD47	74.05357	259.10907	18.790592	2.4693966	0.4123084	187.73419	67.76429	128.23703
DD48	94.16318	244.2227	12.830542	3.0633006	7.8116155	122.9109	56.366875	69.79498
DD49	103.30012	330.94913	16.328709	2.2296565	0.1802319	185.63866	86.14269	65.142204
DD50	91.84203	262.30164	30.153858	4.1054153	1.0911217	173.9518	103.14072	105.76129
DD46	117.48125	111.67901	19.135838	3.4715447	0.095753	201.40439	110.08399	80.63396
DD6	120.6178	311.2901	92.13645	71.22436	3.3753119	207.11008	82.008484	53.042934
DD7	126.135895	214.80954	59.800957	33.49835	0.2281148	253.5939	72.407616	76.58874
DD8	140.61786	396.05457	23.954472	2.0153008	0.4892167	339.80365	88.86546	106.38882
DD5	98.41497	279.32965	12.750488	5.493433	1.0175102	259.80563	113.2956	81.88206
DD9	115.37994	235.45848	37.209686	16.71606	2.6935422	264.78116	112.15694	82.145065
DD103	25.176481	103.32107	45.000206	51.88859	0.716557	68.85386	3.0193503	3671.2278
DD101	112.36473	107.09308	27.399933	37.828445	0.2190057	40.389164	12.208443	1806.9049
DD102	29.776388	109.17693	118.88712	72.593414	0.2638608	96.985954	13.26808	2137.1199
DD105	28.23755	128.85048	18.060259	20.745295	0.4921423	63.59484	22.000164	2281.1973
DD106	128.9457	141.8203	28.77717	45.493908	0.0565493	53.401264	3.3169682	1645.3643
DD62	87.97661	150.27711	29.130209	20.367384	0.8534632	65.94075	25.803108	740.46533
DD64	87.70637	123.52348	78.915764	69.673256	13.092492	95.55394	94.5521	696.65955
DD107	32.482746	106.63425	286.14984	234.59692	0.9032736	93.29973	42.884758	386.12643
DD108	32.584377	203.65671	108.58569	112.59138	9.634585	80.14048	39.303417	1215.2177
DD63	28.862392	204.05669	102.1476	86.45969	11.037898	73.562965	42.09593	1247.0332
DD23	86.29216	324.20148	61.456146	52.40958	2.7058284	85.50437	81.01237	1728.6034
DD24	123.10538	371.1256	27.638012	13.407369	0.7391107	116.89159	67.97111	1399.2719
DD110	119.88464	441.78693	18.688452	2.7740347	7.627281	80.99929	115.21742	613.903
DD22	98.34443	398.52026	18.836727	1.3118534	2.9254391	111.65058	119.88787	814.8058
DD109	74.71403	539.4442	21.828848	3.2221308	1.013122	126.75099	112.97364	897.70154
DD65	42.244995	116.40065	34.29978	39.58485	6.09467	102.52025	109.52433	700.00165
DD66	57.477676	76.19748	36.552326	3.5404296	3.855269	93.48546	127.49489	526.9569
DD67	53.36561	40.72461	27.516977	1.8057917	0.5045489	149.0718	350.1113	1386.6606
DD69	42.095497	67.47773	36.77418	39.15536	14.717362	109.1384	217.45691	427.89752
DD68	8.176594	92.44867	61.530598	43.63512	5.098835	93.539116	220.15298	389.88907
DD25	75.725624	461.49753	23.384388	7.324024	6.9744954	133.03989	173.30948	284.76422
DD28	45.55422	153.47281	22.694687	5.4625607	18.119598	102.77569	160.56009	180.19179
DD29	63.548485	103.16399	35.27557	2.6961267	21.798346	86.8795	260.42	614.63544
DD27	73.8077	104.59551	173.0881	198.83348	0.342442	49.61855	169.53287	241.49484
DD30	65.55705	519.897	26.801924	4.430329	5.615737	100.86978	165.82674	320.4815

On-Line Supplementary Table 1

Animal	1394462_at	1394490_at	1394501_at	1394554_at	1394671_at	1394732_at	1394756_at	1394803_at
DD81	810.4844	207.86394	41.73744	31.115402	87.26778	21.303692	49.297287	83.95325
DD84	713.8101	182.69905	33.611088	16.731842	85.85316	15.635237	47.3309	96.77715
DD85	747.8671	196.25821	22.781168	33.0064	113.97337	14.183682	37.44727	94.89711
DD86	13.153211	214.3508	23.463865	3.5741832	97.68239	23.192999	43.10978	104.02506
DD83	605.32025	141.19875	25.037863	34.15189	90.02955	23.610779	22.699097	81.83369
DD41	139.56447	377.66797	35.827923	31.992079	129.53508	20.686188	33.43763	97.32755
DD42	40.27371	411.06442	39.56205	42.175854	148.64024	11.736976	35.535023	109.59825
DD43	45.87276	305.58795	125.65595	39.60905	123.69366	38.00172	69.20765	116.78301
DD44	49.793583	290.1046	122.76962	31.371218	118.42616	17.74602	36.277115	102.42268
DD87	68.08381	270.56482	35.520447	28.481878	126.61937	15.776499	26.127789	98.40929
DD2	450.07083	203.9793	155.46156	19.78503	97.50599	28.81983	17.615814	118.63584
DD3	102.90139	150.69812	163.96136	29.152346	99.291016	14.404012	25.495432	105.39483
DD89	95.57995	231.18336	155.2187	34.346264	223.76097	21.227417	65.17098	102.12125
DD90	9.981752	250.24518	153.0663	41.800316	231.6564	36.538097	64.651344	98.0211
DD4	20.872011	214.3706	193.03378	31.29178	231.67046	25.646885	52.263752	97.77711
DD47	18.426605	281.94342	102.72616	31.937115	145.74655	12.423358	39.49201	127.2562
DD48	835.357	171.19847	93.417984	25.089188	108.18814	21.42848	56.588318	93.34311
DD49	61.00996	218.77042	143.22615	44.496033	116.2108	18.005898	49.979187	109.38438
DD50	76.102425	171.48604	178.9148	46.51712	107.75269	11.212116	57.619957	116.25617
DD46	108.10623	205.66089	174.53229	28.48825	168.50717	21.612823	28.1721	105.12598
DD6	303.97592	222.09917	155.13864	36.054317	171.18547	25.738905	62.594425	94.55037
DD7	44.02362	254.29608	159.65129	32.020332	167.8599	22.213757	53.428116	106.93456
DD8	1.3987381	247.99596	141.99132	47.678833	165.86732	20.539978	39.692635	82.32236
DD5	29.224016	219.57344	164.51877	44.691174	131.20985	8.968584	31.296665	94.060356
DD9	1.7638448	211.46524	165.96614	35.544933	120.27259	13.428303	41.063854	100.09164
DD103	117.15217	51.339016	22.373426	67.26852	42.75697	53.088654	159.18626	62.875813
DD101	436.5314	37.2754	17.750154	62.968193	56.082233	33.83184	125.31163	24.702797
DD102	163.08351	81.341515	59.81259	63.94098	36.447014	48.85734	200.66348	91.39855
DD105	542.4435	55.682446	18.25471	74.93332	39.367214	56.294018	128.57446	46.784264
DD106	401.30023	46.936245	32.998573	55.279823	95.03707	37.74475	174.09856	27.829416
DD62	324.92575	40.63644	43.754997	62.610703	67.58451	47.951313	129.45888	62.30656
DD64	100.20474	64.01514	46.395737	81.17988	43.01313	35.474854	84.90028	24.263422
DD107	32.612816	55.264626	133.29192	84.379974	71.462265	71.12544	139.1997	77.07535
DD108	70.3924	58.26405	68.5914	59.698475	64.89458	78.26432	174.21858	80.063255
DD63	50.59351	74.7097	64.61744	74.015175	55.303524	57.911625	143.19867	72.26999
DD23	9.326094	84.293274	46.46737	74.69259	62.688835	51.156242	138.67	83.3784
DD24	42.23112	139.1612	34.19175	80.288734	92.321396	50.4596	106.33031	69.14053
DD110	122.94624	98.55489	39.922623	63.596607	135.49107	54.50516	147.5587	61.054058
DD22	111.64224	85.5666	30.952868	64.09302	116.40025	56.173847	77.54161	56.34697
DD109	33.05422	109.60305	63.9463	45.290554	108.67962	37.355507	126.71967	76.08222
DD65	26.463062	72.918365	57.5012	48.195732	113.06465	34.034626	141.7911	21.738096
DD66	7.0555835	58.906048	56.619682	68.08236	110.23952	35.209347	70.021545	14.536092
DD67	47.553036	64.76926	32.914783	56.58786	74.709656	58.53435	91.246475	74.453636
DD69	9.57804	65.03795	65.5779	71.80885	107.10086	49.671856	106.92366	78.467415
DD68	19.56595	61.779987	64.038536	66.70811	66.18988	57.339123	129.95164	73.147285
DD25	2.4175746	120.81829	61.298542	81.845985	57.270134	41.71348	178.85887	78.853615
DD28	3.0546868	44.42653	64.998215	95.61676	65.213036	27.503887	126.94312	13.453701
DD29	5.7987013	61.892567	46.260147	60.009457	118.33525	38.206547	88.29765	76.495445
DD27	68.38478	76.15809	109.87906	70.52884	86.71346	64.11441	134.5826	23.787016
DD30	4.2281814	121.72147	65.90585	86.185875	50.394318	42.568382	159.6322	73.75506

On-Line Supplementary Table 1

Animal	1394805_at	1394839_at	1394844_s_	1394846_at	1395014_at	1395020_at	1395030_at	1395073_at
DD81	13.473451	90.08501	46.131306	5.433346	136.67607	12.879571	150.92549	20.804192
DD84	10.568164	59.59147	2.6727128	7.7700124	122.87011	10.184748	158.80243	9.858188
DD85	25.402025	50.35045	11.35418	1.259316	100.13643	4.778627	139.44966	13.054379
DD86	15.356402	44.564056	1.9893026	0.7467327	126.56484	11.763323	157.29044	14.877734
DD83	16.97963	54.262775	5.012741	6.7214565	122.42884	4.7439876	138.31694	12.836364
DD41	8.851136	36.436718	1.3229942	11.150294	69.66516	7.627965	51.829453	15.101123
DD42	15.124894	26.810665	5.7434855	12.282782	65.82753	5.8265305	49.80421	17.542694
DD43	26.925703	121.25237	1.2294251	11.724096	98.7188	9.675487	65.24967	13.442637
DD44	19.967978	71.739685	17.280996	9.5909605	75.44252	8.103936	44.94974	10.161924
DD87	14.480847	43.515194	24.759426	7.004407	78.38403	4.5683346	53.889412	22.741413
DD2	15.340877	64.83807	10.163799	7.914763	81.422806	4.9788127	112.93649	9.803551
DD3	13.276541	55.552273	7.3148923	2.5340962	136.10815	7.3376684	87.34137	13.767203
DD89	13.529589	21.067446	2.1291444	2.0990803	71.952736	3.2789435	41.860767	12.141476
DD90	13.437265	30.172419	7.4990764	10.306636	95.70834	4.90128	36.84724	19.533218
DD4	13.032935	32.313267	121.69852	6.1498737	88.011345	5.4686985	12.133505	12.745516
DD47	11.796903	63.048985	202.41309	12.284534	114.5969	11.980796	88.678474	16.069376
DD48	18.922764	62.19034	31.668407	5.613939	111.55338	4.271694	111.7104	22.113068
DD49	13.714102	66.91826	21.998398	1.6197495	114.52543	4.674269	75.42048	24.485596
DD50	1.8740625	48.433826	6.523615	3.8059688	128.52391	10.159506	136.95859	28.721985
DD46	19.782286	55.541847	20.54513	9.68679	76.97151	4.631427	44.868797	12.48627
DD6	17.91347	65.132675	2.4543023	10.564014	60.77861	3.2833545	53.416546	19.729565
DD7	16.618364	53.80442	4.44277	10.155532	86.02209	8.567308	46.444813	26.904228
DD8	16.645397	52.717873	0.8710178	1.0596452	64.25992	5.134801	42.058556	17.978565
DD5	10.904226	33.154762	3.366512	5.3228407	69.83486	13.278195	32.279957	6.3345485
DD9	7.0528216	36.753258	0.6182644	6.8463836	72.19666	7.100265	40.75643	10.698923
DD103	53.03902	13.194527	0.9039385	64.1268	261.89294	22.441599	27.467484	68.63977
DD101	18.998186	2.6798599	6.620554	8.346421	284.22034	27.551672	6.4240866	35.446354
DD102	40.313942	10.10444	36.63002	230.66182	251.19188	21.99237	36.945927	47.642433
DD105	20.110056	5.161275	12.452452	65.20777	288.6525	25.223433	32.818043	60.183323
DD106	30.579546	5.5564075	11.131182	77.95178	238.895	12.330737	34.74285	44.94978
DD62	31.97923	28.549683	1.7595736	62.036465	231.5997	18.940098	7.7911377	32.63633
DD64	24.508873	11.752496	0.6172558	2.9762814	231.8719	36.452652	28.314253	36.59123
DD107	37.56182	134.59088	3.75562	133.18439	227.42923	16.989527	9.039476	26.997675
DD108	41.658035	24.167631	0.8087076	279.55875	232.67421	26.932793	17.99755	50.37903
DD63	29.040308	23.531656	0.4951049	265.5235	234.51828	25.25533	6.8555775	54.458828
DD23	23.216282	5.903353	0.792902	199.39407	234.90718	10.077018	48.275898	37.833153
DD24	24.945177	24.351023	0.2930233	151.95566	259.7938	25.60514	4.6517577	48.67174
DD110	25.16205	7.5597305	3.4802296	244.36488	240.27632	18.2608	13.952484	66.24117
DD22	16.997787	12.422207	3.5505435	10.334432	216.06535	24.69782	16.274387	36.926144
DD109	20.041105	5.586181	0.3518447	138.20796	298.58292	38.894833	9.950706	49.29449
DD65	28.966606	1.7946553	1.0573692	303.15555	299.40387	24.259764	23.429932	40.062866
DD66	28.348831	33.07075	1.184515	9.025667	300.32425	20.578335	23.608797	22.714323
DD67	16.97215	1.3578235	49.388035	142.20706	252.29298	16.912685	28.356544	35.71931
DD69	34.700626	31.519476	4.930737	193.40077	277.85165	26.53625	24.43908	55.061615
DD68	31.221958	6.618057	1.5943066	8.549373	266.2848	16.577682	21.103926	48.257202
DD25	15.148359	3.67842	2.6954987	268.47305	274.51947	7.7883897	2.1890724	44.091568
DD28	36.299335	21.298298	1.4155178	332.65683	271.1161	39.473145	26.634346	36.278946
DD29	30.82721	7.3981323	0.2512386	172.00752	262.1257	44.377388	28.627378	43.478416
DD27	29.763552	47.761772	1.1733274	149.12416	221.14178	28.173874	4.5715914	34.861786
DD30	18.707811	5.2921896	0.5813771	249.59418	283.84543	4.7763934	7.6673436	40.158688

On-Line Supplementary Table 1

Animal	1395112_at	1395142_at	1395190_at	1395198_at	1395242_at	1395260_at	1395275_at	1395324_at
DD81	7.3731813	17.850677	76.50712	8.689481	23.884754	56.103634	28.301971	28.507309
DD84	7.4661837	11.866192	116.92931	4.434145	24.726746	43.7974	17.598143	30.099588
DD85	21.236542	11.287863	73.99368	1.5407801	24.221409	50.072235	12.689158	15.430635
DD86	12.350903	19.427242	23.97845	1.5736421	14.977177	60.2774	19.70428	30.108036
DD83	10.74805	13.809331	97.34705	3.153818	19.852757	58.22916	11.600554	15.158512
DD41	12.129364	53.13121	53.22952	3.5252705	37.470234	41.043797	17.315935	15.082414
DD42	4.635961	83.1647	76.35054	5.550324	49.49233	92.87786	17.095974	21.816576
DD43	3.363075	14.513844	26.46666	11.438554	28.4969	74.72827	11.868957	102.56465
DD44	6.9402843	7.9221377	26.793463	5.6998816	18.0056	69.92523	20.166256	67.9891
DD87	2.3051603	49.258343	27.61202	1.9712648	21.893179	58.660248	10.995666	21.191795
DD2	11.773048	18.980883	36.64079	2.925359	16.933586	38.516953	3.9416885	40.517548
DD3	8.117382	19.844793	18.1572	10.939183	12.718135	40.638058	10.36836	20.854235
DD89	0.6129215	17.352762	16.080254	12.871574	31.123589	33.91952	12.547107	20.332148
DD90	6.5614977	23.253044	7.305945	2.1710496	40.60028	32.16505	21.700027	20.936222
DD4	10.910903	18.218739	19.548191	13.140021	17.38155	48.317913	1.0793897	24.437355
DD47	8.855298	50.49644	36.403297	10.779972	15.011189	56.001392	15.270976	11.621033
DD48	23.1602	26.820911	61.130383	5.065964	20.165848	77.89808	24.547382	15.625638
DD49	6.224947	19.277758	12.35164	3.689655	12.446624	103.29151	2.630662	30.835487
DD50	1.1588459	14.281637	14.671171	6.417307	6.7334538	46.735504	11.071347	11.384689
DD46	6.0333843	21.00209	16.92777	5.085078	22.585272	67.40744	11.41417	26.204065
DD6	23.64263	20.602432	31.349348	3.2085989	12.26131	83.01337	19.666313	46.968693
DD7	7.824277	28.954031	6.855412	0.9428078	11.39708	42.175003	9.992654	22.671698
DD8	0.3268542	19.199894	10.731889	1.9829569	14.389105	68.034584	9.755792	26.47586
DD5	11.830216	14.363591	14.98964	1.7486769	13.604439	98.56379	20.561985	28.370464
DD9	2.5182707	20.487608	5.6733913	4.372004	11.082704	65.52889	7.5693035	25.23941
DD103	13.252447	13.448558	18.753576	14.91663	10.457685	151.87338	19.27121	60.71325
DD101	56.508083	15.774842	31.369217	9.194479	2.547927	143.7589	13.528844	25.037794
DD102	17.108562	14.696058	17.935057	19.704699	5.846129	163.71684	14.926834	84.92607
DD105	39.6555	14.489244	16.681774	16.531445	4.4072647	120.64761	29.584888	44.29527
DD106	33.664185	22.78719	14.962991	18.12888	1.3650149	141.028	16.940598	56.181313
DD62	59.23543	1.42258	9.61479	13.714677	12.290696	238.55261	17.137424	16.55912
DD64	40.349014	10.795756	6.7836013	12.850383	16.831398	122.2981	25.943	82.0062
DD107	12.480944	6.743289	8.189828	17.393845	14.5932	220.58592	20.87253	169.73737
DD108	13.725227	3.9510567	11.089057	22.133854	16.456377	273.47833	18.722664	94.96118
DD63	12.477386	8.752108	6.980658	18.067503	11.017017	243.67339	23.45338	94.3978
DD23	8.718968	11.590525	16.402927	21.419432	6.350559	242.84308	28.019524	45.05218
DD24	18.781387	8.948211	25.036993	26.24127	8.985838	202.06615	33.343185	36.18472
DD110	41.617413	7.6291285	9.945881	24.575731	3.3694525	251.87111	23.764147	36.612885
DD22	27.575926	6.2984157	13.985251	22.839638	12.886862	206.58632	21.36987	30.330355
DD109	8.867662	3.8040001	2.974732	29.083996	14.987124	252.05212	25.124758	37.057335
DD65	7.0618277	14.791849	10.024244	32.331383	8.059004	238.17427	25.241264	45.106777
DD66	9.951732	24.661276	30.651794	17.445684	20.616589	134.2811	19.535114	36.007767
DD67	17.2735	2.3350902	4.8104525	8.118496	22.03786	166.66971	21.477003	35.313072
DD69	1.5264405	11.956385	5.5194073	32.110573	10.527099	164.65958	23.01437	74.17246
DD68	15.390288	9.27177	15.175753	18.429413	11.193448	179.81844	22.25801	36.977013
DD25	1.5315623	45.25832	17.203901	30.089497	5.8484426	121.767	28.329008	59.117474
DD28	2.0439138	11.647452	3.3125503	17.962517	3.5234833	193.45909	25.009804	55.981026
DD29	7.0780106	33.387024	31.234499	17.3012	13.23827	190.73431	19.91271	48.36
DD27	17.893066	33.315456	20.65854	16.694464	8.195432	170.56042	19.367353	53.089363
DD30	0.7295569	42.98332	16.768734	29.585531	1.6972262	134.07944	23.78989	37.310925

On-Line Supplementary Table 1

Animal	1395361_at	1395423_at	1395585_at	1395680_at	1395703_at	1395714_at	1395732_at	1395762_at
DD81	61.033905	11.673031	1.5473965	45.134464	45.182003	1.3971317	32.641136	43.58363
DD84	70.59292	1.041721	1.704049	38.533016	45.794186	3.5789213	22.444515	33.885914
DD85	66.3042	9.07171	2.0092335	38.923656	63.7089	12.112731	37.578846	60.228092
DD86	66.93474	6.745844	1.7575387	25.456127	84.10921	1.1694629	45.87245	40.482056
DD83	52.60204	2.523931	13.261419	35.393898	44.4907	1.9722373	32.278694	36.075024
DD41	55.48685	1.3319145	6.6928644	24.681156	56.933216	10.824854	31.07036	52.759403
DD42	41.981575	9.911671	1.3203058	15.555679	81.87068	1.7440461	26.527227	61.084354
DD43	53.554256	18.801178	1.7868296	23.226488	51.24354	1.9185754	30.068588	73.47409
DD44	50.79219	6.240315	7.4913845	25.147203	56.620445	1.1393116	19.477764	44.305126
DD87	50.81568	9.479794	0.9555619	28.159153	42.239616	13.405136	40.708614	44.002163
DD2	45.0851	6.9564743	8.0411825	42.140644	64.61278	3.1711867	31.566092	43.00742
DD3	38.684025	0.6033489	5.480357	31.445913	101.2254	3.5545359	39.33228	29.906816
DD89	67.457306	8.53053	3.0951915	22.876036	81.517296	1.6032853	38.150414	44.686913
DD90	55.359818	16.586128	9.198496	30.80197	103.32912	1.4895381	37.375935	55.06887
DD4	61.189716	15.094557	1.6451184	21.581413	72.513664	3.4010816	35.38243	44.989864
DD47	45.733875	4.726798	0.9531213	17.670776	66.07703	5.409287	26.580198	23.356493
DD48	47.783085	3.4533372	9.599869	38.903534	58.36254	1.1726687	28.017883	38.416386
DD49	53.644196	2.2831118	1.4164777	33.17179	57.817963	2.0552363	45.369595	58.577385
DD50	80.63281	11.486162	1.1415999	20.65867	58.9352	0.8646131	36.123444	60.744278
DD46	49.688324	7.8892527	10.35519	33.27087	96.35307	1.1565676	21.035187	41.739017
DD6	55.118633	16.47337	1.051582	36.875065	50.53253	2.498757	26.157013	51.353294
DD7	53.937534	16.075891	0.9327435	36.297203	66.52966	1.2433686	32.07832	68.6602
DD8	35.2703	10.987742	1.3570296	18.360619	48.58778	3.1986678	33.658344	53.646267
DD5	42.628193	13.165927	10.146481	14.802513	58.164646	2.8239617	24.56276	51.247593
DD9	40.43575	13.866416	5.6273212	16.91096	82.06139	1.7007204	40.923977	45.23374
DD103	3.137527	48.658863	16.282682	9.20035	21.167496	1.6067736	13.914376	125.17249
DD101	12.873076	1.1796076	20.71771	12.96513	12.847736	6.119359	12.605821	110.06593
DD102	7.7843776	62.862614	21.64586	17.019457	20.127811	16.647099	15.443549	114.65393
DD105	16.466137	21.113861	26.944485	14.376061	9.1783495	9.6214075	15.688194	119.52441
DD106	12.155466	11.479039	17.407791	38.35606	17.346354	2.1210132	1.6229199	109.81634
DD62	11.578898	18.776482	17.857576	42.923462	21.011168	55.08559	2.0284712	42.281387
DD64	27.227001	6.7141733	19.255833	20.155432	44.0911	52.001442	10.27149	83.71091
DD107	13.173676	25.277687	21.747969	12.260403	26.549397	1.5302619	11.810765	152.5401
DD108	6.950457	38.110756	14.235536	14.030181	14.974089	2.1323214	16.341486	85.236015
DD63	2.9147952	52.812443	17.023205	10.864632	25.562248	2.3035083	20.383278	82.15183
DD23	15.17353	14.294994	27.460299	23.508224	23.880604	38.803654	24.97503	91.91633
DD24	9.1828985	30.62416	20.839441	28.148773	13.361564	35.24467	20.186516	144.29994
DD110	8.465776	7.1938133	11.727165	27.325573	26.655878	19.807137	19.001356	146.7502
DD22	9.840752	38.954166	10.664087	13.054029	29.561378	88.54708	25.2401	84.44432
DD109	7.7909784	26.965551	20.934269	17.816275	60.48303	67.47743	33.87405	107.70187
DD65	13.973984	8.537284	18.62114	19.408257	30.197542	78.11942	18.77499	119.76092
DD66	23.944994	5.5226216	11.754362	10.047236	19.799154	1.3011781	12.224014	41.930466
DD67	2.2219782	56.973164	15.10708	17.425217	33.503246	25.52086	10.679432	87.22029
DD69	35.682278	35.758095	17.70922	22.669455	22.79565	7.8762693	7.8773794	115.28304
DD68	1.6247876	34.23928	23.596441	1.1062795	46.997097	95.793594	1.2290133	52.406826
DD25	36.40714	50.807053	16.471512	2.665187	40.30852	90.78494	28.740429	81.68837
DD28	3.670056	28.885971	24.134739	22.463419	34.25571	5.2055774	12.553539	66.85813
DD29	5.2962327	43.125927	11.024744	3.5017045	30.360817	63.48506	2.914759	94.39391
DD27	38.72151	17.045765	29.329962	16.510576	45.32529	60.749424	25.044573	136.71889
DD30	23.985107	47.78464	13.246271	5.2618685	51.661198	126.09226	29.081535	98.39594

On-Line Supplementary Table 1

Animal	1395795_at	1395888_at	1395967_at	1396201_at	1396240_at	1396253_at	1396410_at	1396455_at
DD81	37.60758	10.046796	22.426344	4.6012	16.39894	21.822403	24.857891	6.1435018
DD84	31.443506	1.4684777	36.031376	22.345396	24.472649	18.458315	28.557966	6.2625175
DD85	8.478752	3.131989	12.098543	9.482816	18.279558	7.546664	24.541874	5.747614
DD86	22.919836	1.42143	23.452026	14.996802	35.947445	10.674412	21.52538	0.5607158
DD83	28.945576	2.1642091	22.47749	16.956657	15.768495	2.1380007	24.594603	3.3874447
DD41	29.806456	8.845813	9.268566	27.764103	10.446337	16.926285	40.388474	10.465103
DD42	14.00296	3.1409774	13.194685	71.06452	18.07905	14.64634	26.125788	3.9070368
DD43	58.358974	2.3035758	18.142832	22.487003	15.027642	14.570247	60.44891	8.539387
DD44	37.76822	12.462104	31.687347	25.93319	23.590466	15.108203	28.022284	42.260387
DD87	26.01807	12.155416	24.282515	21.196615	20.175486	18.07728	29.824743	7.1709976
DD2	40.952095	12.962031	21.769478	26.886913	18.69187	8.775164	34.200905	9.577862
DD3	20.682928	9.811933	39.281933	24.5359	14.908017	9.176214	16.340593	6.1118164
DD89	41.056194	2.0154054	15.889088	18.108707	9.768423	4.808651	24.444212	8.316846
DD90	32.47621	1.850033	13.85613	15.150309	17.289476	18.951849	33.30611	161.37589
DD4	22.884565	0.8660537	29.75534	23.56172	3.8112938	12.773202	34.745155	10.36329
DD47	16.196115	7.9416137	32.80803	33.98305	11.994546	1.9444598	7.9154205	1.0804532
DD48	38.254025	0.6924104	20.073652	20.678865	15.969404	8.97112	31.021627	11.505008
DD49	30.788937	10.328671	35.288765	13.411887	2.3498783	13.980258	32.668987	7.077886
DD50	24.591665	3.388694	28.185495	8.180993	22.063793	1.500331	28.551832	46.317154
DD46	36.823513	2.2232041	39.619324	31.623404	9.174139	14.462751	33.09983	8.321326
DD6	28.27225	1.5262891	14.771937	32.466743	17.141926	21.609283	50.343903	6.6276503
DD7	47.454163	5.5109353	19.660912	28.390507	11.891186	13.272319	55.53129	3.6581967
DD8	24.992655	0.8859911	12.464838	36.75006	4.1209846	17.87266	28.829056	5.1694636
DD5	11.143201	0.6900734	27.324215	16.802872	4.4855776	9.775894	19.50325	8.514588
DD9	29.634401	0.8549602	24.236355	33.784203	5.364414	8.375712	28.569164	4.2765117
DD103	13.930445	0.8400138	24.32083	24.2363	18.380365	35.782177	52.139725	4.0345473
DD101	10.008364	0.7596381	28.822666	38.49548	53.86976	18.455814	40.95143	0.9180583
DD102	10.228608	28.557081	43.11729	20.433582	46.281757	25.7679	75.294846	6.5161657
DD105	8.859468	1.6794969	33.45153	23.034906	40.138813	25.991003	60.36209	7.4698544
DD106	5.865073	16.796114	52.265244	64.24222	45.48798	24.671942	57.257763	0.5970109
DD62	2.460327	2.5901835	17.08545	43.54714	44.618427	44.758648	42.994965	50.009823
DD64	8.363417	44.28088	50.846676	45.343613	37.886303	31.26341	22.91461	104.34803
DD107	15.963423	22.48903	51.36914	18.046942	39.410942	52.27236	81.33293	74.70571
DD108	2.92787	7.9901285	49.867146	45.412483	30.021147	28.926115	60.34889	148.61345
DD63	12.217741	11.736783	52.220497	24.10341	21.848675	34.480923	61.99976	143.40869
DD23	11.291836	20.921803	66.19095	36.348553	25.9846	33.14719	40.765694	38.908497
DD24	15.51938	13.386818	34.057205	54.575253	25.342258	26.335318	53.738953	34.35327
DD110	8.273953	2.2123039	49.77255	36.24639	46.309746	33.138466	43.545616	197.49323
DD22	14.550955	1.2786815	37.92121	47.579067	34.019573	26.682627	30.021511	116.84484
DD109	4.635371	14.926684	38.45797	47.22278	26.772255	24.515547	54.211864	64.54019
DD65	12.94659	19.21724	51.763855	31.136189	23.815641	27.981169	61.616856	198.50015
DD66	8.669041	11.393662	32.061867	23.355688	28.194834	21.042944	31.442795	2.555283
DD67	5.876378	17.655848	49.757496	43.707176	20.219223	27.691475	62.55129	3.4422848
DD69	3.0566013	25.111994	66.019424	41.158714	25.828749	15.29722	28.902311	222.2944
DD68	9.564358	11.045341	76.242874	56.340492	1.554367	29.5346	57.62001	5.4297476
DD25	12.333646	10.977014	68.5112	42.359062	36.11968	35.360992	81.935356	15.939542
DD28	1.7086325	19.766459	34.808388	33.718655	1.6881663	6.4618864	50.332302	287.8051
DD29	10.986994	9.728702	93.40525	37.35719	15.251017	15.9803	71.32726	340.8438
DD27	6.564306	19.958818	60.250618	32.750744	25.26969	27.063433	83.27325	10.157505
DD30	1.0762987	12.17298	66.79442	37.355618	18.895882	27.344543	72.14969	13.345507

On-Line Supplementary Table 1

Animal	1396481_at	1396542_at	1396714_at	1396743_at	1396779_at	1396877_at	1396917_at	1396952_at
DD81	350.15152	4.7006416	58.532352	71.20599	8.645875	123.55282	94.11847	10.175348
DD84	420.15994	2.8334208	51.24015	35.260788	9.886798	102.60342	55.03139	1.9262328
DD85	395.3619	3.9637601	62.863068	43.697525	9.42058	81.48274	102.80679	1.5252539
DD86	9.886429	1.538833	48.772778	37.815193	1.491143	130.2568	158.91888	0.3626397
DD83	373.71262	2.0097177	53.07356	45.997112	1.0764059	51.134354	79.73718	5.9643493
DD41	87.79295	2.9741747	56.288334	33.249794	0.8972858	71.74362	95.11748	9.129213
DD42	19.440878	2.4317372	46.295925	28.860912	12.186246	54.60689	68.935776	9.64643
DD43	19.567434	12.947194	83.22378	40.32462	1.4273854	74.64609	105.35581	2.0629964
DD44	33.46224	17.263372	79.31129	34.145557	15.436626	59.834946	81.53292	4.865065
DD87	33.756668	1.6204232	51.692818	24.985174	1.9182858	66.440414	81.13755	8.495725
DD2	64.62073	3.598064	60.842667	44.1418	2.4004743	69.1717	65.1209	4.8026576
DD3	48.41633	4.3687725	73.6476	33.26313	8.9016285	76.4622	60.390007	2.9485936
DD89	29.677149	1.9417592	48.111446	42.182545	0.486677	133.325	31.107904	1.2565747
DD90	0.1635303	2.1132722	68.04934	44.45541	1.3961512	135.45576	55.793217	4.4304814
DD4	9.784288	0.9089036	82.635155	51.81956	3.1536717	104.52892	86.04956	4.8716154
DD47	2.9969308	11.405962	43.78663	14.464314	9.457682	76.50068	105.5183	3.643849
DD48	142.08495	10.61925	47.721817	40.399643	2.0307384	131.02835	85.5131	7.7813454
DD49	21.028328	1.4825109	58.37022	21.96127	0.8590594	61.473	78.47421	4.3305907
DD50	7.9375987	10.268824	58.42488	26.23994	6.0406957	89.36038	101.8468	5.453792
DD46	22.923616	7.272102	67.21482	33.52713	6.47738	80.46431	85.53632	4.2473683
DD6	120.07794	12.451478	80.285225	41.753433	10.89774	82.60297	92.141235	3.2063646
DD7	32.44399	4.508017	67.27266	53.909622	10.556406	81.6728	76.210495	4.0798197
DD8	3.528884	5.1812673	58.58344	38.02612	7.501682	80.23905	55.20845	2.2606359
DD5	8.8860855	1.5217177	52.738014	42.355747	10.933365	42.20429	64.55157	2.1916046
DD9	4.291418	2.0867922	72.54084	22.643116	1.6169888	68.19371	69.303215	4.1011972
DD103	141.9898	13.558143	4.136261	24.954315	20.922754	153.78075	4.82778	0.2191274
DD101	398.81854	13.547059	0.4983889	38.340744	26.69755	212.09406	12.685514	0.613923
DD102	148.65163	28.419468	2.0686288	26.194515	39.421642	139.4388	47.228386	6.4044757
DD105	378.39548	2.0052378	7.160219	11.443205	28.488157	254.56474	41.596935	5.8878736
DD106	440.0653	7.048164	4.095363	16.225958	26.850025	332.27692	47.29494	0.6657137
DD62	212.33617	2.2237322	7.168127	12.379902	26.477324	158.15013	11.012138	7.5107975
DD64	52.484894	2.6472595	32.859547	17.332281	38.588257	72.208336	19.585842	4.6617913
DD107	19.715614	41.916626	1.0578427	16.015816	39.768833	72.06356	68.49991	2.3276346
DD108	36.58772	19.139006	4.759888	6.862504	40.749626	165.74538	25.713228	0.7808283
DD63	27.307709	30.167309	11.694679	4.6846013	37.327316	155.62134	57.07871	5.5194335
DD23	7.6682887	19.708706	3.7647612	3.188807	34.98146	209.28455	47.29375	0.3544215
DD24	8.12593	7.243748	3.6375084	7.606548	32.04798	182.08168	7.641756	5.476022
DD110	33.179695	12.01191	0.4770056	24.295202	31.442842	210.59715	63.26534	0.287838
DD22	22.779552	4.567019	3.8406029	9.919828	28.71979	159.91779	2.3517075	3.7103035
DD109	6.420603	6.159415	8.207613	24.571064	27.709246	317.25076	38.389835	1.396829
DD65	8.355246	18.935013	6.7649865	28.812874	31.418636	284.86932	9.014725	0.8105724
DD66	4.255796	10.227126	44.733242	5.8824553	31.22307	161.7062	11.603143	9.086651
DD67	7.751602	4.701191	4.8098083	34.311104	26.986567	242.09923	36.720882	0.7784866
DD69	3.5500755	10.860969	35.35067	29.839119	39.373253	96.08603	8.72932	0.8390509
DD68	1.3204868	4.714345	21.53221	10.559541	35.805233	202.47697	2.5668523	3.997117
DD25	4.9157577	7.7590313	4.357144	8.809092	43.75934	231.32591	64.34721	13.142531
DD28	2.8092544	7.8475533	22.80086	28.308275	33.761154	150.1109	40.228092	1.117527
DD29	1.6695697	11.748568	7.022673	16.086653	32.705025	195.5454	47.45425	9.284406
DD27	13.926111	23.3054	31.707914	30.25902	31.584156	208.63077	14.319681	6.4387956
DD30	1.2092756	2.670459	7.334692	5.591103	39.07896	219.37534	75.37872	14.925436

On-Line Supplementary Table 1

Animal	1397153_at	1397206_at	1397215_at	1397216_at	1397218_at	1397229_at	1397271_at	1397296_at
DD81	23.268204	30.63578	63.14777	57.19655	44.604088	6.4999285	41.438778	26.121178
DD84	21.06083	2.1993883	50.361534	55.067142	54.389206	4.6432886	24.421993	23.15641
DD85	14.513385	21.978697	45.835487	69.47623	39.846973	1.402847	14.429099	41.16154
DD86	20.808807	19.393076	79.69853	100.26609	42.480835	4.72845	2.7910447	42.29464
DD83	15.028612	20.798424	54.60647	65.101494	46.64186	7.420574	26.374647	23.489052
DD41	26.882168	23.270119	80.28491	129.90758	34.158268	13.067814	18.592127	19.824514
DD42	22.686409	15.779748	71.44283	89.40286	30.21925	6.396129	2.7225566	27.243483
DD43	17.5549	29.792477	71.50012	76.912704	42.86172	10.600375	246.06429	5.2234306
DD44	26.175297	17.797573	64.37518	91.791245	43.002712	8.581353	156.71658	20.135586
DD87	18.250715	13.277556	69.81477	108.98823	24.584488	1.3431457	14.580863	18.830103
DD2	20.994154	12.255082	80.28602	66.620865	18.479425	6.3497334	146.19633	15.3071
DD3	18.221437	13.757951	78.16905	91.838806	41.51378	6.145356	10.825243	16.47974
DD89	14.8135	10.444096	100.59171	87.319626	17.461668	14.825265	21.321732	53.505352
DD90	20.796272	18.40134	143.87306	101.38577	26.587648	14.473826	16.655396	47.44702
DD4	18.718163	22.928116	113.92974	90.41987	21.78652	6.207622	14.125794	36.89702
DD47	0.7959007	27.68679	106.26883	73.93856	39.28721	2.963747	8.592005	30.827106
DD48	16.180906	20.06149	114.69984	62.617905	27.851967	10.020898	3.8059227	23.965942
DD49	21.328989	16.237764	111.73854	73.8205	33.533463	2.0466278	1.8686584	45.59138
DD50	24.058949	18.673256	121.42611	58.691414	25.154293	1.8401994	20.37918	44.22495
DD46	19.179628	15.8691	105.77065	89.03428	26.038897	10.042015	4.46843	18.273048
DD6	16.532604	22.180513	98.86049	89.60155	16.932478	7.151156	52.050407	30.470228
DD7	15.367084	18.816124	132.48146	114.72107	16.957144	9.740084	32.516182	33.36112
DD8	15.583106	15.408403	144.87625	140.17351	22.017963	9.0943775	5.7312717	21.186823
DD5	21.863838	23.148878	113.71156	110.53641	29.941296	3.5221286	11.473444	31.815205
DD9	22.45367	18.641535	124.6796	102.56915	26.826788	8.7329645	17.771696	30.71706
DD103	79.07077	66.886345	33.056408	37.887764	56.257782	15.67332	45.36725	35.17405
DD101	40.23994	40.88707	26.57408	37.176056	64.85187	17.246172	31.319138	40.869816
DD102	90.86265	70.12137	44.244743	35.626595	53.598274	13.446653	119.02741	47.445736
DD105	73.899055	25.153027	44.427505	22.115902	60.68831	12.243064	21.166265	52.636932
DD106	49.518307	42.343845	40.335846	33.035477	74.17908	9.773762	41.75059	31.465128
DD62	45.59893	17.934132	46.143288	69.3816	54.36664	6.742392	39.349617	38.26576
DD64	85.614	36.804314	39.760216	41.404682	60.95123	11.832776	93.73147	29.770851
DD107	145.95311	48.1966	43.17316	39.905468	32.256683	13.89514	398.19272	41.509434
DD108	86.487785	26.644522	43.77115	51.426884	82.992035	14.746781	101.52026	38.06057
DD63	77.57168	31.584105	39.762966	55.953262	84.55503	16.58167	113.62199	30.58209
DD23	101.01067	31.628744	45.905663	68.24193	55.83125	21.414492	61.947197	63.16489
DD24	73.9505	44.087814	38.781883	58.25675	73.32474	22.511326	23.4413	71.51448
DD110	99.010635	51.70536	45.990067	61.87645	71.698456	21.425398	21.155125	57.436684
DD22	67.27242	35.181484	33.75047	75.33989	52.81066	17.207626	7.911799	51.24009
DD109	71.92876	8.617671	44.271465	66.877144	65.509636	11.386539	0.990133	60.508064
DD65	68.30767	35.972763	38.184975	61.063538	77.562416	0.6724888	23.32774	61.945736
DD66	54.86104	35.709724	44.48766	63.78561	75.704956	15.259667	8.910424	60.90399
DD67	52.097485	19.65089	40.697994	57.141476	50.50208	11.803803	0.3542185	53.889034
DD69	108.74352	31.633297	38.798737	90.64303	73.17162	11.78083	54.2703	63.34089
DD68	81.313194	21.277452	52.446873	79.925545	113.32826	18.1688	57.360695	88.121994
DD25	88.29431	76.381775	44.803734	50.130352	72.18699	5.2123213	10.303558	54.06438
DD28	83.71805	27.059605	49.322075	70.95956	111.40614	3.4022646	8.526432	56.990425
DD29	69.23296	19.207758	37.361153	56.13577	138.1724	15.143435	8.471833	38.72902
DD27	117.14559	44.23377	44.091347	31.453999	70.426186	22.880898	243.3393	59.9239
DD30	82.344955	85.104126	43.0089	64.350136	65.8797	13.195568	16.831419	58.623493

On-Line Supplementary Table 1

Animal	1397366_at	1397435_at	1397449_at	1397855_at	1397859_x_	1398049_at	1398050_at	1398057_at
DD81	78.16354	4.8525653	62.36526	448.54376	2.8061852	89.53612	6.0742764	18.656841
DD84	56.200447	9.815051	53.781654	378.34344	1.3611523	67.00984	7.4960437	17.059605
DD85	91.84524	11.000799	44.74216	379.40445	0.4970964	81.05089	4.294777	18.52951
DD86	9.398181	3.1536787	45.473686	26.54236	1.3980137	64.9842	8.420984	21.546505
DD83	107.49168	8.904693	46.46176	406.4069	3.813001	55.785892	6.0368576	27.433104
DD41	43.67224	11.372701	53.29392	122.0466	2.5019283	81.507866	14.913754	16.414215
DD42	25.076653	4.5361357	80.122795	44.340385	1.1525071	95.16561	3.7019238	14.948524
DD43	6.5455728	8.15719	39.165123	45.16141	6.35885	70.299484	5.187217	16.77738
DD44	35.808716	4.7362337	32.103687	75.15279	12.39261	65.21472	4.4049397	18.159733
DD87	28.797785	5.4855676	58.60308	56.389343	5.5492167	58.738495	12.791041	27.658787
DD2	138.08261	7.311027	41.590935	217.11868	3.8070958	63.6086	2.3850527	16.995304
DD3	8.480231	7.8365674	45.242573	41.291645	8.337352	55.786667	6.5665317	17.762592
DD89	33.363037	8.823112	32.3737	80.08841	2.543333	79.24419	0.4420076	15.143112
DD90	5.9025064	5.21616	31.941727	11.092561	10.091547	68.89183	0.6743096	17.597002
DD4	22.185684	5.9388776	33.793644	43.80253	7.766305	62.00109	13.629939	23.581884
DD47	27.007788	4.8223877	64.51105	7.136507	12.522827	61.96615	7.81287	16.168692
DD48	137.20512	8.50817	50.95031	187.14487	9.901181	77.945366	5.209826	19.282635
DD49	12.947619	8.102073	35.946	35.34727	10.384214	59.412117	6.3407073	14.694849
DD50	18.863522	1.959714	33.881615	31.822456	12.646674	70.76949	13.622903	9.3929615
DD46	27.267138	4.6643376	24.159775	71.419106	8.633893	44.21479	0.6919715	15.419397
DD6	76.9594	11.054817	25.561455	182.90694	19.263405	64.12179	1.0839136	17.824837
DD7	34.37692	5.1550803	18.666872	40.624287	8.952615	60.844658	0.4363829	15.601811
DD8	5.005803	7.586551	25.809368	1.0722595	12.316172	67.72751	0.7470605	12.994557
DD5	38.040142	5.100441	24.514153	16.898403	9.227801	50.433422	5.5919156	20.053524
DD9	15.216925	5.3113394	18.39731	4.295382	16.370537	49.95631	4.7452707	15.495251
DD103	16.726755	18.004795	20.567287	76.254425	17.40948	38.257042	2.9638512	28.06332
DD101	84.76567	13.074372	14.974733	293.87015	51.238003	18.796047	7.6919594	17.518667
DD102	22.795229	11.811445	27.664244	97.85786	11.082954	35.58131	7.0963707	29.89928
DD105	76.0451	16.379684	48.619267	251.81009	9.859181	34.51606	5.641958	39.996845
DD106	60.583206	13.471141	11.547245	260.70947	61.308598	36.383026	1.4781574	20.734737
DD62	55.50574	13.255141	18.266201	378.64496	66.64818	22.79471	19.011055	44.350414
DD64	49.94317	11.262087	23.997892	164.08418	107.98653	16.79559	15.632283	58.04622
DD107	21.726192	18.329948	29.210392	44.173187	219.59254	41.589745	23.131947	15.814655
DD108	23.542768	11.589675	26.957882	61.009853	55.69518	28.633936	8.359212	24.410864
DD63	21.578573	15.12287	29.897802	49.4592	54.41964	21.88934	13.687553	29.080524
DD23	7.527729	13.159036	21.916254	25.483864	21.067947	26.610855	26.023466	54.336086
DD24	9.641514	13.055437	12.887033	23.267067	84.30456	29.434021	15.096428	42.37206
DD110	23.477375	14.955047	15.734947	100.66837	102.99876	36.647423	9.684622	37.06866
DD22	25.998053	11.824173	14.649291	51.21634	24.457268	29.224632	9.639036	19.785664
DD109	9.651971	7.543678	22.2063	12.996501	27.05995	24.930777	31.463045	43.084606
DD65	10.142272	13.998882	25.132551	27.280823	245.73567	23.46078	22.720482	45.23705
DD66	3.3018088	20.856165	51.38032	2.009067	104.58996	30.488956	9.9546175	31.139055
DD67	5.1037083	7.584262	0.6058239	28.102804	70.03587	26.949104	19.368427	31.333918
DD69	3.8402317	14.173425	11.483939	8.746254	135.9562	20.882421	13.85505	39.98286
DD68	9.230856	8.192453	17.493872	11.850117	133.23026	26.17318	11.993426	45.182858
DD25	15.190796	6.6488166	67.50015	0.596252	35.33662	32.459778	18.377329	62.689873
DD28	4.816365	8.913107	6.056589	16.40655	94.608345	28.39853	12.056725	19.793497
DD29	5.164623	12.553597	25.166706	0.585132	107.85619	23.110273	6.8717985	34.15439
DD27	24.121687	13.066361	35.36929	25.491074	34.571335	35.335205	13.626083	24.156511
DD30	9.332951	19.810152	64.778114	0.9160132	35.461975	38.522938	22.127737	57.16038

On-Line Supplementary Table 1

Animal	1398243_at	1398248_s_	1398261_at	1398528_at	1398551_at	1398566_at	1398581_at	1398612_at
DD81	1494.3816	2431.9663	3.1177447	8.642545	139.25133	62.03676	16.32301	170.37659
DD84	932.8671	2392.8633	50.147102	3.2401316	191.63344	65.4733	26.76118	161.89244
DD85	833.9588	2402.1328	34.789207	9.487614	172.52855	93.74785	29.966276	166.72182
DD86	40.230946	107.75862	19.16596	7.4512196	185.55946	80.50804	2.1383815	263.09106
DD83	1009.6714	2244.9727	14.44106	7.7143984	178.8136	57.15143	20.465818	173.19449
DD41	229.11076	608.5861	47.571175	10.532491	180.34099	105.94746	28.268597	366.71368
DD42	113.25481	151.26628	44.743824	11.765768	278.5616	55.660976	16.465197	289.7039
DD43	83.76634	164.90575	49.91408	7.0204053	184.31381	78.1374	5.6035743	278.81873
DD44	99.68174	171.44888	34.00346	6.924801	226.6358	62.846897	21.797382	313.335
DD87	89.74377	167.3125	19.60084	5.702485	241.46156	79.17078	17.051281	347.777
DD2	560.28674	1397.7125	15.741176	8.268206	18.38318	156.4299	22.56892	302.14935
DD3	151.71713	285.6914	34.940556	4.046634	6.0355673	177.2589	9.047255	397.75455
DD89	132.07867	168.49362	12.968188	8.364858	15.781355	169.77885	20.257877	496.20255
DD90	16.117392	23.621176	3.5659113	10.956528	5.9973125	179.56786	28.247482	639.0091
DD4	57.973846	130.13303	6.4444594	7.9216747	6.441876	143.24884	20.853882	530.5584
DD47	50.761654	127.31846	21.689959	1.1516364	22.683962	196.07674	19.382504	356.34277
DD48	733.22253	1842.7866	33.578236	5.716131	3.8427942	135.27777	29.80934	372.67462
DD49	62.468243	155.37	44.269527	4.008059	3.6622503	177.9172	9.7625885	441.90668
DD50	42.951546	148.01404	2.918897	14.134998	3.1810324	227.50555	1.4640474	541.52576
DD46	108.62127	285.11026	26.301126	4.1095767	8.662143	206.69849	21.309307	522.1509
DD6	307.12823	653.89606	15.993153	9.06707	3.6818762	197.21126	36.078873	498.19394
DD7	147.54271	129.06912	2.5275087	7.58288	16.29714	280.71896	25.813951	644.387
DD8	10.552584	3.778471	19.798979	4.887984	4.0071626	203.84116	24.600899	657.66614
DD5	36.410934	54.53764	13.190532	14.512037	4.611474	153.58015	19.377705	657.2169
DD9	15.050943	27.198261	2.1412184	8.72659	12.192248	213.10358	21.20704	621.5831
DD103	324.96838	981.1967	57.288853	24.777124	324.07834	27.21551	5.276124	69.271515
DD101	983.4682	2229.1333	43.85797	71.03674	169.51605	35.480705	16.32089	44.387688
DD102	461.8457	1239.4008	67.850365	18.144388	95.276825	23.452724	1.5315319	57.20505
DD105	1338.1183	2400.36	40.574245	53.03653	125.80051	47.626026	21.778282	60.830288
DD106	1245.4614	1951.1896	43.249237	69.85445	149.6517	33.797066	13.696438	68.00198
DD62	701.6324	1298.8347	31.10812	20.298761	55.18299	39.11291	27.248653	228.05266
DD64	145.50749	261.5465	40.84541	31.067951	35.79717	34.832504	13.986571	229.2532
DD107	37.065346	79.69232	11.510637	24.521067	5.209803	36.748154	15.286256	287.28683
DD108	161.4175	253.588	38.56844	30.855904	103.65963	31.705677	2.1395247	141.28064
DD63	161.69391	280.3868	39.54787	16.98802	108.24494	28.273874	2.2577736	132.94186
DD23	31.462711	50.73872	43.388042	21.219624	88.28468	57.718376	6.8682	362.02982
DD24	85.17324	167.11296	52.919483	21.65355	133.71083	51.710327	6.8843307	361.87692
DD110	23.521406	31.054977	32.275684	27.610743	26.15224	51.820538	17.99611	144.37192
DD22	184.36244	173.80994	47.54136	31.988867	66.3125	50.743233	0.7446376	343.46207
DD109	14.431211	45.597626	20.779	26.748835	37.265114	90.54127	4.3008065	150.18407
DD65	10.405816	47.29215	15.238623	27.916037	29.619232	50.90573	3.5478125	380.211
DD66	23.472622	50.74541	16.345886	25.931578	54.031624	74.65905	0.901767	498.61008
DD67	62.86612	100.66086	19.513594	13.243685	117.0506	11.00155	0.7174197	126.60852
DD69	2.601442	41.71985	43.551083	41.40642	29.035152	46.078026	1.4923725	362.46783
DD68	34.074635	37.034412	29.613052	28.58724	6.904064	55.71171	10.392089	148.99692
DD25	3.416431	3.565288	7.5637527	20.846018	16.797106	49.347107	11.964822	344.32794
DD28	14.226464	8.215191	18.818735	35.128	16.175259	47.525078	8.612172	331.53748
DD29	2.594747	4.0289464	15.635466	36.999302	46.766113	39.204464	8.5785055	108.57798
DD27	15.012842	31.382366	52.43792	2.490058	5.6710644	31.50647	12.139375	102.16494
DD30	2.6722903	2.2562892	19.097332	24.728632	16.92845	60.084377	6.062532	333.03006

On-Line Supplementary Table 1

Animal	1398623_at	1398655_at	1398720_at	1398821_s_	1399073_at
DD81	62.68094	315.0049	13.035479	782.5678	68.858536
DD84	68.903625	233.12462	20.740522	815.7539	118.43695
DD85	77.0182	133.98885	20.045786	655.08575	102.56538
DD86	72.27244	9.0604725	28.340324	36.78048	152.45537
DD83	70.9892	196.27208	18.753922	698.6272	99.8995
DD41	51.146675	105.6097	35.210266	300.38333	180.40347
DD42	58.356564	29.822388	39.56132	109.20239	341.81256
DD43	48.161037	40.471428	45.90551	127.426	222.57262
DD44	33.93191	59.687416	13.240483	135.84433	201.55399
DD87	61.76648	26.185099	29.627748	149.10695	328.24332
DD2	12.797403	119.41221	22.217354	692.6369	171.62791
DD3	32.783314	45.008335	48.607178	234.10901	171.51135
DD89	26.448896	62.233707	35.82947	195.58531	41.218636
DD90	26.250076	5.7973685	32.310978	29.834194	11.290837
DD4	18.260118	23.389376	84.82714	107.65356	26.3772
DD47	18.350128	9.923116	25.24404	52.16915	157.24074
DD48	10.866239	166.27423	3.8567665	1064.3264	204.16873
DD49	20.257929	32.45164	27.078976	175.72162	234.75581
DD50	16.95151	12.522122	28.297329	105.56055	130.44598
DD46	19.367512	43.348316	3.235268	268.94202	128.66072
DD6	13.059859	106.34703	11.381741	536.0505	90.484634
DD7	11.312624	29.672215	36.79971	145.6111	33.141457
DD8	18.801266	5.332949	41.277058	18.405022	79.99268
DD5	13.43885	17.57601	33.30611	33.96196	87.89827
DD9	13.030667	1.4311254	29.769243	21.961304	90.26223
DD103	66.64482	58.861668	10.395968	178.36096	348.85733
DD101	83.726295	262.92905	1.0665222	614.9294	319.5777
DD102	57.092926	93.22095	3.3205597	230.49689	305.88483
DD105	44.564484	272.43228	5.074642	500.67883	308.71216
DD106	72.814766	276.74384	2.79449	511.1683	342.4014
DD62	42.655502	177.2338	11.734354	755.5678	265.96448
DD64	56.2053	63.78556	11.391118	236.04547	273.95685
DD107	39.369034	20.357822	1.0592142	73.470955	255.90062
DD108	36.4492	34.055645	3.1001422	124.25731	302.24738
DD63	30.258259	35.51208	2.5093896	134.82881	303.4777
DD23	45.945496	2.161695	1.8280871	38.059017	333.8459
DD24	46.765366	23.681534	2.6550977	65.89639	357.4508
DD110	40.982845	62.91587	4.255901	221.43951	315.33023
DD22	35.499783	55.417625	1.828973	132.27519	344.2465
DD109	46.546055	10.023985	7.962739	49.510353	292.82083
DD65	54.840614	16.682255	3.1949112	58.936207	241.10037
DD66	48.63212	1.0676988	3.4671419	25.507662	313.7802
DD67	77.73957	30.780647	1.6987983	98.450134	355.07178
DD69	35.35407	7.852113	5.3861346	34.51239	282.49573
DD68	24.73519	9.993273	3.208789	35.74514	347.80057
DD25	37.615562	4.336717	1.2923594	13.691367	266.36365
DD28	20.1489	1.5474876	1.402427	24.428017	290.62985
DD29	50.75996	7.451982	2.8430934	14.016226	271.45175
DD27	31.25646	26.841743	2.5665977	131.45006	293.102
DD30	36.24368	1.3371449	3.1455433	7.048259	339.7762
