# Supplementary material for: Adipose Tissue Deficiency and Chronic Inflammation in Diabetic Goto-Kakizaki Rats
Source: PLoS One. 2011 Feb 25;6(2):e17386. doi: 10.1371/journal.pone.0017386 (PMC3045458; doi:10.1371/journal.pone.0017386)
Supplement: Table S2 — Differentially Expressed Probe Sets by Function. (DOC) [file pone.0017386.s003.doc]

**ON-LINE SUPPLEMENTARY TABLE**

| Table 2. Differentially Expressed Probe Sets in Adipose Tissue of GK Rats. | | | | | | |
| --- | --- | --- | --- | --- | --- | --- |
|  | | | | | | |
| A. Immune/Inflammatory (103 probe sets, 98 genes) | | | | | | |
| **Age** | **Strain** | **Probe ID** | **Gene Name** | **Symbol** | **Accession** | **Gene Function** |
|  |  |  |  |  |  |  |
| 5 | WKY | 1369764_at | complement component 4 binding protein, alpha | *C4bpa* | NM_012516 | regulation of complement response |
| 5 | WKY | 1373889_at | immunoglobulin superfamily, member 7 | *Igsf7* | BF387360 | immunoglobulin |
| 5 | WKY | 1388046_at | integrin alpha M | *Itgam* | AF268593 | regulation of leukocyte adherence |
| 5 | WKY | 1370382_at | major histocompatability antigen, Class II | *Mhc* | BI279526 | antigen processing & presentation |
| 5 | WKY | 1370428_x_at | major histocompatability antigen, Class I | *Mhc* | AJ249701 | antigen processing & presentation |
| 5 | WKY | 1370429_at | major histocompatability antigen, Class I | *Mhc* | L40362 | antigen processing & presentation |
| 5 | WKY | 1370822_at | major histocompatability antigen, Class II | *Mhc* | AF307302 | antigen processing & presentation |
| 5 | WKY | 1371033_at | major histocompatability antigen, Class II | *Mhc* | AI715202 | antigen processing & presentation |
| 5 | WKY | 1371171_at | major histocompatability antigen, Class I | *Mhc* | M10094 | antigen processing & presentation |
| 5 | WKY | 1379357_at | major histocompatability antigen, Class I | *Mhc* | AI408767 | antigen processing & presentation |
| 5 | WKY | 1383449_at | major histocompatability antigen, Class I | *Mhc* | AA801218 | antigen processing & presentation |
| 5 | WKY | 1388071_x_at | major histocompatability antigen, Class I | *Mhc* | M24024 | antigen processing & presentation |
| 5 | WKY | 1388202_at | major histocompatability antigen, Class I | *Mhc* | BI395698 | antigen processing & presentation |
| 5 | WKY | 1389734_x_at | major histocompatability antigen, Class I | *Mhc* | BI282965 | antigen processing & presentation |
| 5 | WKY | 1393108_at | major histocompatability antigen, Class I | *Mhc* | BM387813 | antigen processing & presentation |
| 5 | WKY | 1397859_x_at | major histocompatability antigen, Class I | *Mhc* | BI291927 | antigen processing & presentation |
| 5 | WKY | 1391258_at | major histocompatability antigen, Class I | *Mhc* | AI178337 | antigen processing & presentation |
| 5 | WKY | 1369171_at | Macrophage stimulating 1 (hepatocyte growth factor-like) | *Mst1; E2F2* | NM_024352 | serine-throenine kinase; tumor suppression |
| 5 | WKY | 1394846_at | paired-Ig-like receptor A2 | *Pira2* | AI237640 | immune regulator |
| 5 | WKY | 1368128_at | phospholipase A2, group IIA (platelets, synovial fluid) | *Pla2g2a; sPLA2* | NM_031598 | mediator of inflammation |
| 5 | WKY | 1392788_at | sprouty protein with EVH-1 domain 1 | Spred-1 | AA944323 | negative regulator of inflammation |
| 5 | WKY | 1385465_at | sialic acid binding Ig-like lectin 5 | *Siglec5* | BG379188 | sialic acid-recognizing receptor; neutrophils |
| 4 | WKY | 1387902_a_at | immunoglobulin kappa chain, constant region | Igk-C | L22655 | immunoglobulin |
| 4 | WKY | 1367942_at | acid phosphatase 5, tartrate resistant | *Acp5; Trap* | NM_019144 | macrophages; preadipocyte proliferation & differentiation |
| 4 | WKY | 1368637_at | caspase recruitment domain family, member 9 | *Card9* | NM_022303 | activation of NF-kappaB signaling |
| 4 | WKY | 1368167_at | cathepsin E | *Ctse* | NM_012938 | antigen processing & presentation |
| 4 | WKY | 1395073_at | ecotropic viral integration site 1 | *Evi1* | BI288681 | chemokine signalling; lymphocyte regulation |
| 4 | WKY | 1391207_at | family with sequence similarity 19 (chemokine (C-C motif)-like), member A5 | *Fam19a5* | BF557676 | chemokine activity |
| 4 | WKY | 1388255_x_at | major histocompatability antigen, Class I | *Mhc* | AJ243338 | antigen processing & presentation |
| 4 | WKY | 1371209_at | major histocompatability antigen, Class I | *Mhc* | AJ243338 | antigen processing & presentation |
| 4 | WKY | 1379496_at | major histocompatability antigen, Class I | *Mhc* | AI029460 | antigen processing & presentation |
| 4 | WKY | *1372958_at | OTU domain, ubiquitin aldehyde binding 1 | *Otub1* | BE111986 | inhibition of cytokine signalling |
| 4 | WKY | 1379065_at | ser/cys peptidase inhibitor, clade A (alpha-1 antiproteinase, antitrypsin), member 12 | *Serpina12; Ol-64; Vaspin* | BI295014 | inflammation; serine protease inhibitor |
| 4 | WKY | *1376562_at | TRAF2 and NCK interacting kinase | *Tnik* | BI291396 | activation of stress-activated protein kinase/c-Jun |
| 4 | WKY | 1393791_at | T cell receptor associated transmembrane adaptor 1 | *Trat1* | AI044622 | T cell receptor signaling |
| 3 | WKY | 1376373_at | butyrophilin-like 7 | *Btnl7* | AI012393 | negative regulator of T cell proliferation |
| 3 | WKY | 1368000_at | complement component 3 | *C3* | NM_016994 | regulation of complement cascade |
| 3 | WKY | 1391925_at | chemokine (C-C motif) ligand 19 | *Ccl19* | AA996885 | chemokine activity; lymphocyte recirculation and migration |
| 3 | WKY | 1393929_at | chemokine (C-C motif) receptor 6 | *Ccr6* | AI045155 | T-cell migration & recruitment |
| 3 | WKY | 1387472_at | CD3 molecule delta polypeptide | *Cd3d* | NM_013169 | T-cell development |
| 3 | WKY | 1387749_at | Cd79b molecule, immunoglobulin-associated beta | *Cd79b* | NM_133533 | B-cell receptor signaling |
| 3 | WKY | 1392171_at | chitinase 3-like 1 | *Chi3l1* | AA945643 | pathogen defense |
| 3 | WKY | 1388233_at | cytokine inducible SH2-containing protein | *Cish* | AF065161 | inhibition of cytokine signalling |
| 3 | WKY | *1379446_at | complement receptor 2 | *Cr2; CD21* | BF282187 | regulation of complement cascade |
| 3 | WKY | *1381327_a_at | complement receptor 2 | *Cr2; CD21* | AI556056 | regulation of complement cascade |
| 3 | WKY | *1385700_at | complement receptor 2 | *Cr2; CD21* | BF545268 | regulation of complement cascade |
| 3 | WKY | *1392233_at | complement receptor 2 | *Cr2; CD21* | AI556056 | regulation of complement cascade |
| 3 | WKY | 1391072_at | Fc fragment of IgE, low affinity II, receptor for (CD23) | *Fcer2* | AI410068 | B-cell activation & differentiation |
| 3 | WKY | 1382950_at | guanylate binding protein 4 | *Gbp4; Gbp3* | AA901350 | IFN gamma regulated |
| 3 | WKY | 1368337_at | glycosylation dependent cell adhesion molecule 1 | *Glycam1* | NM_012794 | regulation of lymphocyte adhesion |
| 3 | WKY | 1370394_at | gamma-2a immunoglobulin heavy chain | *IgG-2a* | L22654 | immunoglobulin |
| 3 | WKY | 1388272_at | immunoglobulin heavy chain 1a (serum IgG2a) | *Igh-1a* | AI411947 | immunoglobulin |
| 3 | WKY | 1388166_at | immunoglobulin heavy chain 6 | *Igh-6* | AI411693 | immunoglobulin |
| 3 | WKY | 1388277_at | immunoglobulin lambda chain complex | *Igl* | AI029631 | immunoglobulin |
| 3 | WKY | 1370967_at | immunoglobulin lambda chain, constant region 2 | *Igl-C2* | BG374683 | immunoglobulin |
| 3 | WKY | 1384218_at | immunoglobulin G heavy chain | *LOC362795* | AW534335 | immunoglobulin |
| 3 | WKY | 1388181_at | immunoglobulin delta heavy chain constant region | *LOC641523; IgD* | J00741 | immunoglobulin |
| 3 | WKY | 1387839_at | major histocompatability antigen, Class I | *Mhc* | NM_012646 | antigen processing & presentation |
| 3 | WKY | 1388203_x_at | major histocompatability antigen, Class I | *Mhc* | BI395698 | antigen processing & presentation |
| 3 | WKY | 1378193_at | membrane-spanning 4-domains, subfamily A, member 7 | *Ms4a7* | AI408286 | regulation of B cells; signal transduction |
| 3 | WKY | 1388056_at | 2-5 oligoadenylate synthetase 1B | *Oas1b* | AF068268 | IFN regulated; viral resistance |
| Table 2. Cont’d. | | | | | | |
| A. Immune/Inflammatory (103 probe sets, 98 genes) | | | | | | |
| **Age** | **Strain** | **Probe ID** | **Gene Name** | **Symbol** | **Accession** | **Gene Function** |
|  |  |  |  |  |  |  |
| 3 | WKY | *1399073_at | OTU domain, ubiquitin aldehyde binding 1 | *Otub1* | BI274378 | inhibition of cytokine signalling |
| 3 | WKY | 1379994_at | polycystic kidney and hepatic disease 1-like 1 | *Pkhd1l1* | AI070694 | possible role in cellular immunity |
| 3 | WKY | 1393297_at | POU class 2 associating factor 1 | *Pou2af1* | BG377979 | B-cell-specific transcriptional coactivator |
| 3 | WKY | 1388102_at | prostaglandin reductase 1 | *Ptgr1* | U66322 | inhibition of inflammatory response |
| 3 | WKY | 1383407_at | SAM and SH3 domain containing 3 | *Sash3* | BM390872 | lymphocyte signaling adapter protein |
| 3 | WKY | 1379653_a_at | SH2 domain protein 1A | *Sh2d1a; SAP* | AI176327 | inhibition of T-cell expansion |
| 3 | WKY | 1388275_at | T-cell receptor beta chain | *Tcrb* | AW919577 | T cell specific antigen recognition |
| 3 | WKY | 1397271_at | tumor necrosis factor receptor superfamily, member 13c | *Tnfrsf13c* | AA851313 | regulation of mature B-cell survival & response |
| 3 | WKY | 1376327_at | tumor necrosis factor receptor superfamily, member 14 (herpesvirus entry mediator) | *Tnfrsf14; Hvem* | AI169601 | lymphocyte activation |
| 3 | WKY | *1380180_at | TRAF2 and NCK interacting kinase | *Tnik* | AW528635 | activation of stress-activated protein kinase/c-Jun |
| 3 | WKY | 1393809_at | Tnf receptor-associated factor 6 | *Traf6* | BE115919 | cytokine signaling adaptor protein |
| 3 | WKY | 1368762_at | ubiquitin D | *Ubd* | NM_053299 | antigen processing & presentation; IFN gamma regulated |
| 5 | GK | 1388485_at | chemokine (C-X-C motif) ligand 14 | *Cxcl14; BRAK* | BG380414 | chemokine signalling |
| 5 | GK | 1369836_at | interferon-induced protein with tetratricopeptide repeats 1 | *Ifit1; Garg16* | NM_020096 | IFN-gamma regulated; inhibits cellular stress response |
| 5 | GK | 1377950_at | interferon inducible GTPase 1 | *Iigp1* | AA955213 | IFN-gamma regulated; resistance to intracellular pathogens |
| 5 | GK | 1369110_x_at | major histocompatability antigen, Class I | *Mhc* | NM_012645 | antigen processing & presentation |
| 5 | GK | 1370463_x_at | major histocompatability antigen, Class I | *Mhc* | U50449 | antigen processing & presentation |
| 5 | GK | 1371119_at | major histocompatability antigen, Class I | *Mhc* | L40364 | antigen processing & presentation |
| 5 | GK | 1371213_at | major histocompatability antigen, Class I | *Mhc* | AJ005023 | antigen processing & presentation |
| 5 | GK | 1379818_at | major histocompatability antigen, Class I | *Mhc* | BF288109 | antigen processing & presentation |
| 5 | GK | 1378265_at | major histocompatability antigen, Class I | *Mhc* | BE103245 | antigen processing & presentation |
| 5 | GK | 1396714_at | phospholipase A2, activating protein | *Plaa; Plap* | BF394289 | endothelial & smooth muscle response to inflammation |
| 4 | GK | 1382678_at | complement component factor h-like 1 | *Cfhl1* | AA859019 | regulation of complement & coagulation cascade |
| 4 | GK | 1390100_s_at | major histocompatability antigen, Class I | *Mhc* | BG371810 | antigen processing & presentation |
| 4 | GK | 1391266_at | major histocompatability antigen, Class I | *Mhc* | AI072881 | antigen processing & presentation |
| 4 | GK | 1370027_a_at | murinoglobulin 1 | *Mug1* | NM_023103 | acute phase response; plasma proteinase inhibitor |
| 3 | GK | 1368338_at | CD52 antigen | *Cd52; B7* | NM_053983 | antigen processing & presentation |
| 3 | GK | 1385635_at | Cd5 molecule-like | *Cd5l* | AI029143 | inhibition of apoptosis |
| 3 | GK | 1383516_at | fibrinogen-like 2 | *Fgl2* | BG663284 | regulation of lymphocyte function |
| 3 | GK | 1375842_at | glycophorin A | *Gypa* | AI177505 | immune response to influenza virus |
| 3 | GK | 1391612_at | interleukin 22 receptor, alpha 2 | *Il22ra2* | BF282190 | IL22 antagonist |
| 3 | GK | 1387992_at | leukocyte immunoglobulin-like receptor, subfamily B (TM and ITIM), member 3-like | *Lilrb3l* | AF169637 | binds to MHC class I molecules |
| 3 | GK | 1390585_at | mannan-binding lectin serine peptidase 1 | *Masp1* | AI169829 | activation of complement cascade |
| 3 | GK | 1377334_at | major histocompatability antigen, Class I | *Mhc* | BG378249 | antigen processing & presentation |
| 3 | GK | 1390562_s_at | major histocompatability antigen, Class I | *Mhc* | BE102350 | antigen processing & presentation |
| 3 | GK | 1369202_at | myxovirus (influenza virus) resistance 2 | *Mx2* | NM_017028 | IFN-alpha/beta regulated; resistance to vesicular stomatitis virus |
| 3 | GK | 1368683_at | oxidized low density lipoprotein (lectin-like) receptor 1 | *Olr1; LOX-1* | NM_133306 | inflammation; cytokine regulated |
| 3 | GK | 1367647_at | serpin peptidase inhibitor, clade A (alpha-1 antiproteinase, antitrypsin), member 1 | *Serpina1* | NM_022519 | inhibition of immune response |
| 3 | GK | 1368048_at | serine (cysteine) peptidase inhibitor, clade A, member 3K | *Serpina3k* | NM_012657 | inhibition of apoptosis |
| 3 | GK | 1367581_a_at | secreted phosphoprotein 1 | *Spp1; OSP; OPN* | AB001382 | cytokine activity; regulation of immune response |
| 3 | GK | 1392731_at | tumor necrosis factor receptor superfamily, member 1b | *Tnfrsf1b* | BM390522 | regulation of TNF-alpha induced apoptosis |
| 3 | GK | 1394803_at | vacuolar protein sorting 52 homolog (S. cerevisiae) | *Vps52; Are1; Sacm2l* | AA957188 | antigen processing & presentation |
| B. Energy Metabolism (44 probe sets, 36 genes) | | | | | | |
| **Age** | **Strain** | **Probe ID** | **Gene Name** | **Symbol** | **Accession** | **Gene Function** |
|  |  |  |  |  |  |  |
| 5 | WKY | 1379435_at | deoxyguanosine kinase | *Dguok* | AI599463 | mitochondrial DNA replication |
| 5 | WKY | 1369663_at | epoxide hydrolase 2, cytoplasmic | *Ephx2* | NM_022936 | eicosanoid breakdown; hypercholesterolemia |
| 5 | WKY | 1379306_at | mitochondrial ribosomal protein L48 | *Mrpl48* | BE111794 | translation of mitochondrial genes |
| 4 | WKY | 1384499_at | butyryl Coenzyme A synthetase 1 | *Bucs1* | BG373566 | fatty acid metabolism |
| 4 | WKY | 1389725_at | transmembrane 7 superfamily member 2 | *Tm7sf2* | BM390364 | cholesterol biosynthesis |
| 3 | WKY | 1369698_at | ATP-binding cassette, sub-family C (CFTR/MRP), member 3 | *Abcc3* | AF072816 | cholesterol efflux pump; cellular lipid removal |
| 3 | WKY | 1367854_at | ATP citrate lyase | *Acly* | NM_016987 | fatty acid & cholesterol biosynthesis |
| 3 | WKY | 1377097_at | cytochrome c oxidase subunit VIb polypeptide 2 | *Cox6b2* | BF545080 | electron transfer chain complex assembly |
| 3 | WKY | *1372318_at | ELOVL family member 6, elongation of long chain fatty acids (yeast) | *Elovl6; Lce2; rELO2* | AI235528 | fatty acid metabolism |
| 3 | WKY | *1388108_at | ELOVL family member 6, elongation of long chain fatty acids (yeast) | *Elovl6; Lce2; rELO2* | BE116152 | fatty acid metabolism |
| 3 | WKY | *1394401_at | ELOVL family member 6, elongation of long chain fatty acids (yeast) | *Elovl6; Lce2; rELO2* | BF396857 | fatty acid metabolism |
| 3 | WKY | 1367707_at | fatty acid synthase | *Fasn* | NM_017332 | fatty acid biosynthesis |
| 3 | WKY | 1382462_at | L-2-hydroxyglutarate dehydrogenase | *L2hgdh* | BF290937 | mitochondrial electron transport |
| 3 | WKY | 1395967_at | low density lipoprotein receptor adaptor protein 1 | *Ldlrap1* | BI275057 | LDL binding & endocytosis |
| 3 | WKY | *1370067_at | malic enzyme 1, NADP(+)-dependent, cytosolic | *Me1* | NM_012600 | carbohydrate/lipid metabolism |
| 3 | WKY | *1370870_at | malic enzyme 1, NADP(+)-dependent, cytosolic | *Me1* | M30596 | carbohydrate/lipid metabolism |
| 3 | WKY | 1393689_at | NADH dehydrogenase (ubiquinone) 1 alpha subcomplex, assembly factor 1 | *Ndufaf1* | BE097322 | NADH:ubiquinone oxidoreductase complex assembly |
| 3 | WKY | *1367668_a_at | stearoyl-CoA desaturase (delta-9-desaturase) | *Scd; Scd2* | NM_031841 | fatty acid biosynthesis |
| 3 | WKY | *1386889_at | stearoyl-CoA desaturase (delta-9-desaturase) | *Scd; Scd2* | BE107760 | fatty acid biosynthesis |
| Table 2. Cont’d. | | | | | | |
| B. Energy Metabolism (44 probe sets, 36 genes) | | | | | | |
| **Age** | **Strain** | **Probe ID** | **Gene Name** | **Symbol** | **Accession** | **Gene Function** |
|  |  |  |  |  |  |  |
| 3 | WKY | 1370355_at | stearoyl-Coenzyme A desaturase 1 | *Scd1* | J02585 | fatty acid biosynthesis |
| 3 | WKY | 1387174_a_at | steroidogenic acute regulatory protein | *Star* | AB006007 | steroid hormone biosynthesis |
| 3 | WKY | 1398261_at | translocase of inner mitochondrial membrane 44 homolog | *Timm44* | NM_017267 | translocation of mitochodrial protein |
| 3 | WKY | *1367611_at | transketolase | *Tkt* | BG667093 | glucose metabolism; pentose phosphate pathway |
| 3 | WKY | *1386859_at | transketolase | *Tkt* | NM_022592 | glucose metabolism; pentose phosphate pathway |
| 3 | WKY | 1387033_at | uncoupling protein 1 (mitochondrial, proton carrier) | *Ucp1* | NM_012682 | uncoupling oxidative phosphorylation from ATP synthesis |
| 5 | GK | *1394490_at | ATP-binding cassette, sub-family A (ABC1), member 1 | *Abca1* | AI502114 | cholesterol efflux pump; cellular lipid removal |
| 5 | GK | *1382431_at | ATP-binding cassette, sub-family A (ABC1), member 1 | *Abca1* | AI103530 | cholesterol efflux pump; cellular lipid removal |
| 5 | GK | 1379846_at | alkaline ceramidase 2 | *Acer2; Asah3l* | AI500753 | hydrolysis of sphingolipid ceramide |
| 5 | GK | *1393421_at | peroxisomal membrane protein 4 | *Pxmp4* | BG664011 | may play a role in ROS metabolism |
| 5 | GK | *1383117_at | peroxisomal membrane protein 4 | *Pxmp4* | AI232414 | may play a role in ROS metabolism |
| 4 | GK | 1368335_at | apolipoprotein A-I | *Apoa1* | NM_012738 | apolipoprotein; HDL |
| 4 | GK | 1367627_at | glycine amidinotransferase (L-arginine:glycine amidinotransferase) | *Gatm* | NM_031031 | creatine synthesis |
| 4 | GK | *1388603_a_at | iron-sulfur cluster assembly 1 homolog | *Isca1; HIscA* | BM384289 | biogenesis of iron-sulfur clusters |
| 3 | GK | 1384247_at | acyl-CoA synthetase long-chain family member 6 | *Acsl6* | AI070365 | fatty acid metabolism |
| 3 | GK | 1387796_at | arachidonate 15-lipoxygenase | *Alox15* | NM_031010 | fatty acid metabolism |
| 3 | GK | 1370359_at | amylase, alpha 1A (salivary) | *Amy1a* | AB057450 | carbohydrate metabolism |
| 3 | GK | 1369502_a_at | amylase 2, pancreatic | *Amy2* | NM_031502 | carbohydrate metabolism |
| 3 | GK | 1368587_at | apolipoprotein C-I | *Apoc1* | NM_012824 | apolipoprotein; VLDL |
| 3 | GK | 1393139_at | apolipoprotein C-II | *Apoc2* | BM385272 | apolipoprotein; LDL |
| 3 | GK | 1387508_at | bile acid Coenzyme A: amino acid N-acyltransferase (glycine N-choloyltransferase) | *Baat* | NM_017300 | lipid metabolism; hydrolysis of CoA esters |
| 3 | GK | 1369111_at | fatty acid binding protein 1, liver | *Fabp1* | NM_012556 | fatty acid transport |
| 3 | GK | 1387139_at | hydroxyacid oxidase 2 (long chain) | *Hao2* | NM_032082 | fatty acid metabolism |
| 3 | GK | *1388613_at | iron-sulfur cluster assembly 1 homolog | *Isca1; HIscA* | BE113034 | biogenesis of iron-sulfur clusters |
| 3 | GK | 1389142_at | sulfide quinone reductase-like (yeast) | *Sqrdl* | AI013361 | mitochondrial organelle biosynthesis |
| C. Transcription/Translation (68 probe sets, 66 genes) | | | | | | |
| **Age** | **Strain** | **Probe ID** | **Gene Name** | **Symbol** | **Accession** | **Gene Function** |
|  |  |  |  |  |  |  |
| 5 | WKY | 1392678_a_at | DNA (cytosine-5-)-methyltransferase 3 alpha | Dnmt3a | AA956455 | DNA de novo methylation; transcriptional repression |
| 5 | WKY | *1379615_at | kelch domain containing 5 | Klhdc5 | AI011501 | repression; BTB/POZ domain |
| 5 | WKY | *1395423_at | kelch domain containing 5 | Klhdc5 | AW920217 | repression; BTB/POZ domain |
| 5 | WKY | 1378541_at | pseudouridylate synthase 7 homolog (S. cerevisiae)-like | Pus7l | BE116562 | tRNA processing |
| 5 | WKY | 1380651_at | ring finger protein (C3H2C3 type) 6 | Rnf6 | BI296352 | DNA, protein binding, ubiquitin-protein ligase activity |
| 5 | WKY | 1382291_at | SRY-box containing gene 9 | Sox9 | AI454332 | apoptosis & cell proliferation |
| 5 | WKY | 1370224_at | signal transducer and activator of transcription 3 | Stat3 | BE113920 | transcription factor |
| 5 | WKY | 1379735_at | TAF7 RNA polymerase II, TATA box binding protein (TBP)-associated factor | Taf7 | AA850836 | transcription factor, a basal regulator |
| 5 | WKY | 1375664_at | trinucleotide repeat containing 6a | Tnrc6a | BI294732 | gene silencing, miRNA processing |
| 5 | WKY | 1369108_at | tumor protein p73-like, TUMOR PROTEIN p63; TP63 | Tp73l; Tp63 | NM_019221 | transcription factor; stress responses and development |
| 5 | WKY | 1379249_at | Wilms tumor 1 associated protein | Wtap | AA900400 | transcriptional & posttranscriptional regulation |
| 5 | WKY | 1371960_at | YTH domain family, member 2 | Ythdf2 | AI230548 | RNA splicing |
| 5 | WKY | 1385892_at | Host cell factor 2 | Hcfc2 | AA900322 | regulation of transcription, RNA polymerase II promoter |
| 4 | WKY | *1391948_at | B-cell CLL/lymphoma 11B (zinc finger protein) | Bcl11b | BM390227 | transcriptional repression; cell proliferation |
| 4 | WKY | 1370138_at | lymphoid enhancer binding factor 1 | Lef1 | NM_130429 | Wnt signaling; DNA binding |
| 4 | WKY | 1391011_at | zinc finger and SCAN domain containing 18 | Zscan18 | AW527691 | DNA binding |
| 4 | WKY | 1384794_at | glucocorticoid modulatory element binding protein 1 | Gmeb1 | BE106136 | transcription regulation; glucocorticoid regulation |
| 3 | WKY | 1385202_at | additional sex combs like 3 (Drosophila) | Asxl3 | BE118820 | transcriptional repression; development |
| 3 | WKY | *1384944_at | B-cell leukemia/lymphoma 11B | Bcl11b | BE116855 | transcriptional regulation;differntiation & cell survival |
| 3 | WKY | 1374283_at | bromodomain PHD finger transcription factor | Bptf; Falz | BF419505 | chromatin remodeling |
| 3 | WKY | 1382809_at | cold inducible RNA binding protein | Cirbp | AW921084 | RNA binding; suppression of cell proliferation |
| 3 | WKY | 1385871_at | DEAH (Asp-Glu-Ala-His) box polypeptide 36 | Dhx36 | AA859982 | RNA helicase |
| 3 | WKY | 1392064_at | distal-less homeobox 1 | Dlx1 | BF400590 | interacts with Smad4 through its homeodomain |
| 3 | WKY | 1377797_at | EMG1 nucleolar protein homolog (S. cerevisiae) | Emg1 | AI071470 | rRNA processing and ribosome biogenesis |
| 3 | WKY | 1387704_at | estrogen receptor 1 | Esr1 | NM_012689 | hormone activated transcription factor |
| 3 | WKY | 1382848_at | forkhead box A1 | Foxa1; Hnf3a | BM383972 | transcriptional activator |
| 3 | WKY | 1385464_at | forkhead box Q1 | Foxq1; Hfh1 | AI070944 | transcriptional activator |
| 3 | WKY | 1382163_at | GATA binding protein 3 | Gata3 | BE118901 | transcriptional activator |
| 3 | WKY | 1387827_x_at | histone cluster 1, H2bl | Hist1h2bl | NM_022647 | nucleosome organization |
| 3 | WKY | 1380835_at | interferon stimulated exonuclease gene 20-like 2 | Isg20l2 | BF389476 | rRNA processing & ribosome biogenesis |
| 3 | WKY | 1390813_at | Musashi homolog 2 (Drosophila) | Msi2 | AI236624 | RNA binding; posttranscriptional regulation |
| 3 | WKY | 1375891_at | ring finger protein 1 | Ring1 | BG379665 | transcriptional repressor |
| 3 | WKY | 1376197_at | transcription factor 7, T-cell specific | Tcf7 | AW251860 | transcriptional activator; Wnt signaling |
| 3 | WKY | 1382524_at | zinc finger and BTB domain containing 20 | Zbtb20 | AA850361 | transcription factor; closely related to BCL-6 |
| 3 | WKY | 1380804_at | zinc finger protein 185 | Zfp185 | BM391896 | LIM-domain zinc finger protein; cellular proliferation and/or differentiation |
| 3 | WKY | 1393259_at | avian musculoaponeurotic fibrosarcoma (v-maf) AS42 oncogene homolog | Maf | AI044342 | transcription regulation; amino acid metabolism |
| 3 | WKY | 1385120_at | premature ovarian failure 1B | Pof1b | BG375052 | protein binding; signal transduction |
| Table 2. Cont’d. | | | | | | |
| C. Transcription/Translation (68 probe sets, 66 genes) | | | | | | |
| **Age** | **Strain** | **Probe ID** | **Gene Name** | **Symbol** | **Accession** | **Gene Function** |
|  |  |  |  |  |  |  |
| 5 | GK | 1395030_at | kruppel-like factor 9 | Klf9; Bteb1 | BM390366 | transcription factor |
| 5 | GK | 1391600_at | MAX gene associated | Mga | BE118049 | regulates expression of both Max-network & T-box family genes |
| 5 | GK | 1380094_a_at | Zinc finger protein 212 | Zfp212 | AI178375 | DNA binding |
| 5 | GK | 1381414_at | zinc finger protein 26 | Zfp26 | AA964872 | transcription factor |
| 4 | GK | 1382648_at | glutamyl-prolyl-tRNA synthetase | Eprs | AI235566 | tRNA processing |
| 4 | GK | 1382401_at | non-coding transcript 1 | Nctc1 | AI172597 | coding for a non-translated RNA |
| 4 | GK | 1369067_at | nuclear receptor subfamily 4, group A, member 3 | Nr4a3; NOR-2 | NM_031628 | ligand-dependent nuclear receptor |
| 4 | GK | 1386621_at | nuclear fragile X mental retardation protein interacting protein 2 | Nufip2 | AW142419 | RNA binding |
| 4 | GK | 1390979_at | zinc finger protein 14 | Zfp14 | AI409884 | transcriptional repression |
| 3 | GK | 1376748_at | aristaless related homeobox | Arx | AI406684 | Homeodomain transcription factor |
| 3 | GK | 1382467_at | brain expressed X-linked 2 | Bex2 | C07140 | part of a DNA-binding complex that recognized the E-box element |
| 3 | GK | 1381795_at | castor homolog 1, zinc finger (Drosophila) | Casz1 | AA963295 | transcription factor; mesenchyme differentiation |
| 3 | GK | 1375655_at | cAMP responsive element binding protein 1 | Creb1 | BE107208 | binding to the cAMP responsive element (CRE) |
| 3 | GK | 1391635_at | CTD (carboxy-terminal, RNA polymerase II, polypeptide A) small phosphatase-like | Ctdspl | AI044500 | phosphoprotein phosphatase activity; phosphoric monoester hydrolase activity |
| 3 | GK | 1368321_at | early growth response 1 | Egr1 | NM_012551 | transcriptional regulation; differentiation |
| 3 | GK | 1392969_at | hepatic nuclear factor 4, alpha | Hnf4a; Tcf4 | BI282309 | transcription factor which binds DNA as a homodimer |
| 3 | GK | 1396743_at | heterogeneous nuclear ribonucleoprotein D | Hnrnpd | BF391580 | pre-mRNA processing; regulation of mRNA stability |
| 3 | GK | 1373684_at | kelch-like 31 (Drosophila) | Klhl31 | AA818643 | protein binding |
| 3 | GK | 1398655_at | myogenic differentiation 1 | Myod1 | AA955902 | mesenchymal stem cell muscle differention |
| 3 | GK | 1375040_at | nuclear factor, erythroid derived 2 | Nfe2 | AW252129 | regulation of transcription from RNA pol 2 promoter |
| 3 | GK | 1383577_at | RAB, member of RAS oncogene family-like 3 | Rabl3 | AA859277 | ATP & GTP binding; transcription factor binding |
| 3 | GK | 1393338_at | scleraxis | Scx | AW528719 | Dimerizes with bHLH protein; E-box binding |
| 3 | GK | 1377960_at | splicing factor 3a, subunit 1 | Sf3a1 | BF407455 | spliceosome pre-mRNA binding |
| 3 | GK | 1398581_at | sine oculis-binding protein homolog (Drosophila) | Sobp | AA956307 | Transcription factor, positive regulation of IGFbp5 |
| 3 | GK | 1378857_at | transcription elongation factor A (SII)-like 5 | Tceal5 | AI716196 | transcriptional regulation |
| 3 | GK | 1382368_at | TROVE domain family, member 2 | Trove2 | AA943075 | transcription from RNA polymerase III promoter |
| 3 | GK | 1382171_at | TSC22 domain family, member 2 | Tsc22d2 | AI454310 | ligand dependent nuclear receptor activity |
| 3 | GK | 1384033_at | vestigial like 2 (Drosophila) | Vgll2 | BF284307 | transcription coactivator; development |
| 3 | GK | 1390554_at | zinc finger protein 385B | Zfp385b | AA818377 | nucleic acid binding; zinc ion binding |
| 3 | GK | 1382205_at | zinc finger protein of the cerebellum 1 | Zic1 | AW527509 | transactivation of apolipoprotein E gene |
| 3 | GK | 1392392_at | nuclear receptor co-repressor 1 | Ncor1 | AI012510 | transcriptional repression; histone deacetylation |
| D. Signaling (64 probe sets, 62 genes) | | | | | | |
| **Age** | **Strain** | **Probe ID** | **Gene Name** | **Symbol** | **Accession** | **Gene Function** |
|  |  |  |  |  |  |  |
| 5 | WKY | 1370114_a_at | phosphoinositide-3-kinase, regulatory subunit 1 (alpha) | Pik3r1; PI3KA | D64048 | insulin signaling |
| 5 | WKY | 1395020_at | pleckstrin homology domain containing, family H (with MyTH4 domain) member 1 | Plekhh1 | BI275435 | domain present in many signaling proteins |
| 5 | WKY | 1381386_at | ring finger protein 10 | Rnf10 | AI407297 | signal transduction |
| 4 | WKY | 1387938_at | brain and acute leukemia, cytoplasmic | Baalc | AB073318 | synapse signal transduction |
| 4 | WKY | 1384836_at | calmodulin-like 3 | Calml3 | AI030853 | signal molecule; differentiation |
| 4 | WKY | 1388199_at | epithelial cell adhesion molecule | Epcam; Tacstd1 | BG376410 | calcium signal transducer |
| 4 | WKY | 1368872_a_at | homer homolog 2 (Drosophila) | Homer2; Vesl-2 | NM_053309 | regulation of intracellular signaling |
| 4 | WKY | 1368145_at | Purkinje cell protein 4 | Pcp4 | NM_013002 | inhibition of calmodulin signaling |
| 4 | WKY | *1372423_at | PERP, TP53 apoptosis effector | Perp | BI286396 | p53-dependent apoptosis signaling |
| 4 | WKY | 1389118_s_at | S100 calcium binding protein A14 | S100a14 | BM387197 | Ca binding protein; signal transduction |
| 4 | WKY | 1391509_at | tumor-associated calcium signal transducer 2 | Tacstd2 | AA891826 | tumor-associated calcium signal transducer |
| 4 | WKY | 1396455_at | uroplakin 1A | Upk1a | BI302778 | cellular signal transduction; regulation of membrane physiology |
| 4 | WKY | 1383875_at | uroplakin 1B | Upk1b | BE096378 | cellular signal transduction; regulation of membrane physiology |
| 3 | WKY | 1369773_at | bone morphogenetic protein 3 | Bmp3 | NM_017105 | signal molecule; development & differentiation |
| 3 | WKY | 1370216_at | discoidin domain receptor tyrosine kinase 1 | Ddr1; Cak; PTK3D | BE112590 | extracellular matrix-associated signaling |
| 3 | WKY | 1384031_at | EF-hand domain (C-terminal) containing 1 | Efhc1 | AW532656 | Ca cascade signaling; apoptosis |
| 3 | WKY | 1369594_at | ephrin A5 | Efna5; Lerk7 | NM_053903 | EPH-related receptor tyrosine kinase ligand |
| 3 | WKY | 1374699_at | family with sequence similarity 84, member A | Fam84a | BI286340 | cell morphology and migration signaling |
| 3 | WKY | 1395324_at | FCH and double SH3 domains 2 | Fchsd2 | BM385905 | Membrane-associated guanylate kinase inter acting protein |
| 3 | WKY | 1375961_at | frizzled-related protein | Frzb | AI072892 | secreted glycoprotein; inhibition of Wnt signaling |
| 3 | WKY | 1387063_at | inositol hexakisphosphate kinase 2 | Ip6k2 | NM_021660 | insulin secretion signaling; stress regulated growth suppression & apoptosis |
| 3 | WKY | 1388157_at | myristoylated alanine rich protein kinase C substrate | Marcks | BE111706 | protein kinase C signaling |
| 3 | WKY | 1386969_at | neuritin 1 | Nrn1 | NM_053346 | angiogenic-endothelial signaling |
| 3 | WKY | 1387710_at | oxytocin receptor | Oxtr | L81169 | G-protein coupled receptor IP3 signaling |
| 3 | WKY | 1380433_at | progestin and adipoQ receptor family member VII | Paqr7 | AI229240 | integral membrane protein; receptor activity |
| 3 | WKY | *1389177_at | PERP, TP53 apoptosis effector | Perp | AI598971 | p53-dependent apoptosis signaling |
| 3 | WKY | 1387459_at | protein kinase (cAMP-dependent, catalytic) inhibitor beta | Pkib | NM_012627 | inhibition of cAMP signaling pathway |
| 3 | WKY | 1393657_at | prolylcarboxypeptidase (angiotensinase C) | Prcp | BI285860 | renin-angiotensin and kallikrein-kinin signaling regulation |
| 3 | WKY | 1370989_at | ret proto-oncogene | Ret | AI639318 | RET tyrosine kinase activity |
| 3 | WKY | 1376062_at | syndecan 1 | Sdc1 | BG375315 | extracellular matrix-associated signaling molecule; lipoprotein lipase secretion |
| 3 | WKY | 1393926_at | signal transducing adaptor family member 1 | Stap1 | AW920039 | BCR signaling; interacts with STAT5 and tyrosine kinase Tec |
| Table 2. Cont’d. | | | | | | |
| D. Signaling (64 probe sets, 62 genes) | | | | | | |
| **Age** | **Strain** | **Probe ID** | **Gene Name** | **Symbol** | **Accession** | **Gene Function** |
|  |  |  |  |  |  |  |
| 3 | WKY | 1388396_at | serine/threonine kinase 25 (STE20 homolog, yeast) | Stk25 | BI275932 | stress regulated cellular signaling |
| 5 | GK | 1369716_s_at | lectin, galactose binding, soluble 5 | Lgals5 | NM_012976 | NF-kappaB signaling regulation |
| 5 | GK | 1392767_at | TBC1 domain family, member 20 | Tbc1d20 | AI502349 | GTPase activator activity |
| 5 | GK | 1392382_at | transforming growth factor, beta 2 | Tgfb2 | BE117736 | signal molecule; growth, development & apoptosis |
| 4 | GK | 1383894_at | folliculin interacting protein 1 | Fnip1 | BI289486 | AMPK and mTOR signaling |
| 4 | GK | 1387027_a_at | lectin, galactoside-binding, soluble, 9 | Lgals9 | U72741 | NF-kappaB signaling regulation |
| 4 | GK | 1369550_at | myostatin | Mstn; Gdf8 | NM_019151 | cytokine signaling; negative regulator of skeletal muscle & adipose growth |
| 4 | GK | *1385799_at | neurexophilin 1 | Nxph1 | AW531533 | cAMP signaling cascade; development |
| 4 | GK | 1394462_at | obscurin, cytoskeletal calmodulin and titin-interacting RhoGEF | Obscn | AI716969 | G protein-coupled sacromeric signaling |
| 4 | GK | 1385974_at | par-6 partitioning defective 6 homolog gamma (C. elegans) | Pard6g | BM384723 | interacts directly with GTP-bound CRIB-like motif (RAC or CDC42) |
| 4 | GK | 1379433_at | phosphoinositide-3-kinase, class 2, alpha polypeptide | Pik3c2a | AI059449 | insulin signaling |
| 4 | GK | 1373582_at | CAP-GLY domain containing linker protein 3 | Clip3 | BF418394 | Interacts with Akt & Regulates Akt Cellular Compartmentalization |
| 3 | GK | 1369326_at | A kinase (PRKA) anchor protein 6 | Akap6 | NM_022618 | localization of PKA to nulcear membrane |
| 3 | GK | 1372641_at | alpha-kinase 3 | Alpk3 | AW434261 | cardiomyocyte differentiation |
| 3 | GK | 1374207_at | angiopoietin 2 | Angpt2 | BI275292 | angiogenesis |
| 3 | GK | 1388741_at | cardiomyopathy associated 5 | Cmya5 | AW533234 | PKA anchor protein; PKA-CREB signaling |
| 3 | GK | 1393783_at | contactin associated protein-like 2 | Cntnap2 | AI706662 | cell adhesion signal transduction |
| 3 | GK | 1368064_a_at | dopa decarboxylase (aromatic L-amino acid decarboxylase) | Ddc | U31884 | signal molecule conversion |
| 3 | GK | 1382778_at | dual specificity phosphatase 6 | Dusp6 | AI231350 | protein tyrosine phosphatase; inactivation of MAPK activity |
| 3 | GK | 1387146_a_at | endothelin receptor type B | Ednrb | X57764 | G protein-coupled receptor IP3 signaling |
| 3 | GK | 1387082_at | fetuin B | Fetub | NM_053348 | insulin signaling regulation |
| 3 | GK | 1368459_at | growth differentiation factor 10 | Gdf10 | NM_024375 | cytokine signaling |
| 3 | GK | 1387906_a_at | GNAS complex locus | Gnas; Gnas1; Gnpas | AF107845 | participates in adenylate cyclase activating pathway |
| 3 | GK | 1391512_at | myotubularin related protein 1 | Mtmr1 | AI029734 | dephosphorylates phosphatidylinositol 3-phosphate and Ins(1,3)P2 |
| 3 | GK | 1371801_at | myozenin 2 | Myoz2 | AI104354 | modulation of calcineurin signaling |
| 3 | GK | *1386697_at | neurexophilin 1 | Nxph1 | BF567897 | cAMP signaling cascade |
| 3 | GK | 1385381_at | phosphodiesterase 10A | Pde10a | AA996491 | regulation of cAMP- and cGMP-mediated intracellular signaling |
| 3 | GK | 1387065_at | phospholipase C, delta 4 | Plcd4 | NM_080688 | PKC activation; IP3 and Erk signaling |
| 3 | GK | 1373108_at | protein phosphatase 1, regulatory (inhibitor) subunit 3C | Ppp1r3c | BM390827 | dephosphorylation of signaling components; glycogen synthesis |
| 3 | GK | 1383322_at | RAS-like family 11 member B | Rasl11b | BG375198 | small GTPase mediated signal transduction; cell growth inhibition |
| 3 | GK | 1388072_at | SH3-binding domain kinase 1 | Sbk1 | AB010154 | Serine/threonine protein kinase |
| 3 | GK | 1379382_at | sema, immunoglobulin (Ig), and short basic domains, secreted, (semaphorin) 3E | Sema3e | AI144865 | cell-cell interaction signaling |
| 3 | GK | 1369580_at | taste receptor, type 2, member 118 | Tas2r118 | NM_023994 | G-protein coupled receptor signaling |
| E. Small Molecule Metabolism (28 probe sets, 26 genes) | | | | | | |
| **Age** | **Strain** | **Probe ID** | **Gene Name** | **Symbol** | **Accession** | **Gene Function** |
|  |  |  |  |  |  |  |
| 5 | WKY | 1370902_at | aldo-keto reductase family 1, member B8 | Akr1b8 | AI233740 | detoxification of aldehydes and ketones |
| 5 | WKY | 1374006_at | kynurenine aminotransferase III | Kat3; Ccbl2 | BI295878 | transaminase activity |
| 4 | WKY | 1392539_at | biliverdin reductase B (flavin reductase (NADPH)) | Blvrb | AI009013 | antioxidant enzyme; heme metabolism |
| 4 | WKY | 1372297_at | glutathione S-transferase alpha 4 | Gsta4 | AI234527 | glutathione conjugation of hydrophobic electrophiles |
| 4 | WKY | 1379390_at | ST6 (alpha-N-acetyl-neuraminyl-2,3-beta-galactosyl-1,3)-N-acetylgalactosaminide alpha-2,6-sialyltransferase 2 | St6galnac2 | AA891414 | transferation of sialic acid to glycoconjugates |
| 3 | WKY | 1393945_at | fucosyltransferase 9 | Fut9 | AI145487 | Lewis antigen biosynthesis |
| 3 | WKY | 1369097_s_at | guanylate cyclase 1, soluble, beta 3 | Gucy1b3; SGC | NM_012769 | conversion of GTP to cyclic GMP |
| 3 | WKY | 1389425_at | 5',3'-nucleotidase, cytosolic | Nt5c | AI408674 | dephosphorylation of nucleotide monophosphate |
| 3 | WKY | 1389369_at | superoxide dismutase 2, mitochondrial | Sod2 | BF289017 | detoxification of oxidative radicals |
| 5 | GK | 1368826_at | catechol-O-methyltransferase | Comt | NM_012531 | O-methylation of catecholamine and catechol |
| 5 | GK | 1368304_at | flavin containing monooxygenase 3 | Fmo3 | NM_053433 | methimazole S-oxidation and NADPH oxidation |
| 4 | GK | 1368569_at | aldo-keto reductase family 1, member B7 | Akr1b7; Avdp; MVDP | NM_053781 | reduction of aldehydes; inhibition of adipose maturation |
| 4 | GK | 1391417_at | betaine-homocysteine methyltransferase 2 | Bhmt2 | AA925145 | methylation of homocysteine |
| 4 | GK | *1368738_at | cytochrome P450, subfamily 11B, polypeptide 1 | Cyp11b1 | D11354 | steroid hormone metabolism |
| 4 | GK | *1368739_s_at | cytochrome P450, subfamily 11B, polypeptide 1 | Cyp11b1 | D11354 | steroid hormone metabolism |
| 4 | GK | *1387305_s_at | cytochrome P450, subfamily 11B, polypeptide 1 | Cyp11b1 | NM_012539 | steroid hormone metabolism |
| 4 | GK | 1369264_at | cytochrome P450, subfamily 21A, polypeptide 1 | Cyp21a1; 21-OH | NM_057101 | steroid hormone synthesis |
| 4 | GK | 1368578_at | hydroxy-delta-5-steroid dehydrogenase, 3 beta- and steroid delta-isomerase 6 | Hsd3b6 | NM_017265 | steroid hormone synthesis |
| 3 | GK | 1395190_at | aldo-keto reductase family 1, member B10 (aldose reductase) | Akr1b10 | AI408099 | reduction of aliphatic & aromatic aldehydes; inhibition of adipose differentiation |
| 3 | GK | 1398612_at | aldo-keto reductase family 1, member C12 | Akr1c12 | BM383010 | reduction of progesterone |
| 3 | GK | 1397855_at | cysteine sulfinic acid decarboxylase | Csad | BE096287 | taurine biosynthesis; response to oxidative stress |
| 3 | GK | 1368990_at | cytochrome P450, family 1, subfamily b, polypeptide 1 | Cyp1b1 | NM_012940 | polycyclic aromatic hydrocarbon metabolism |
| 3 | GK | 1387118_at | cytochrome P450, family 3, subfamily a, polypeptide 23/polypeptide 1 | Cyp3a23/3a1 | NM_013105 | xenobiotics oxidation; steroids metabolism |
| 3 | GK | 1394844_s_at | cytochrome P450, family 4, subfamily a, polypeptide 2 | Cyp4a2 | AA893326 | hydroxylation of medium chain fatty acid |
| 3 | GK | 1368467_at | cytochrome P450, family 4, subfamily f, polypeptide 1 | Cyp4f1 | NM_019623 | inactivation of leukotriene B4 |
| 3 | GK | 1375909_at | glutathione S-transferase mu 4 | Gstm4 | BI285792 | oxidative stress; xenobiotic degradation |
| 3 | GK | 1371054_at | phenylethanolamine-N-methyltransferase | Pnmt | BF408734 | epinephrine and phosphatidylcholine synthesis |
| 3 | GK | 1368397_at | UDP glucuronosyltransferase 2 family, polypeptide B36 | Ugt2b36 | NM_031980 | glucuronidation of monoterpenoid alcohol; xenobiotic detoxification |
| Table 2. Cont’d. | | | | | | |
| F. Transport (27 probe sets, 25 genes) | | | | | | |
| **Age** | **Strain** | **Probe ID** | **Gene Name** | **Symbol** | **Accession** | **Gene Function** |
|  |  |  |  |  |  |  |
| 5 | WKY | 1384219_at | SNF8, ESCRT-II complex subunit, homolog | Snf8; D11moh34 | BF522747 | protein transport |
| 5 | WKY | 1370115_at | solute carrier family 7, (neutral amino acid transporter, y+ system) member 10 | *Slc7a10; Asc-1* | NM_053726 | small neutral amino acid transport |
| 4 | WKY | 1387100_at | aquaporin 3 | *Aqp3* | NM_031703 | urea & glycerol transport |
| 4 | WKY | 1374015_at | potassium voltage-gated channel, Isk-related family, member 1-like | *Kcne1l; Kcne5* | BF281697 | potassium ion channel |
| 4 | WKY | *1370781_a_at | Kv channel-interacting protein 1 | *Kcnip1* | AY082657 | potassium ion channel regulation |
| 4 | WKY | 1386679_at | solute carrier family 14 (urea transporter), member 1 | *Slc14a1* | BF562962 | urea transport |
| 4 | WKY | 1369680_at | solute carrier family 2 (facilitated glucose transporter), member 13 | *Slc2a13; Hmit* | NM_133611 | myo-inositol transport |
| 4 | WKY | 1390835_at | solute carrier family 47, member 1 | *Slc47a1* | AI013568 | xenobiotic transport |
| 3 | WKY | 1398623_at | cholinergic receptor, nicotinic, beta polypeptide 4 | *Chrnb4* | AI454607 | ion channel regulation |
| 3 | WKY | 1374630_at | chloride intracellular channel 3 | *Clic3* | AI234249 | chloride ion transport |
| 3 | WKY | *1394097_at | Kv channel-interacting protein 1 | *Kcnip1* | AA849706 | potassium ion channel regulation |
| 3 | WKY | 1369064_a_at | sodium channel, voltage gated, type VIII, alpha subunit | *Scn8a* | NM_019266 | voltage-dependent sodium ion transport |
| 3 | WKY | 1368440_at | solute carrier family 3, member 1 | *Slc3a1* | NM_017216 | neutral and basic amino acid transport |
| 3 | WKY | 1394805_at | exportin 6 | *Xpo6* | BF402425 | transport across nuclear pore |
| 5 | GK | *1386770_x_at | potassium voltage-gated channel, Isk-related subfamily, gene 2 | *Kcne2; Mirp1* | AW920063 | potassium ion transport regulation |
| 5 | GK | 1370176_at | trafficking protein, kinesin binding 2 | *Trak2; GRIF-1* | BG378620 | intracellular transport |
| 4 | GK | *1369322_at | potassium voltage-gated channel, Isk-related subfamily, gene 2 | *Kcne2; Mirp1* | NM_133603 | potassium ion transport regulation |
| 3 | GK | 1372190_at | aquaporin 4 | *Aqp4; Miwc* | AI235942 | water channel protein |
| 3 | GK | 1388451_at | calcium channel, voltage-dependent, alpha2/delta subunit 1 | *Cacna2d1* | AA817802 | calcium ion transport |
| 3 | GK | 1387025_at | dynein cytoplasmic 1 intermediate chain 1 | *Dync1i1* | NM_019234 | subcellular targeting and organelle transport |
| 3 | GK | 1387768_at | myoglobin | *Mb* | NM_021588 | oxygen storage and transport |
| 3 | GK | 1393821_at | Na+/K+ transporting ATPase interacting 3 | *Nkain3* | BF416086 | sodium/potassium ion transport regulation |
| 3 | GK | 1370609_a_at | solute carrier family 16, member 7 (monocarboxylic acid transporter 2) | *Slc16a7; Mct2* | X97445 | monocarboxylate & ketone body transport |
| 3 | GK | 1387656_at | solute carrier family 4 (anion exchanger), member 1 | *Slc4a1* | BE113640 | carbon dioxide transport |
| 3 | GK | 1387808_at | solute carrier family 7 (cationic amino acid transporter, y+ system), member 7 | *Slc7a7* | AF200684 | cationic & large neutral amino acid transport |
| 3 | GK | 1375877_at | synaptotagmin IV | *Syt4* | AI454612 | vesicle transport; Ca-dependent exocytosis |
| 3 | GK | 1377448_at | transmembrane protein 38a | *Tmem38a* | BG374556 | potassium counter-ion channel for Ca release |
| G. Protein Processing (24 probe sets, 22 genes) | | | | | | |
| **Age** | **Strain** | **Probe ID** | **Gene Name** | **Symbol** | **Accession** | **Gene Function** |
|  |  |  |  |  |  |  |
| 5 | WKY | 1383422_at | BEN domain containing 5 | *Bend5* | AW252401 | protein processing |
| 5 | WKY | 1379747_at | protease, serine, 35 | *Prss35* | AA866443 | protein catabolism |
| 5 | WKY | *1385395_at | syntaxin 17 | *Stx17* | AI549268 | membrane trafficking; vessicular transport |
| 5 | WKY | 1369977_at | ubiquitin carboxyl-terminal esterase L1 (ubiquitin thiolesterase) | *Uchl1* | NM_017237 | negative regulator of protein catabolism |
| 4 | WKY | 1369866_at | chymosin | *Cym* | NM_020091 | protein catabolism; aspartate protease |
| 4 | WKY | *1370597_at | syntaxin 17 | *Stx17* | AF115435 | membrane trafficking; vessicular transport |
| 3 | WKY | *1382999_at | ADP-ribosyltransferase 2b | *Art2b; RT6.2* | AI411747 | ADP-ribosylation |
| 3 | WKY | *1370629_at | ADP-ribosyltransferase 2b | *Art2b; RT6.2* | M85193 | ADP-ribosylation |
| 3 | WKY | 1372345_at | coatomer protein complex, subunit gamma 2, antisense 2 | *Copg2as2; Lb9; Mit1* | AA894210 | vesicle-mediated protein transport |
| 3 | WKY | 1390050_at | similar to Golgi phosphoprotein 2 (Golgi membrane protein GP73) | *LOC682869* | BI288898 | transport through Golgi |
| 3 | WKY | 1391222_at | Nedd4 binding protein 1 | *N4bp1* | AA965076 | protein catabolism |
| 3 | WKY | 1376129_at | similar to vacuolar protein sorting 13C protein | *RGD1560364* | BE108174 | vesicle-mediated protein transport |
| 3 | WKY | 1371677_at | serine peptidase inhibitor, Kazal type 8 | *Spink8* | BE113200 | negative regulator of protein catabolism |
| 3 | WKY | 1382984_at | torsin family 1, member B | *Tor1b* | AI073272 | chaparone; protein folding |
| 5 | GK | 1383510_at | ubiquitin protein ligase E3A | *Ube3a* | AI230360 | protein catabolism |
| 4 | GK | 1374330_at | Kell blood group, metallo-endopeptidase | *Kel* | AI179459 | zinc endopeptidase activity; endothelin conversion |
| 3 | GK | 1393842_at | coiled-coil domain containing 77 | *Ccdc77* | BF391556 | glycoprotein synthesis |
| 3 | GK | 1392598_at | DnaJ (Hsp40) homolog, subfamily B, member 6 | *Dnajb6* | AA899326 | chaperone; protein folding & complex assembly |
| 3 | GK | 1381990_at | UDP-N-acetyl-alpha-D-galactosamine:polypeptide N-acetylgalactosaminyltransferase 1 | *Galnt1* | AA963979 | glycoprotein synthesis |
| 3 | GK | 1369410_at | golgi SNAP receptor complex member 1 | *Gosr1* | NM_053584 | ER-Golgi protein trafficking |
| 3 | GK | 1371442_at | hypoxia up-regulated 1 | *Hyou1; Cab140; Orp150* | BI282904 | chaparone; HSP |
| 3 | GK | 1371124_a_at | kininogen 2 | *Kng2; Kngk* | M11884 | negative regulator of protein catabolism |
| 3 | GK | 1391018_at | myosin VC | *Myo5c* | BF420807 | membrane trafficking |
| 3 | GK | 1374828_at | protein disulfide isomerase family A, member 5 | *Pdia5* | AI045590 | stress regulated protein folding |
| H. Cell Cycle Control (10 probe sets, 10 genes) | | | | | | |
| **Age** | **Strain** | **Probe ID** | **Gene Name** | **Symbol** | **Accession** | **Gene Function** |
|  |  |  |  |  |  |  |
| 5 | WKY | 1387391_at | cyclin-dependent kinase inhibitor 1A (p21, Cip1) | *Cdkn1a* | U24174 | negative regulation of cell cycle |
| 5 | WKY | 1390722_at | nuclear casein kinase and cyclin-dependent kinase substrate 1 | *Nucks1* | AW531272 | mitosis |
| 3 | WKY | 1381503_at | DNA-damage inducible protein 2 | *Ddi2* | AI073168 | DNA repair |
| 3 | WKY | 1376875_at | telomeric repeat binding factor 2 | *Terf2* | AI454691 | regulation of telomere maintenance |
| 4 | GK | 1398049_at | nitrilase 1 | *Nit1* | BF405475 | tumor suppressor signaling |
| 3 | GK | 1383895_at | dynein light chain roadblock-type 2 | *Dynlrb2* | AI144946 | regulation of cell division |
| Table 2. Cont’d. | | | | | | |
| H. Cell Cycle Control (10 probe sets, 10 genes) | | | | | | |
| **Age** | **Strain** | **Probe ID** | **Gene Name** | **Symbol** | **Accession** | **Gene Function** |
|  |  |  |  |  |  |  |
| 3 | GK | 1389996_at | NIMA (never in mitosis gene a)-related expressed kinase 1 | *Nek1* | AI406369 | checkpoint control of cell cycle |
| 3 | GK | 1370854_at | nexilin | *Nexn* | AA799423 | regulation of cytoskeleton organization/ biogenesis |
| 3 | GK | 1388578_at | TatD DNase domain containing 3 | *Tatdn3* | BI288621 | deoxyribonuclease |
| 3 | GK | 1391552_at | translin | *Tsn* | BM391661 | DNA replication |
| I. Miscellaneous (76 probe sets, 67 genes) | | | | | | |
| **Age** | **Strain** | **Probe ID** | **Gene Name** | **Symbol** | **Accession** | **Gene Function** |
|  |  |  |  |  |  |  |
| 5 | WKY | 1377778_at | ischemia related factor vof-16 | *Vof16* | BE107282 | brain ischemia regulated |
| 4 | WKY | 1386947_at | cadherin 1 | *Cdh1* | NM_031334 | cell-cell adhesion |
| 4 | WKY | *1386128_at | C-type lectin domain family 3, member b | *Clec3b; Tna* | BF524010 | exocytosis |
| 4 | WKY | 1372153_at | keratin 15 | *Krt15* | BM385502 | intermediate filament protein; cell structure |
| 4 | WKY | 1371530_at | keratin 8 | *Krt8; CYKER* | BF281337 | intermediate filament protein; muscle contraction |
| 4 | WKY | 1371248_at | small proline-rich protein 1A-like | *Sprr1al* | BI286387 | crosslinking envelope formation |
| 4 | WKY | 1375098_at | seminal vesicle secretory protein 7 | *Svs7* | BI280571 | secreted protein in Ly-6 superfamily |
| 4 | WKY | 1367592_at | troponin T type 2 (cardiac) | *Tnnt2* | NM_012676 | motor activity |
| 4 | WKY | 1384695_at | uroplakin 3A | *Upk3a* | BM387108 | cytoskeleton |
| 3 | WKY | 1386869_at | actin, gamma 2, smooth muscle, enteric | *Actg2* | NM_012893 | actin cytoskeleton organization |
| 3 | WKY | *1367555_at | albumin | *Alb* | NM_134326 | nitric oxide signaling; serum lipid carrier protein; osmotic pressure |
| 3 | WKY | *1367556_s_at | albumin | *Alb* | NM_134326 | nitric oxide signaling; serum lipid carrier protein; osmotic pressure |
| 3 | WKY | 1393469_at | cell adhesion molecule with homology to L1CAM | *Chl1* | BE102340 | cell-cell adhesion |
| 3 | WKY | 1385058_at | claudin 8 | *Cldn8* | BI292090 | intercellular tight junction organization |
| 3 | WKY | *1377452_at | C-type lectin domain family 3, member b | *Clec3b; Tna* | AW251280 | exocytosis |
| 3 | WKY | 1388101_at | dihydropyrimidinase-like 3 | *Dpysl3* | AF389425 | extracellular matrix |
| 3 | WKY | 1393910_at | family with sequence similarity 13, member A1 | *Fam13a1* | BF563961 | transmembrane protein; unknown function |
| 3 | WKY | 1377011_at | furry homolog (Drosophila) | *Fry* | BF415061 | regulation of actin cytoskeleton |
| 3 | WKY | 1391575_at | hyaluronan and proteoglycan link protein 4 | *Hapln4* | BG380566 | extracellular matrix |
| 3 | WKY | 1388155_at | keratin 18 | *Krt18* | BI286012 | intermediate filament protein; cell structure |
| 3 | WKY | 1388433_at | keratin 19 | *Krt19* | BI279605 | intermediate filament protein |
| 3 | WKY | 1370863_at | keratin 5 | *Krt5* | BF283692 | intermediate filament protein |
| 3 | WKY | 1385216_at | LIM domain containing 2 | *Limd2* | BI290864 | Zn binding protein |
| 3 | WKY | 1390235_at | MAP6 domain containing 1 | *Map6d1* | BF410589 | microtubule binding & stabilization |
| 3 | WKY | 1384818_at | myosin light chain kinase | *Mylk* | BM383873 | motor activity |
| 3 | WKY | 1386941_at | plectin 1 | *Plec1* | NM_022401 | regulation of cytoskeleton organization & dynamics |
| 3 | WKY | 1375996_at | septin 1 | *Sept1* | AA894262 | GTPase activity; regulation of cytokinesis |
| 3 | WKY | 1385225_at | sphingomyelin phosphodiesterase 3, neutral | *Smpd3* | BF386619 | hydrolysis of sphingomyelin to form ceramide and phosphocholine |
| 3 | WKY | 1379513_at | transmembrane protein 30B | *Tmem30b* | BE108949 | transmembrane protein; unknown function |
| 3 | WKY | 1380123_at | transmembrane protein 79 | *Tmem79* | BG374569 | transmembrane protein; unknown function |
| 3 | WKY | 1398551_at | tetratricopeptide repeat domain 25 | *Ttc25* | AA997115 | structural protein |
| 3 | WKY | 1385609_at | uroplakin 2 | *Upk2* | BF405879 | structural protein |
| 5 | GK | 1394839_at | phosphoribosylglycinamide formyltransferase | Gart | AA850773 | de novo purine biosynthesis |
| 5 | GK | 1378866_at | actin-binding LIM protein 1 | Ablim1 | BF401176 | cytoskeletal organization |
| 5 | GK | 1374160_at | transmembrane and coiled-coil domain family 2 | *Tmcc2* | BE111296 | transmembrane protein; unknown function |
| 5 | GK | 1381574_at | transmembrane protein 195 | *Tmem195* | BF403907 | transmembrane protein; unknown function |
| 4 | GK | 1387313_at | myocilin | *Myoc* | NM_030865 | extracellular matrix & membrane interaction |
| 4 | GK | *1370350_x_at | urinary protein 2 | *Rup2* | AF368860 | ATPase inhibitor; unknown function |
| 3 | GK | 1372595_at | actinin alpha 2 | *Actn2* | BF284889 | actin cytoskeleton organization |
| 3 | GK | 1398243_at | cysteine and glycine-rich protein 3 | *Csrp3; MLP* | NM_057144 | mechano-sensory stress signalling |
| 3 | GK | 1382618_at | erythrocyte protein band 4.2 | *Epb4.2* | AI103100 | regulation of erythrocyte shape & mechanical property |
| 3 | GK | 1389160_at | erythroid associated factor | *Eraf* | AI230287 | stabilization of alpha-hemoglobin |
| 3 | GK | 1382907_at | family with sequence similarity 46, member C | *Fam46c* | BI289723 | transmembrane protein; unknown function |
| 3 | GK | 1390929_at | filamin, beta | *Flnb* | BI275447 | actin cytoskeleton organization |
| 3 | GK | 1377610_at | leiomodin 2 (cardiac) | *Lmod2* | AI453854 | regulation of actin filament assembly |
| 3 | GK | 1374248_at | myosin binding protein C, slow type | *Mybpc1* | BM389619 | motor activity |
| 3 | GK | *1370971_at | myosin, heavy polypeptide 2, skeletal muscle, adult | *Myh2* | BI277545 | motor activity |
| 3 | GK | *1388139_at | myosin, heavy polypeptide 2, skeletal muscle, adult | *Myh2* | BI277586 | motor activity |
| 3 | GK | *1367928_at | myosin, heavy chain 7, cardiac muscle, beta | *Myh7; Myhcb* | BF395647 | motor activity |
| 3 | GK | *1398248_s_at | myosin, heavy chain 7, cardiac muscle, beta | *Myh7; Myhcb* | NM_017240 | motor activity |
| 3 | GK | *1386993_at | myosin, heavy chain 7, cardiac muscle, beta | *Myh7; Myhcb* | NM_017240 | motor activity |
| 3 | GK | 1388200_at | myosin, light polypeptide 2, regulatory, cardiac, slow | *Myl2; Mlc2* | BF419995 | trafficking of bile salt export protein |
| 3 | GK | 1367572_at | myosin, light chain 3, alkali; ventricular, skeletal, slow | *Myl3; Mylc1v* | NM_012606 | motor activity |
| 3 | GK | 1374049_at | myosin, light chain 6B, alkali, smooth muscle and non-muscle | *Myl6b* | AI230220 | motor activity |
| 3 | GK | 1398821_s_at | myosin light chain kinase 2 | *Mylk2* | AA924075 | motor activity |
| 3 | GK | 1375092_at | myosin XVIIIb | *Myo18b* | AW252664 | motor activity |
| 3 | GK | 1392999_at | neuropilin (NRP) and tolloid (TLL)-like 2 | *Neto2* | AA819279 | transmembrane protein; endocytosis |
| 3 | GK | 1374099_at | nebulin-related anchoring protein | *Nrap* | BM386665 | motor activity |
| Table 2. Cont’d. | | | | | | |
| I. Miscellaneous (76 probe sets, 67 genes) | | | | | | |
| **Age** | **Strain** | **Probe ID** | **Gene Name** | **Symbol** | **Accession** | **Gene Function** |
|  |  |  |  |  |  |  |
| 3 | GK | 1393627_at | ovary-specific acidic protein | *Osap* | AA957929 | transmembrane protein; unknown function |
| 3 | GK | 1372745_at | PDZ and LIM domain 5 | *Pdlim5; Enh* | BE112453 | scaffolding protein |
| 3 | GK | 1389007_at | similar to Friedreich ataxia region gene X123 | *RGD1307524* | AI231799 | transmembrane protein; unknown function |
| 3 | GK | 1380407_at | similar to HTGN29 protein; keratinocytes associated transmembrane protein 2 | *RGD1310352* | AI179886 | transmembrane protein; unknown function |
| 3 | GK | *1370349_a_at | urinary protein 2 | *Rup2* | AF368860 | ATPase inhibitor; unknown function |
| 3 | GK | *1389270_x_at | urinary protein 2 | *Rup2* | AA893518 | ATPase inhibitor; unknown function |
| 3 | GK | 1371554_at | similar to Telethonin (Titin cap protein) | *Tcap* | AA799471 | regulation of muscle assembly |
| 3 | GK | 1386160_at | trichohyalin | *Tchh* | AI639401 | intermediate filament-associated protein; cell envelope organization |
| 3 | GK | 1385751_at | thrombospondin 2 | *Thbs2* | BF408413 | cell-to-cell & cell-to-matrix adhesion |
| 3 | GK | 1376106_at | transmembrane protein 178 | *Tmem178* | AI010157 | transmembrane protein; unknown function |
| 3 | GK | 1371354_at | troponin C, cardiac/slow skeletal | *Tnnc1* | AI710682 | motor activity |
| 3 | GK | *1371339_at | troponin I, skeletal, slow 1 | *Tnni1* | AI599017 | motor activity |
| 3 | GK | *1386873_at | troponin I type 1 (skeletal, slow) | *Tnni1* | NM_017184 | motor activity |
| 3 | GK | 1370412_at | troponin T type 1 (skeletal, slow) | *Tnnt1; Fang2* | AF399874 | motor activity |
| 3 | GK | *1370198_at | triadin | *Trdn* | AJ243304 | calcium release |
| 3 | GK | *1370738_a_at | triadin | *Trdn* | AF220558 | calcium release |
| 3 | GK | 1383211_at | tuftelin 1 | *Tuft1* | BE109736 | mesenchymal stem cell, biomineralizing tissues |
| 3 | GK | 1382539_at | WD repeat domain 37 | *Wdr37* | AI171022 | multiprotein complex formation |
| J. ESTs (167 probe sets) | | | | | | |
| **Age** | **Strain** | **Probe ID** | **Gene Name** | **Symbol** | **Accession** | **Gene Function** |
|  |  |  |  |  |  |  |
| 5 | WKY | 1376840_at | EST | --- | AI103040 | Unknown |
| 5 | WKY | 1376332_at | EST | --- | BF396082 | Unknown |
| 5 | WKY | 1397153_at | EST | --- | BE115262 | Unknown |
| 5 | WKY | 1374558_at | EST | --- | AI010316 | Unknown |
| 5 | WKY | 1392954_at | EST | --- | AI029637 | Unknown |
| 5 | WKY | 1374276_at | EST | --- | BE104102 | Unknown |
| 5 | WKY | 1378413_at | EST | --- | BG379394 | Unknown |
| 5 | WKY | 1380365_at | EST | --- | BF403932 | Unknown |
| 5 | WKY | 1382926_s_at | EST | --- | BF287720 | Unknown |
| 5 | WKY | 1384104_at | EST | --- | BE115892 | Unknown |
| 5 | WKY | 1391990_at | EST | --- | BF405569 | Unknown |
| 5 | WKY | 1392074_at | EST | --- | AA926082 | Unknown |
| 5 | WKY | 1392860_at | EST | --- | AI045117 | Unknown |
| 5 | WKY | 1395260_at | EST | --- | BM383147 | Unknown |
| 5 | WKY | 1395585_at | EST | --- | BG375111 | Unknown |
| 5 | WKY | 1398528_at | EST | --- | BE117514 | Unknown |
| 5 | WKY | 1370377_at | EST | --- | M25143 | Unknown |
| 5 | WKY | 1380670_at | EST | --- | BE095613 | Unknown |
| 5 | WKY | 1381218_at | EST | --- | BF386446 | Unknown |
| 5 | WKY | 1381508_at | EST | --- | BE098143 | Unknown |
| 5 | WKY | 1382437_at | EST | --- | BM383464 | Unknown |
| 5 | WKY | 1391697_at | EST | --- | BM392082 | Unknown |
| 5 | WKY | 1391977_at | EST | --- | AA819560 | Unknown |
| 5 | WKY | 1394732_at | EST | --- | BF289237 | Unknown |
| 5 | WKY | 1394756_at | EST | --- | AA955494 | Unknown |
| 5 | WKY | 1395014_at | EST | --- | BI282068 | Unknown |
| 5 | WKY | 1395198_at | EST | --- | BE099956 | Unknown |
| 5 | WKY | 1395714_at | EST | --- | AT005664 | Unknown |
| 5 | WKY | 1396779_at | EST | --- | BF402702 | Unknown |
| 5 | WKY | 1396877_at | EST | --- | AI715187 | Unknown |
| 5 | WKY | 1386855_at | EST | --- | AA944136 | Unknown |
| 4 | WKY | 1377407_at | EST | --- | BI290154 | Unknown |
| 4 | WKY | 1374583_at | EST | --- | BI288131 | Unknown |
| 4 | WKY | 1380264_at | EST | --- | BF399532 | Unknown |
| 4 | WKY | 1382987_at | EST | --- | AA874945 | Unknown |
| 4 | WKY | 1396253_at | EST | --- | BE116294 | Unknown |
| 4 | WKY | 1371262_at | EST | --- | AJ391299 | Unknown |
| 4 | WKY | 1373900_at | EST | --- | BI284344 | Unknown |
| 4 | WKY | 1376226_at | EST | --- | BG377636 | Unknown |
| 4 | WKY | 1378458_at | EST | --- | BI281745 | Unknown |
| 4 | WKY | 1380956_at | EST | --- | BG672418 | Unknown |
| 4 | WKY | 1385343_at | EST | --- | AW523620 | Unknown |
| 4 | WKY | 1385647_at | EST | --- | AA875123 | Unknown |
| Table 2. Cont’d. | | | | | | |
| J. ESTs (167 probe sets) | | | | | | |
| **Age** | **Strain** | **Probe ID** | **Gene Name** | **Symbol** | **Accession** | **Gene Function** |
|  |  |  |  |  |  |  |
| 4 | WKY | 1390654_at | EST | --- | AA874809 | Unknown |
| 4 | WKY | 1391305_at | EST | --- | AI576233 | Unknown |
| 4 | WKY | 1394554_at | EST | --- | BF564500 | Unknown |
| 4 | WKY | 1398050_at | EST | --- | BI300450 | Unknown |
| 3 | WKY | 1381590_at | EST | --- | BI295719 | Unknown |
| 3 | WKY | 1386466_at | EST | --- | H33093 | Unknown |
| 3 | WKY | 1391757_at | EST | --- | AA926072 | Unknown |
| 3 | WKY | 1395762_at | EST | --- | BG670091 | Unknown |
| 3 | WKY | 1372481_at | EST | --- | AI102873 | Unknown |
| 3 | WKY | 1374345_at | EST | --- | AI111707 | Unknown |
| 3 | WKY | 1375699_at | EST | --- | BM389190 | Unknown |
| 3 | WKY | 1376292_at | EST | --- | BM383423 | Unknown |
| 3 | WKY | 1376635_at | EST | --- | BF283341 | Unknown |
| 3 | WKY | 1378624_at | EST | --- | BE107074 | Unknown |
| 3 | WKY | 1380047_at | EST | --- | AI500979 | Unknown |
| 3 | WKY | 1380209_at | EST | --- | AI176102 | Unknown |
| 3 | WKY | 1380531_at | EST | --- | AI102178 | Unknown |
| 3 | WKY | 1380701_at | EST | --- | BF400722 | Unknown |
| 3 | WKY | 1380782_at | EST | --- | AW528654 | Unknown |
| 3 | WKY | 1381328_at | EST | --- | AI177373 | Unknown |
| 3 | WKY | 1383058_at | EST | --- | AI410438 | Unknown |
| 3 | WKY | 1383163_at | EST | --- | AA817898 | Unknown |
| 3 | WKY | 1383836_at | EST | --- | BM389408 | Unknown |
| 3 | WKY | 1383864_at | EST | --- | BF412512 | Unknown |
| 3 | WKY | 1384073_at | EST | --- | BF543577 | Unknown |
| 3 | WKY | 1384236_at | EST | --- | BI281089 | Unknown |
| 3 | WKY | 1384271_at | EST | --- | BI291798 | Unknown |
| 3 | WKY | 1384667_x_at | EST | --- | AI385327 | Unknown |
| 3 | WKY | 1386383_at | EST | --- | AI639186 | Unknown |
| 3 | WKY | 1390481_a_at | EST | --- | AW531714 | Unknown |
| 3 | WKY | 1390790_a_at | EST | --- | AA964219 | Unknown |
| 3 | WKY | 1390890_at | EST | --- | AI406854 | Unknown |
| 3 | WKY | 1391875_at | EST | --- | AI176106 | Unknown |
| 3 | WKY | 1392037_at | EST | --- | BM383048 | Unknown |
| 3 | WKY | 1392776_at | EST | --- | AI058733 | Unknown |
| 3 | WKY | 1393187_at | EST | --- | BF417625 | Unknown |
| 3 | WKY | 1393440_at | EST | --- | AI071835 | Unknown |
| 3 | WKY | 1395112_at | EST | --- | AI555810 | Unknown |
| 3 | WKY | 1395275_at | EST | --- | BM390168 | Unknown |
| 3 | WKY | 1395888_at | EST | --- | BF559498 | Unknown |
| 3 | WKY | 1396201_at | EST | --- | BF543295 | Unknown |
| 3 | WKY | 1396240_at | EST | --- | AI575096 | Unknown |
| 3 | WKY | 1396410_at | EST | --- | BE116258 | Unknown |
| 3 | WKY | 1396542_at | EST | --- | BF393011 | Unknown |
| 3 | WKY | 1397206_at | EST | --- | AI548120 | Unknown |
| 3 | WKY | 1397218_at | EST | --- | BF420803 | Unknown |
| 3 | WKY | 1397229_at | EST | --- | BF565781 | Unknown |
| 3 | WKY | 1397296_at | EST | --- | AI716498 | Unknown |
| 3 | WKY | 1397435_at | EST | --- | BF394050 | Unknown |
| 3 | WKY | 1398057_at | EST | --- | AI716292 | Unknown |
| 5 | GK | 1393476_at | EST | --- | AI717594 | Unknown |
| 5 | GK | 1389935_at | EST | --- | BI300457 | Unknown |
| 5 | GK | 1380165_at | EST | --- | AI234713 | Unknown |
| 5 | GK | 1371776_at | EST | --- | AA819268 | Unknown |
| 5 | GK | 1381626_at | EST | --- | BE105470 | Unknown |
| 5 | GK | 1384058_at | EST | --- | AI059089 | Unknown |
| 5 | GK | 1379890_at | EST | --- | AI547434 | Unknown |
| 5 | GK | 1380880_at | EST | --- | AA900583 | Unknown |
| 5 | GK | 1384054_at | EST | --- | BF556666 | Unknown |
| 5 | GK | 1391138_at | EST | --- | BF410536 | Unknown |
| 5 | GK | 1392304_at | EST | --- | BI295865 | Unknown |
| 5 | GK | 1395795_at | EST | --- | AW528651 | Unknown |
| 5 | GK | 1398566_at | EST | --- | AW526014 | Unknown |
| 5 | GK | 1398720_at | EST | --- | BI289222 | Unknown |
| 4 | GK | 1372239_at | EST | --- | AW527228 | Unknown |
| 4 | GK | 1373873_at | EST | --- | AI175346 | Unknown |
| Table 2. Cont’d. | | | | | | |
| J. ESTs (167 probe sets) | | | | | | |
| **Age** | **Strain** | **Probe ID** | **Gene Name** | **Symbol** | **Accession** | **Gene Function** |
|  |  |  |  |  |  |  |
| 4 | GK | 1376550_at | EST | --- | BF405084 | Unknown |
| 4 | GK | 1377873_at | EST | --- | AW251736 | Unknown |
| 4 | GK | 1380167_at | EST | --- | BE116526 | Unknown |
| 4 | GK | 1381407_at | EST | --- | AA900580 | Unknown |
| 4 | GK | 1381620_at | EST | --- | BF389656 | Unknown |
| 4 | GK | 1381747_at | EST | --- | BM385105 | Unknown |
| 4 | GK | 1383272_at | EST | --- | BI289784 | Unknown |
| 4 | GK | 1384717_at | EST | --- | AA894199 | Unknown |
| 4 | GK | 1389436_at | EST | --- | AI236615 | Unknown |
| 4 | GK | 1395242_at | EST | --- | BF419005 | Unknown |
| 4 | GK | 1395361_at | EST | --- | AW142913 | Unknown |
| 4 | GK | 1395703_at | EST | --- | H34760 | Unknown |
| 3 | GK | 1375924_at | EST | --- | AA892778 | Unknown |
| 3 | GK | 1391793_at | EST | --- | BF284687 | Unknown |
| 3 | GK | 1392504_at | EST | --- | BI297184 | Unknown |
| 3 | GK | 1393751_at | EST | --- | AA859029 | Unknown |
| 3 | GK | 1374710_at | EST | --- | BI289549 | Unknown |
| 3 | GK | 1376929_at | EST | --- | BF544829 | Unknown |
| 3 | GK | 1377529_at | EST | --- | BF404045 | Unknown |
| 3 | GK | 1378062_at | EST | --- | BF398677 | Unknown |
| 3 | GK | 1379047_at | EST | --- | AW251632 | Unknown |
| 3 | GK | 1379534_at | EST | --- | AI104922 | Unknown |
| 3 | GK | 1379707_at | EST | --- | BF284363 | Unknown |
| 3 | GK | 1379859_at | EST | --- | AA964506 | Unknown |
| 3 | GK | 1380418_at | EST | --- | BF390432 | Unknown |
| 3 | GK | 1380632_at | EST | --- | BE112962 | Unknown |
| 3 | GK | 1381166_at | EST | --- | AA944650 | Unknown |
| 3 | GK | 1382389_at | EST | --- | AI179755 | Unknown |
| 3 | GK | 1382936_at | EST | --- | BF396501 | Unknown |
| 3 | GK | 1382966_at | EST | --- | AI011717 | Unknown |
| 3 | GK | 1383672_at | EST | --- | AI716973 | Unknown |
| 3 | GK | 1383802_at | EST | --- | AW531948 | Unknown |
| 3 | GK | 1384334_at | EST | --- | AI547761 | Unknown |
| 3 | GK | 1384598_at | EST | --- | AA894105 | Unknown |
| 3 | GK | 1385096_at | EST | --- | BG380261 | Unknown |
| 3 | GK | 1386333_at | EST | --- | AI044097 | Unknown |
| 3 | GK | 1389486_at | EST | --- | BE108354 | Unknown |
| 3 | GK | 1389905_at | EST | --- | AI175728 | Unknown |
| 3 | GK | 1390491_at | EST | --- | BM390772 | Unknown |
| 3 | GK | 1390971_at | EST | --- | AI171284 | Unknown |
| 3 | GK | 1391089_at | EST | --- | AW527270 | Unknown |
| 3 | GK | 1391295_at | EST | --- | AI136393 | Unknown |
| 3 | GK | 1392026_at | EST | --- | BF391823 | Unknown |
| 3 | GK | 1392794_at | EST | --- | AA893579 | Unknown |
| 3 | GK | 1393018_at | EST | --- | AI071984 | Unknown |
| 3 | GK | 1394020_at | EST | --- | AI412164 | Unknown |
| 3 | GK | 1394501_at | EST | --- | BM390379 | Unknown |
| 3 | GK | 1394671_at | EST | --- | AI454769 | Unknown |
| 3 | GK | 1395142_at | EST | --- | BG371864 | Unknown |
| 3 | GK | 1395680_at | EST | --- | BF399925 | Unknown |
| 3 | GK | 1395732_at | EST | --- | BF567236 | Unknown |
| 3 | GK | 1396481_at | EST | --- | AW918413 | Unknown |
| 3 | GK | 1396917_at | EST | --- | AI535209 | Unknown |
| 3 | GK | 1396952_at | EST | --- | BF404446 | Unknown |
| 3 | GK | 1397215_at | EST | --- | BF393080 | Unknown |
| 3 | GK | 1397216_at | EST | --- | BF288606 | Unknown |
| 3 | GK | 1397366_at | EST | --- | AI410878 | Unknown |
| 3 | GK | 1397449_at | EST | --- | AI059353 | Unknown |

Column labeled “Strain” indicates the strain which shows higher relative expression of that probe set.

Probe sets marked with an asterisk ( * ) indicates duplicate probe sets for a gene.
